# A Liquid Chromatography with Tandem Mass Spectrometry-Based Proteomic Analysis of Cells Cultured in DMEM 10% FBS and Chemically Defined Medium Using Human Adipose-Derived Mesenchymal Stem Cells

**DOI:** 10.3390/ijms19072042

**Published:** 2018-07-13

**Authors:** Yoshiki Nakashima, Saifun Nahar, Chika Miyagi-Shiohira, Takao Kinjo, Naoya Kobayashi, Issei Saitoh, Masami Watanabe, Jiro Fujita, Hirofumi Noguchi

**Affiliations:** 1 Department of Regenerative Medicine, Graduate School of Medicine, University of the Ryukyus, Okinawa 903-0215, Japan; nakasima@med.u-ryukyu.ac.jp (Y.N.); chika@med.u-ryukyu.ac.jp (C.M.-S.); 2 Department of Infectious, Respiratory, and Digestive Medicine, Graduate School of Medicine, University of the Ryukyus, Okinawa 903-0215, Japan; snaharmd@gmail.com (S.N.); fujita@med.u-ryukyu.ac.jp (J.F.); 3 Department of Basic Laboratory Sciences, School of Health Sciences in the Faculty of Medicine, University of the Ryukyus, Okinawa 903-0215, Japan; kinjotko@med.u-ryukyu.ac.jp; 4 Okayama Saidaiji Hospital, Okayama 704-8192, Japan; n-kobayashi@saidaiji-hp.or.jp; 5 Division of Pediatric Dentistry, Graduate School of Medical and Dental Science, Niigata University, Niigata 951-8514, Japan; isaito@dent.niigata-u.ac.jp; 6 Department of Urology, Okayama University Graduate School of Medicine, Dentistry and Pharmaceutical Sciences, Okayama 700-8558, Japan; masami5@md.okayama-u.ac.jp

**Keywords:** adult stem cells, mesenchymal stem cell, regenerative medicine

## Abstract

Human adipose-derived mesenchymal stem cells (hADSCs) are representative cell sources for cell therapy. Classically, Dulbecco’s Modified Eagle’s medium (DMEM) containing 10% fetal bovine serum (FBS) has been used as culture medium for hADSCs. A chemically defined medium (CDM) containing no heterologous animal components has recently been used to produce therapeutic hADSCs. However, how the culture environment using a medium without FBS affects the protein expression of hADSC is unclear. We subjected hADSCs cultured in CDM and DMEM (10% FBS) to a protein expression analysis by tandem mass spectrometry liquid chromatography and noted 98.2% agreement in the proteins expressed by the CDM and DMEM groups. We classified 761 proteins expressed in both groups by their function in a gene ontology analysis. Thirty-one groups of proteins were classified as growth-related proteins in the CDM and DMEM groups, 16 were classified as antioxidant activity-related, 147 were classified as immune system process-related, 557 were involved in biological regulation, 493 were classified as metabolic process-related, and 407 were classified as related to stimulus responses. These results show that the trend in the expression of major proteins related to the therapeutic effect of hADSCs correlated strongly in both groups.

## 1. Introduction

Mesenchymal stem cells (MSCs) [1] are clinically applied as therapeutic cells [2]. They are collected from the bone marrow [3], umbilical cord [4], dental pulp [5,6,7], and adipose tissue [8,9,10,11]. Adipose tissue-derived mesenchymal stem cells (ADSCs) [8,12] are particularly useful as they can be easily collected from the patient’s own subcutaneous fat [13,14,15]. Clinical-grade ADSC isolation methods [16] and culture methods have already been reported at hospitals and treatment facilities around the world according to GMP standards. However, regarding the medium in which ADSCs are cultured, there are few cases in which hospitals and treatment facilities produce their own media, and it is common to purchase and use commercial media.

Generally, therapeutic ADSCs are administered to patients as therapeutic cells isolated from passages 3–5 since contamination of the interstitial vascular fraction (SVF) [17,18,19], a mixture of the immune system cells contained in adipose tissue, by the primary cultured cells to the first two passages has been reported. It has also been reported that ADSCs cultured for a long period of time (more than 6 passages) tend to be genetically abnormal [20,21]. However, our previous study revealed that the secreted protein content decreases as the number of passages increases (in submission). This indicates that the cell function decreases when ADSCs are cultured for a long period of time.

For cells cultured in vitro from primary culture cells, such as ADSCs, Dulbecco’s Modified Eagle’s medium (DMEM) containing 10% fetal bovine serum (FBS) [22,23] has been used classically for many years. However, using media containing components derived from heterologous animals carries a risk of infection. Furthermore, the risk of administering heterologous animal-derived proteins into a patient’s body cannot be disregarded. Therefore, a clinical-grade chemically defined medium (CDM) suitable for therapeutic cells has become commercially available. Human albumin (derived from human serum [24] or recombinant protein) and growth factor protein [25] are also added to these media in large quantities, reportedly resulting in the promotion of cell proliferation when ADSCs are cultured in CDM [15].

However, a high cell proliferative capacity is not the only indicator to consider when selecting culture media for ADSC. Because functions required for the therapeutic cells ADSCs are therapeutic cells, they are involved in activities such as immunoregulation [26,27] and the secretion of growth factors [27]; as such, high-quality therapeutic ADSCs must have superior immune regulatory ability and growth factor secretion ability. A culture medium capable of producing such high-quality ADSCs can be said to be an excellent medium for therapeutic ADSCs. In this study, the protein components of ADSCs cultured in DMEM containing 10% FBS and CDM were identified using a liquid chromatography with tandem mass spectrometry (LC-MS/MS) protein analysis. We then classified the functions of the identified proteins by gene ontology (GO) [28,29]. These functions were examined, and the functions of ADSCs cultured in two kinds of media were compared.

## 2. Results

### 2.1. The Characteristics and Cell Quality of hADSCs Cultured in CDM

**Figure 1:**
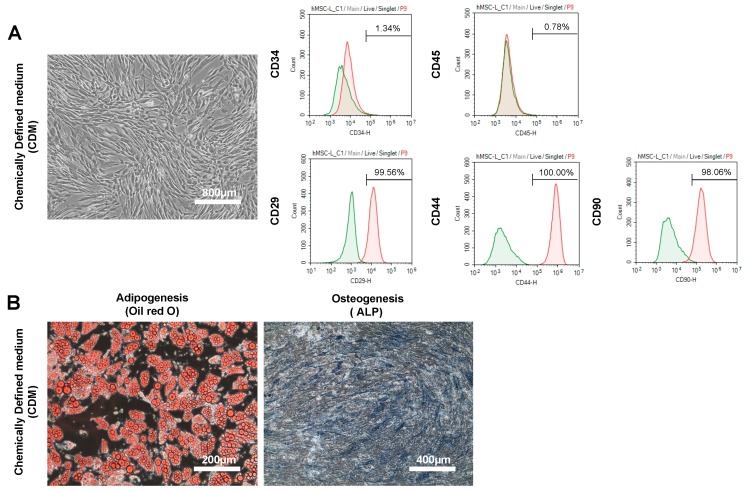

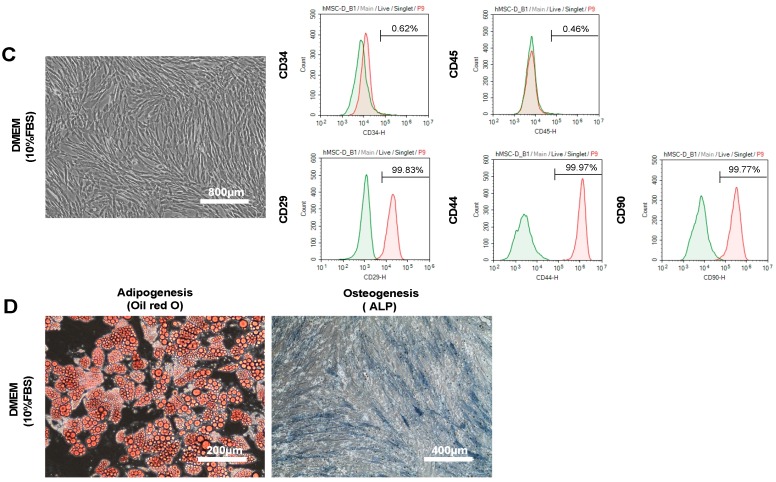
Phenotype and differentiation potential of hADSCs in culture chemically defined medium (CDM) or Dulbecco’s Modified Eagle’s medium (DMEM). The morphological appearance of CDM human Adipose tissue-derived mesenchymal stem cells (hADSCs) ((**A**), left panel) and cell surface markers of CDM hADSCs by flow cytometry. *n* = 3 ((**A**), right panels). Representative images of adipocyte. *n* = 3 ((**B**), left panel) and osteocyte differentiation. *n* = 3 ((**B**), right panel) from CDM hADSCs cultured in differentiation medium. The morphological appearance of DMEM hADSCs ((**C**), left panel) and cell surface markers of DMEM hADSCs by flow cytometry. *n* = 3 ((**C**), right panels). Representative images of adipocyte. *n* = 3 ((**D**), left panel) and osteocyte differentiation. *n* = 3 ((**D**), right panel) from DMEM hADSCs cultured in differentiation medium.

Human ADSCs (hADSCs) were cultured to 80% confluence using CDM. The whole medium was exchanged every two days. We observed no abnormalities in the cell size, shape, or culture state with a normal microscope (Figure 1A, left panel). Flow cytometry was performed using markers of hADSCs (CD29, CD44 and CD90.2), hematopoietic stem cells (CD34) and leukocytes (CD45). CD29, CD44 and CD90.2 were expressed in hADSCs, while CD34 and CD45 were not detected (Figure 1A, right panels).

hADSCs were seeded onto a six-well plate and cultured in the CDM for four days. The cells were confirmed to be confluent on day 4 of seeding, and differentiation induction of ADSC was started using differentiation induction medium. We measured the amount of CD34 expressed on ADSCs cultured using CDM and DMEM (10% FBS) three times using flow cytometry. The relative mean fluorescence intensity (MFI) staining (specific antibody staining vs. IgG-control) of CD34 expression was 1.34, 1.49, and 2.17 in CDM and 0.62, 1.03, and 1.14 in DMEM (10% FBS). The CD34 expression of hADSCs cultured in CDM tended to be higher than that in hADSCs cultured in DMEM, but not to a significant degree. In contrast, neither the CD34 nor CD45 mRNA expression was detected using polymerase chain reaction (PCR) (50 cycles) in hADSCs cultured in CDM or DMEM (10% FBS) (Figure S2C). We induced differentiation into adipocytes (Figure 1B, left panel) and osteoblasts (Figure 1B, right panel) using hADSCs cultured in CDM. Mature adipocytes were stained with Oil Red O, and mature osteoblasts were stained with alkaline phosphatase (ALP). hADSCs were cultured in three wells of a six-well plate. Adipocytes stained red with Oil Red O staining in all three wells and osteoblasts stained blue with alkaline phosphatase staining in all three wells were confirmed with a normal microscope. The induction period of differentiation into adipocytes was 20–30 days. The induction period of osteoblast differentiation was 14–21 days. To investigate the effect of CDM on the induction of initial differentiation of adipocytes, hADSCs cultured with both CDM and DMEM (10% FBS) were used to induce differentiation of adipocytes and the expression of adipocyte differentiation marker mRNA on day 4. The expression of adipocyte differentiation markers (peroxisome proliferator-activated receptor γ (PPARγ) [30], fatty acid binding protein 4 (FABP4) [31,32,33], and CCAAT/enhancer binding protein α (C/EBPα) [34]) was assessed, with the expression of β-actin as a housekeeping gene set as 1. The expression of C/EBPα is known to not be increased by day 4 of induction of adipocyte differentiation [35]. The results showed that the mRNA expression of FABP4, an early differentiation marker, in hADSCs cultured in CDM was significantly lower than in those cultured in DMEM (10% FBS) (Figure S2D).

Next, we examined the relationship of cell proliferation-regulating proteins with nuclear factor-kappa B (NF-κB), argininosuccinate synthase (ASS1, which is regulated by HIF-1α), and the c-Myc transcription network [36] or integrin α-5 (ITGA5), which is known to promote the proliferation and inhibit the differentiation of hADSCs [37]. Our results showed that the expression of ITGA5 mRNA cultured in CDM was about 70% of that of hADSCs cultured in DMEM (10% FBS). The p50 and p65 constituent proteins of NF-κB and the mRNA expression of ASS1 were lower in hADSCs cultured with CDM than in those cultured in DMEM (10% FBS) (Figure S2C).

### 2.2. The Characteristics and Cell Quality of hADSCs Cultured in DMEM Containing 10% FBS

hADSCs were cultured to 80% confluence using DMEM containing 10% FBS. The whole medium was exchanged every two days. The passage of cells was performed every 3 to 4 days after reaching 80% confluence. We observed no abnormalities in cell size, shape, or culture state with a normal microscope (Figure 1C, left panel). Flow cytometry was performed using markers of hADSCs (CD44, CD90.2), hematopoietic stem cells (CD34), and leukocytes (CD45). CD29, CD44, and CD90.2 were expressed in hADSCs, while CD34 and CD45 were not detected (Figure 1C, right panels). The expression of CD29, CD44, and CD90, which are surface markers of hADSCs, was higher in hADSCs cultured in DMEM (10% FBS) than in those cultured in CDM (Figure S2C). In addition, hADSCs cultured with DMEM (10% FBS) had a slightly higher cell number on days 2 and 4 after cell seeding than those cultured using CDM (Figure S2A). When viable cell activity was measured 4 days after seeding using the MTT assay method, the viable cell activity of hADSCs cultured in DMEM (10% FBS) was significantly higher than in those cultured in CDM (Figure S2B). Furthermore, the mRNA expression of cell proliferation markers (Ki67 and proliferating cell nuclear antigen (PCNA)) on hADSCs cultured with DMEM (10% FBS) was also higher than in those cultured in CDM (Figure S2C).

hADSCs were seeded into six-wells plates and cultured in DMEM (10% FBS) for 4 days. The cells were confirmed to be confluent on day 4 of seeding, and differentiation induction of ADSC was started using differentiation induction medium. We induced differentiation into adipocytes (Figure 1D, left panel) and osteoblasts (Figure 1D, right panel) using hADSCs cultured in DMEM containing 10% FBS. Mature adipocytes were stained with Oil Red O, and mature osteoblasts were stained with ALP. hADSCs were cultured in three wells of a six-well plate. Adipocytes stained red with Oil Red O staining in all three wells and osteoblasts stained blue with alkaline phosphatase staining in all three wells were confirmed with a normal microscope. The induction period of differentiation into adipocytes was 20–30 days. The induction period of osteoblast differentiation was 14–21 days.

### 2.3. A Comprehensive Protein Expression Analysis of hADSCs (CDM And DMEM)

A sample of the CDM group was obtained at a concentration of 3541 μg/mL. The DMEM group samples were obtained at a concentration of 3800 μg/mL. Nanoflow LC-MS/MS and a database search (Mascot analysis) were conducted by Ikuko Sagawa of “Support Center for Advanced Medical Sciences, Tokushima University Graduate School of Biomedical Sciences”. The data obtained by LC-MS/MS were quantified by the theoretical value (emPAI) [38,39,40,41] estimated based on the function of the Scaffold software program. Table S1 lists all proteins (1716 types) detected in the CDM group. Table S2 lists all proteins (1745 types) detected in the DMEM group. The numerical value of emPAI is shown on the right end of Tables S1 and S2. The ratio of the number of measured peptides to the number of theoretical peptides was linearly related to the logarithm of the protein concentration, and the number obtained by subtracting 1 from the index of the peptide number ratio was defined as emPAI. The larger the emPAI value, the greater the amount of protein. A shotgun method was used for the proteome analysis. The protein was digested with trypsin or the like to obtain a peptide mixture, and then the protein was identified from the amino acid sequence information. The detected amino acid sequence was then searched for using the online protein database. However, in contrast to the PCR method, peptides, which are sample proteins and degradation products of sample proteins, cannot be amplified using measuring equipment. The weaknesses of this proteome analysis are supplemented by highly accurate detection technology. There is a possibility that non-specific detection may occur when reading the information regarding the amino acid sequence. The most likely cause of this was considered to be the structure of heterologous proteins and changes in the protein structure due to genetic mutations, such as SNP. Therefore, predicting the presence of protein just because one short peptide sequence is detected is a scientifically unstable assertion. We must instead detect many peptide regions and a large number of peptide fragments constituting proteins using liquid chromatography with tandem mass spectrometry (LC-MS/MS) in order to assert the existence of proteins based on scientific evidence.

**Figure 2:**
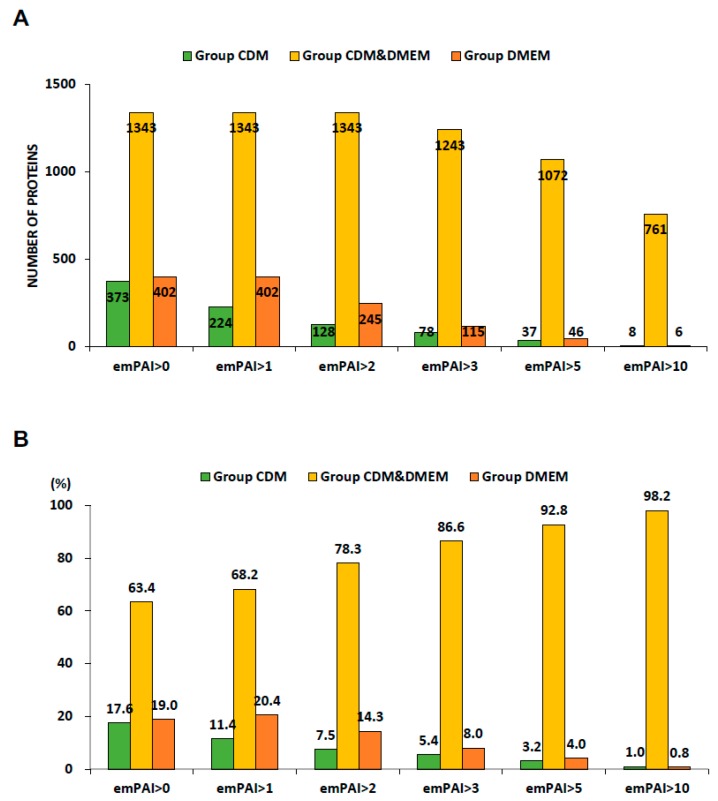
Bar graph of proteins detected on LC-MS/MS. Top panel, there were 1716–769 proteins identified from the CDM hADSCs sample and 1745–767 proteins identified from the DMEM hADSCs (emPAI > 0–10). *n* = 1 (**A**). Lower panel, 63.4–98.2% of proteins were identified in the CDM & DMEM hADSCs (emPAI > 0–10). *n* = 1 (**B**).

Figure 2 shows the types of proteins detected in the range from emPAI > 0 to > 10 (Figure 2A), presented as the percentage of the protein fraction (Figure 2B). As a result, when emPAI > 1 was compared to emPAI > 0, 149 types of proteins detected in the CDM group were deleted. However, when emPAI > 0 was compared to emPAI > 2, 1343 types of proteins were detected in both the CDM and DMEM groups. For emPAI > 10, 761 (98.2%) types of proteins were detected in both groups, while 8 types (1.0%) were detected in the CDM group and 6 types (0.8%) in the DMEM group. These results indicate that 56.7% of the proteins detected in both groups at emPAI > 0 were also detected at emPAI > 10.

In addition, the housekeeping inheritance (TFRC, YWHAZ, RPLP0, RPLP1, RPLP2, B2M, RPS18, PGK1, PPIA, and GAPDH) was assessed. We found that the proteins detected at emPAI > 10 were derived from the cellular components of the sample. As background information, the present study used ADSCs, one sample at a time, cultured in different media as sources for the protein analysis. It is therefore necessary to consider the data reliability. In order to keep the reliability of the data as high as possible, we performed a protein analysis focusing on the proteins detected at high concentrations using an emPAI value > 10 in this paper.

### 2.4. Quantitative Values of hADSCs (Group CDM & DMEM) (emPAI Corrected by the Expression Level of Housekeeping Gene)

**Figure 3:**
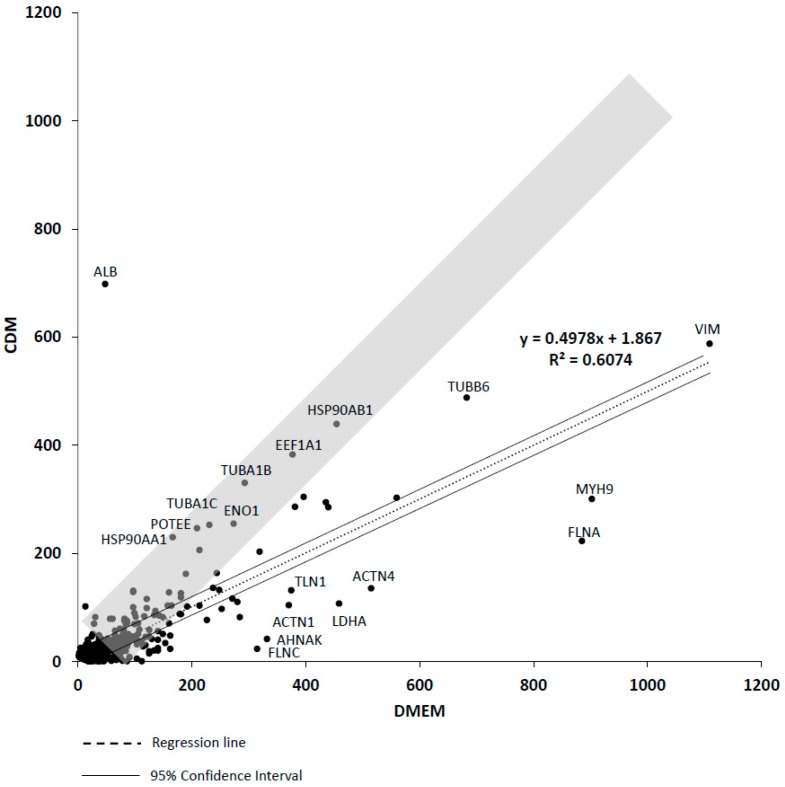
A scatter plot of the quantitative value (normalized emPAI) per housekeeping gene. A scatter plot showing correlation (R^2^ = 0.6074; gray band indicates “R^2^ = 1”) between the quantitative value of the CDM hADSCs and DMEM hADSCs (*n* = 1139). The dotted line is the regression line. The two lines indicate the 95% confidence interval. Each dot shows the abbreviated name of the protein. *n* = 1.

The quantitative values of the proteins expressed in both CDM hADSCs and DMEM hADSCs were represented with a scatter plot (*y*-axis = CDM, *x*-axis = DMEM). The average quantitative value of CDM-expressed proteins decreased to 53.4% compared to DMEM-expressed proteins (Figure 3) (housekeeping genes: TFRC, YWHAZ, RPLP0, RPLP1, RPLP2, B2M, RPS18, PGK1, PPIA, and GAPDH; Supplementary Figure S1). The quantitative values of ALB, TUBB6, HSP90AB1, EEF1A1, TUBA1B, ENO1, TUBA1C, POTEE. and HSP90AA1 were higher in CDM hADSCs than in DMEM hADSCs. In contrast, the quantitative values of MYH9, FLNA, ACTN4, LDHA, TLN1, LDHA, ACTN1, AHNAK, and FLNC were higher in DMEM hADSCs than in CDM hADSCs (Figure 3). The CDM we used in this study is MSCGM-CDTM Mesenchymal Stem Cell Growth Medium BulletKit™ (Lonza, Basel, Switzerland). This medium has been approved by the Japanese Ministry of Health, Labor and Welfare as a clinical cell culture medium for human beings. In the LC-MS/MS protein analysis of hADSCs, not only the proteins contained in cells but also the human proteins contained initially in the media sold by manufacturers are detected. However, it is impossible to judge whether the identified protein is an hADSC-secreted protein or a medium constituent protein. Therefore, we examined the human protein contained in the medium from the beginning to prepare for this study. The protein components are as follows: ALB, TRFE, HPT, A1BG, HEMO, FETUA, HPTR, PGRP2, ITIH4, AFAM, TTHY, APOH, VTDB, ZA2G, A2GL, IGKC, IGLC2, C1R, IGLL5, CERU, RET4, A1AG2, ATRN, IGHG1, CPN2, HBB, HBA, AMBP, APOD, A1AG1, DYH5, CFAI, IC1, C1RL, THBG, AGRF4, KNG1, FETUB, MYO5B, CF163, 5NT3B, FA11, KLKB1, and LCAT. Therefore, the human ALB that was detected in large amounts in the CDM group in our measurement was considered to be a constituent protein component of the commercial medium.

### 2.5. Gene ontology (GO) Classification of Proteins Expressed in CDM and DMEM hADSCs

**Figure 4:**
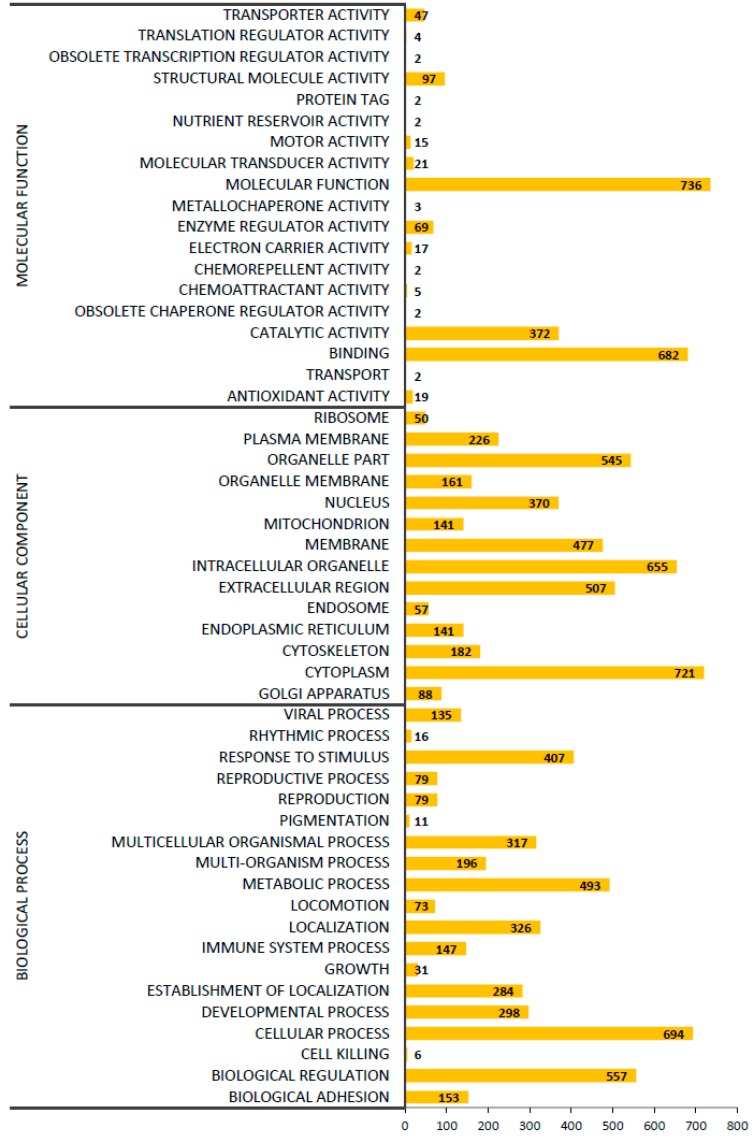
The biological processes, cellular components, and molecular functions of the CDM & DMEM hADSCs (as determined by the GO analysis). The ordinate indicates the biological function, cellular component, and molecular function of each protein. The abscissa indicates the number of identified proteins in the CDM & DMEM hADSCs. The names of the proteins classified in Tables S3, S5, S6, S7 and S8 are listed by their detailed biological process (growth, immune system process, biological regulation, metabolic process, and response to stimulus). The names of the proteins classified in Table S4 are listed by their detailed molecular function (antioxidant activity). *n* = 1.

Using the Gene Ontology Consortium (http://www.geneontology.org/) database, the detected proteins were classified into three subcategories based on their function: biological processes, cellular components, and molecular functions. The proteins expressed in CDM and DMEM hADSCs were classified into the both CDM and DMEM group (Figure 4). The molecular functions classification is shown in the top panel of Figure 4, that of the cellular components in the middle panel of Figure 4, and that of the biological processes in the bottom panel of Figure 4.

## 3. Discussion

In this study, the proteins expressed only in the CDM group were POTEKP, TAGLN3, TF, MT2A (a key protein involved in endothelial cell proliferation and migration [42]), HP, DHCR24 (a key protein involved in cell homeostasis and cholesterol biosynthesis [43]), NQO1 (a key protein involved in cellular adaptation to stress [44]), and TRAP1 (a key protein of the molecular chaperone involved in the regulation of energetic metabolism in cancer cells [45]). The proteins expressed only in the DMEM group were PDLIM5 (proteins phosphorylated by AMPK activation that suppress cell migration [46]), PDLIM7 (a key protein of scaffolds for the formation of multiprotein complexes [47]), Integrin alpha 5 (ITGA5), ASS1 (an enzyme involved in the clearance of nitrogenous waste via the urea cycle and de novo arginine biosynthesis [48]), FHL1 (inhibits the growth of cancer cells via G1/S cell cycle arrest [49]), and voltage-dependent anion channel 3 (VDAC3). These results indicate that the proteins specifically expressed in the CDM and DMEM groups included proteins involved in cell adhesion, cell migration, cell cycle regulation, and lipid metabolism. However, none of these proteins was considered to be an immune regulatory cytokine, even though immune regulation is a major function of hADSCs as therapeutic cells.

The expression of the *housekeeping gene* (*HKG*) protein of the CDM group was 2.00 times that of the DMEM group. Therefore, the mean value of the total protein expression of CDM after correction for the *HKG* expression was 53.4% compared with the DMEM group (Figure 3).

Thirty-one kinds of proteins involved in the “growth”-related biological processes as classified by the GO analysis were investigated (Figure 4). Following correction with *HKG*, the emiPAI values of both myotrophin (MTPN; promotes dimerization of NF-κB subunits and regulates NF-κB transcription factor activity [50]) and calcium-dependent phospholipid-binding protein (CPNE1) were higher in the CDM group than in the DMEM group (Table S3). This likely affected the NF-κB signaling of CPNE1. STRING web software is a database (STRING: https://string-db.org) for predicting mutual protein binding using computer simulation. STRING web software predicted protein-protein binding of CPNE1 protein and NFKB1 protein (https://string-db.org/network/9606.ENSP00000317257). Future studies are expected to explore this matter in further detail. Previous studies have reported that NF-κB signaling suppresses the osteogenic differentiation of MSCs [51]. In addition, NF-κB controls cell growth and differentiation through the transcriptional regulation of cyclin D1 [52,53]. These findings therefore suggest that hADSCs cultured in CDM may promote cell proliferation by activating NF-κB signaling.

One limitation of this study is the insufficient patient numbers. All analyses were performed with hADSCs from the same patient, as the hADSCs used in this study were cells explicitly provided for testing, such as gene transfer, functional genome, drug screening, high-speed screening, and toxicology, and quality assurance tests have been conducted (Certificate of Analysis: Lot number 394Z027.1). While we agree that any research results obtained using hADSCs for testing also reflect donor-specific features, it should be noted that our previous paper found that culture in CDM showed no impairment in the ability of hADSCs to differentiate into adipocytes and osteoblasts [15].

The culture of hADSCs in CDM promotes cell proliferation without impairing the expression of the cell surface markers and the differentiation-inducing ability. Furthermore, our proteome analysis presumes that the increased cell proliferation ability acquired by hADSCs cultured in CDM is caused by the activation of NF-κB signaling. In addition, our LC-MS/MS analysis showed that, the 98.2% of the proteins expressed by hADSCs cultured in CDM were also expressed by hADSCs cultured in DMEM containing 10% FBS. The 1.8% of proteins with inconsistent expression, included no immune regulators or growth factors. These results show that hADSCs cultured in CDM were of sufficient quality for use as therapeutic cells. In conclusion, CDM can be safely used as culture medium without impairing the therapeutic cell quality of hADSCs.

## 4. Materials and Methods

### 4.1. Reagents

The MSCGM-CDTM Mesenchymal Stem Cell Growth Medium BulletKit™ was obtained from Lonza (Basel, Switzerland). hADSCs (from a 46-year-old Caucasian female; PromoCell, Heidelberg, Germany) were cultured. FBS was obtained from BioWest (Nuaille, France). DMEM was obtained from Wako Pure Chemical Industries (Osaka, Japan). Plastic dishes were obtained from TPP (Trasadingen, Switzerland). All other materials used were of the highest commercial grade.

### 4.2. Preparation of hADSCs

hADSCs were cultured (37 °C, 5% CO_2_) on a coated 100-mm culture plate (TPP 93100). The passage of cells was performed every 3 to 4 days after reaching 80% confluence after sowing the cells. The cells were washed with phosphate-buffered saline (PBS; calcium, magnesium-free), and hADSCs were dissociated using a dissociation solution. Subculturing was carried out by plating on an uncoated 100-mm culture plate. An MSCGM-CD mesenchymal stem cell BulletKit™ (00190632; Lonza) was used for the culture medium. Trypsin/EDTA (CC-3232; Lonza) was used for the dissociation solution. hADSCs were cultured using different media from the time they were seeded into culture vessels. hADSCs used for all measurements were cultured using a different medium for a minimum of four days.

### 4.3. Flow Cytometry

Cell flow cytometry was performed using a NovoCyte^®^ Flow Cytometer (ACEA Biosciences, Inc., San Diego, CA, USA) according to the manufacturer’s instructions. In brief, hADSCs (1 × 10^5^ cells) were mixed into 0.5 mL of Perfusion Solution (CORNING, Manassas, VA, USA). Each antibody (1/100 of the volume) was added to the cell admixture, which was then incubated on ice for 30 min. After washing the cells with Brilliant Stain Buffer (BD Biosciences, Franklin Lakes, NJ, USA), FACSs measurement was carried out. The following primary antibodies was used: APC Mouse Anti-Human CD29, BV421 Mouse Anti-Human CD44, BV421 Mouse IgG2b κ Isotype Control, APC Mouse IgG1 κ Isotype Control (BD Biosciences); FITC anti-human CD90.2 (Thy1) Antibody, FITC Mouse IgG1 κ Isotype Ctrl Antibody, PerCP anti-human CD34 Antibody, PerCP Mouse IgG1 κ Isotype Ctrl Antibody, PE/Cy7 anti-human CD45 Antibody and PE/Cy7 Mouse IgG1 κ Isotype Ctrl Antibody (BioLegend, Inc., San Diego, CA, USA).

### 4.4. Cell Differentiation

Adipogenic differentiation was performed using Adipogenic Differentiation Medium (DM-2; Zen-Bio, Inc., Research Triangle Park, NC, USA) and a Lipid Assay Kit (AK09F; Cosmo Bio Co., Ltd., Tokyo, Japan) according to the manufacturer’s instructions. The hADSCs that became confluent four days after seeding into six-well plates using the designated medium were cultured for seven days using Adipocyte Differentiation Medium (DM-2; Zen-Bio, Inc.) to induce differentiation into adipocytes. After that, the medium was switched to Adipocyte Maintenance Medium (AM-1; Zen-Bio, Inc.) and changed every three days. Adipocytes with lipid droplets were confirmed under a microscope at 20–30 days from the start of differentiation induction (Detailed Protocol of Adipocyte Differentiation; http://www.zen-bio.com/pdf/ZBM0001.01SQAdipocyteCare.pdf). The composition of the medium was as follows: AM-1 (DMEM/Ham’s F-12 (1:1, *v*/*v*), HEPES pH 7.4, FBS, Biotin, Pantothenate, Human insulin, Dexamethasone, Penicillin, Streptomycin and Amphotericin B) and DM-2 (DMEM/Ham’s F-12 (1:1, *v*/*v*), HEPES pH 7.4, FBS, Biotin, Pantothenate, Human insulin, Dexamethasone, 3-Isobutyl-1-methylxanthine (IBMX), PPARγ agonist, Penicillin, Streptomycin and Amphotericin B). Osteogenic differentiation was performed using Osteoblast Differentiation Medium for Adipose (OB-1; Zen-Bio, Inc.) and a Calcified Nodule Staining Kit (AK21; Cosmo Bio Co., Ltd.) according to the manufacturer’s instructions.

### 4.5. Cell Proliferation Assays

The cells were seeded into 96-well plates. Each well received 1 × 10^5^ cells/mL of medium. The cells were detached with trypsin/EDTA solution at two and four days after cell seeding, and the number of cells was counted according to a conventional method. Cell proliferation was measured using the MTT Cell Count Kit (Nacalai Tesque, Kyoto, Japan) according to the manufacturers’ instructions. In brief, the cells were seeded into 96-well plates. Each well received 1 × 10^5^ cells/mL of medium. Absorbance was measured with a microplate reader at a wavelength of 570 nm.

### 4.6. Real Time PCR and RT-PCR

Cells were cultured in 96-well plates in medium to approximately 80% confluence. RNA was prepared using a SuperPrep Cell Lysis and RT kit for qPCR according to the manufacturer’s instructions (Toyobo Co., Ltd., Osaka, Japan). Real-time PCR analyses were performed using a LightCycler 96 Real-Time PCR system (Roche, Basel, Switzerland). The Luna^®^ Universal qPCR Master Mix was used according to the manufacturer’s instructions (New England BioLabs Inc., Ipswich, MA, USA). For the design of primers other than the primers cited in other papers, the gene names were retrieved from the US National Library of Medicine National Institutes of Health website (https://www.ncbi.nlm.nih.gov/pubmed/). The primers were designed using the Primer 3 Plus application (http://www.bioinformatics.nl/cgi-bin/primer3plus/primer3plus.cgi), and analyses were performed using the primers listed in Table S9.

### 4.7. Protein Identification by a Nano LC-MS/MS Analysis

A protein solution of the CDM group was obtained at a concentration of 3541 μg/mL. The DMEM group samples were obtained at a concentration of 3800 μg/mL. Finally, 0.4 μg of protein was used for nano LC-MS/MS. The samples were analyzed via nano LC using an UltiMate 3000 RSLC nano system (Thermo Fisher Scientific, Tokyo, Japan) at the Support Center for Advanced Medical Sciences, Institute of Biomedical Sciences, Tokushima University Graduate School by Ikuko Sagawa.

In brief, protein-containing solutions were reduced with 10 mM DTT/8 M urea and Tris buffer containing 2 mM EDTA (pH 8.5), alkylated with 25 mM iodoacetamide/8 M Urea and Tris buffer containing 2 mM EDTA (pH 8.5), subsequently diluted with trypsin (pig-derived trypsin) and digested overnight at 37 °C. Peptides were purified and concentrated by solid-phase extraction (SPE) in ZipTip C18 pipette tips (Merck Millipore, Darmstadt, Germany). Nano LC-MS/MS was carried out using an UltiMate 3000 RSLC nano system (Thermo Fisher Scientific). The reconstituted peptides were injected into an Acclaim PepMap C18 trap column (75 μm × 15 cm, 2 μm, C18) (Merck Millipore, Darmstadt, Germany). Solvent A was 0.1% formic acid. Solvent B was 80% acetonitrile/0.08% formic acid. The peptides were eluted in a 229-min gradient of 4% solvent B in solvent A to 90% solvent B in solvent A at 300 nL/min. Orbitrap Elite’s ionization method was set to Nanoflow-LC ESI, positive, and the capillary voltage was set to 1.7 kV.

Tandem mass spectrometry was performed using the Proteome Discoverer software program, version 1.4 (Thermo Fisher Scientific). Charge state deconvolution and deisotoping were not performed.

### 4.8. Data Analyses

• Database searching

Tandem mass spectra were extracted using the Proteome Discoverer software program, version 1.4 (Thermo Fisher Scientific). Charge state deconvolution and deisotoping were not performed. All MS/MS samples were analyzed using the Mascot software program (version 2.5.1; Matrix Science, London, UK). Mascot was set up to search the SwissProt_2017_12 database (unknown version, 556388 entries) assuming the digestion enzyme strict trypsin was used. Mascot was searched with a fragment ion mass tolerance of 0.60 Da and a parent ion tolerance of 5.0 PPM.

• Criteria for protein identification

The comprehensive expression analysis of proteins using LC-MS/MS was performed according to the method reported previously [54]. In brief, the relative abundance of the proteins identified by LC-MS/MS was estimated by determining the protein abundance index (PAI) and the emPAI. Visualized and validated complex LC-MS/MS proteomics experiments were performed using the Scaffold software program (version 4.7.3; Proteome Software Inc., Portland, OR, USA) (http://www.proteomesoftware.com/) to compare samples in order to identify biological relevance. The Scaffold software program was used to validate MS/MS-based peptide and protein identifications. Peptide identifications were accepted if they could be established at greater than 66.0% probability to achieve an FDR of <1.0% using the Scaffold Local FDR algorithm. Protein identifications were accepted if they could be established at greater than 95.0% probability and contained at least one identified peptide. Protein probabilities were assigned by the Protein Prophet algorithm [55]. Proteins that contained similar peptides and could not be differentiated based on an MS/MS analysis alone were grouped to satisfy the principles of parsimony.

• The protein GO analysis

The protein GO analysis was performed using the GO analysis function of the Scaffold 4 software program with imported data (goa_uniprot_all.gaf [downloaded 2016/10/14]) [28] from the external GO Annotation Source database.

### 4.9. Statistical Analyses

Statistical analyses were performed using Student’s *t*-test to compare two samples. Statistical significance was set at * *p* < 0.05 or ** *p* < 0.01 for all tests. The data shown are representative examples of two independent experiments.

## Supplementary Materials

The following are available online at http://www.mdpi.com/1422-0067/19/7/2042/s1.

## References

[1] Wagers A.J., Weissman I.L. (2004). Plasticity of adult stem cells. Cell.

[2] Wei X., Yang X., Han Z.P., Qu F.F., Shao L., Shi Y.F. (2013). Mesenchymal stem cells: A new trend for cell therapy. Acta Pharmacol. Sin..

[3] Ito K., Aoyama T., Fukiage K., Otsuka S., Furu M., Jin Y., Nasu A., Ueda M., Kasai Y., Ashihara E. (2010). A novel method to isolate mesenchymal stem cells from bone marrow in a closed system using a device made by nonwoven fabric. Tissue Eng. Part C Methods.

[4] Shohara R., Yamamoto A., Takikawa S., Iwase A., Hibi H., Kikkawa F., Ueda M. (2012). Mesenchymal stromal cells of human umbilical cord Wharton’s jelly accelerate wound healing by paracrine mechanisms. Cytotherapy.

[5] Song M., Lee J.H., Bae J., Bu Y., Kim E.C. (2017). Human Dental Pulp Stem Cells Are More Effective Than Human Bone Marrow-Derived Mesenchymal Stem Cells in Cerebral Ischemic Injury. Cell Transplant..

[6] Nakashima M., Iohara K. (2011). Regeneration of dental pulp by stem cells. Adv. Dent. Res..

[7] Murakami T., Saitoh I., Inada E., Kurosawa M., Iwase Y., Noguchi H., Terao Y., Yamasaki Y., Hayasaki H., Sato M. (2013). STO Feeder Cells Are Useful for Propagation of Primarily Cultured Human Deciduous Dental Pulp Cells by Eliminating Contaminating Bacteria and Promoting Cellular Outgrowth. Cell Med..

[8] Miyagi-Shiohira C., Kurima K., Kobayashi N., Saitoh I., Watanabe M., Noguchi Y., Matsushita M., Noguchi H. (2015). Cryopreservation of Adipose-Derived Mesenchymal Stem Cells. Cell Med..

[9] Yukawa H., Noguchi H., Oishi K., Takagi S., Hamaguchi M., Hamajima N., Hayashi S. (2009). Cell transplantation of adipose tissue-derived stem cells in combination with heparin attenuated acute liver failure in mice. Cell Transplant..

[10] Yukawa H., Mizufune S., Mamori C., Kagami Y., Oishi K., Kaji N., Okamoto Y., Takeshi M., Noguchi H., Baba Y. (2009). Quantum dots for labeling adipose tissue-derived stem cells. Cell Transplant..

[11] Yukawa H., Nakagawa S., Yoshizumi Y., Watanabe M., Saito H., Miyamoto Y., Noguchi H., Oishi K., Ono K., Sawada M. (2014). Novel positively charged nanoparticle labeling for in vivo imaging of adipose tissue-derived stem cells. PLoS ONE.

[12] Szoke K., Brinchmann J.E. (2012). Concise review: Therapeutic potential of adipose tissue-derived angiogenic cells. Stem Cells Transl. Med..

[13] Seki A., Sakai Y., Komura T., Nasti A., Yoshida K., Higashimoto M., Honda M., Usui S., Takamura M., Takamura T. (2013). Adipose tissue-derived stem cells as a regenerative therapy for a mouse steatohepatitis-induced cirrhosis model. Hepatology.

[14] Ullah I., Subbarao R.B., Rho G.J. (2015). Human mesenchymal stem cells-current trends and future prospective. Biosci. Rep..

[15] Miyagi-Shiohira C., Kobayashi N., Saitoh I., Watanabe M., Noguchi Y., Matsushita M., Noguchi H. (2017). Evaluation of Serum-Free, Xeno-Free Cryopreservation Solutions for Human Adipose-Derived Mesenchymal Stem Cells. Cell Med..

[16] Schneider S., Unger M., van Griensven M., Balmayor E.R. (2017). Adipose-derived mesenchymal stem cells from liposuction and resected fat are feasible sources for regenerative medicine. Eur. J. Med. Res..

[17] Bora P., Majumdar A.S. (2017). Adipose tissue-derived stromal vascular fraction in regenerative medicine: A brief review on biology and translation. Stem Cell Res. Ther..

[18] Han S., Sun H.M., Hwang K.C., Kim S.W. (2015). Adipose-Derived Stromal Vascular Fraction Cells: Update on Clinical Utility and Efficacy. Crit. Rev. Eukaryot. Gene Expr..

[19] Nguyen A., Guo J., Banyard D.A., Fadavi D., Toranto J.D., Wirth G.A., Paydar K.Z., Evans G.R., Widgerow A.D. (2016). Stromal vascular fraction: A regenerative reality? Part 1: Current concepts and review of the literature. J. Plast. Reconstr. Aesthet. Surg..

[20] Gu Y., Li T., Ding Y., Sun L., Tu T., Zhu W., Hu J., Sun X. (2016). Changes in mesenchymal stem cells following long-term culture in vitro. Mol. Med. Rep..

[21] Basciano L., Nemos C., Foliguet B., de Isla N., de Carvalho M., Tran N., Dalloul A. (2011). Long term culture of mesenchymal stem cells in hypoxia promotes a genetic program maintaining their undifferentiated and multipotent status. BMC Cell Biol..

[22] Ben Azouna N., Jenhani F., Regaya Z., Berraeis L., Ben Othman T., Ducrocq E., Domenech J. (2012). Phenotypical and functional characteristics of mesenchymal stem cells from bone marrow: Comparison of culture using different media supplemented with human platelet lysate or fetal bovine serum. Stem Cell Res. Ther..

[23] Hemeda H., Giebel B., Wagner W. (2014). Evaluation of human platelet lysate versus fetal bovine serum for culture of mesenchymal stromal cells. Cytotherapy.

[24] Aldahmash A., Haack-Sorensen M., Al-Nbaheen M., Harkness L., Abdallah B.M., Kassem M. (2011). Human Serum is as Efficient as Fetal Bovine Serum in Supporting Proliferation and Differentiation of Human Multipotent Stromal (Mesenchymal) Stem Cells In Vitro and In Vivo. Stem Cell Rev. Rep..

[25] Mimura S., Kimura N., Hirata M., Tateyama D., Hayashida M., Umezawa A., Kohara A., Nikawa H., Okamoto T., Furue M.K. (2011). Growth factor-defined culture medium for human mesenchymal stem cells. Int. J. Dev. Biol..

[26] Nauta A.J., Fibbe W.E. (2007). Immunomodulatory properties of mesenchymal stromal cells. Blood.

[27] Shi Y.F., Su J.J., Roberts A.I., Shou P.S., Rabson A.B., Ren G.W. (2012). How mesenchymal stem cells interact with tissue immune responses. Trends Immunol..

[28] Ashburner M., Ball C.A., Blake J.A., Botstein D., Butler H., Cherry J.M., Davis A.P., Dolinski K., Dwight S.S., Eppig J.T. (2000). Gene ontology: Tool for the unification of biology. The Gene Ontology Consortium. Nat. Genet..

[29] Huntley R.P., Sawford T., Martin M.J., O’Donovan C. (2014). Understanding how and why the Gene Ontology and its annotations evolve: The GO within UniProt. Gigascience.

[30] Rosen E.D., Hsu C.H., Wang X.Z., Sakai S., Freeman M.W., Gonzalez F.J., Spiegelman B.M. (2002). C/EBP alpha induces adipogenesis through PPAR gamma: A unified pathway. Genes Dev..

[31] Furuhashi M., Hotamisligil G.S. (2008). Fatty acid-binding proteins: Role in metabolic diseases and potential as drug targets. Nat. Rev. Drug Discov..

[32] Furuhashi M., Ishimura S., Ota H., Miura T. (2011). Lipid chaperones and metabolic inflammation. Int. J. Inflamm..

[33] Furuhashi M., Saitoh S., Shimamoto K., Miura T. (2014). Fatty Acid-Binding Protein 4 (FABP4): Pathophysiological Insights and Potent Clinical Biomarker of Metabolic and Cardiovascular Diseases. Clin. Med. Insights Cardiol..

[34] Linhart H.G., Ishimura-Oka K., DeMayo F., Kibe T., Repka D., Poindexter B., Bick R.J., Darlington G.J. (2001). C/EBP alpha is required for differentiation of white, but not brown, adipose tissue. Proc. Natl. Acad. Sci. USA.

[35] Porse B.T., Pedersen T.A., Xu X.F., Lindberg B., Wewer U.M., Friis-Hansen L., Nerlov C. (2001). E2F repression by C/EBP alpha is required for adipogenesis and granulopoiesis in vivo. Cell.

[36] Long Y., Tsai W.B., Chang J.T., Estecio M., Wangpaichitr M., Savaraj N., Feun L.G., Chen H.H., Kuo M.T. (2016). Cisplatin-induced synthetic lethality to arginine-starvation therapy by transcriptional suppression of ASS1 is regulated by DEC1, HIF-1alpha, and c-Myc transcription network and is independent of ASS1 promoter DNA methylation. Oncotarget.

[37] Morandi E.M., Verstappen R., Zwierzina M.E., Geley S., Pierer G., Ploner C. (2016). ITGAV and ITGA5 diversely regulate proliferation and adipogenic differentiation of human adipose derived stem cells. Sci. Rep..

[38] Shinoda K., Tomita M., Ishihama Y. (2010). emPAI Calc-for the estimation of protein abundance from large-scale identification data by liquid chromatography-tandem mass spectrometry. Bioinformatics.

[39] Ishihama Y., Oda Y., Tabata T., Sato T., Nagasu T., Rappsilber J., Mann M. (2005). Exponentially modified protein abundance index (emPAI) for estimation of absolute protein amount in proteomics by the number of sequenced peptides per protein. Mol. Cell Proteom..

[40] Zhu W.H., Smith J.W., Huang C.M. (2010). Mass Spectrometry-Based Label-Free Quantitative Proteomics. J. Biomed. Biotechnol..

[41] Rappsilber J., Ryder U., Lamond A.I., Mann M. (2002). Large-scale proteomic analysis of the human spliceosome. Genome Res..

[42] Ma H., Su L., Yue H., Yin X., Zhao J., Zhang S., Kung H., Xu Z., Miao J. (2015). HMBOX1 interacts with MT2A to regulate autophagy and apoptosis in vascular endothelial cells. Sci. Rep..

[43] Drzewinska J., Pulaski L., Soszynski M., Bartosz G. (2009). Seladin-1/DHCR24: A key protein of cell homeostasis and cholesterol biosynthesis. Postepy Hig. Med. Dosw. (Online).

[44] Ross D., Siegel D. (2017). Functions of NQO1 in Cellular Protection and CoQ10 Metabolism and its Potential Role as a Redox Sensitive Molecular Switch. Front. Physiol..

[45] Matassa D.S., Agliarulo I., Avolio R., Landriscina M., Esposito F. (2018). TRAP1 Regulation of Cancer Metabolism: Dual Role as Oncogene or Tumor Suppressor. Genes (Basel).

[46] Yan Y., Tsukamoto O., Nakano A., Kato H., Kioka H., Ito N., Higo S., Yamazaki S., Shintani Y., Matsuoka K. (2015). Augmented AMPK activity inhibits cell migration by phosphorylating the novel substrate Pdlim5. Nat. Commun..

[47] Krcmery J., Gupta R., Sadleir R.W., Ahrens M.J., Misener S., Kamide C., Fitchev P., Losordo D.W., Crawford S.E., Simon H.G. (2013). Loss of the cytoskeletal protein Pdlim7 predisposes mice to heart defects and hemostatic dysfunction. PLoS ONE.

[48] Kremer J.C., Van Tine B.A. (2017). Therapeutic arginine starvation in ASS1-deficient cancers inhibits the Warburg effect. Mol. Cell Oncol..

[49] Ren W., Lian P., Cheng L., Du P., Guan X., Wang H., Ding L., Gao Z., Huang X., Xiao F. (2015). FHL1 inhibits the growth of tongue squamous cell carcinoma cells via G1/S cell cycle arrest. Mol. Med. Rep..

[50] Gupta S., Purcell N.H., Lin A., Sen S. (2002). Activation of nuclear factor-kappaB is necessary for myotrophin-induced cardiac hypertrophy. J. Cell Biol..

[51] Chang J., Liu F., Lee M., Wu B., Ting K., Zara J.N., Soo C., Al Hezaimi K., Zou W., Chen X. (2013). NF-kappaB inhibits osteogenic differentiation of mesenchymal stem cells by promoting beta-catenin degradation. Proc. Natl. Acad. Sci. USA.

[52] Guttridge D.C., Albanese C., Reuther J.Y., Pestell R.G., Baldwin A.S. (1999). NF-kappa B controls cell growth and differentiation through transcriptional regulation of cyclin D1. Mol. Cell Biol..

[53] Joyce D., Albanese C., Steer J., Fu M., Bouzahzah B., Pestell R.G. (2001). NF-kappaB and cell-cycle regulation: The cyclin connection. Cytokine Growth Factor Rev..

[54] Nakashima Y., Miyagi-Shiohira C., Kobayashi N., Saitoh I., Watanabe M., Noguchi H. (2017). A proteome analysis of pig pancreatic islets and exocrine tissue by liquid chromatography with tandem mass spectrometry. Islets.

[55] Nesvizhskii A.I., Keller A., Kolker E., Aebersold R. (2003). A statistical model for identifying proteins by tandem mass spectrometry. Anal. Chem..

