# Supplementary material for: A Liquid Chromatography with Tandem Mass Spectrometry-Based Proteomic Analysis of Cells Cultured in DMEM 10% FBS and Chemically Defined Medium Using Human Adipose-Derived Mesenchymal Stem Cells
Source: Int J Mol Sci. 2018 Jul 13;19(7):2042. doi: 10.3390/ijms19072042 (PMC6073410; doi:10.3390/ijms19072042)
Supplement: Supplementary file 1 [file ijms-19-02042-s001.pdf]

Supplementary Materials

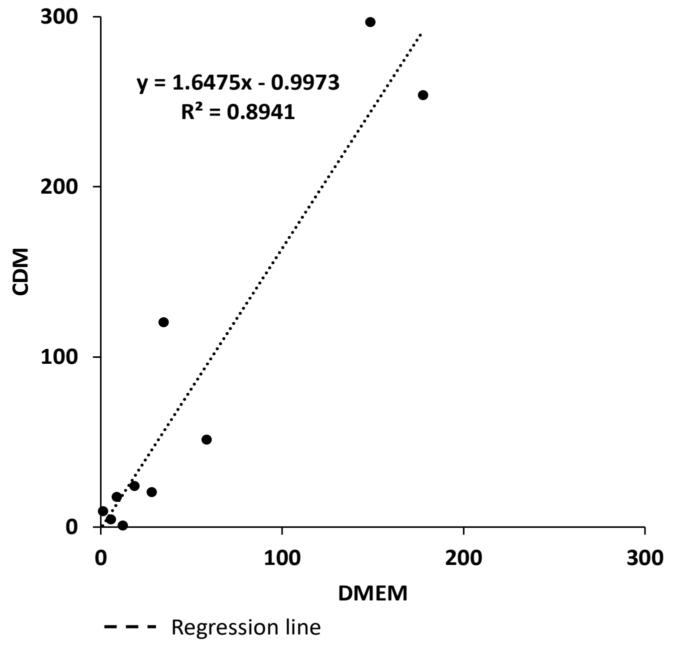

Figure S1. A scatter plot of the housekeeping genes' quantitative values. A scatter plot showing the correlation ($R^2 = 0.8941$) between the quantitative value of the CDM hADSCs and DMEM hADSCs: TFRC, YWHAZ, RPLP0, RPLP1, RPLP2, B2M, RPS18, PGK1, PPIA, and GAPDH ($n = 10$). The dotted line is the regression line. Each dot shows the abbreviated name of the protein. $n = 1$.

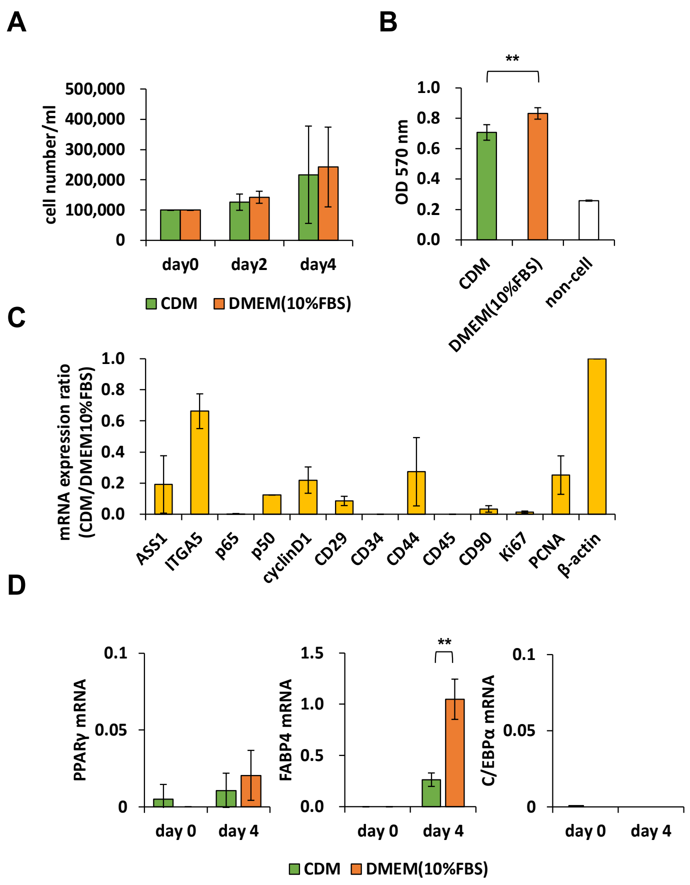

Figure S2. The evaluation of the cell growth ability, gene expression characteristics, and early adipocyte differentiation potential of hADSCs cultured in CDM or DMEM. Cell proliferation assays. hADSCs were counted after 2 and 4 days of culture in the CDM or DMEM (10% FBS). $n = 4$. Data are represented as the mean ± S.D (**A**). MTT cell count assays. hADSCs were analyzed after 4 days of culture in CDM or DMEM (10% FBS). Each measurement was obtained using a microplate reader. $n = 4$. Data are represented as the mean ± S.D. ** $p < 0.01$ (**B**). The expression rate of various gene mRNAs expressed in ADSCs cultured in CDM was compared with that of ADSCs cultured in DMEM (10% FBS). cDNA was synthesized using ADSCs that had been cultured in both CDM and DMEM (10% FBS) for 48 h. The expression was calculated using the ΔΔCt method. The expression of the target gene was corrected by the expression of the housekeeping gene (β-actin). Real time PCR was performed to detect factors related to the cell cycle, cell surface markers, and cell proliferation. The expression of various factors expressed by hADSCs cultured in DMEM (10% FBS) medium was defined as 1. The vertical axis shows the expression ratio of various factors expressed by hADSCs cultured in CDM. $n = 4$. Data are represented as the mean ± S.D. (**C**). The expression of adipocyte differentiation marker gene mRNA on days 0 and 4 was shown by inducing the differentiation of ADSCs cultured in CDM or DMEM (10% FBS) into adipocytes. The expression was calculated using the ΔΔCt method. When determining the expression of the target gene, the expression of the housekeeping gene (β-actin) was taken as 1. $n = 4$. Data are represented as the mean ± S.D. ** $p < 0.01$ (**D**).

Table S1. Identification of endogenous proteins contained in hADSC cultured in medium, Chemically Defined.

Table S1. Identification of endogenous proteins contained in hADSC cultured in medium, Chemically Defined.

UniProt/SWISS-PROT ID	Description	Protein score[a]	Protein mass (kDa)	pI[b]	Num. of matches[c]	Num. of significant matches[d]	Num. of sequences[e]	Num. of significant sequences[f]	Num. of unique sequences[g]	Sequence coverage[h]	emPAI[i]
ACTBM_HUMAN	Putative beta-actin-like protein 3	914	41989	5.91	240	84	5	5	1	0.18	0.64
ALBU_HUMAN	Serum albumin	9054	69321	5.92	1087	544	44	35	44	0.76	10.79
VIME_HUMAN	Vimentin	8790	53619	5.06	1038	421	48	39	48	0.79	21.41
MYH9_HUMAN	Myosin-9	7160	226392	5.5	438	231	84	54	75	0.54	1.98
ENOA_HUMAN	Alpha-enolase	5608	47139	7.01	410	221	20	16	12	0.67	4.35
G3P_HUMAN	Glyceraldehyde-3-phosphate dehydrogenase	5249	36030	8.57	489	202	15	14	8	0.71	11.59
KPYM_HUMAN	Pyruvate kinase PKM	4800	57900	7.96	385	231	33	29	7	0.7	8.31
TBB2A_HUMAN	Tubulin beta-2A chain	3170	49875	4.78	331	188	23	17	3	0.75	4.78
TBB6_HUMAN	Tubulin beta-6 chain	2182	49825	4.77	233	151	20	16	10	0.7	4.32
S10A6_HUMAN	Protein S100-A6	4230	10173	5.33	148	104	7	4	7	0.39	6.25
FLNA_HUMAN	Filamin-A	3559	280564	5.7	359	190	79	47	75	0.52	1.11
FLNB_HUMAN	Filamin-B	796	277990	5.47	60	29	30	17	25	0.25	0.29
FLNC_HUMAN	Filamin-C	478	290841	5.65	41	19	25	13	22	0.18	0.21
HS90A_HUMAN	Heat shock protein HSP 90-alpha	3130	84607	4.94	343	197	35	25	21	0.65	3.19
ENPL_HUMAN	Endoplasmin	1890	92411	4.76	150	77	35	21	33	0.53	1.58
TRAP1_HUMAN	Heat shock protein 75 kDa, mitochondrial	265	80060	8.3	32	24	5	2	5	0.11	0.11
TLN1_HUMAN	Talin-1	3355	269599	5.77	176	127	64	43	64	0.47	1.01
FAS_HUMAN	Fatty acid synthase	3142	273254	6.01	188	120	63	45	63	0.48	1.12
EF2_HUMAN	Elongation factor 2	3095	95277	6.41	203	152	35	26	35	0.65	2.57
GRP78_HUMAN	78 kDa glucose-regulated protein	3083	72288	5.07	182	131	29	22	25	0.66	2.78
GRP75_HUMAN	Stress-70 protein, mitochondrial	1019	73635	5.87	37	26	16	11	16	0.36	0.87
HS71A_HUMAN	Heat shock 70 kDa protein 1A	267	70009	5.48	25	15	7	6	5	0.22	0.43
LEG1_HUMAN	Galectin-1	3041	14706	5.34	223	114	7	5	7	0.66	4.34
AMPN_HUMAN	Aminopeptidase N	3035	109471	5.31	129	96	30	22	30	0.42	1.6
CO6A3_HUMAN	Collagen alpha-3(VI) chain	2932	343457	6.26	164	122	55	39	55	0.35	0.63
CAP1_HUMAN	Adenylyl cyclase-associated protein 1	2626	51869	8.24	148	92	18	11	4	0.49	1.42
IQGA1_HUMAN	Ras GTPase-activating-like protein IQGAP1	2624	189134	6.08	131	97	43	35	43	0.44	1.27
PDIA1_HUMAN	Protein disulfide-isomerase	2584	57081	4.76	227	96	26	14	26	0.61	1.78
ALDOA_HUMAN	Fructose-bisphosphate aldolase A	2578	39395	8.3	178	87	20	16	20	0.78	6.42
DYHC1_HUMAN	Cytoplasmic dynein 1 heavy chain 1	2533	532072	6.01	153	92	67	43	67	0.29	0.4
TERA_HUMAN	Transitional endoplasmic reticulum ATPase	2460	89266	5.14	111	86	31	24	31	0.55	2.54
UBA1_HUMAN	Ubiquitin-like modifier-activating enzyme 1	2362	117774	5.49	134	98	34	27	16	0.65	2.01
CAN2_HUMAN	Calpain-2 catalytic subunit	2091	79945	4.87	95	76	24	21	23	0.59	2.51
ACTN1_HUMAN	Alpha-actinin-1	2032	102993	5.25	156	96	43	34	29	0.68	3.49
ACTN4_HUMAN	Alpha-actinin-4	1417	104788	5.27	114	70	33	21	19	0.52	1.41
CO6A1_HUMAN	Collagen alpha-1(VI) chain	2024	108462	5.26	108	67	25	17	25	0.45	1.16
SERPH_HUMAN	Serpin H1	1995	46411	8.75	167	114	23	20	23	0.65	6.2
GELS_HUMAN	Gelsolin	1950	85644	5.9	110	77	22	17	15	0.6	1.78
STAT1_HUMAN	Signal transducer and activator of transcription 1-alpha/beta	1909	87280	5.74	75	55	22	17	22	0.39	1.61
CH60_HUMAN	60 kDa heat shock protein, mitochondrial	1873	61016	5.7	103	72	25	20	16	0.6	3.2
1433F_HUMAN	14-3-3 protein eta	175	28201	4.76	30	9	4	3	2	0.15	0.55
ANXA2_HUMAN	Annexin A2	1776	38580	7.57	152	102	19	16	19	0.62	5.23
THIO_HUMAN	Thioredoxin	1685	11730	4.82	61	51	7	4	7	0.74	4.63
LDHA_HUMAN	L-lactate dehydrogenase A chain	1670	36665	8.44	156	82	20	14	15	0.79	4.48
LDHB_HUMAN	L-lactate dehydrogenase B chain	1369	36615	5.71	142	64	13	10	12	0.55	2.48
TKT_HUMAN	Transketolase	1662	67835	7.58	88	67	22	20	22	0.64	2.87
PLEC_HUMAN	Plectin	1634	531466	5.74	126	72	60	34	60	0.22	0.31
FINC_HUMAN	Fibronectin	1631	262460	5.46	131	81	47	33	47	0.36	0.78
ACLY_HUMAN	ATP-citrate synthase	1597	120762	6.95	99	61	29	19	29	0.45	0.93
GSTP1_HUMAN	Glutathione S-transferase P	1561	23341	5.43	75	51	12	10	9	0.86	6.02
COF1_HUMAN	Cofilin-1	1554	18491	8.22	183	99	10	9	7	0.6	6.37
PROF1_HUMAN	Profilin-1	1514	15045	8.44	172	83	10	7	10	0.68	7.75
S10AB_HUMAN	Protein S100-A11	1465	11733	6.56	92	73	5	5	5	0.65	21.41
IMB1_HUMAN	Importin subunit beta-1	1416	97108	4.68	81	57	24	20	24	0.54	1.81
PDIA3_HUMAN	Protein disulfide-isomerase A3	1376	56747	5.98	134	67	21	15	5	0.57	2.24
TSP1_HUMAN	Thrombospondin-1	1301	129300	4.71	131	69	27	19	27	0.32	0.97
ATPB_HUMAN	ATP synthase subunit beta, mitochondrial	1272	56525	5.26	116	62	26	24	6	0.78	5.33
TPIS_HUMAN	Triosephosphate isomerase	1247	30772	5.65	101	63	14	11	12	0.72	4.04
ANXA1_HUMAN	Annexin A1	1240	38690	6.57	85	52	18	14	18	0.61	3.49
TAGL2_HUMAN	Transgelin-2	1233	22377	8.41	91	61	15	11	15	0.74	8.1
ECHA_HUMAN	Trifunctional enzyme subunit alpha, mitochondrial	1215	82947	9.16	54	39	17	14	17	0.45	1.13
PGK1_HUMAN	Phosphoglycerate kinase 1	1192	44586	8.3	67	46	18	11	18	0.6	2.36
PRKDC_HUMAN	DNA-dependent protein kinase catalytic subunit	1180	468788	6.75	78	41	44	23	44	0.21	0.25
MVP_HUMAN	Major vault protein	1145	99266	5.34	61	45	21	17	21	0.5	1.14
PLIN3_HUMAN	Perilipin-3	868	47046	5.3	49	38	16	15	2	0.61	2.77
TCPB_HUMAN	T-complex protein 1 subunit beta	1110	57452	6.01	53	30	15	10	15	0.48	1.07
CO1A1_HUMAN	Collagen alpha-1(I) chain	1106	138857	5.6	99	55	29	20	11	0.39	1
HYOU1_HUMAN	Hypoxia up-regulated protein 1	1077	111266	5.16	47	34	12	9	7	0.26	0.46
MARCS_HUMAN	Myristoylated alanine-rich C-kinase substrate	1071	31536	4.47	103	80	5	4	5	0.3	0.93
DPYL2_HUMAN	Dihydropyrimidinase-related protein 2	1058	62255	5.95	54	35	18	12	16	0.49	1.39
DPYL3_HUMAN	Dihydropyrimidinase-related protein 3	536	61924	6.04	29	18	12	6	10	0.45	0.5
WDR1_HUMAN	WD repeat-containing protein 1	1036	66152	6.17	59	39	18	12	18	0.59	1.27
GDIB_HUMAN	Rab GDP dissociation inhibitor beta	1030	50631	6.11	64	37	14	11	9	0.43	1.47
GDIA_HUMAN	Rab GDP dissociation inhibitor alpha	565	50550	5	45	22	11	6	6	0.37	0.64
FSCN1_HUMAN	Fascin	1025	54496	6.84	55	43	16	13	16	0.52	1.92
RPN2_HUMAN	Dolichyl-diphosphooligosaccharide--protein glycosyltransferase subunit 2	1016	69241	5.44	45	35	15	12	15	0.51	1.06
BGH3_HUMAN	Transforming growth factor-beta-induced protein ig-h3	1000	74634	7.62	52	35	14	7	14	0.45	0.57
ML12B_HUMAN	Myosin regulatory light chain 12B	995	19767	4.71	49	26	11	8	6	0.83	4.3
MYL9_HUMAN	Myosin regulatory light polypeptide 9	350	19814	4.8	31	12	7	4	2	0.48	1.29
CATB_HUMAN	Cathepsin B	977	37797	5.88	68	34	9	8	9	0.42	2
GARS_HUMAN	Glycine--tRNA ligase	972	83113	6.61	59	34	16	11	10	0.4	0.83
TCPZ_HUMAN	T-complex protein 1 subunit zeta	965	57988	6.23	45	34	13	11	13	0.47	1.21
ANXA5_HUMAN	Annexin A5	964	35914	4.94	112	51	18	11	18	0.69	4.04
ATPA_HUMAN	ATP synthase subunit alpha, mitochondrial	947	59714	9.16	60	45	16	11	3	0.44	1.31
PRDX6_HUMAN	Peroxiredoxin-6	928	25019	6	71	40	14	10	14	0.7	4.23
LPPRC_HUMAN	Leucine-rich PPR motif-containing protein, mitochondrial	925	157805	5.81	51	31	22	11	22	0.3	0.34
CATK_HUMAN	Cathepsin K	916	36942	8.72	34	28	7	6	7	0.53	1.75
ARF4_HUMAN	ADP-ribosylation factor 4	575	20498	6.59	39	23	8	5	4	0.66	2.33
RINI_HUMAN	Ribonuclease inhibitor	898	49941	4.71	68	31	19	15	19	0.73	2.49
COPG1_HUMAN	Coatomer subunit gamma-1	885	97655	5.32	51	35	22	15	22	0.45	1.07
PLST_HUMAN	Plastin-3	856	70766	5.41	52	38	19	15	19	0.57	1.42
PGAM1_HUMAN	Phosphoglycerate mutase 1	846	28786	6.67	102	45	12	7	12	0.66	2.17
PABP4_HUMAN	Polyadenylate-binding protein 4	831	70738	9.31	27	17	8	4	8	0.21	0.34
G6PI_HUMAN	Glucose-6-phosphate isomerase	825	63107	8.43	54	37	16	7	16	0.55	0.81
ESTD_HUMAN	S-formylglutathione hydrolase	817	31442	6.54	34	22	12	7	12	0.73	1.52
PSMD2_HUMAN	26S proteasome non-ATPase regulatory subunit 2	809	100136	5.08	42	25	21	16	21	0.45	0.95
GANAB_HUMAN	Neutral alpha-glucosidase AB	794	106807	5.74	70	40	28	17	28	0.57	0.95
NNMT_HUMAN	Nicotinamide N-methyltransferase	792	29555	5.56	41	34	10	9	10	0.66	3.68
CATD_HUMAN	Cathepsin D	792	44524	6.1	64	37	13	9	13	0.54	1.55
CKAP4_HUMAN	Cytoskeleton-associated protein 4	788	65983	5.63	50	26	16	11	16	0.36	1.01

[a]Protein score is calculated from the score of the peptide attributed to the protein.
[b]pI is (Predicted) isoelectric point.
[c]Number of matches is spectrum number matched to protein[#1].
[d]Number of significant matches is spectrum number that matches protein and exceeds the identification criteria.
[e]Number of sequences is number of peptides matched to protein[#2].
[f]Number of significant sequences is number of peptides exceeding the identification criteria matched to proteins.
[g]Number of unique sequences is a unique[#3] number of peptides matched to proteins.
[h]Sequence coverage is the ratio of the total number of matched peptide residues to the total length of the protein.
[i]Exponentially Modified Protein Abundance Index (http://www.matrixscience.com/help/quant_empai_help.html).

Table S1. Cont 1.

UniProt/SWISS-PROT ID	Description	Protein score[a]	Protein mass (kDa)	pI[b]	Num. of matches[c]	Num. of significant matches[d]	Num. of sequences[e]	Num. of significant sequences[f]	Num. of unique sequences[g]	Sequence coverage[h]	emPAI[i]
SAP_HUMAN	Prosaposin	774	58074	5.06	59	36	12	8	12	0.49	1.37
EF1G_HUMAN	Elongation factor 1-gamma	772	50087	6.25	61	39	17	12	17	0.61	1.71
P4HA2_HUMAN	Prolyl 4-hydroxylase subunit alpha-2	770	60864	5.49	40	26	11	10	11	0.36	0.99
LRP1_HUMAN	Prolow-density lipoprotein receptor-related protein 1	763	504276	5.16	76	40	37	21	37	0.19	0.2
TCPE_HUMAN	T-complex protein 1 subunit epsilon	762	59633	5.45	46	30	13	8	13	0.49	0.88
CPNS1_HUMAN	Calpain small subunit 1	755	28298	5.05	36	25	10	7	8	0.78	1.79
MOES_HUMAN	Moesin	741	67778	6.08	68	30	24	11	17	0.53	0.97
IDHC_HUMAN	Isocitrate dehydrogenase [NADP] cytoplasmic	738	46630	6.53	66	33	13	9	12	0.55	1.23
FPPS_HUMAN	Farnesyl pyrophosphate synthase	735	48245	5.83	32	24	9	8	9	0.47	1.17
HNRPK_HUMAN	Heterogeneous nuclear ribonucleoprotein K	731	50944	5.39	35	21	12	9	12	0.42	1.27
VINC_HUMAN	Vinculin	728	123722	5.5	72	30	26	16	26	0.34	0.72
PDC6I_HUMAN	Programmed cell death 6-interacting protein	727	95963	6.13	38	30	12	10	12	0.24	0.61
PPIB_HUMAN	Peptidyl-prolyl cis-trans isomerase B	715	23728	9.42	77	40	12	6	12	0.56	2.38
NDKB_HUMAN	Nucleoside diphosphate kinase B	714	17287	8.52	65	25	7	5	3	0.59	2.27
NDKA_HUMAN	Nucleoside diphosphate kinase A	306	17138	5.83	86	23	9	7	5	0.78	5.83
NEP_HUMAN	Neprilysin	706	85460	5.54	44	26	21	12	21	0.43	0.89
SYIC_HUMAN	Isoleucine--tRNA ligase, cytoplasmic	700	144406	5.82	69	38	27	14	27	0.37	0.5
UGDH_HUMAN	UDP-glucose 6-dehydrogenase	696	54989	6.73	40	27	19	13	19	0.61	1.89
6PGD_HUMAN	6-phosphogluconate dehydrogenase, decarboxylating	692	53106	6.8	41	32	14	12	13	0.43	1.78
PDIA6_HUMAN	Protein disulfide-isomerase A6	692	48091	4.95	50	33	13	12	11	0.56	2.08
GCN1_HUMAN	eIF-2-alpha kinase activator GCN1	689	292572	7.29	40	20	19	8	19	0.15	0.12
OST48_HUMAN	Dolichyl-diphosphooligosaccharide--protein glycosyltransferase 48 kDa subunit	688	50769	6.09	30	18	6	6	6	0.33	0.64
DC1I2_HUMAN	Cytoplasmic dynein 1 intermediate chain 2	687	71412	5.08	30	25	4	4	4	0.2	0.34
2AAA_HUMAN	Serine/threonine-protein phosphatase 2A 65 kDa regulatory subunit A alpha isoform	684	65267	5	40	30	13	10	13	0.36	1.02
ANXA6_HUMAN	Annexin A6	679	75826	5.42	40	25	16	12	16	0.43	0.94
XPO2_HUMAN	Exportin-2	667	110346	5.51	32	20	14	10	14	0.29	0.52
TCPA_HUMAN	T-complex protein 1 subunit alpha	663	60306	5.8	37	19	18	10	18	0.56	1.14
GBB4_HUMAN	Guanine nucleotide-binding protein subunit beta-4	41	37543	5.6	11	3	7	2	4	0.32	0.25
TCPH_HUMAN	T-complex protein 1 subunit eta	643	59329	7.55	43	22	16	11	16	0.48	1.17
SYEP_HUMAN	Bifunctional glutamate/proline--tRNA ligase	643	170483	7.02	60	25	24	11	24	0.31	0.31
SND1_HUMAN	Staphylococcal nuclease domain-containing protein 1	631	101934	6.74	49	37	16	13	16	0.36	0.85
AL9A1_HUMAN	4-trimethylaminobutyraldehyde dehydrogenase	602	53767	5.69	20	12	7	5	3	0.27	0.59
CO1A2_HUMAN	Collagen alpha-2(I) chain	627	129235	9.08	50	25	18	10	16	0.24	0.38
HSP74_HUMAN	Heat shock 70 kDa protein 4	616	94271	5.11	53	29	23	15	23	0.48	0.95
RRBP1_HUMAN	Ribosome-binding protein 1	614	152381	8.69	47	22	15	9	15	0.18	0.28
CLIC1_HUMAN	Chloride intracellular channel protein 1	612	26906	5.09	68	39	12	9	11	0.63	3
IPO5_HUMAN	Importin-5	610	123550	4.83	44	27	23	13	23	0.42	0.61
PYGB_HUMAN	Glycogen phosphorylase, brain form	609	96635	6.4	31	16	13	8	13	0.29	0.48
TRFE_HUMAN	Serotransferrin	608	77014	6.81	42	19	15	8	15	0.32	0.54
RCN3_HUMAN	Reticulocalbin-3	594	37470	4.74	27	21	10	8	10	0.45	1.43
SYWC_HUMAN	Tryptophan--tRNA ligase, cytoplasmic	586	53132	5.83	20	12	8	5	8	0.31	0.48
PDIA4_HUMAN	Protein disulfide-isomerase A4	580	72887	4.96	43	19	16	10	16	0.36	0.88
TCPD_HUMAN	T-complex protein 1 subunit delta	573	57888	7.96	40	24	15	12	15	0.53	1.55
AHNK_HUMAN	Neuroblast differentiation-associated protein AHNAK	572	628699	5.8	84	33	56	30	56	0.17	0.22
MT2_HUMAN	Metallothionein-2	566	6037	8.23	21	20	2	2	1	0.52	2.73
SYSC_HUMAN	Serine--tRNA ligase, cytoplasmic	566	58740	6.05	30	25	8	7	8	0.33	0.64
ECHM_HUMAN	Enoyl-CoA hydratase, mitochondrial	558	31367	8.34	23	17	7	5	7	0.44	1.21
AT1A1_HUMAN	Sodium/potassium-transporting ATPase subunit alpha-1	558	112824	5.33	29	25	10	9	10	0.19	0.4
EIF3K_HUMAN	Eukaryotic translation initiation factor 3 subunit K	557	25043	4.81	14	13	4	3	4	0.45	0.64
ITB1_HUMAN	Integrin beta-1	557	88357	5.27	36	19	10	7	8	0.24	0.39
PSME2_HUMAN	Proteasome activator complex subunit 2	554	27384	5.54	28	16	6	6	6	0.4	1.47
CO6A2_HUMAN	Collagen alpha-2(VI) chain	552	108512	5.85	34	26	14	13	14	0.25	0.72
P4HA1_HUMAN	Prolyl 4-hydroxylase subunit alpha-1	552	61011	5.7	48	25	11	9	11	0.36	0.98
UGGG1_HUMAN	UDP-glucose:glycoprotein glucosyltransferase 1	548	177078	5.42	37	22	19	11	19	0.28	0.3
CAPG_HUMAN	Macrophage-capping protein	537	38474	5.82	48	25	8	7	8	0.45	1.13
MDHM_HUMAN	Malate dehydrogenase, mitochondrial	537	35481	8.92	32	21	11	7	11	0.52	1.27
THIC_HUMAN	Acetyl-CoA acetyltransferase, cytosolic	533	41324	6.47	20	19	7	6	7	0.47	1.02
ARPC2_HUMAN	Actin-related protein 2/3 complex subunit 2	532	34311	6.84	28	23	6	5	6	0.31	1.33
DDX3X_HUMAN	ATP-dependent RNA helicase DDX3X	528	73198	6.73	27	18	6	4	6	0.18	0.26
G6PD_HUMAN	Glucose-6-phosphate 1-dehydrogenase	524	59219	6.39	38	21	12	8	12	0.43	0.76
CALX_HUMAN	Calnexin	522	67526	4.47	55	27	12	5	12	0.4	0.64
APT_HUMAN	Adenine phosphoribosyltransferase	517	19595	5.78	13	11	6	4	6	0.49	1.31
SYAC_HUMAN	Alanine--tRNA ligase, cytoplasmic	516	106743	5.34	37	26	17	12	17	0.33	0.66
GNAI2_HUMAN	Guanine nucleotide-binding protein G(i) subunit alpha-2	435	40425	5.34	21	14	10	5	6	0.41	0.67
MYOF_HUMAN	Myoferlin	510	234561	5.84	61	29	28	15	28	0.26	0.33
PSMD1_HUMAN	26S proteasome non-ATPase regulatory subunit 1	505	105769	5.25	31	24	16	12	16	0.4	0.67
SPTN1_HUMAN	Spectrin alpha chain, non-erythrocytic 1	496	284364	5.22	37	18	19	9	19	0.16	0.14
PARK7_HUMAN	Protein/nucleic acid deglycase DJ-1	496	19878	6.33	43	26	8	6	8	0.72	3.27
ZYX_HUMAN	Zyxin	494	61238	6.22	36	14	8	4	8	0.24	0.31
CAN1_HUMAN	Calpain-1 catalytic subunit	490	81838	5.49	30	17	13	9	13	0.41	0.58
HNRPU_HUMAN	Heterogeneous nuclear ribonucleoprotein U	488	90528	5.76	22	20	9	8	9	0.23	0.51
XRCC5_HUMAN	X-ray repair cross-complementing protein 5	483	82652	5.55	51	24	15	11	15	0.49	0.74
CNN3_HUMAN	Calponin-3	479	36391	5.69	39	19	11	8	10	0.54	1.79
EF1D_HUMAN	Elongation factor 1-delta	476	31103	4.9	29	18	8	6	7	0.4	1.23
HSPB1_HUMAN	Heat shock protein beta-1	476	22768	5.98	55	32	9	7	9	0.71	2.56
CNDP2_HUMAN	Cytosolic non-specific dipeptidase	471	52845	5.66	18	14	6	4	6	0.29	0.37
SC23A_HUMAN	Protein transport protein Sec23A	469	86105	6.64	28	15	14	7	14	0.41	0.4
CALU_HUMAN	Calumenin	389	37084	4.47	45	18	14	10	4	0.6	2.07
DHE3_HUMAN	Glutamate dehydrogenase 1, mitochondrial	461	61359	7.66	30	15	7	5	7	0.23	0.5
TPM4_HUMAN	Tropomyosin alpha-4 chain	460	28504	4.67	38	20	10	4	6	0.34	0.79
SYVC_HUMAN	Valine--tRNA ligase	459	140387	7.53	30	20	12	8	12	0.21	0.27
LAMB1_HUMAN	Laminin subunit beta-1	454	197909	4.83	33	25	11	8	11	0.14	0.21
EF1B_HUMAN	Elongation factor 1-beta	453	24748	4.5	28	18	9	7	4	0.55	4.31
NIBL1_HUMAN	Niban-like protein 1	451	84085	5.82	38	16	17	8	17	0.33	0.49
AL1L2_HUMAN	Mitochondrial 10-formyltetrahydrofolate dehydrogenase	449	101681	6.13	19	13	9	5	9	0.21	0.23
PSB7_HUMAN	Proteasome subunit beta type-7	440	29946	7.57	10	9	2	2	2	0.17	0.32
ARC1B_HUMAN	Actin-related protein 2/3 complex subunit 1B	439	40923	8.69	20	15	8	5	7	0.39	0.66
NUCL_HUMAN	Nucleolin	437	76568	4.6	16	12	4	4	4	0.11	0.24
AP1B1_HUMAN	AP-1 complex subunit beta-1	362	104570	4.94	24	17	10	8	6	0.2	0.38
PCBP2_HUMAN	Poly(rC)-binding protein 2	249	38556	6.33	42	19	9	5	1	0.43	0.72
ERD23_HUMAN	ER lumen protein-retaining receptor 3	166	25010	9.07	10	7	3	3	2	0.22	0.64
RL9_HUMAN	60S ribosomal protein L9	431	21850	9.96	21	15	5	4	5	0.53	1.57
CLIC4_HUMAN	Chloride intracellular channel protein 4	429	28754	5.45	39	17	12	7	11	0.64	1.74
TCPG_HUMAN	T-complex protein 1 subunit gamma	427	60495	6.1	24	14	15	8	15	0.52	0.74
SEPR_HUMAN	Prolyl endopeptidase FAP	425	87657	6.21	29	19	13	9	13	0.35	0.54
PSB4_HUMAN	Proteasome subunit beta type-4	421	29185	5.72	15	8	5	3	5	0.36	0.53
UCHL1_HUMAN	Ubiquitin carboxyl-terminal hydrolase isozyme L1	410	24808	5.33	55	25	9	8	9	0.65	3.5
MAP1B_HUMAN	Microtubule-associated protein 1B	407	270468	4.73	29	16	17	11	17	0.15	0.19
SYHC_HUMAN	Histidine--tRNA ligase, cytoplasmic	406	57374	5.72	15	9	5	3	5	0.2	0.24
GSTO1_HUMAN	Glutathione S-transferase omega-1	406	27548	6.23	41	24	10	6	10	0.56	2.33

[a]Protein score is calculated from the score of the peptide attributed to the protein.
[b]pI is (Predicted) isoelectric point.
[c]Number of matches is spectrum number matched to protein[#1].
[d]Number of significant matches is spectrum number that matches protein and exceeds the identification criteria.
[e]Number of sequences is number of peptides matched to protein[#2].
[f]Number of significant sequences is number of peptides exceeding the identification criteria matched to proteins.
[g]Number of unique sequences is a unique[#3] number of peptides matched to proteins.
[h]Sequence coverage is the ratio of the total number of matched peptide residues to the total length of the protein.
[i]Exponentially Modified Protein Abundance Index (http://www.matrixscience.com/help/quant_empai_help.html).

Table S1. Cont 2.

UniProt/SWISS-PROT ID	Description	Protein score[a]	Protein mass (kDa)	pI[b]	Num. of matches[c]	Num. of significant matches[d]	Num. of sequences[e]	Num. of significant sequences[f]	Num. of unique sequences[g]	Sequence coverage[h]	emPAI[i]
TCPQ_HUMAN	T-complex protein 1 subunit theta	403	59583	5.42	26	13	12	7	12	0.34	0.63
FERM2_HUMAN	Fermitin family homolog 2	400	77811	6.26	20	12	7	5	7	0.23	0.31
ADHX_HUMAN	Alcohol dehydrogenase class-3	399	39698	7.45	22	14	8	7	8	0.51	1.31
PLOD1_HUMAN	Procollagen-lysine,2-oxoglutarate 5-dioxygenase 1	396	83497	6.47	32	16	12	7	12	0.35	0.49
PLOD2_HUMAN	Procollagen-lysine,2-oxoglutarate 5-dioxygenase 2	262	84632	6.24	19	10	7	4	4	0.16	0.22
PLOD3_HUMAN	Procollagen-lysine,2-oxoglutarate 5-dioxygenase 3	231	84731	5.69	19	12	8	8	8	0.2	0.48
UAP1_HUMAN	UDP-N-acetylhexosamine pyrophosphorylase	395	58732	5.92	27	11	10	5	10	0.31	0.43
PRDX1_HUMAN	Peroxiredoxin-1	394	22096	8.27	57	25	13	6	10	0.75	2.07
PRDX4_HUMAN	Peroxiredoxin-4	370	30521	5.86	29	14	12	6	10	0.65	1.26
PRDX2_HUMAN	Peroxiredoxin-2	294	21878	5.66	18	12	5	5	4	0.56	1.57
PFKAP_HUMAN	ATP-dependent 6-phosphofructokinase, platelet type	390	85542	7.5	28	20	14	12	13	0.41	0.8
PFKAL_HUMAN	ATP-dependent 6-phosphofructokinase, liver type	325	84964	7.26	16	11	9	7	8	0.27	0.48
COPA_HUMAN	Coatomer subunit alpha	388	138258	7.7	42	19	24	11	24	0.35	0.4
VDAC1_HUMAN	Voltage-dependent anion-selective channel protein 1	385	30754	8.62	10	9	7	6	7	0.43	1.25
BZW1_HUMAN	Basic leucine zipper and W2 domain-containing protein 1	383	48013	5.75	24	15	11	6	11	0.39	0.68
VAT1_HUMAN	Synaptic vesicle membrane protein VAT-1 homolog	374	41893	5.88	40	18	14	9	14	0.54	1.45
PTBP1_HUMAN	Polypyrimidine tract-binding protein 1	374	57186	9.22	19	12	8	5	8	0.38	0.44
P3H1_HUMAN	Prolyl 3-hydroxylase 1	371	83341	5.05	36	21	13	7	13	0.41	0.57
YBOX3_HUMAN	Y-box-binding protein 3	126	40066	9.77	8	5	5	3	3	0.23	0.37
COR1C_HUMAN	Coronin-1C	370	53215	6.65	26	14	7	6	7	0.26	0.73
RD23B_HUMAN	UV excision repair protein RAD23 homolog B	367	43145	4.79	17	13	7	6	7	0.33	0.78
ALDR_HUMAN	Aldose reductase	364	35830	6.51	23	13	7	3	7	0.5	0.42
PP1R7_HUMAN	Protein phosphatase 1 regulatory subunit 7	364	41539	4.84	15	11	7	4	7	0.27	0.65
MK01_HUMAN	Mitogen-activated protein kinase 1	364	41363	6.5	18	11	8	4	6	0.48	0.49
CALD1_HUMAN	Caldesmon	363	93175	5.62	26	12	8	5	8	0.17	0.25
MYPT1_HUMAN	Protein phosphatase 1 regulatory subunit 12A	357	115211	5.31	10	6	4	1	4	0.06	0.04
C1QBP_HUMAN	Complement component 1 Q subcomponent-binding protein, mitochondrial	357	31343	4.74	22	13	6	5	6	0.46	1.21
CNN2_HUMAN	Calponin-2	357	33675	6.95	34	17	9	7	8	0.54	1.37
IPO7_HUMAN	Importin-7	356	119440	4.7	25	17	11	7	11	0.23	0.23
MYO1C_HUMAN	Unconventional myosin-Ic	349	121606	9.46	26	13	14	7	14	0.23	0.27
GPNMB_HUMAN	Transmembrane glycoprotein NMB	342	63882	6.17	23	13	5	3	5	0.09	0.22
XRCC6_HUMAN	X-ray repair cross-complementing protein 6	342	69799	6.23	39	25	11	7	11	0.23	0.52
FUMH_HUMAN	Fumarate hydratase, mitochondrial	341	54602	8.85	21	12	8	7	8	0.33	0.84
PAPS2_HUMAN	Bifunctional 3'-phosphoadenosine 5'-phosphosulfate synthase 2	338	69457	8.18	20	12	9	6	9	0.34	0.43
CAVN1_HUMAN	Caveolae-associated protein 1	336	43450	5.51	11	10	6	5	6	0.25	0.62
PEBP1_HUMAN	Phosphatidylethanolamine-binding protein 1	335	21044	7.01	31	13	9	5	9	0.74	2.23
SCRB2_HUMAN	Lysosome membrane protein 2	334	54255	5	10	7	5	3	5	0.21	0.26
SC31A_HUMAN	Protein transport protein Sec31A	334	132931	6.43	23	14	13	8	13	0.22	0.29
XPO1_HUMAN	Exportin-1	333	123306	5.71	25	14	12	8	12	0.25	0.31
IF4G1_HUMAN	Eukaryotic translation initiation factor 4 gamma 1	331	175382	5.25	36	16	14	8	14	0.18	0.21
MDHC_HUMAN	Malate dehydrogenase, cytoplasmic	329	36403	6.91	41	18	11	8	11	0.6	1.79
POSTN_HUMAN	Periostin	328	93255	7.27	21	13	12	7	12	0.29	0.43
IF2A_HUMAN	Eukaryotic translation initiation factor 2 subunit 1	328	36089	5.02	12	10	6	5	6	0.38	1
LRC59_HUMAN	Leucine-rich repeat-containing protein 59	326	34909	9.61	11	6	5	2	5	0.22	0.27
TAGL_HUMAN	Transgelin	323	22596	8.87	39	18	10	5	10	0.6	1.49
GLYM_HUMAN	Serine hydroxymethyltransferase, mitochondrial	323	55958	8.76	14	11	6	4	6	0.27	0.56
DREB_HUMAN	Drebrin	323	71385	4.41	15	13	5	4	5	0.19	0.26
SEPT2_HUMAN	Septin-2	323	41461	6.15	20	14	10	7	9	0.51	1.02
RLA0_HUMAN	60S acidic ribosomal protein P0	321	34252	5.71	35	17	8	4	8	0.44	0.83
ERO1A_HUMAN	ERO1-like protein alpha	320	54358	5.48	23	15	9	6	9	0.36	0.71
LA_HUMAN	Lupus La protein	315	46808	6.68	31	13	8	6	8	0.26	0.7
SAR1A_HUMAN	GTP-binding protein SAR1a	312	22353	6.21	13	8	4	3	4	0.47	0.74
ESYT1_HUMAN	Extended synaptotagmin-1	310	122780	5.57	24	14	14	7	14	0.24	0.27
AMPB_HUMAN	Aminopeptidase B	307	72549	5.51	28	11	8	6	8	0.31	0.41
PAK2_HUMAN	Serine/threonine-protein kinase PAK 2	305	58006	5.69	13	10	5	5	3	0.24	0.43
IF4H_HUMAN	Eukaryotic translation initiation factor 4H	305	27368	6.67	20	14	4	2	4	0.38	0.35
CATL1_HUMAN	Cathepsin L1	305	37540	5.31	12	7	4	1	4	0.29	0.12
SDCB1_HUMAN	Syntenin-1	304	32424	7.05	18	12	9	8	9	0.62	1.78
TMEDA_HUMAN	Transmembrane emp24 domain-containing protein 10	303	24960	6.97	14	10	3	3	3	0.22	0.64
MOB1B_HUMAN	MOB kinase activator 1B	303	25075	6.24	8	6	3	1	3	0.31	0.18
NADC_HUMAN	Nicotinate-nucleotide pyrophosphorylase [carboxylating]	303	30826	5.81	18	11	4	4	4	0.45	1.24
GFPT1_HUMAN	Glutamine--fructose-6-phosphate aminotransferase [isomerizing] 1	302	78756	6.66	22	16	14	11	12	0.36	0.79
GFPT2_HUMAN	Glutamine--fructose-6-phosphate aminotransferase [isomerizing] 2	230	76882	7.03	14	11	9	7	7	0.28	0.46
NQO1_HUMAN	NAD(P)H dehydrogenase [quinone] 1	297	30848	8.91	15	12	7	4	7	0.41	0.96
VIGLN_HUMAN	Vigilin	295	141368	6.43	22	10	13	7	2	0.17	0.23
ATPO_HUMAN	ATP synthase subunit O, mitochondrial	295	23263	9.97	6	6	2	2	2	0.18	0.43
AR6P1_HUMAN	ADP-ribosylation factor-like protein 6-interacting protein 1	292	23347	9.38	6	5	1	1	1	0.14	0.19
PRDX5_HUMAN	Peroxiredoxin-5, mitochondrial	288	22073	8.93	23	10	8	6	8	0.57	2.07
ASPH_HUMAN	Aspartyl/asparaginyl beta-hydroxylase	288	85809	4.92	15	10	5	5	5	0.12	0.28
COPB_HUMAN	Coatomer subunit beta	285	107074	5.72	37	18	17	10	17	0.32	0.48
LKHA4_HUMAN	Leukotriene A-4 hydrolase	283	69241	5.8	11	8	6	3	6	0.2	0.27
DECR_HUMAN	2,4-dienoyl-CoA reductase, mitochondrial	282	36045	9.35	10	8	2	2	2	0.15	0.41
NB5R3_HUMAN	NADH-cytochrome b5 reductase 3	281	34213	7.18	16	12	7	6	7	0.5	1.07
KAD2_HUMAN	Adenylate kinase 2, mitochondrial	281	26461	7.67	10	10	4	4	4	0.23	0.87
IKIP_HUMAN	Inhibitor of nuclear factor kappa-B kinase-interacting protein	279	39285	9.21	9	6	4	2	4	0.21	0.24
SH3G1_HUMAN	Endophilin-A2	277	41464	5.31	14	11	4	3	4	0.2	0.49
GSLG1_HUMAN	Golgi apparatus protein 1	276	134464	6.52	29	8	13	5	13	0.18	0.17
SRC8_HUMAN	Src substrate cortactin	275	61549	5.24	13	7	7	4	7	0.24	0.31
FKB10_HUMAN	Peptidyl-prolyl cis-trans isomerase FKBP10	274	64204	5.36	31	9	13	5	13	0.4	0.38
TXND5_HUMAN	Thioredoxin domain-containing protein 5	273	47599	5.63	51	27	10	5	10	0.43	0.55
ECHB_HUMAN	Trifunctional enzyme subunit beta, mitochondrial	273	51262	9.45	26	13	10	4	10	0.49	0.38
PTGR1_HUMAN	Prostaglandin reductase 1	272	35847	8.45	13	11	5	4	5	0.4	0.78
PGRC1_HUMAN	Membrane-associated progesterone receptor component 1	271	21658	4.56	14	12	4	4	4	0.39	1.14
NCPR_HUMAN	NADPH--cytochrome P450 reductase	269	76641	5.38	12	7	6	4	6	0.2	0.24
COPB2_HUMAN	Coatomer subunit beta'	268	102422	5.15	23	14	14	8	14	0.33	0.39
MYO1B_HUMAN	Unconventional myosin-Ib	265	131902	9.43	30	13	17	7	17	0.26	0.25
PARVA_HUMAN	Alpha-parvin	263	42217	5.69	5	5	2	2	2	0.08	0.22
USO1_HUMAN	General vesicular transport factor p115	263	107828	4.85	15	11	6	4	6	0.14	0.21
RL3_HUMAN	60S ribosomal protein L3	263	46080	10.19	21	13	9	5	9	0.34	0.57
CAZA1_HUMAN	F-actin-capping protein subunit alpha-1	263	32902	5.45	20	17	8	7	6	0.48	1.42
TNPO2_HUMAN	Transportin-2	125	101322	4.87	11	8	4	3	3	0.14	0.28
PUR4_HUMAN	Phosphoribosylformylglycinamidine synthase	260	144643	5.5	12	8	7	4	7	0.13	0.12
SQOR_HUMAN	Sulfide:quinone oxidoreductase, mitochondrial	260	49929	9.18	14	12	7	6	7	0.22	0.65
ODO1_HUMAN	2-oxoglutarate dehydrogenase, mitochondrial	257	115861	6.4	12	10	5	3	5	0.1	0.11
UBP14_HUMAN	Ubiquitin carboxyl-terminal hydrolase 14	256	56033	5.2	26	13	8	5	8	0.35	0.56
NP1L4_HUMAN	Nucleosome assembly protein 1-like 4	256	42797	4.6	15	9	7	4	7	0.36	0.47
BZW2_HUMAN	Basic leucine zipper and W2 domain-containing protein 2	256	48132	6.26	11	8	5	2	5	0.19	0.41
PSD13_HUMAN	26S proteasome non-ATPase regulatory subunit 13	254	42918	5.53	14	10	6	5	6	0.29	0.62
NEUL_HUMAN	Neurolysin, mitochondrial	253	80600	6.21	24	8	4	3	4	0.13	0.23
EIF3F_HUMAN	Eukaryotic translation initiation factor 3 subunit F	251	37540	5.24	13	10	4	4	4	0.29	0.74

[a]Protein score is calculated from the score of the peptide attributed to the protein.
[b]pI is (Predicted) isoelectric point.
[c]Number of matches is spectrum number matched to protein[#1].
[d]Number of significant matches is spectrum number that matches protein and exceeds the identification criteria.
[e]Number of sequences is number of peptides matched to protein[#2].
[f]Number of significant sequences is number of peptides exceeding the identification criteria matched to proteins.
[g]Number of unique sequences is a unique[#3] number of peptides matched to proteins.
[h]Sequence coverage is the ratio of the total number of matched peptide residues to the total length of the protein.
[i]Exponentially Modified Protein Abundance Index (http://www.matrixscience.com/help/quant_empai_help.html).

Table S1. Cont 3.

UniProt/SWISS-PROT ID	Description	Protein score[a]	Protein mass (kDa)	pI[b]	Num. of matches[c]	Num. of significant matches[d]	Num. of sequences[e]	Num. of significant sequences[f]	Num. of unique sequences[g]	Sequence coverage[h]	emPAI[i]
PSA_HUMAN	Puromycin-sensitive aminopeptidase	250	103211	5.49	18	10	6	4	6	0.18	0.18
LAMC1_HUMAN	Laminin subunit gamma-1	248	177489	5.01	18	11	14	8	14	0.16	0.24
PSMD6_HUMAN	26S proteasome non-ATPase regulatory subunit 6	247	45502	5.45	10	8	4	2	4	0.17	0.2
PP14B_HUMAN	Protein phosphatase 1 regulatory subunit 14B	246	15901	4.75	19	15	4	3	4	0.59	1.17
LGUL_HUMAN	Lactoylglutathione lyase	246	20764	5.12	13	9	4	3	4	0.37	0.81
PDIA5_HUMAN	Protein disulfide-isomerase A5	244	59556	8.08	10	3	4	1	4	0.22	0.07
RL5_HUMAN	60S ribosomal protein L5	243	34341	9.73	22	9	8	3	8	0.43	0.44
COMT_HUMAN	Catechol O-methyltransferase	243	30018	5.26	17	12	7	5	7	0.41	1
CISY_HUMAN	Citrate synthase, mitochondrial	242	51680	8.45	20	16	7	6	7	0.29	0.76
EIFCL_HUMAN	Eukaryotic translation initiation factor 3 subunit C-like protein	241	105407	5.45	7	4	6	4	6	0.18	0.17
TM9S3_HUMAN	Transmembrane 9 superfamily member 3	241	67843	6.83	7	6	2	1	2	0.06	0.06
PMVK_HUMAN	Phosphomevalonate kinase	240	21981	5.56	7	6	1	1	1	0.18	0.46
PEPD_HUMAN	Xaa-Pro dipeptidase	240	54513	5.64	10	8	5	5	5	0.24	0.47
COPE_HUMAN	Coatomer subunit epsilon	238	34460	4.97	16	10	8	7	8	0.57	1.33
API5_HUMAN	Apoptosis inhibitor 5	237	58968	6.99	8	6	4	2	4	0.15	0.15
PROF2_HUMAN	Profilin-2	237	15036	6.55	8	8	2	2	2	0.28	0.73
RSU1_HUMAN	Ras suppressor protein 1	236	31521	8.57	20	13	5	4	5	0.43	0.93
TCTP_HUMAN	Translationally-controlled tumor protein	236	19583	4.84	12	8	6	5	6	0.44	1.87
COTL1_HUMAN	Coactosin-like protein	233	15935	5.54	14	10	5	4	5	0.55	1.79
TM9S2_HUMAN	Transmembrane 9 superfamily member 2	232	75725	7.23	11	10	3	3	3	0.12	0.32
ATLA3_HUMAN	Atlastin-3	231	60503	5.43	19	12	9	7	9	0.37	0.62
CRTAP_HUMAN	Cartilage-associated protein	230	46532	5.5	12	10	6	5	5	0.29	0.56
SC65_HUMAN	Endoplasmic reticulum protein SC65	96	50349	4.68	4	2	2	2	1	0.1	0.18
RCN1_HUMAN	Reticulocalbin-1	230	38866	4.86	15	9	6	3	6	0.3	0.38
VATB2_HUMAN	V-type proton ATPase subunit B, brain isoform	230	56465	5.57	14	7	10	5	10	0.43	0.45
RL6_HUMAN	60S ribosomal protein L6	230	32708	10.59	12	9	4	2	4	0.19	0.29
LMF2_HUMAN	Lipase maturation factor 2	230	79647	10.1	15	6	5	4	5	0.14	0.23
BIEA_HUMAN	Biliverdin reductase A	230	33407	6.06	14	10	5	4	5	0.36	0.64
APEX1_HUMAN	DNA-(apurinic or apyrimidinic site) lyase	229	35532	8.33	15	11	5	4	5	0.28	0.6
LICH_HUMAN	Lysosomal acid lipase/cholesteryl ester hydrolase	228	45390	6.42	9	7	5	4	5	0.28	0.44
RBGPR_HUMAN	Rab3 GTPase-activating protein non-catalytic subunit	228	155886	5.4	12	7	7	3	7	0.14	0.14
P5CS_HUMAN	Delta-1-pyrroline-5-carboxylate synthase	226	87248	6.66	43	11	10	5	10	0.29	0.27
SCRN1_HUMAN	Secernin-1	226	46353	4.66	18	13	6	5	6	0.29	0.57
HNRPF_HUMAN	Heterogeneous nuclear ribonucleoprotein F	225	45643	5.38	9	4	4	2	4	0.25	0.2
HNRPM_HUMAN	Heterogeneous nuclear ribonucleoprotein M	225	77464	8.84	16	9	6	3	6	0.17	0.24
HEBP1_HUMAN	Heme-binding protein 1	224	21084	5.71	8	7	2	1	2	0.17	0.22
CD109_HUMAN	CD109 antigen	223	161587	5.59	17	11	10	6	10	0.15	0.2
CO3A1_HUMAN	Collagen alpha-1(III) chain	220	138479	6.21	22	17	9	7	6	0.1	0.24
PSDE_HUMAN	26S proteasome non-ATPase regulatory subunit 14	217	34555	6.06	18	10	5	3	5	0.39	0.62
PPGB_HUMAN	Lysosomal protective protein	217	54431	6.16	11	8	7	4	7	0.27	0.36
ADT2_HUMAN	ADP/ATP translocase 2	92	32831	9.71	18	7	7	4	4	0.32	0.66
OLA1_HUMAN	Obg-like ATPase 1	217	44715	7.64	12	8	5	3	5	0.3	0.32
RAB35_HUMAN	Ras-related protein Rab-35	134	23011	8.52	12	6	4	3	3	0.23	0.71
TSPO_HUMAN	Translocator protein	215	18816	9.43	13	8	3	3	3	0.48	0.92
KAP2_HUMAN	cAMP-dependent protein kinase type II-alpha regulatory subunit	212	45490	4.96	5	3	2	1	2	0.08	0.1
AN32B_HUMAN	Acidic leucine-rich nuclear phosphoprotein 32 family member B	211	28770	3.94	6	6	3	3	2	0.27	0.54
AN32A_HUMAN	Acidic leucine-rich nuclear phosphoprotein 32 family member A	87	28568	3.99	5	2	3	2	2	0.2	0.34
SEPT9_HUMAN	Septin-9	211	65361	9.06	23	8	9	4	9	0.31	0.29
NRDC_HUMAN	Nardilysin	211	131488	4.89	13	5	8	2	8	0.14	0.1
EFTU_HUMAN	Elongation factor Tu, mitochondrial	210	49510	7.26	23	9	9	4	9	0.34	0.4
PUR2_HUMAN	Trifunctional purine biosynthetic protein adenosine-3	209	107699	6.26	13	6	9	4	9	0.23	0.17
GDS1_HUMAN	Rap1 GTPase-GDP dissociation stimulator 1	209	66275	5.17	8	4	4	2	4	0.13	0.13
MIF_HUMAN	Macrophage migration inhibitory factor	209	12468	7.74	46	18	3	3	3	0.36	1.67
DPP3_HUMAN	Dipeptidyl peptidase 3	209	82538	5.02	16	13	9	8	9	0.26	0.58
VATA_HUMAN	V-type proton ATPase catalytic subunit A	205	68260	5.35	16	7	8	5	8	0.34	0.36
FAAA_HUMAN	Fumarylacetoacetase	205	46344	6.46	12	8	6	3	6	0.35	0.31
HMCS1_HUMAN	Hydroxymethylglutaryl-CoA synthase, cytoplasmic	205	57257	5.22	24	9	9	6	9	0.38	0.66
FKBP5_HUMAN	Peptidyl-prolyl cis-trans isomerase FKBP5	205	51180	5.7	9	6	5	3	5	0.21	0.28
IMA7_HUMAN	Importin subunit alpha-7	204	59991	4.89	20	10	8	5	7	0.35	0.63
ABHDA_HUMAN	Mycophenolic acid acyl-glucuronide esterase, mitochondrial	203	33911	8.8	7	7	2	2	2	0.12	0.28
PRDX3_HUMAN	Thioredoxin-dependent peroxide reductase, mitochondrial	203	27675	7.67	9	8	4	3	4	0.42	0.57
SCFD1_HUMAN	Sec1 family domain-containing protein 1	203	72334	5.89	12	7	6	3	6	0.22	0.26
SNAA_HUMAN	Alpha-soluble NSF attachment protein	203	33211	5.23	15	11	6	4	6	0.35	0.65
STAT2_HUMAN	Signal transducer and activator of transcription 2	203	97855	5.34	16	7	7	4	7	0.17	0.19
IMA1_HUMAN	Importin subunit alpha-1	202	57826	5.25	5	4	2	1	2	0.09	0.07
SGMR1_HUMAN	Sigma non-opioid intracellular receptor 1	201	25112	5.61	10	6	4	3	4	0.43	0.64
PSMD4_HUMAN	26S proteasome non-ATPase regulatory subunit 4	200	40711	4.68	6	5	4	3	4	0.19	0.36
HPT_HUMAN	Haptoglobin	198	45177	6.13	18	11	6	3	6	0.26	0.32
RWDD1_HUMAN	RWD domain-containing protein 1	198	27922	4.11	9	7	2	2	2	0.23	0.34
SET_HUMAN	Protein SET	198	33469	4.23	16	11	4	3	4	0.27	0.45
NUDC_HUMAN	Nuclear migration protein nudC	198	38219	5.27	10	7	2	2	2	0.1	0.24
AKAP2_HUMAN	A-kinase anchor protein 2	198	94603	5.04	8	6	2	1	2	0.07	0.14
STIP1_HUMAN	Stress-induced-phosphoprotein 1	197	62599	6.4	24	5	8	4	8	0.27	0.31
FKBP9_HUMAN	Peptidyl-prolyl cis-trans isomerase FKBP9	196	63044	4.91	9	6	5	3	5	0.18	0.22
DC1L1_HUMAN	Cytoplasmic dynein 1 light intermediate chain 1	194	56544	6.01	11	7	3	3	3	0.11	0.25
ATP5L_HUMAN	ATP synthase subunit g, mitochondrial	194	11421	9.65	6	3	4	1	4	0.48	0.43
SYK_HUMAN	Lysine--tRNA ligase	194	68005	5.94	17	8	8	4	8	0.3	0.36
PGS2_HUMAN	Decorin	194	39722	8.75	4	3	2	2	2	0.12	0.23
VDAC2_HUMAN	Voltage-dependent anion-selective channel protein 2	193	31547	7.49	10	7	5	2	5	0.36	0.3
LMAN1_HUMAN	Protein ERGIC-53	193	57513	6.3	16	3	8	3	8	0.4	0.24
OTUB1_HUMAN	Ubiquitin thioesterase OTUB1	193	31264	4.85	14	7	8	5	8	0.61	0.94
GLU2B_HUMAN	Glucosidase 2 subunit beta	192	59388	4.33	17	13	5	4	5	0.2	0.42
NASP_HUMAN	Nuclear autoantigenic sperm protein	191	85186	4.26	17	8	6	4	6	0.19	0.22
TWF2_HUMAN	Twinfilin-2	190	39523	6.37	14	10	7	5	7	0.35	0.69
NP1L1_HUMAN	Nucleosome assembly protein 1-like 1	190	45346	4.36	24	14	7	5	7	0.39	0.58
MGST1_HUMAN	Microsomal glutathione S-transferase 1	188	17587	9.41	9	8	3	3	3	0.46	1.02
CTHR1_HUMAN	Collagen triple helix repeat-containing protein 1	188	26207	8.31	7	7	1	1	1	0.12	0.17
NIPS1_HUMAN	Protein NipSnap homolog 1	187	33289	9.35	13	8	4	3	4	0.27	0.45
T126A_HUMAN	Transmembrane protein 126A	186	21513	9.36	5	5	1	1	1	0.11	0.21
DHX9_HUMAN	ATP-dependent RNA helicase A	186	140869	6.41	15	7	7	3	7	0.12	0.09
IPYR_HUMAN	Inorganic pyrophosphatase	185	32639	5.54	17	10	8	6	8	0.54	1.14
PCNA_HUMAN	Proliferating cell nuclear antigen	184	28750	4.57	11	8	7	5	7	0.56	1.06
DYN2_HUMAN	Dynamin-2	183	98003	7.04	5	4	2	1	2	0.07	0.04
CPNE1_HUMAN	Copine-1	183	59022	5.52	16	12	5	4	5	0.24	0.42
HEXB_HUMAN	Beta-hexosaminidase subunit beta	183	63071	6.29	13	7	6	3	6	0.19	0.22
PAI1_HUMAN	Plasminogen activator inhibitor 1	183	45031	6.68	8	5	6	3	6	0.29	0.32
BASP1_HUMAN	Brain acid soluble protein 1	181	22680	4.64	54	15	7	7	7	0.54	2.58
ABRAL_HUMAN	Costars family protein ABRACL	180	9051	5.86	8	5	2	1	2	0.46	0.56
PPIC_HUMAN	Peptidyl-prolyl cis-trans isomerase C	180	22749	8.48	12	10	2	2	2	0.31	1.07
HNRH1_HUMAN	Heterogeneous nuclear ribonucleoprotein H	179	49198	5.89	9	7	4	3	4	0.18	0.29

[a]Protein score is calculated from the score of the peptide attributed to the protein.
[b]pI is (Predicted) isoelectric point.
[c]Number of matches is spectrum number matched to protein[#1].
[d]Number of significant matches is spectrum number that matches protein and exceeds the identification criteria.
[e]Number of sequences is number of peptides matched to protein[#2].
[f]Number of significant sequences is number of peptides exceeding the identification criteria matched to proteins.
[g]Number of unique sequences is a unique[#3] number of peptides matched to proteins.
[h]Sequence coverage is the ratio of the total number of matched peptide residues to the total length of the protein.
[i]Exponentially Modified Protein Abundance Index (http://www.matrixscience.com/help/quant_empai_help.html).

Table S1. Cont 4.

UniProt/SWISS-PROT ID	Description	Protein score[a]	Protein mass (kDa)	pI[b]	Num. of matches[c]	Num. of significant matches[d]	Num. of sequences[e]	Num. of significant sequences[f]	Num. of unique sequences[g]	Sequence coverage[h]	emPAI[i]
TIF1B_HUMAN	Transcription intermediary factor 1-beta	178	88493	5.52	10	7	6	4	6	0.17	0.27
SCOT1_HUMAN	Succinyl-CoA:3-ketoacid coenzyme A transferase 1, mitochondrial	175	56122	7.14	10	8	6	5	6	0.29	0.45
HEXA_HUMAN	Beta-hexosaminidase subunit alpha	175	60664	5.04	9	6	5	4	4	0.21	0.32
GLRX1_HUMAN	Glutaredoxin-1	174	11768	8.33	12	6	2	2	2	0.47	1.82
AATC_HUMAN	Aspartate aminotransferase, cytoplasmic	174	46219	6.52	13	5	4	2	4	0.16	0.2
TFR1_HUMAN	Transferrin receptor protein 1	173	84818	6.18	27	15	8	4	8	0.2	0.22
AL1B1_HUMAN	Aldehyde dehydrogenase X, mitochondrial	173	57170	6.36	20	13	5	3	5	0.19	0.44
AMPL_HUMAN	Cytosol aminopeptidase	172	56131	8.03	17	10	10	6	10	0.36	0.56
PYRG1_HUMAN	CTP synthase 1	172	66648	6.02	8	8	3	3	3	0.13	0.28
MOGS_HUMAN	Mannosyl-oligosaccharide glucosidase	172	91861	8.97	21	11	9	6	9	0.24	0.31
HMOX2_HUMAN	Heme oxygenase 2	171	36010	5.31	5	3	4	2	4	0.25	0.26
ORN_HUMAN	Oligoribonuclease, mitochondrial	171	26816	6.41	6	5	2	1	2	0.2	0.17
DDX58_HUMAN	Probable ATP-dependent RNA helicase DDX58	167	106531	6.03	6	4	4	2	4	0.11	0.08
PHB2_HUMAN	Prohibitin-2	166	33276	9.83	13	8	7	4	7	0.39	0.65
PGM1_HUMAN	Phosphoglucomutase-1	166	61411	6.3	8	6	5	3	5	0.19	0.23
DNJA2_HUMAN	DnaJ homolog subfamily A member 2	165	45717	6.06	11	7	4	2	4	0.24	0.2
ACSL3_HUMAN	Long-chain-fatty-acid--CoA ligase 3	165	80368	8.65	18	9	9	5	9	0.2	0.3
DHC24_HUMAN	Delta(24)-sterol reductase	165	60062	8.42	20	9	9	4	9	0.38	0.32
TMX3_HUMAN	Protein disulfide-isomerase TMX3	165	51839	4.8	6	4	4	2	4	0.21	0.17
ETFA_HUMAN	Electron transfer flavoprotein subunit alpha, mitochondrial	165	35058	8.62	8	6	4	2	4	0.26	0.27
2A5D_HUMAN	Serine/threonine-protein phosphatase 2A 56 kDa regulatory subunit delta isoform	163	69947	8.24	4	3	3	2	3	0.1	0.13
ECH1_HUMAN	Delta(3,5)-Delta(2,4)-dienoyl-CoA isomerase, mitochondrial	163	35793	8.16	13	8	4	2	4	0.19	0.26
RHG01_HUMAN	Rho GTPase-activating protein 1	162	50404	5.85	21	15	8	6	8	0.36	0.64
PCP_HUMAN	Lysosomal Pro-X carboxypeptidase	162	55764	6.75	7	4	2	2	2	0.1	0.16
GDIR1_HUMAN	Rho GDP-dissociation inhibitor 1	161	23193	5.02	25	12	8	5	8	0.51	1.44
CSRP1_HUMAN	Cysteine and glycine-rich protein 1	161	20554	8.9	9	6	6	4	6	0.42	1.23
PUR9_HUMAN	Bifunctional purine biosynthesis protein PURH	160	64575	6.27	17	7	11	4	11	0.38	0.29
COPZ2_HUMAN	Coatomer subunit zeta-2	159	23533	5.08	7	6	3	3	3	0.25	0.69
UBP5_HUMAN	Ubiquitin carboxyl-terminal hydrolase 5	158	95725	4.91	14	8	8	5	8	0.2	0.24
SQSTM_HUMAN	Sequestosome-1	158	47657	5.1	9	6	6	3	6	0.3	0.3
NLTP_HUMAN	Non-specific lipid-transfer protein	157	58956	6.44	5	3	3	1	3	0.07	0.07
CATC_HUMAN	Dipeptidyl peptidase 1	157	51820	6.54	10	8	6	4	6	0.36	0.38
ANM1_HUMAN	Protein arginine N-methyltransferase 1	157	41489	5.24	7	7	3	3	3	0.14	0.35
MP2K2_HUMAN	Dual specificity mitogen-activated protein kinase kinase 2	157	44396	6.12	5	5	2	2	1	0.12	0.21
MP2K1_HUMAN	Dual specificity mitogen-activated protein kinase kinase 1	150	43411	6.18	8	6	4	2	3	0.28	0.21
HCD2_HUMAN	3-hydroxyacyl-CoA dehydrogenase type-2	156	26906	7.66	8	6	5	4	5	0.3	0.85
LAMP1_HUMAN	Lysosome-associated membrane glycoprotein 1	156	44854	9	15	6	4	2	4	0.14	0.2
SNX6_HUMAN	Sorting nexin-6	156	46620	5.81	8	5	3	3	3	0.13	0.31
GT251_HUMAN	Procollagen galactosyltransferase 1	155	71590	6.85	20	8	9	5	9	0.27	0.34
MAOX_HUMAN	NADP-dependent malic enzyme	155	64109	5.79	5	4	4	4	4	0.19	0.3
ERP29_HUMAN	Endoplasmic reticulum resident protein 29	154	28975	6.77	16	6	6	5	6	0.38	1.04
DDX1_HUMAN	ATP-dependent RNA helicase DDX1	154	82380	6.81	13	5	7	4	7	0.18	0.22
ACSL1_HUMAN	Long-chain-fatty-acid--CoA ligase 1	153	77893	6.81	6	4	5	3	5	0.16	0.17
DCTN1_HUMAN	Dynactin subunit 1	152	141607	5.61	18	8	9	3	9	0.14	0.13
DCUP_HUMAN	Uroporphyrinogen decarboxylase	152	40761	5.77	16	7	8	3	8	0.5	0.36
TPP1_HUMAN	Tripeptidyl-peptidase 1	150	61210	6.01	6	4	3	1	3	0.11	0.07
MK03_HUMAN	Mitogen-activated protein kinase 3	149	43108	6.28	11	6	7	4	5	0.39	0.47
IMA3_HUMAN	Importin subunit alpha-3	116	57851	4.8	15	4	8	3	6	0.35	0.24
GNA1_HUMAN	Glucosamine 6-phosphate N-acetyltransferase	149	20736	8.17	8	6	4	3	4	0.42	0.81
GSHB_HUMAN	Glutathione synthetase	148	52352	5.67	8	5	7	5	7	0.25	0.49
ATP5H_HUMAN	ATP synthase subunit d, mitochondrial	148	18480	5.21	8	5	3	2	3	0.34	0.56
CNPY2_HUMAN	Protein canopy homolog 2	148	20639	4.81	8	4	5	4	5	0.43	1.22
NOMO1_HUMAN	Nodal modulator 1	148	134239	5.54	18	7	10	5	10	0.19	0.17
IF4E_HUMAN	Eukaryotic translation initiation factor 4E	147	25082	5.79	12	6	4	3	4	0.33	0.64
DNJB4_HUMAN	DnaJ homolog subfamily B member 4	146	37783	8.65	8	5	6	3	6	0.37	0.39
NONO_HUMAN	Non-POU domain-containing octamer-binding protein	145	54197	9.01	10	3	2	1	2	0.11	0.08
TOM70_HUMAN	Mitochondrial import receptor subunit TOM70	145	67412	6.75	7	5	4	2	4	0.16	0.13
TENA_HUMAN	Tenascin	145	240700	4.79	11	5	7	2	7	0.09	0.04
SAHH_HUMAN	Adenosylhomocysteinase	144	47685	5.92	23	10	7	4	7	0.2	0.42
FUBP2_HUMAN	Far upstream element-binding protein 2	144	73070	6.85	10	6	8	4	8	0.24	0.26
CTND1_HUMAN	Catenin delta-1	144	108103	5.86	5	5	2	2	2	0.06	0.08
PLBL2_HUMAN	Putative phospholipase B-like 2	144	65430	6.34	18	6	4	3	4	0.2	0.29
PUR6_HUMAN	Multifunctional protein ADE2	144	47049	6.95	13	4	9	3	9	0.46	0.3
LIMS1_HUMAN	LIM and senescent cell antigen-like-containing domain protein 1	143	37226	8.43	8	7	5	4	5	0.33	0.75
MPRD_HUMAN	Cation-dependent mannose-6-phosphate receptor	143	30973	5.57	4	4	2	2	2	0.16	0.49
SYYC_HUMAN	Tyrosine--tRNA ligase, cytoplasmic	143	59106	6.61	16	9	5	4	5	0.15	0.33
SEC13_HUMAN	Protein SEC13 homolog	142	35518	5.22	8	7	4	3	4	0.26	0.42
RPE_HUMAN	Ribulose-phosphate 3-epimerase	142	24911	5.33	5	5	1	1	1	0.16	0.39
PSMD3_HUMAN	26S proteasome non-ATPase regulatory subunit 3	141	60939	8.47	15	6	8	4	8	0.25	0.31
DDX6_HUMAN	Probable ATP-dependent RNA helicase DDX6	140	54382	8.85	11	8	5	4	5	0.27	0.36
QORX_HUMAN	Quinone oxidoreductase PIG3	140	35514	6.67	5	3	4	2	4	0.2	0.26
SPB8_HUMAN	Serpin B8	138	42739	5.41	3	3	1	1	1	0.06	0.1
PICAL_HUMAN	Phosphatidylinositol-binding clathrin assembly protein	138	70710	7.7	8	4	4	1	4	0.22	0.13
SCYL1_HUMAN	N-terminal kinase-like protein	138	89575	5.94	4	3	3	2	3	0.11	0.1
EIF3I_HUMAN	Eukaryotic translation initiation factor 3 subunit I	137	36479	5.38	9	6	7	4	7	0.39	0.77
KINH_HUMAN	Kinesin-1 heavy chain	137	109617	6.12	13	5	10	3	10	0.22	0.12
SYMC_HUMAN	Methionine--tRNA ligase, cytoplasmic	136	101052	5.82	9	2	4	1	4	0.11	0.04
SYQ_HUMAN	Glutamine--tRNA ligase	136	87743	6.71	18	7	6	3	6	0.19	0.15
UFL1_HUMAN	E3 UFM1-protein ligase 1	135	89540	6.35	7	4	3	2	3	0.09	0.1
SRPRB_HUMAN	Signal recognition particle receptor subunit beta	135	29684	9.17	3	3	2	2	2	0.13	0.32
RO60_HUMAN	60 kDa SS-A/Ro ribonucleoprotein	135	60631	8.27	9	7	5	3	5	0.19	0.23
EHD4_HUMAN	EH domain-containing protein 4	135	61137	6.33	13	3	5	1	4	0.16	0.07
DHB4_HUMAN	Peroxisomal multifunctional enzyme type 2	135	79636	8.96	14	6	9	4	8	0.28	0.23
PSB1_HUMAN	Proteasome subunit beta type-1	134	26472	8.27	11	5	6	3	6	0.4	0.87
PTTG_HUMAN	Pituitary tumor-transforming gene 1 protein-interacting protein	133	20310	9.14	4	4	1	1	1	0.1	0.22
AP2A1_HUMAN	AP-2 complex subunit alpha-1	133	107478	6.63	33	8	11	6	9	0.23	0.26
AP2A2_HUMAN	AP-2 complex subunit alpha-2	83	103895	6.53	10	4	6	3	4	0.15	0.13
ERP44_HUMAN	Endoplasmic reticulum resident protein 44	132	46941	5.09	7	7	2	2	2	0.11	0.19
C1TC_HUMAN	C-1-tetrahydrofolate synthase, cytoplasmic	132	101495	6.89	27	10	13	7	7	0.28	0.33
SODC_HUMAN	Superoxide dismutase [Cu-Zn]	132	15926	5.7	14	5	4	3	4	0.54	1.17
IMA5_HUMAN	Importin subunit alpha-5	132	60184	4.94	7	5	3	2	2	0.14	0.23
GLRX3_HUMAN	Glutaredoxin-3	132	37408	5.31	16	7	6	3	6	0.3	0.4
SIAS_HUMAN	Sialic acid synthase	130	40281	6.29	17	8	8	4	8	0.49	0.51
THY1_HUMAN	Thy-1 membrane glycoprotein	130	17923	8.96	2	1	2	1	2	0.18	0.26
DCTN3_HUMAN	Dynactin subunit 3	130	21106	5.37	8	4	2	2	2	0.31	0.8
SCMC1_HUMAN	Calcium-binding mitochondrial carrier protein SCaMC-1	130	53320	6	7	5	4	2	4	0.22	0.17
FHL2_HUMAN	Four and a half LIM domains protein 2	129	32171	7.8	6	2	6	2	6	0.37	0.29
MAP4_HUMAN	Microtubule-associated protein 4	129	120930	5.32	25	7	11	3	11	0.18	0.11
TIGAR_HUMAN	Fructose-2,6-bisphosphatase TIGAR	128	30043	7.6	6	5	4	3	4	0.24	0.51
ENPP1_HUMAN	Ectonucleotide pyrophosphatase/phosphodiesterase family member 1	128	104857	6.76	11	7	6	3	6	0.12	0.13

[a]Protein score is calculated from the score of the peptide attributed to the protein.
[b]pI is (Predicted) isoelectric point.
[c]Number of matches is spectrum number matched to protein[#1].
[d]Number of significant matches is spectrum number that matches protein and exceeds the identification criteria.
[e]Number of sequences is number of peptides matched to protein[#2].
[f]Number of significant sequences is number of peptides exceeding the identification criteria matched to proteins.
[g]Number of unique sequences is a unique[#3] number of peptides matched to proteins.
[h]Sequence coverage is the ratio of the total number of matched peptide residues to the total length of the protein.
[i]Exponentially Modified Protein Abundance Index (http://www.matrixscience.com/help/quant_empai_help.html).

Table S1. Cont 5.

UniProt/SWISS-PROT ID	Description	Protein score[a]	Protein mass (kDa)	pI[b]	Num. of matches[c]	Num. of significant matches[d]	Num. of sequences[e]	Num. of significant sequences[f]	Num. of unique sequences[g]	Sequence coverage[h]	emPAI[i]
NAMPT_HUMAN	Nicotinamide phosphoribosyltransferase	128	55487	6.69	8	5	5	4	5	0.22	0.46
AT2A2_HUMAN	Sarcoplasmic/endoplasmic reticulum calcium ATPase 2	128	114683	5.23	15	5	10	5	2	0.18	0.2
GCP60_HUMAN	Golgi resident protein GCP60	126	60556	5.02	6	3	5	3	5	0.2	0.23
CBR1_HUMAN	Carbonyl reductase [NADPH] 1	126	30356	8.55	9	6	4	2	4	0.27	0.51
SPB6_HUMAN	Serpin B6	126	42594	5.18	9	5	4	4	4	0.17	0.48
MRC2_HUMAN	C-type mannose receptor 2	125	166568	5.54	15	8	9	5	9	0.16	0.13
RL22L_HUMAN	60S ribosomal protein L22-like 1	125	14598	9.37	5	5	1	1	1	0.2	0.32
1C12_HUMAN	HLA class I histocompatibility antigen, Cw-12 alpha chain	125	40860	5.91	11	6	5	4	3	0.27	0.5
NACA2_HUMAN	Nascent polypeptide-associated complex subunit alpha-2	101	23209	4.68	7	5	1	1	1	0.07	0.19
SYNC_HUMAN	Asparagine--tRNA ligase, cytoplasmic	123	62903	5.9	12	7	6	3	6	0.22	0.22
ESYT2_HUMAN	Extended synaptotagmin-2	123	102294	9.33	5	3	3	1	3	0.07	0.04
QPCTL_HUMAN	Glutaminyl-peptide cyclotransferase-like protein	122	42897	9.84	10	7	4	3	4	0.33	0.34
VKOR1_HUMAN	Vitamin K epoxide reductase complex subunit 1	122	18223	9.53	7	5	2	2	2	0.19	0.57
IASPP_HUMAN	RelA-associated inhibitor	121	89036	6.37	4	4	1	1	1	0.03	0.05
TOM1_HUMAN	Target of Myb protein 1	120	53785	4.59	8	6	5	3	5	0.28	0.26
NQO2_HUMAN	Ribosyldihydronicotinamide dehydrogenase [quinone]	120	25902	5.88	4	4	1	1	1	0.09	0.17
FLII_HUMAN	Protein flightless-1 homolog	120	144659	5.75	18	6	12	4	12	0.22	0.16
MX1_HUMAN	Interferon-induced GTP-binding protein Mx1	119	75473	5.6	10	6	5	3	5	0.18	0.25
LPXN_HUMAN	Leupaxin	119	43304	5.62	11	7	2	2	2	0.11	0.21
FA98A_HUMAN	Protein FAM98A	119	55366	9.15	6	5	1	1	1	0.05	0.08
CATZ_HUMAN	Cathepsin Z	118	33846	6.7	3	3	2	2	2	0.12	0.28
IMDH2_HUMAN	Inosine-5'-monophosphate dehydrogenase 2	118	55770	6.44	13	6	8	5	8	0.34	0.45
PAPS1_HUMAN	Bifunctional 3'-phosphoadenosine 5'-phosphosulfate synthase 1	118	70788	6.4	6	2	4	2	4	0.16	0.13
HSPB6_HUMAN	Heat shock protein beta-6	118	17125	5.95	6	2	3	1	3	0.39	0.27
MBB1A_HUMAN	Myb-binding protein 1A	117	148762	9.34	8	4	7	3	7	0.09	0.09
KAT3_HUMAN	Kynurenine--oxoglutarate transaminase 3	117	51368	8.4	8	4	3	2	3	0.18	0.18
ACBP_HUMAN	Acyl-CoA-binding protein	117	10038	6.12	11	2	3	2	3	0.51	1.25
NAA15_HUMAN	N-alpha-acetyltransferase 15, NatA auxiliary subunit	117	101208	7.23	10	4	8	3	8	0.16	0.13
GOLP3_HUMAN	Golgi phosphoprotein 3	116	33790	6.05	8	4	4	2	4	0.23	0.28
PML_HUMAN	Protein PML	116	97489	5.88	15	5	8	2	8	0.19	0.09
FABPH_HUMAN	Fatty acid-binding protein, heart	115	14849	6.29	17	6	4	4	3	0.39	2.01
RTRAF_HUMAN	RNA transcription, translation and transport factor protein	114	28051	6.19	6	4	3	3	3	0.21	0.56
EHD2_HUMAN	EH domain-containing protein 2	114	61123	6.03	15	8	7	4	6	0.24	0.31
ASAH1_HUMAN	Acid ceramidase	113	44631	7.52	4	2	4	2	4	0.22	0.21
CY1_HUMAN	Cytochrome c1, heme protein, mitochondrial	113	35399	9.15	10	6	4	3	4	0.2	0.42
TXD17_HUMAN	Thioredoxin domain-containing protein 17	113	13932	5.4	7	5	5	3	5	0.62	1.4
GALK1_HUMAN	Galactokinase	113	42246	6.04	13	7	3	2	3	0.23	0.22
PYR1_HUMAN	CAD protein	113	242829	6.02	24	6	12	5	12	0.13	0.09
RL29_HUMAN	60S ribosomal protein L29	112	17741	11.66	9	5	2	1	2	0.17	0.26
AIFM1_HUMAN	Apoptosis-inducing factor 1, mitochondrial	112	66859	9.04	3	3	3	3	3	0.11	0.21
ANXA7_HUMAN	Annexin A7	110	52706	5.52	9	3	5	2	5	0.2	0.17
CUL4B_HUMAN	Cullin-4B	112	103916	7.01	3	2	3	2	3	0.05	0.08
SC24D_HUMAN	Protein transport protein Sec24D	111	112936	6.91	13	5	10	4	9	0.22	0.16
AIMP2_HUMAN	Aminoacyl tRNA synthase complex-interacting multifunctional protein 2	111	35326	8.45	13	7	7	5	7	0.53	0.8
DNPH1_HUMAN	2'-deoxynucleoside 5'-phosphate N-hydrolase 1	111	19097	4.97	5	3	2	2	2	0.29	0.91
SC24A_HUMAN	Protein transport protein Sec24A	110	119674	7.58	3	3	1	1	1	0.03	0.04
HNRL1_HUMAN	Heterogeneous nuclear ribonucleoprotein U-like protein 1	110	95679	6.49	7	2	4	1	4	0.08	0.04
S10AG_HUMAN	Protein S100-A16	109	11794	6.28	6	4	2	1	2	0.29	0.41
GNS_HUMAN	N-acetylglucosamine-6-sulfatase	109	62042	8.6	16	5	6	3	6	0.19	0.22
ANM5_HUMAN	Protein arginine N-methyltransferase 5	109	72638	5.88	17	11	5	4	5	0.21	0.26
LYPA2_HUMAN	Acyl-protein thioesterase 2	108	24721	6.75	8	5	2	1	2	0.22	0.18
NU160_HUMAN	Nuclear pore complex protein Nup160	108	162017	5.31	9	3	6	2	6	0.1	0.05
CD81_HUMAN	CD81 antigen	108	25792	5.09	6	3	2	2	2	0.18	0.38
ARF6_HUMAN	ADP-ribosylation factor 6	108	20069	9.04	10	7	5	3	5	0.46	0.85
HCDH_HUMAN	Hydroxyacyl-coenzyme A dehydrogenase, mitochondrial	107	34272	8.88	10	5	4	2	4	0.25	0.27
MESD_HUMAN	LRP chaperone MESD	107	26060	7.6	7	5	4	3	4	0.3	0.61
SERC_HUMAN	Phosphoserine aminotransferase	107	40397	7.56	17	4	8	3	8	0.49	0.36
SYAP1_HUMAN	Synapse-associated protein 1	106	39909	4.45	4	4	1	1	1	0.1	0.23
MACF1_HUMAN	Microtubule-actin cross-linking factor 1, isoforms 1/2/3/5	106	837787	5.28	50	4	28	3	28	0.08	0.02
GSTK1_HUMAN	Glutathione S-transferase kappa 1	105	25480	8.5	10	4	5	4	5	0.38	0.91
SYDC_HUMAN	Aspartate--tRNA ligase, cytoplasmic	105	57100	6.11	7	3	6	2	6	0.24	0.16
GBP1_HUMAN	Guanylate-binding protein 1	105	67888	5.97	10	5	6	3	6	0.18	0.2
ACAD9_HUMAN	Acyl-CoA dehydrogenase family member 9, mitochondrial	104	68717	8.16	5	4	3	2	3	0.12	0.2
LTN1_HUMAN	E3 ubiquitin-protein ligase listerin	104	200423	5.89	8	3	6	2	6	0.06	0.04
INF2_HUMAN	Inverted formin-2	104	135540	5.26	13	4	9	4	9	0.17	0.13
AL7A1_HUMAN	Alpha-aminoadipic semialdehyde dehydrogenase	104	58450	8.21	7	6	7	6	7	0.27	0.53
BIN1_HUMAN	Myc box-dependent-interacting protein 1	103	64659	4.97	2	2	1	1	1	0.03	0.07
TBCD4_HUMAN	TBC1 domain family member 4	102	146471	6.57	7	2	6	1	6	0.1	0.03
SPSY_HUMAN	Spermine synthase	101	41242	4.87	10	6	7	4	7	0.43	0.5
IPO4_HUMAN	Importin-4	101	118640	4.88	9	5	6	3	6	0.13	0.11
DOCK7_HUMAN	Dedicator of cytokinesis protein 7	101	242407	6.34	12	6	7	4	7	0.08	0.07
MPPA_HUMAN	Mitochondrial-processing peptidase subunit alpha	101	58216	6.45	8	6	2	2	2	0.08	0.15
S35B2_HUMAN	Adenosine 3'-phospho 5'-phosphosulfate transporter 1	101	47484	9.32	2	1	2	1	2	0.07	0.09
SSBP_HUMAN	Single-stranded DNA-binding protein, mitochondrial	100	17249	9.59	6	2	4	2	4	0.41	0.61
RL13_HUMAN	60S ribosomal protein L13	100	24247	11.65	15	5	4	2	4	0.19	0.41
GRN_HUMAN	Granulins	100	63500	6.43	14	4	5	3	5	0.09	0.22
PEX14_HUMAN	Peroxisomal membrane protein PEX14	100	41212	4.89	3	3	1	1	1	0.1	0.11
PSD12_HUMAN	26S proteasome non-ATPase regulatory subunit 12	100	52871	7.53	11	3	7	2	7	0.32	0.17
USMG5_HUMAN	Up-regulated during skeletal muscle growth protein 5	100	6453	9.78	4	2	1	1	1	0.26	0.85
DTD1_HUMAN	D-aminoacyl-tRNA deacylase 1	98	23409	8.35	11	3	4	2	4	0.36	0.42
OSGEP_HUMAN	Probable tRNA N6-adenosine threonylcarbamoyltransferase	98	36403	5.94	4	2	3	1	3	0.23	0.12
CD63_HUMAN	CD63 antigen	98	25619	8.14	37	13	3	2	3	0.21	0.62
ECM29_HUMAN	Proteasome-associated protein ECM29 homolog	98	204160	6.74	18	4	13	2	13	0.19	0.04
THIL_HUMAN	Acetyl-CoA acetyltransferase, mitochondrial	98	45171	8.98	4	4	2	2	2	0.12	0.2
IVD_HUMAN	Isovaleryl-CoA dehydrogenase, mitochondrial	97	46290	8.45	5	2	3	2	3	0.18	0.2
OAT_HUMAN	Ornithine aminotransferase, mitochondrial	97	48504	6.57	9	5	7	4	7	0.32	0.54
HPRT_HUMAN	Hypoxanthine-guanine phosphoribosyltransferase	96	24564	6.21	3	3	2	2	2	0.28	0.4
MYO9B_HUMAN	Unconventional myosin-Ixb	96	243249	8.91	9	2	7	1	7	0.06	0.02
EIF3A_HUMAN	Eukaryotic translation initiation factor 3 subunit A	95	166468	6.38	8	3	5	1	5	0.06	0.03
APOL2_HUMAN	Apolipoprotein L2	95	37069	6.28	6	4	2	2	2	0.17	0.4
RUVB1_HUMAN	RuvB-like 1	94	50196	6.02	8	1	3	1	3	0.1	0.09
AGM1_HUMAN	Phosphoacetylglucosamine mutase	94	59814	5.84	15	7	5	3	5	0.25	0.23
ACON_HUMAN	Aconitate hydratase, mitochondrial	94	85372	7.36	10	4	4	2	4	0.12	0.1
TADBP_HUMAN	TAR DNA-binding protein 43	94	44711	5.85	6	4	5	4	5	0.29	0.45
ANFY1_HUMAN	Rabankyrin-5	93	128318	5.7	18	9	8	2	8	0.15	0.07
P3H3_HUMAN	Prolyl 3-hydroxylase 3	93	81786	5.93	8	4	6	2	6	0.16	0.17
SELN_HUMAN	Selenoprotein N	93	65773	5.32	2	2	2	2	2	0.09	0.14
IF6_HUMAN	Eukaryotic translation initiation factor 6	93	26582	4.56	12	6	6	4	6	0.58	1.18
ERG28_HUMAN	Probable ergosterol biosynthetic protein 28	92	15854	9.86	2	2	1	1	1	0.09	0.29
SYLC_HUMAN	Leucine--tRNA ligase, cytoplasmic	92	134379	6.95	11	8	6	4	6	0.14	0.13

[a]Protein score is calculated from the score of the peptide attributed to the protein.
[b]pI is (Predicted) isoelectric point.
[c]Number of matches is spectrum number matched to protein[#1].
[d]Number of significant matches is spectrum number that matches protein and exceeds the identification criteria.
[e]Number of sequences is number of peptides matched to protein[#2].
[f]Number of significant sequences is number of peptides exceeding the identification criteria matched to proteins.
[g]Number of unique sequences is a unique[#3] number of peptides matched to proteins.
[h]Sequence coverage is the ratio of the total number of matched peptide residues to the total length of the protein.
[i]Exponentially Modified Protein Abundance Index (http://www.matrixscience.com/help/quant_empai_help.html).

Table S1. Cont 6.

UniProt/SWISS-PROT ID	Description	Protein score[a]	Protein mass (kDa)	pI[b]	Num. of matches[c]	Num. of significant matches[d]	Num. of sequences[e]	Num. of significant sequences[f]	Num. of unique sequences[g]	Sequence coverage[h]	emPAI[i]
ISG15_HUMAN	Ubiquitin-like protein ISG15	92	17876	6.84	4	2	4	2	4	0.36	0.58
UBA6_HUMAN	Ubiquitin-like modifier-activating enzyme 6	92	117895	5.76	12	3	8	1	8	0.17	0.04
IPYR2_HUMAN	Inorganic pyrophosphatase 2, mitochondrial	92	37896	7.07	5	3	4	2	4	0.2	0.25
VP13C_HUMAN	Vacuolar protein sorting-associated protein 13C	92	422124	6.38	12	4	10	2	10	0.07	0.02
TYPH_HUMAN	Thymidine phosphorylase	92	49924	5.36	8	5	3	2	3	0.13	0.18
ANXA4_HUMAN	Annexin A4	91	35860	5.84	6	3	3	2	3	0.16	0.26
PRAF3_HUMAN	PRA1 family protein 3	91	21600	9.77	7	4	2	1	2	0.16	0.21
GBF1_HUMAN	Golgi-specific brefeldin A-resistance guanine nucleotide exchange factor 1	91	206315	5.48	27	7	11	5	10	0.13	0.13
2ABA_HUMAN	Serine/threonine-protein phosphatase 2A 55 kDa regulatory subunit B alpha isoform	91	51659	5.82	5	2	4	2	4	0.16	0.18
COCA1_HUMAN	Collagen alpha-1(XII) chain	90	332941	5.38	11	1	6	1	6	0.03	0.01
PGFRB_HUMAN	Platelet-derived growth factor receptor beta	90	123889	4.88	9	4	3	2	3	0.06	0.07
RETST_HUMAN	All-trans-retinol 13,14-reductase	90	66777	8.54	3	3	1	1	1	0.06	0.13
SYRC_HUMAN	Arginine--tRNA ligase, cytoplasmic	90	75331	6.26	10	5	8	4	8	0.17	0.25
CK5P3_HUMAN	CDK5 regulatory subunit-associated protein 3	90	56885	4.68	6	5	3	3	3	0.09	0.25
ABD12_HUMAN	Monoacylglycerol lipase ABHD12	80	45068	8.87	6	3	4	2	2	0.23	0.2
EIF3H_HUMAN	Eukaryotic translation initiation factor 3 subunit H	89	39905	6.09	19	6	8	3	8	0.35	0.37
AKT2_HUMAN	RAC-beta serine/threonine-protein kinase	89	55733	5.98	4	2	3	1	3	0.11	0.08
ARP10_HUMAN	Actin-related protein 10	89	46277	7.07	4	4	3	3	3	0.21	0.31
PP4R1_HUMAN	Serine/threonine-protein phosphatase 4 regulatory subunit 1	89	106936	4.64	4	2	3	1	3	0.08	0.04
HDGF_HUMAN	Hepatoma-derived growth factor	89	26772	4.7	4	2	3	2	3	0.26	0.36
CYB5_HUMAN	Cytochrome b5	88	15321	4.88	2	2	1	1	1	0.28	0.71
CUTA_HUMAN	Protein CutA	88	19104	5.42	6	6	2	2	2	0.25	0.54
NXP20_HUMAN	Protein NOXP20	88	60704	4.61	7	4	3	2	3	0.15	0.23
GHC1_HUMAN	Mitochondrial glutamate carrier 1	87	34448	9.42	9	2	6	1	6	0.37	0.27
MAOM_HUMAN	NAD-dependent malic enzyme, mitochondrial	87	65402	7.53	4	2	3	1	3	0.1	0.07
QCR2_HUMAN	Cytochrome b-c1 complex subunit 2, mitochondrial	87	48413	8.74	7	2	4	1	4	0.14	0.09
MAGD1_HUMAN	Melanoma-associated antigen D1	87	86107	5.64	5	2	4	1	4	0.12	0.05
RTN4_HUMAN	Reticulon-4	87	129851	4.43	2	2	2	2	2	0.04	0.07
KLC1_HUMAN	Kinesin light chain 1	87	65269	5.87	8	3	5	2	5	0.12	0.14
LRC47_HUMAN	Leucine-rich repeat-containing protein 47	86	63434	8.55	8	4	2	1	2	0.1	0.07
CYFP1_HUMAN	Cytoplasmic FMR1-interacting protein 1	86	145089	6.46	11	5	8	3	8	0.15	0.09
AEBP1_HUMAN	Adipocyte enhancer-binding protein 1	85	130847	5.05	3	1	2	1	2	0.03	0.03
ACADV_HUMAN	Very long-chain specific acyl-CoA dehydrogenase, mitochondrial	84	70345	8.92	4	2	4	2	4	0.15	0.13
ABCD3_HUMAN	ATP-binding cassette sub-family D member 3	84	75428	9.41	5	1	2	1	2	0.04	0.06
GLSK_HUMAN	Glutaminase kidney isoform, mitochondrial	84	73414	7.85	8	5	4	3	4	0.12	0.19
TWF1_HUMAN	Twinfilin-1	83	40258	6.48	9	4	4	4	4	0.28	0.51
CD99_HUMAN	CD99 antigen	83	18836	4.58	3	2	2	1	2	0.39	0.24
SAP3_HUMAN	Ganglioside GM2 activator	83	20825	5.17	13	4	3	2	3	0.5	0.48
TP8L3_HUMAN	Tumor necrosis factor alpha-induced protein 8-like protein 3	83	32639	8.75	2	2	1	1	1	0.07	0.14
SC24C_HUMAN	Protein transport protein Sec24C	82	118249	6.71	6	3	4	1	3	0.09	0.07
NEK6_HUMAN	Serine/threonine-protein kinase Nek6	82	35691	8.26	5	2	2	1	2	0.21	0.12
ATP5J_HUMAN	ATP synthase-coupling factor 6, mitochondrial	82	12580	9.52	4	2	2	1	2	0.32	0.38
FUBP1_HUMAN	Far upstream element-binding protein 1	82	67518	7.18	8	3	7	3	7	0.2	0.2
GSTT2_HUMAN	Glutathione S-transferase theta-2B	82	27490	5.99	3	3	1	1	1	0.1	0.16
GSTM3_HUMAN	Glutathione S-transferase Mu 3	82	26542	5.37	3	3	1	1	1	0.07	0.17
ATOX1_HUMAN	Copper transport protein ATOX1	82	7397	6.71	3	2	2	1	2	0.53	0.71
GLGB_HUMAN	1,4-alpha-glucan-branching enzyme	81	80423	5.87	25	12	9	3	9	0.29	0.17
CDC37_HUMAN	Hsp90 co-chaperone Cdc37	81	44440	5.17	6	5	3	3	3	0.16	0.32
SYTC_HUMAN	Threonine--tRNA ligase, cytoplasmic	81	83382	6.23	13	5	8	4	8	0.18	0.22
NUDT5_HUMAN	ADP-sugar pyrophosphatase	81	24312	4.87	4	3	3	2	3	0.18	0.4
LZIC_HUMAN	Protein LZIC	81	21481	4.88	5	3	1	1	1	0.15	0.21
ICAL_HUMAN	Calpastatin	81	76526	4.98	6	4	2	1	2	0.06	0.12
GALT2_HUMAN	Polypeptide N-acetylgalactosaminyltransferase 2	79	64691	8.63	5	2	5	2	5	0.14	0.14
CDV3_HUMAN	Protein CDV3 homolog	81	27318	6.06	4	2	3	1	3	0.33	0.16
NSDHL_HUMAN	Sterol-4-alpha-carboxylate 3-dehydrogenase, decarboxylating	80	41874	8.16	6	1	5	1	5	0.29	0.1
SF01_HUMAN	Splicing factor 1	80	68286	9.07	6	4	3	1	3	0.1	0.06
ARFG1_HUMAN	ADP-ribosylation factor GTPase-activating protein 1	80	44640	5.46	4	2	4	2	4	0.18	0.21
THIM_HUMAN	3-ketoacyl-CoA thiolase, mitochondrial	80	41898	8.32	5	3	4	3	4	0.28	0.35
QCR1_HUMAN	Cytochrome b-c1 complex subunit 1, mitochondrial	80	52612	5.94	15	2	4	2	4	0.15	0.17
LSM6_HUMAN	U6 snRNA-associated Sm-like protein LSm6	80	9122	9.61	1	1	1	1	1	0.33	0.56
LIMA1_HUMAN	LIM domain and actin-binding protein 1	80	85173	6.41	4	1	3	1	3	0.08	0.05
SAMH1_HUMAN	Deoxynucleoside triphosphate triphosphohydrolase SAMHD1	80	72155	6.69	4	2	4	2	4	0.11	0.12
U2AF2_HUMAN	Splicing factor U2AF 65 kDa subunit	80	53467	9.19	4	3	3	2	3	0.1	0.17
OPTN_HUMAN	Optineurin	80	65880	5.17	1	1	1	1	1	0.03	0.07
ARL3_HUMAN	ADP-ribosylation factor-like protein 3	79	20443	6.74	6	1	3	1	3	0.36	0.22
MMP2_HUMAN	72 kDa type IV collagenase	79	73835	5.26	6	3	3	2	3	0.13	0.18
BID_HUMAN	BH3-interacting domain death agonist	79	21981	5.27	7	4	3	2	3	0.29	0.46
LMAN2_HUMAN	Vesicular integral-membrane protein VIP36	78	40203	6.46	10	5	4	4	4	0.28	0.51
NU133_HUMAN	Nuclear pore complex protein Nup133	78	128898	4.97	5	3	4	2	4	0.06	0.07
PDXK_HUMAN	Pyridoxal kinase	78	35080	5.75	9	3	7	2	7	0.49	0.27
GRHPR_HUMAN	Glyoxylate reductase/hydroxypyruvate reductase	78	35646	7.01	9	6	4	3	4	0.27	0.42
UBE2Z_HUMAN	Ubiquitin-conjugating enzyme E2 Z	78	38186	5.37	2	2	1	1	1	0.06	0.11
AKA12_HUMAN	A-kinase anchor protein 12	78	191367	4.37	18	6	9	4	9	0.13	0.09
TPBG_HUMAN	Trophoblast glycoprotein	77	46003	6.35	7	3	2	2	2	0.09	0.2
G6PE_HUMAN	GDH/6PGL endoplasmic bifunctional protein	77	88836	6.84	15	4	7	3	7	0.18	0.15
DIAP1_HUMAN	Protein diaphanous homolog 1	77	141258	5.31	16	4	10	3	10	0.17	0.09
PCY1A_HUMAN	Choline-phosphate cytidylyltransferase A	77	41705	6.82	6	1	3	1	3	0.13	0.1
FDFT_HUMAN	Squalene synthase	77	48084	6.1	9	2	6	2	6	0.23	0.19
CUL2_HUMAN	Cullin-2	77	86927	6.45	5	3	2	1	2	0.1	0.05
LARP1_HUMAN	La-related protein 1	77	123434	8.91	5	4	3	2	3	0.04	0.07
IF1AX_HUMAN	Eukaryotic translation initiation factor 1A, X-chromosomal	76	16450	5.07	4	3	1	1	1	0.1	0.28
AASS_HUMAN	Alpha-aminoadipic semialdehyde synthase, mitochondrial	76	102066	6.18	6	3	5	2	5	0.11	0.09
PA2G4_HUMAN	Proliferation-associated protein 2G4	76	43759	6.13	5	2	4	2	4	0.21	0.21
EXOC3_HUMAN	Exocyst complex component 3	76	86790	5.8	3	2	2	1	2	0.08	0.1
TRI25_HUMAN	E3 ubiquitin/ISG15 ligase TRIM25	76	70928	8.44	8	4	3	1	3	0.08	0.12
EIF3J_HUMAN	Eukaryotic translation initiation factor 3 subunit J	76	29045	4.72	1	1	1	1	1	0.05	0.15
GMPPB_HUMAN	Mannose-1-phosphate guanyltransferase beta	75	39809	6.16	5	2	4	2	4	0.24	0.23
HEMO_HUMAN	Hemopexin	76	51643	6.55	18	7	7	3	7	0.31	0.38
ABHEB_HUMAN	Protein ABHD14B	75	22332	5.94	3	3	3	3	3	0.3	0.74
SPEE_HUMAN	Spermidine synthase	75	33803	5.3	10	6	6	3	6	0.5	0.44
PLAP_HUMAN	Phospholipase A-2-activating protein	75	87101	5.96	9	3	5	1	5	0.12	0.05
ETFB_HUMAN	Electron transfer flavoprotein subunit beta	75	27826	8.24	1	1	1	1	1	0.07	0.16
COR1B_HUMAN	Coronin-1B	67	54200	5.61	17	3	5	3	5	0.18	0.26
CMBL_HUMAN	Carboxymethylenebutenolidase homolog	75	28030	6.71	6	2	3	1	3	0.21	0.16
GDN_HUMAN	Glia-derived nexin	75	43974	9.35	2	2	1	1	1	0.07	0.1
NCLN_HUMAN	Nicalin	74	62935	6.4	8	5	3	2	3	0.12	0.14
HINT1_HUMAN	Histidine triad nucleotide-binding protein 1	74	13793	6.43	16	4	3	2	3	0.6	0.81
MXRA7_HUMAN	Matrix-remodeling-associated protein 7	74	21452	4.24	2	2	1	1	1	0.14	0.21
FABP5_HUMAN	Fatty acid-binding protein, epidermal	74	15155	6.6	15	3	3	1	3	0.34	0.31
TBCB_HUMAN	Tubulin-folding cofactor B	74	27308	5.06	6	1	3	1	3	0.19	0.16

[a]Protein score is calculated from the score of the peptide attributed to the protein.
[b]pI is (Predicted) isoelectric point.
[c]Number of matches is spectrum number matched to protein[#1].
[d]Number of significant matches is spectrum number that matches protein and exceeds the identification criteria.
[e]Number of sequences is number of peptides matched to protein[#2].
[f]Number of significant sequences is number of peptides exceeding the identification criteria matched to proteins.
[g]Number of unique sequences is a unique[#3] number of peptides matched to proteins.
[h]Sequence coverage is the ratio of the total number of matched peptide residues to the total length of the protein.
[i]Exponentially Modified Protein Abundance Index (http://www.matrixscience.com/help/quant_empai_help.html).

Table S1. Cont 7.

UniProt/SWISS-PROT ID	Description	Protein score[a]	Protein mass (kDa)	pI[b]	Num. of matches[c]	Num. of significant matches[d]	Num. of sequences[e]	Num. of significant sequences[f]	Num. of unique sequences[g]	Sequence coverage[h]	emPAI[i]
CSN8_HUMAN	COP9 signalosome complex subunit 8	74	23211	5.25	7	4	4	2	4	0.49	0.71
KCD12_HUMAN	BTB/POZ domain-containing protein KCTD12	74	35679	5.51	9	4	4	2	4	0.23	0.26
TPD54_HUMAN	Tumor protein D54	73	22224	5.26	7	3	3	2	3	0.33	0.75
CD44_HUMAN	CD44 antigen	73	81487	5.13	2	2	2	2	2	0.04	0.11
RFA2_HUMAN	Replication protein A 32 kDa subunit	72	29228	5.75	6	2	4	1	4	0.31	0.15
TXTP_HUMAN	Tricarboxylate transport protein, mitochondrial	72	33991	9.91	9	3	4	2	4	0.22	0.28
CHM4B_HUMAN	Charged multivesicular body protein 4b	72	24935	4.76	2	2	2	2	2	0.19	0.39
CO5A1_HUMAN	Collagen alpha-1(V) chain	72	183447	4.94	18	2	10	2	10	0.14	0.05
SPTC2_HUMAN	Serine palmitoyltransferase 2	72	62884	7.89	3	1	3	1	3	0.13	0.07
HGS_HUMAN	Hepatocyte growth factor-regulated tyrosine kinase substrate	72	86138	5.84	7	4	3	2	3	0.08	0.1
VASP_HUMAN	Vasodilator-stimulated phosphoprotein	72	39805	9.05	5	2	4	2	4	0.14	0.23
IFM3_HUMAN	Interferon-induced transmembrane protein 3	72	14622	6.49	2	2	2	2	2	0.38	0.75
SYCC_HUMAN	Cysteine--tRNA ligase, cytoplasmic	72	85419	6.31	7	3	4	1	4	0.13	0.05
SPNS1_HUMAN	Protein spinster homolog 1	72	56594	6.19	3	2	2	1	2	0.08	0.08
GUAA_HUMAN	GMP synthase [glutamine-hydrolyzing]	71	76667	6.42	3	2	3	2	3	0.13	0.12
VATL_HUMAN	V-type proton ATPase 16 kDa proteolipid subunit	71	15725	7.98	3	3	1	1	1	0.2	0.3
H2A2A_HUMAN	Histone H2A type 2-A	71	14087	10.9	3	3	1	1	1	0.22	0.34
LMNA_HUMAN	Prelamin-A/C	71	74095	6.57	10	3	6	2	6	0.15	0.12
CKAP5_HUMAN	Cytoskeleton-associated protein 5	71	225352	7.95	6	2	6	2	6	0.05	0.04
ARK72_HUMAN	Aflatoxin B1 aldehyde reductase member 2	71	39564	6.7	5	3	2	2	2	0.14	0.23
ACPH_HUMAN	Acylamino-acid-releasing enzyme	70	81173	5.29	9	3	6	3	6	0.17	0.17
MMS19_HUMAN	MMS19 nucleotide excision repair protein homolog	70	113217	5.92	5	2	4	1	4	0.07	0.04
RPB1B_HUMAN	DNA-directed RNA polymerase II subunit RPB11-b1	70	13080	6.28	5	4	1	1	1	0.26	0.86
TMOD3_HUMAN	Tropomodulin-3	70	39570	5.08	10	6	3	2	3	0.24	0.37
UCRIL_HUMAN	Putative cytochrome b-c1 complex subunit Rieske-like protein 1	70	30796	9.04	1	1	1	1	1	0.07	0.14
SCRB1_HUMAN	Scavenger receptor class B member 1	70	60838	8.55	9	2	5	1	5	0.28	0.07
RPN1_HUMAN	Dolichyl-diphosphooligosaccharide--protein glycosyltransferase subunit 1	70	68527	5.96	27	11	10	4	10	0.29	0.28
MGAT1_HUMAN	Alpha-1,3-mannosyl-glycoprotein 2-beta-N-acetylglucosaminyltransferase	70	50846	9.25	7	3	4	1	4	0.13	0.09
EFHD2_HUMAN	EF-hand domain-containing protein D2	70	26680	5.15	3	2	2	1	2	0.15	0.17
ISOC2_HUMAN	Isochorismatase domain-containing protein 2	69	22323	7.67	4	3	3	2	3	0.35	0.45
CRK_HUMAN	Adapter molecule crk	69	33810	5.38	2	2	2	2	2	0.12	0.28
DHX29_HUMAN	ATP-dependent RNA helicase DHX29	69	155139	8.3	2	2	1	1	1	0.02	0.03
ABI1_HUMAN	Abl interactor 1	69	55047	6.57	5	3	3	1	3	0.14	0.08
PSME1_HUMAN	Proteasome activator complex subunit 1	67	28705	5.78	4	3	3	2	3	0.17	0.33
PMM2_HUMAN	Phosphomannomutase 2	68	28064	6.35	6	2	4	1	4	0.25	0.16
S10A4_HUMAN	Protein S100-A4	68	11721	5.85	7	4	4	1	3	0.58	1
CND1_HUMAN	Condensin complex subunit 1	67	157082	6.19	7	1	6	1	6	0.1	0.03
IF4B_HUMAN	Eukaryotic translation initiation factor 4B	67	69110	5.55	9	4	3	2	3	0.11	0.13
HAP28_HUMAN	28 kDa heat- and acid-stable phosphoprotein	67	20618	8.84	3	2	2	2	2	0.16	0.49
FKB14_HUMAN	Peptidyl-prolyl cis-trans isomerase FKBP14	67	24156	5.69	7	5	3	2	3	0.24	0.41
BAG2_HUMAN	BAG family molecular chaperone regulator 2	67	23757	6.25	12	6	5	1	5	0.47	0.19
IF2P_HUMAN	Eukaryotic translation initiation factor 5B	67	138742	5.39	18	7	11	5	11	0.19	0.16
PGM2_HUMAN	Phosphoglucomutase-2	67	68240	6.28	2	2	2	2	2	0.06	0.13
ATPD_HUMAN	ATP synthase subunit delta, mitochondrial	66	17479	5.38	5	3	3	2	3	0.66	1.03
AK1C3_HUMAN	Aldo-keto reductase family 1 member C3	66	36830	8.06	2	1	2	1	2	0.15	0.12
IRF3_HUMAN	Interferon regulatory factor 3	66	47190	5.17	2	1	2	1	2	0.08	0.09
TTC37_HUMAN	Tetratricopeptide repeat protein 37	65	175375	7.47	9	2	8	2	8	0.11	0.05
LYOX_HUMAN	Protein-lysine 6-oxidase	65	46915	8.36	5	5	1	1	1	0.08	0.19
RIR1_HUMAN	Ribonucleoside-diphosphate reductase large subunit	65	90013	6.76	8	4	6	2	6	0.18	0.1
TRIP4_HUMAN	Activating signal cointegrator 1	65	66105	8.05	1	1	1	1	1	0.06	0.07
SYMPK_HUMAN	Symplekin	64	141059	5.82	4	1	3	1	3	0.07	0.03
OCAD1_HUMAN	OCIA domain-containing protein 1	64	27609	7.02	1	1	1	1	1	0.05	0.16
TM214_HUMAN	Transmembrane protein 214	64	77101	9.28	7	4	4	3	4	0.14	0.18
CPNE3_HUMAN	Copine-3	64	60092	5.6	6	5	4	3	4	0.19	0.23
NEST_HUMAN	Nestin	64	177332	4.35	4	1	3	1	3	0.03	0.02
DCXR_HUMAN	L-xylulose reductase	64	25897	8.33	4	3	2	2	2	0.19	0.38
MYADM_HUMAN	Myeloid-associated differentiation marker	64	35250	8.53	2	1	1	1	1	0.07	0.12
CYGB_HUMAN	Cytoglobin	63	21391	6.32	4	2	2	1	2	0.24	0.21
NIT1_HUMAN	Deaminated glutathione amidase	63	35873	7.91	5	3	3	1	3	0.31	0.12
FOCAD_HUMAN	Focadhesin	63	199944	6.17	12	4	8	3	8	0.1	0.06
SNX8_HUMAN	Sorting nexin-8	62	52536	6.96	8	2	6	1	6	0.16	0.08
RAPH1_HUMAN	Ras-associated and pleckstrin homology domains-containing protein 1	62	135171	8.97	10	1	3	1	3	0.04	0.03
SERC1_HUMAN	Serine incorporator 1	62	50461	5.59	2	1	2	1	2	0.1	0.09
CPPED_HUMAN	Serine/threonine-protein phosphatase CPPED1	62	35526	5.78	6	3	4	3	4	0.27	0.42
CHM2A_HUMAN	Charged multivesicular body protein 2a	62	25088	5.88	6	1	3	1	3	0.17	0.18
MUTA_HUMAN	Methylmalonyl-CoA mutase, mitochondrial	61	83082	6.48	4	2	3	1	3	0.09	0.05
MPV17_HUMAN	Protein Mpv17	61	19720	9.54	6	4	2	1	2	0.36	0.52
GNA14_HUMAN	Guanine nucleotide-binding protein subunit alpha-14	61	41544	5.81	4	3	3	2	2	0.11	0.22
GCDH_HUMAN	Glutaryl-CoA dehydrogenase, mitochondrial	60	48096	8.31	3	2	1	1	1	0.04	0.09
ACOD_HUMAN	Acyl-CoA desaturase	60	41496	9.07	6	4	3	2	3	0.09	0.22
DP13B_HUMAN	DCC-interacting protein 13-beta	60	74446	4.87	6	2	5	1	5	0.2	0.06
E2AK2_HUMAN	Interferon-induced, double-stranded RNA-activated protein kinase	60	62056	8.58	5	2	4	2	4	0.13	0.14
GPAA1_HUMAN	Glycosylphosphatidylinositol anchor attachment 1 protein	60	67580	8.16	3	2	1	1	1	0.04	0.06
PIMT_HUMAN	Protein-L-isoaspartate(D-aspartate) O-methyltransferase	60	24621	6.7	6	4	4	4	2	0.45	0.96
CX6A1_HUMAN	Cytochrome c oxidase subunit 6A1, mitochondrial	59	12147	9.3	3	2	2	1	2	0.46	0.4
MVD1_HUMAN	Diphosphomevalonate decarboxylase	59	43377	6.79	9	4	5	2	5	0.39	0.21
PCOC1_HUMAN	Procollagen C-endopeptidase enhancer 1	59	47942	7.41	13	4	5	3	5	0.29	0.3
RBM3_HUMAN	RNA-binding protein 3	59	17160	8.86	6	3	3	2	3	0.33	0.61
MFGM_HUMAN	Lactadherin	59	43095	8.47	9	4	3	2	3	0.14	0.21
ARPC5_HUMAN	Actin-related protein 2/3 complex subunit 5	58	16310	5.47	2	1	2	1	2	0.35	0.29
SGMR2_HUMAN	Sigma intracellular receptor 2	58	20834	9.42	6	3	2	1	2	0.29	0.22
NUCB1_HUMAN	Nucleobindin-1	58	53846	5.15	11	3	5	2	5	0.26	0.17
RU2A_HUMAN	U2 small nuclear ribonucleoprotein A'	58	28398	8.72	5	1	3	1	3	0.28	0.16
BOLA2_HUMAN	BolA-like protein 2	58	10110	6.07	2	1	2	1	2	0.31	0.49
IAH1_HUMAN	Isoamyl acetate-hydrolyzing esterase 1 homolog	58	27581	5.13	4	3	2	2	2	0.16	0.35
EWS_HUMAN	RNA-binding protein EWS	58	68436	9.37	1	1	1	1	1	0.03	0.06
GLCM_HUMAN	Glucosylceramidase	57	59678	7.29	12	4	4	3	4	0.19	0.23
TIM44_HUMAN	Mitochondrial import inner membrane translocase subunit TIM44	57	51323	8.51	2	1	2	1	2	0.09	0.08
PSMD5_HUMAN	26S proteasome non-ATPase regulatory subunit 5	57	56160	5.35	11	2	4	2	4	0.17	0.16
DAZP1_HUMAN	DAZ-associated protein 1	57	43356	8.73	1	1	1	1	1	0.04	0.1
ATPG_HUMAN	ATP synthase subunit gamma, mitochondrial	57	32975	9.23	4	2	3	2	3	0.22	0.29
ODO2_HUMAN	Dihydrolipoyllysine-residue succinyltransferase component of 2-oxoglutarate dehydrogenase complex, mitochondrial	57	48724	9.11	7	3	5	2	5	0.23	0.19
BROX_HUMAN	BRO1 domain-containing protein BROX	57	46447	7.55	2	1	2	1	2	0.11	0.09
LAT1_HUMAN	Large neutral amino acids transporter small subunit 1	57	54974	7.9	5	4	2	1	2	0.1	0.16
ATP5I_HUMAN	ATP synthase subunit e, mitochondrial	57	7928	9.34	1	1	1	1	1	0.2	0.66
TMM33_HUMAN	Transmembrane protein 33	56	27960	9.75	4	3	2	1	2	0.23	0.16
ABCB6_HUMAN	ATP-binding cassette sub-family B member 6, mitochondrial	56	93826	8.75	3	3	1	1	1	0.03	0.05
UBR4_HUMAN	E3 ubiquitin-protein ligase UBR4	56	573476	5.7	25	4	19	4	19	0.09	0.03
PREP_HUMAN	Presequence protease, mitochondrial	56	117338	6.45	15	4	7	3	7	0.13	0.11
IPO9_HUMAN	Importin-9	56	115889	4.71	8	4	5	3	5	0.15	0.11

[a]Protein score is calculated from the score of the peptide attributed to the protein.
[b]pI is (Predicted) isoelectric point.
[c]Number of matches is spectrum number matched to protein[#1].
[d]Number of significant matches is spectrum number that matches protein and exceeds the identification criteria.
[e]Number of sequences is number of peptides matched to protein[#2].
[f]Number of significant sequences is number of peptides exceeding the identification criteria matched to proteins.
[g]Number of unique sequences is a unique[#3] number of peptides matched to proteins.
[h]Sequence coverage is the ratio of the total number of matched peptide residues to the total length of the protein.
[i]Exponentially Modified Protein Abundance Index (http://www.matrixscience.com/help/quant_empai_help.html).

Table S1. Cont 8.

UniProt/SWISS-PROT ID	Description	Protein score[a]	Protein mass (kDa)	pI[b]	Num. of matches[c]	Num. of significant matches[d]	Num. of sequences[e]	Num. of significant sequences[f]	Num. of unique sequences[g]	Sequence coverage[h]	emPAI[i]
PGRC2_HUMAN	Membrane-associated progesterone receptor component 2	55	23804	4,76	2	2	1	1	1	0,14	0,19
SPRE_HUMAN	Sepiapterin reductase	55	28031	8,24	1	1	1	1	1	0,08	0,16
TTHY_HUMAN	Transthyretin	55	15877	5,52	2	2	1	1	1	0,15	0,29
HS105_HUMAN	Heat shock protein 105 kDa	55	96804	5,28	11	1	8	1	8	0,15	0,04
LASP1_HUMAN	LIM and SH3 domain protein 1	55	29698	6,61	10	3	4	3	4	0,21	0,52
RM12_HUMAN	39S ribosomal protein L12, mitochondrial	55	21335	9,04	3	1	3	1	3	0,31	0,21
STX7_HUMAN	Syntaxin-7	55	29797	5,41	3	2	2	1	2	0,19	0,15
MYDGF_HUMAN	Myeloid-derived growth factor	55	18783	6,2	4	2	2	1	2	0,12	0,25
JOS2_HUMAN	Josephin-2	55	20743	6,9	1	1	1	1	1	0,11	0,22
TS101_HUMAN	Tumor susceptibility gene 101 protein	55	43916	6,06	6	1	4	1	4	0,2	0,1
FACE1_HUMAN	CAAX prenyl protease 1 homolog	54	54778	7,12	3	1	2	1	2	0,07	0,08
QOR_HUMAN	Quinone oxidoreductase	54	35185	8,56	8	4	6	4	6	0,34	0,6
DERL1_HUMAN	Derlin-1	54	28782	9,54	1	1	1	1	1	0,08	0,15
CD59_HUMAN	CD59 glycoprotein	54	14168	6,02	2	1	2	1	2	0,19	0,33
LAMP2_HUMAN	Lysosome-associated membrane glycoprotein 2	54	44932	5,35	1	1	1	1	1	0,04	0,1
RDH14_HUMAN	Retinol dehydrogenase 14	54	36841	9,02	3	1	2	1	2	0,11	0,12
HNRPL_HUMAN	Heterogeneous nuclear ribonucleoprotein L	54	64092	8,46	4	3	3	2	3	0,18	0,14
IF16_HUMAN	Gamma-interferon-inducible protein 16	54	88199	9,31	5	1	3	1	3	0,08	0,05
MMP14_HUMAN	Matrix metalloproteinase-14	54	65852	7,63	4	1	4	1	4	0,08	0,07
PPM1F_HUMAN	Protein phosphatase 1F	54	49800	4,99	3	2	2	2	2	0,13	0,18
TBL2_HUMAN	Transducin beta-like protein 2	53	49766	9,52	1	1	1	1	1	0,03	0,09
CHSP1_HUMAN	Calcium-regulated heat-stable protein 1	53	15882	8,41	2	2	1	1	1	0,24	0,29
DDTL_HUMAN	D-dopachrome decarboxylase-like protein	53	14186	5,89	7	5	3	2	3	0,52	0,78
ARFP1_HUMAN	Arfaptin-1	53	41713	6,24	6	2	2	1	2	0,1	0,22
NU4M_HUMAN	NADH-ubiquinone oxidoreductase chain 4	53	51547	9,4	1	1	1	1	1	0,03	0,08
DOLK_HUMAN	Dolichol kinase	53	59229	8,87	5	2	4	1	4	0,12	0,07
SWP70_HUMAN	Switch-associated protein 70	52	68954	5,66	10	4	5	2	5	0,15	0,13
JUPI1_HUMAN	Jupiter microtubule associated homolog 1	52	16005	5,47	1	1	1	1	1	0,12	0,29
SH3K1_HUMAN	SH3 domain-containing kinase-binding protein 1	52	73082	6,24	5	2	4	2	4	0,09	0,12
PGBM_HUMAN	Basement membrane-specific heparan sulfate proteoglycan core protein	51	468532	6,06	9	1	8	1	8	0,04	0,01
H2A1B_HUMAN	Histone H2A type 1-B/E	51	14127	11,05	1	1	1	1	1	0,22	0,34
MSMO1_HUMAN	Methylsterol monooxygenase 1	51	35193	6,75	5	3	3	2	3	0,18	0,27
TRI47_HUMAN	Tripartite motif-containing protein 47	51	69488	6,03	10	1	2	1	2	0,06	0,06
SC16A_HUMAN	Protein transport protein Sec16A	51	233373	5,4	10	2	7	1	7	0,08	0,02
SOAT1_HUMAN	Sterol O-acyltransferase 1	51	64692	9,08	5	2	4	1	4	0,18	0,07
BDH2_HUMAN	3-hydroxybutyrate dehydrogenase type 2	51	26707	7,56	2	2	1	1	1	0,09	0,17
ADRO_HUMAN	NADPH:adrenodoxin oxidoreductase, mitochondrial	51	53803	8,72	5	2	2	1	2	0,1	0,08
CSN1_HUMAN	COP9 signalosome complex subunit 1	51	55501	6,3	3	1	3	1	3	0,15	0,08
AP3B1_HUMAN	AP-3 complex subunit beta-1	51	121244	5,76	13	5	9	3	9	0,14	0,11
ULA1_HUMAN	NEDD8-activating enzyme E1 regulatory subunit	47	60209	5,25	4	2	4	2	4	0,21	0,15
MIC13_HUMAN	MICOS complex subunit MIC13	50	13079	9,44	2	2	2	2	2	0,44	0,86
TCP4_HUMAN	Activated RNA polymerase II transcriptional coactivator p15	50	14386	9,6	4	1	2	1	2	0,31	0,33
AATM_HUMAN	Aspartate aminotransferase, mitochondrial	50	47487	9,14	4	3	3	3	3	0,1	0,3
VASN_HUMAN	Vasorin	50	71668	7,16	2	2	1	1	1	0,06	0,06
GPX1_HUMAN	Glutathione peroxidase 1	50	22075	6,15	10	4	4	2	4	0,4	0,45
RECQ1_HUMAN	ATP-dependent DNA helicase Q1	50	73410	8,12	3	1	3	1	3	0,1	0,06
HUWE1_HUMAN	E3 ubiquitin-protein ligase HUWE1	50	481589	5,1	14	3	8	2	8	0,05	0,02
HYEP_HUMAN	Epoxide hydrolase 1	50	52915	6,77	4	1	3	1	3	0,15	0,08
PTPA_HUMAN	Serine/threonine-protein phosphatase 2A activator	50	40641	5,63	5	2	2	1	2	0,18	0,23
TBCD_HUMAN	Tubulin-specific chaperone D	50	132515	5,8	7	2	7	2	7	0,11	0,07
ALG1_HUMAN	Chitobiosyldiphosphodolichol beta-mannosyltransferase	49	52484	6,81	2	2	2	2	2	0,1	0,17
NPC2_HUMAN	Epididymal secretory protein E1	49	16559	7,56	3	1	3	1	3	0,33	0,28
PNPH_HUMAN	Purine nucleoside phosphorylase	49	32097	6,45	8	5	5	3	5	0,31	0,47
LSM7_HUMAN	U6 snRNA-associated Sm-like protein LSm7	49	11595	5,1	6	3	2	1	2	0,57	0,42
ECM1_HUMAN	Extracellular matrix protein 1	49	60635	6,25	3	1	3	1	3	0,11	0,07
LGMN_HUMAN	Legumain	49	49379	6,07	1	1	1	1	1	0,07	0,09
TX1B3_HUMAN	Tax1-binding protein 3	48	13726	8,04	3	1	2	1	2	0,38	0,35
PSMD8_HUMAN	26S proteasome non-ATPase regulatory subunit 8	48	39587	9,73	1	1	1	1	1	0,04	0,11
CBX3_HUMAN	Chromobox protein homolog 3	43	20798	5,23	4	2	2	2	2	0,25	0,49
BCAT2_HUMAN	Branched-chain-amino-acid aminotransferase, mitochondrial	48	44259	8,88	4	3	2	1	2	0,15	0,1
FKB1A_HUMAN	Peptidyl-prolyl cis-trans isomerase FKBP1A	48	11943	7,88	18	5	6	2	6	0,78	0,97
4F2_HUMAN	4F2 cell-surface antigen heavy chain	48	67952	4,89	5	2	3	2	3	0,09	0,13
SBDS_HUMAN	Ribosome maturation protein SBDS	48	28745	8,91	4	2	3	1	3	0,22	0,15
YIF1B_HUMAN	Protein YIF1B	48	34413	9,21	3	3	1	1	1	0,08	0,13
CREL2_HUMAN	Cysteine-rich with EGF-like domain protein 2	48	38166	4,5	2	2	1	1	1	0,04	0,11
NICA_HUMAN	Nicastrin	48	78362	5,67	2	1	1	1	1	0,04	0,05
KCY_HUMAN	UMP-CMP kinase	48	22208	5,44	3	2	2	1	2	0,17	0,2
MAP1A_HUMAN	Microtubule-associated protein 1A	47	305298	4,85	14	3	9	3	9	0,06	0,04
EMC1_HUMAN	ER membrane protein complex subunit 1	47	111689	7,38	3	3	2	2	2	0,05	0,08
PYC_HUMAN	Pyruvate carboxylase, mitochondrial	47	129551	6,37	3	1	2	1	2	0,02	0,03
HM13_HUMAN	Minor histocompatibility antigen H13	47	41462	6,01	4	2	3	2	3	0,2	0,22
SSRD_HUMAN	Translocon-associated protein subunit delta	47	18987	5,76	9	4	2	2	2	0,18	0,54
CHMP3_HUMAN	Charged multivesicular body protein 3	47	25057	5,1	1	1	1	1	1	0,14	0,18
DHRS7_HUMAN	Dehydrogenase/reductase SDR family member 7	47	38274	8,59	7	1	3	1	3	0,21	0,11
RT06_HUMAN	28S ribosomal protein S6, mitochondrial	47	14218	9,3	3	2	1	1	1	0,19	0,33
AK1C1_HUMAN	Aldo-keto reductase family 1 member C1	47	36765	8,02	6	3	4	3	4	0,33	0,4
ITAV_HUMAN	Integrin alpha-V	47	115964	5,45	8	2	6	2	6	0,1	0,07
DPP2_HUMAN	Dipeptidyl peptidase 2	47	54307	5,91	4	1	2	1	2	0,09	0,08
APMAP_HUMAN	Adipocyte plasma membrane-associated protein	47	46451	5,82	11	6	3	2	3	0,19	0,2
VAC14_HUMAN	Protein VAC14 homolog	47	87917	5,76	6	1	4	1	4	0,12	0,05
ACOC_HUMAN	Cytoplasmic aconitate hydratase	46	98337	6,23	7	2	3	1	3	0,07	0,04
AP1G1_HUMAN	AP-1 complex subunit gamma-1	46	91293	6,36	5	3	4	2	4	0,11	0,1
PSMG2_HUMAN	Proteasome assembly chaperone 2	46	29377	6,6	3	2	1	1	1	0,14	0,33
IKBB_HUMAN	NF-kappa-B inhibitor beta	46	37748	4,7	1	1	1	1	1	0,06	0,12
RM28_HUMAN	39S ribosomal protein L28, mitochondrial	46	30138	8,34	2	2	2	2	2	0,16	0,32
DCNL1_HUMAN	DCN1-like protein 1	46	30105	5,18	1	1	1	1	1	0,11	0,15
CPT1A_HUMAN	Carnitine O-palmitoyltransferase 1, liver isoform	46	88311	8,85	6	1	3	1	3	0,12	0,05
THOP1_HUMAN	Thimet oligopeptidase	46	78789	5,72	8	2	7	2	7	0,18	0,11
BACH_HUMAN	Cytosolic acyl coenzyme A thioester hydrolase	46	41769	8,85	5	1	3	1	3	0,13	0,1
NRBP_HUMAN	Nuclear receptor-binding protein	45	59807	5,02	3	2	2	1	2	0,09	0,15
PNPO_HUMAN	Pyridoxine-5'-phosphate oxidase	45	29969	6,62	2	1	1	1	1	0,07	0,15
NU205_HUMAN	Nuclear pore complex protein Nup205	45	227776	5,81	8	1	5	1	5	0,05	0,02
EP15R_HUMAN	Epidermal growth factor receptor substrate 15-like 1	45	94197	4,93	3	1	3	1	3	0,08	0,05
SF3B1_HUMAN	Splicing factor 3B subunit 1	45	145738	6,65	6	3	3	1	3	0,05	0,03
EPG5_HUMAN	Ectopic P granules protein 5 homolog	45	292293	5,99	6	1	5	1	5	0,04	0,01
PXDN_HUMAN	Peroxidasin homolog	45	165170	6,79	5	3	4	3	4	0,06	0,08
MYCB2_HUMAN	E3 ubiquitin-protein ligase MYCBP2	45	509759	6,63	24	3	17	1	17	0,06	0,01
CSN7A_HUMAN	COP9 signalosome complex subunit 7a	44	30258	8,33	2	2	1	1	1	0,11	0,15
DCTN2_HUMAN	Dynactin subunit 2	44	44204	5,1	3	1	3	1	3	0,12	0,1
RMD3_HUMAN	Regulator of microtubule dynamics protein 3	44	52086	5,02	2	2	2	2	2	0,1	0,17

[a]Protein score is calculated from the score of the peptide attributed to the protein.
[b]pI is (Predicted) isoelectric point.
[c]Number of matches is spectrum number matched to protein[#1].
[d]Number of significant matches is spectrum number that matches protein and exceeds the identification criteria.
[e]Number of sequences is number of peptides matched to protein[#2].
[f]Number of significant sequences is number of peptides exceeding the identification criteria matched to proteins.
[g]Number of unique sequences is a unique[#3] number of peptides matched to proteins.
[h]Sequence coverage is the ratio of the total number of matched peptide residues to the total length of the protein.
[i]Exponentially Modified Protein Abundance Index (http://www.matrixscience.com/help/quant_empai_help.html).

Table S1. Cont 9.

UniProt/SWISS-PROT ID	Description	Protein score[a]	Protein mass (kDa)	pI[b]	Num. of matches[c]	Num. of significant matches[d]	Num. of sequences[e]	Num. of significant sequences[f]	Num. of unique sequences[g]	Sequence coverage[h]	emPAI[i]
BOP1_HUMAN	Ribosome biogenesis protein BOP1	44	83577	5,8	4	3	1	1	1	0,04	0,11
FAH2A_HUMAN	Fumarylacetoacetate hydrolase domain−containing protein 2A	44	34574	8,48	7	4	2	1	2	0,17	0,13
ASC_HUMAN	Apoptosis−associated speck−like protein containing a CARD	44	21613	5,95	2	1	2	1	2	0,23	0,21
TRAM1_HUMAN	Translocating chain−associated membrane protein 1	43	43044	9,65	2	2	1	1	1	0,06	0,1
STX16_HUMAN	Syntaxin−16	43	37008	5,75	4	2	2	1	2	0,1	0,12
CN37_HUMAN	2',3'−cyclic−nucleotide 3'−phosphodiesterase	43	47549	9,17	4	1	4	1	4	0,21	0,09
ROCK2_HUMAN	Rho−associated protein kinase 2	43	160799	5,75	19	2	10	2	10	0,09	0,05
TMM43_HUMAN	Transmembrane protein 43	43	44847	7,86	4	1	3	1	3	0,21	0,1
AHSA1_HUMAN	Activator of 90 kDa heat shock protein ATPase homolog 1	43	38250	5,41	1	1	1	1	1	0,04	0,11
CIRBP_HUMAN	Cold−inducible RNA−binding protein	43	18637	9,51	2	1	2	1	2	0,27	0,25
STX12_HUMAN	Syntaxin−12	43	31622	5,45	3	1	3	1	3	0,27	0,14
CBPD_HUMAN	Carboxypeptidase D	43	152835	5,68	3	1	3	1	3	0,06	0,03
UB2L6_HUMAN	Ubiquitin/ISG15−conjugating enzyme E2 L6	43	17757	7,71	3	1	3	1	3	0,39	0,26
NDRG1_HUMAN	Protein NDRG1	43	42808	5,49	7	3	3	2	3	0,17	0,21
COMD8_HUMAN	COMM domain−containing protein 8	43	21077	5,29	6	3	2	1	2	0,22	0,48
DFFA_HUMAN	DNA fragmentation factor subunit alpha	43	36500	4,68	5	3	2	1	2	0,14	0,12
CH033_HUMAN	UPF0488 protein C8orf33	42	24977	9,99	1	1	1	1	1	0,14	0,18
TIPRL_HUMAN	TIP41−like protein	42	31424	5,6	4	1	3	1	3	0,25	0,14
PEX19_HUMAN	Peroxisomal biogenesis factor 19	42	32786	4,26	2	2	1	1	1	0,1	0,14
AT5F1_HUMAN	ATP synthase F(0) complex subunit B1, mitochondrial	42	28890	9,37	3	2	3	2	3	0,23	0,33
NOL9_HUMAN	Polynucleotide 5'−hydroxyl−kinase NOL9	42	79272	9,3	1	1	1	1	1	0,04	0,05
SUN2_HUMAN	SUN domain−containing protein 2	42	80262	6,27	4	1	2	1	2	0,08	0,05
HNRPQ_HUMAN	Heterogeneous nuclear ribonucleoprotein Q	38	69560	8,68	5	3	3	2	3	0,1	0,13
ARHG7_HUMAN	Rho guanine nucleotide exchange factor 7	42	89955	6,66	6	2	4	2	4	0,1	0,1
COG3_HUMAN	Conserved oligomeric Golgi complex subunit 3	42	94036	5,39	5	2	2	1	2	0,05	0,05
TB182_HUMAN	182 kDa tankyrase−1−binding protein	42	181685	4,77	2	2	2	2	2	0,02	0,05
GGYF2_HUMAN	GRB10−interacting GYF protein 2	42	149978	5,45	3	2	2	1	2	0,03	0,06
SRP68_HUMAN	Signal recognition particle subunit SRP68	42	70686	8,75	9	1	6	1	6	0,19	0,06
AKAP8_HUMAN	A−kinase anchor protein 8	41	76061	5,03	1	1	1	1	1	0,03	0,06
SLFN5_HUMAN	Schlafen family member 5	41	100990	8,54	4	1	4	1	4	0,11	0,04
GNAS1_HUMAN	Guanine nucleotide−binding protein G(s) subunit alpha isoforms XLas	41	110956	4,91	7	2	5	1	5	0,09	0,04
ILVBL_HUMAN	Acetolactate synthase−like protein	41	67825	8,43	1	1	1	1	1	0,05	0,06
RBP56_HUMAN	TATA−binding protein−associated factor 2N	41	61793	8,04	7	1	3	1	2	0,1	0,07
S23IP_HUMAN	SEC23−interacting protein	41	111007	5,35	11	1	6	1	6	0,14	0,04
DP13A_HUMAN	DCC−interacting protein 13−alpha	41	79614	5,28	2	2	1	1	1	0,03	0,05
ARLY_HUMAN	Argininosuccinate lyase	41	51625	6,04	3	2	3	2	3	0,15	0,18
ADK_HUMAN	Adenosine kinase	41	40520	6,24	4	2	3	1	3	0,2	0,11
S17A5_HUMAN	Sialin	41	54604	8,51	10	3	3	2	3	0,19	0,16
TIM13_HUMAN	Mitochondrial import inner membrane translocase subunit Tim13	41	10493	8,42	2	1	2	1	2	0,31	0,47
SCRIB_HUMAN	Protein scribble homolog	40	174778	5,01	4	1	4	1	4	0,06	0,02
PEMT_HUMAN	Phosphatidylethanolamine N−methyltransferase	40	22120	8,89	1	1	1	1	1	0,16	0,21
FBLN5_HUMAN	Fibulin−5	40	50147	4,58	6	3	4	3	4	0,21	0,28
SCPDL_HUMAN	Saccharopine dehydrogenase−like oxidoreductase	40	47121	9,24	3	1	3	1	3	0,19	0,09
VAPA_HUMAN	Vesicle−associated membrane protein−associated protein A	40	27875	8,8	5	1	3	1	3	0,21	0,16
SAC1_HUMAN	Phosphatidylinositide phosphatase SAC1	40	66924	6,66	1	1	1	1	1	0,06	0,06
DHB12_HUMAN	Very−long−chain 3−oxoacyl−CoA reductase	40	34302	9,34	12	5	5	3	5	0,37	0,44
DHRS4_HUMAN	Dehydrogenase/reductase SDR family member 4	40	29518	8,81	9	4	3	2	3	0,26	0,32
ABCE1_HUMAN	ATP−binding cassette sub−family E member 1	40	67271	8,63	8	3	6	3	6	0,26	0,2
CHIP_HUMAN	E3 ubiquitin−protein ligase CHIP	40	34834	5,61	2	1	1	1	1	0,08	0,13
GLOD4_HUMAN	Glyoxalase domain−containing protein 4	40	34771	5,4	5	2	2	1	2	0,14	0,13
TOM34_HUMAN	Mitochondrial import receptor subunit TOM34	39	34538	9,12	1	1	1	1	1	0,06	0,13
TRML2_HUMAN	Trem−like transcript 2 protein	39	35104	9,68	1	1	1	1	1	0,03	0,13
SRP72_HUMAN	Signal recognition particle subunit SRP72	39	74560	9,31	9	4	6	3	6	0,17	0,18
BAG5_HUMAN	BAG family molecular chaperone regulator 5	39	51168	5,76	1	1	1	1	1	0,05	0,08
CUL1_HUMAN	Cullin−1	39	89622	8,19	2	1	2	1	2	0,04	0,05
EMAL2_HUMAN	Echinoderm microtubule−associated protein−like 2	39	70634	5,86	6	2	4	1	4	0,18	0,06
LBR_HUMAN	Lamin−B receptor	39	70658	9,41	2	1	1	1	1	0,04	0,06
IDI1_HUMAN	Isopentenyl−diphosphate Delta−isomerase 1	39	26302	5,93	4	1	4	1	4	0,36	0,17
CTBP1_HUMAN	C−terminal−binding protein 1	39	47505	6,28	3	1	2	1	2	0,12	0,09
TPP2_HUMAN	Tripeptidyl−peptidase 2	38	138263	5,9	7	3	3	1	3	0,05	0,03
MYO1D_HUMAN	Unconventional myosin−Id	38	116129	9,44	23	2	8	2	8	0,12	0,07
MANF_HUMAN	Mesencephalic astrocyte−derived neurotrophic factor	38	20687	8,93	1	1	1	1	1	0,08	0,22
DD19B_HUMAN	ATP−dependent RNA helicase DDX19B	38	53893	5,96	4	1	4	1	4	0,23	0,08
SPON2_HUMAN	Spondin−2	38	35824	5,35	3	2	1	1	1	0,08	0,12
MIC60_HUMAN	MICOS complex subunit MIC60	38	83626	6,08	3	1	2	1	2	0,07	0,05
PCKGM_HUMAN	Phosphoenolpyruvate carboxykinase [GTP], mitochondrial	38	70685	7,57	4	2	4	2	4	0,15	0,13
NB5R1_HUMAN	NADH−cytochrome b5 reductase 1	38	34073	9,41	3	1	3	1	3	0,25	0,13
SEC63_HUMAN	Translocation protein SEC63 homolog	38	87942	5,21	7	3	4	2	4	0,07	0,1
PA1B3_HUMAN	Platelet−activating factor acetylhydrolase IB subunit gamma	38	25718	6,33	1	1	1	1	1	0,1	0,17
LEMD2_HUMAN	LEM domain−containing protein 2	38	56940	9,16	1	1	1	1	1	0,06	0,08
SDHA_HUMAN	Succinate dehydrogenase [ubiquinone] flavoprotein subunit, mitochondrial	36	72645	7,06	8	4	5	3	5	0,2	0,19
PTRD1_HUMAN	Putative peptidyl−tRNA hydrolase PTRHD1	38	15795	9,2	2	2	1	1	1	0,18	0,3
ARMX3_HUMAN	Armadillo repeat−containing X−linked protein 3	37	42474	8,6	5	1	3	1	3	0,16	0,1
RCN2_HUMAN	Reticulocalbin−2	37	36854	4,26	3	3	3	3	3	0,16	0,4
ABC3C_HUMAN	DNA dC−>dU−editing enzyme APOBEC−3C	37	22811	7,52	1	1	1	1	1	0,08	0,2
DHB11_HUMAN	Estradiol 17−beta−dehydrogenase 11	37	32915	9,2	1	1	1	1	1	0,05	0,13
QCR6_HUMAN	Cytochrome b−c1 complex subunit 6, mitochondrial	37	10732	4,39	2	2	1	1	1	0,2	0,46
METK2_HUMAN	S−adenosylmethionine synthase isoform type−2	37	43633	6,02	5	2	2	1	2	0,1	0,1
SNX18_HUMAN	Sorting nexin−18	37	68851	5,44	5	1	4	1	4	0,12	0,06
SGTA_HUMAN	Small glutamine−rich tetratricopeptide repeat−containing protein alpha	37	34042	4,81	3	2	2	1	2	0,05	0,13
TPC2A_HUMAN	Trafficking protein particle complex subunit 2	37	16434	6,02	1	1	1	1	1	0,09	0,28
OGA_HUMAN	Protein O−GlcNAcase	36	102849	4,82	2	1	2	1	2	0,04	0,04
GPDM_HUMAN	Glycerol−3−phosphate dehydrogenase, mitochondrial	36	80802	7,57	3	1	2	1	2	0,07	0,05
COASY_HUMAN	Bifunctional coenzyme A synthase	36	62290	6,51	9	3	3	2	3	0,1	0,14
PHOCN_HUMAN	MOB−like protein phocein	36	26016	5,5	2	2	1	1	1	0,13	0,17
CPT2_HUMAN	Carnitine O−palmitoyltransferase 2, mitochondrial	36	73730	8,38	8	2	5	2	5	0,15	0,12
TBRG4_HUMAN	Protein TBRG4	36	70693	6,98	6	1	4	1	4	0,12	0,06
PAIRB_HUMAN	Plasminogen activator inhibitor 1 RNA−binding protein	36	44938	8,66	6	2	2	1	2	0,12	0,1
ATLA1_HUMAN	Atlastin−1	36	63503	5,82	4	2	3	1	3	0,13	0,07
GRWD1_HUMAN	Glutamate−rich WD repeat−containing protein 1	36	49388	4,82	1	1	1	1	1	0,05	0,09
BCAT1_HUMAN	Branched−chain−amino−acid aminotransferase, cytosolic	36	42939	5,17	5	1	3	1	3	0,2	0,1
SHPK_HUMAN	Sedoheptulokinase	36	51458	6,35	2	1	2	1	2	0,07	0,08
GALE_HUMAN	UDP−glucose 4−epimerase	36	38257	6,26	2	1	2	1	2	0,19	0,11
SNX2_HUMAN	Sorting nexin−2	36	58435	5,04	7	2	4	1	4	0,13	0,07
TNR6_HUMAN	Tumor necrosis factor receptor superfamily member 6	36	37708	8,29	3	2	2	1	2	0,13	0,12
XG_HUMAN	Glycoprotein Xg	35	19710	6,96	1	1	1	1	1	0,12	0,23
DAD1_HUMAN	Dolichyl−diphosphooligosaccharide—protein glycosyltransferase subunit DAD1	35	12489	6,52	4	1	2	1	2	0,29	0,38
MTAP_HUMAN	S−methyl−5'−thioadenosine phosphorylase	35	31216	6,75	7	2	2	1	2	0,18	0,14
PTPS_HUMAN	6−pyruvoyl tetrahydrobiopterin synthase	35	16375	6,2	2	2	1	1	1	0,2	0,28
TFG_HUMAN	Protein TFG	35	43421	4,94	13	1	5	1	5	0,25	0,1

[a]Protein score is calculated from the score of the peptide attributed to the protein.
[b]pI is (Predicted) isoelectric point.
[c]Number of matches is spectrum number matched to protein[#1].
[d]Number of significant matches is spectrum number that matches protein and exceeds the identification criteria.
[e]Number of sequences is number of peptides matched to protein[#2].
[f]Number of significant sequences is number of peptides exceeding the identification criteria matched to proteins.
[g]Number of unique sequences is a unique[#3] number of peptides matched to proteins.
[h]Sequence coverage is the ratio of the total number of matched peptide residues to the total length of the protein.
[i]Exponentially Modified Protein Abundance Index (http://www.matrixscience.com/help/quant_empai_help.html).

Table S1. Cont 10.

UniProt/SWISS-PROT ID	Description	Protein score[a]	Protein mass (kDa)	pI[b]	Num. of matches[c]	Num. of significant matches[d]	Num. of sequences[e]	Num. of significant sequences[f]	Num. of unique sequences[g]	Sequence coverage[h]	emPAI[i]
XPO5_HUMAN	Exportin-5	35	136222	5.56	8	2	3	1	3	0.05	0.06
GSHR_HUMAN	Glutathione reductase, mitochondrial	35	56221	8.74	7	2	4	2	4	0.16	0.16
HMOX1_HUMAN	Heme oxygenase 1	35	32798	7.88	5	1	3	1	3	0.19	0.14
ASNS_HUMAN	Asparagine synthetase [glutamine-hydrolyzing]	35	64329	6.39	4	1	4	1	4	0.19	0.07
SPART_HUMAN	Spartin	35	72788	5.66	3	2	2	2	2	0.11	0.12
PON2_HUMAN	Serum paraoxonase/arylesterase 2	35	39372	5.33	3	1	2	1	2	0.19	0.11
GLMP_HUMAN	Glycosylated lysosomal membrane protein	35	43836	6.1	2	1	1	1	1	0.05	0.1
TGO1_HUMAN	Transport and Golgi organization protein 1 homolog	35	213570	4.76	5	2	3	1	3	0.04	0.02
EIF2A_HUMAN	Eukaryotic translation initiation factor 2A	34	64949	9	2	2	1	1	1	0.03	0.07
TMED3_HUMAN	Transmembrane emp24 domain-containing protein 3	34	24761	5.4	1	1	1	1	1	0.13	0.18
EXOC2_HUMAN	Exocyst complex component 2	34	104000	6.46	10	1	6	1	6	0.16	0.04
GLMN_HUMAN	Glomulin	34	68165	5.24	1	1	1	1	1	0.05	0.06
IFIT5_HUMAN	Interferon-induced protein with tetratricopeptide repeats 5	34	55812	7	4	1	4	1	4	0.21	0.08
RIC8A_HUMAN	Synembryn-A	34	59671	5.2	16	1	4	1	4	0.13	0.07
ERG7_HUMAN	Lanosterol synthase	34	83255	6.16	2	1	1	1	1	0.02	0.05
DCPS_HUMAN	m7GpppX diphosphatase	34	38585	5.93	2	2	2	2	2	0.1	0.24
C1TM_HUMAN	Monofunctional C1-tetrahydrofolate synthase, mitochondrial	34	105724	8.32	1	1	1	1	1	0.02	0.04
USP9X_HUMAN	Probable ubiquitin carboxyl-terminal hydrolase FAF-X	34	292094	5.52	18	1	11	1	11	0.08	0.01
TRXR1_HUMAN	Thioredoxin reductase 1, cytoplasmic	33	70862	7.16	5	2	4	2	4	0.12	0.13
DPM1_HUMAN	Dolichol-phosphate mannosyltransferase subunit 1	33	29616	9.57	7	1	4	1	4	0.34	0.15
NSF1C_HUMAN	NSFL1 cofactor p47	33	40548	4.99	3	1	2	1	2	0.11	0.11
RM54_HUMAN	39S ribosomal protein L54, mitochondrial	33	15809	9.6	1	1	1	1	1	0.19	0.3
TXNL1_HUMAN	Thioredoxin-like protein 1	33	32231	4.84	4	2	3	2	3	0.21	0.29
SF3B4_HUMAN	Splicing factor 3B subunit 4	33	44357	8.54	2	1	2	1	2	0.11	0.1
GSKIP_HUMAN	GSK3B-interacting protein	33	15638	4.36	3	1	2	1	2	0.19	0.3
CD68_HUMAN	Macrosialin	33	37385	9.1	2	1	1	1	1	0.09	0.12
ODBB_HUMAN	2-oxoisovalerate dehydrogenase subunit beta, mitochondrial	33	43095	5.89	3	1	3	1	3	0.17	0.1
COMD2_HUMAN	COMM domain-containing protein 2	32	22731	6.24	5	2	3	1	3	0.24	0.2
UACA_HUMAN	Uveal autoantigen with coiled-coil domains and ankyrin repeats	32	162404	6.6	22	1	6	1	6	0.07	0.03
RO52_HUMAN	E3 ubiquitin-protein ligase TRIM21	32	54135	5.98	5	1	5	1	5	0.17	0.08
CDK4_HUMAN	Cyclin-dependent kinase 4	32	33708	6.51	6	2	3	1	3	0.28	0.13
VP37A_HUMAN	Vacuolar protein sorting-associated protein 37A	32	44287	5.37	1	1	1	1	1	0.1	0.1
ITA2_HUMAN	Integrin alpha-2	32	129214	5.16	5	1	5	1	5	0.11	0.03
ACATN_HUMAN	Acetyl-coenzyme A transporter 1	32	60870	6.99	4	2	2	1	2	0.11	0.07
S39A9_HUMAN	Zinc transporter ZIP9	32	32231	6.21	1	1	1	1	1	0.07	0.14
EXOC7_HUMAN	Exocyst complex component 7	32	83330	6.33	5	3	4	2	4	0.12	0.11
S52A1_HUMAN	Solute carrier family 52, riboflavin transporter, member 1	32	46287	5.9	2	2	1	1	1	0.05	0.09
RT16_HUMAN	28S ribosomal protein S16, mitochondrial	32	15335	9.58	1	1	1	1	1	0.15	0.31
BGAL_HUMAN	Beta-galactosidase	32	76027	6.1	3	1	2	1	2	0.09	0.06
M2OM_HUMAN	Mitochondrial 2-oxoglutarate/malate carrier protein	32	34040	9.92	4	1	3	1	3	0.22	0.13
FBLN1_HUMAN	Fibulin-1	32	77162	5.07	6	4	4	3	4	0.12	0.18
KDM6B_HUMAN	Lysine-specific demethylase 6B	32	176523	8.83	12	1	5	1	5	0.07	0.02
PSME4_HUMAN	Proteasome activator complex subunit 4	31	211199	6.45	4	1	3	1	3	0.03	0.02
AGAL_HUMAN	Alpha-galactosidase A	31	48735	5.35	3	2	1	1	1	0.06	0.09
EBPL_HUMAN	Emopamil-binding protein-like	31	23189	5.91	2	2	1	1	1	0.15	0.19
CD9_HUMAN	CD9 antigen	31	25399	6.8	3	2	2	1	2	0.27	0.18
PCYOX_HUMAN	Prenylcysteine oxidase 1	31	56604	5.8	3	2	1	1	1	0.05	0.08
XPO4_HUMAN	Exportin-4	31	130056	4.95	9	2	7	2	7	0.13	0.07
SF3A3_HUMAN	Splicing factor 3A subunit 3	31	58812	5.27	2	1	2	1	2	0.1	0.07
CAB45_HUMAN	45 kDa calcium-binding protein	31	41780	4.76	5	1	2	1	2	0.14	0.1
CD276_HUMAN	CD276 antigen	31	57199	4.77	2	2	2	2	2	0.09	0.16
TX264_HUMAN	Testis-expressed protein 264	31	34167	4.79	1	1	1	1	1	0.09	0.13
CCAR1_HUMAN	Cell division cycle and apoptosis regulator protein 1	31	132739	5.57	7	1	4	1	4	0.06	0.03
KDEL1_HUMAN	KDEL motif-containing protein 1	31	58005	7.59	1	1	1	1	1	0.03	0.07
EGLN_HUMAN	Endoglin	31	70533	6.14	8	2	6	1	6	0.13	0.06
FUCO_HUMAN	Tissue alpha-L-fucosidase	31	53655	6.37	1	1	1	1	1	0.08	0.08
AFAM_HUMAN	Afamin	31	69024	5.64	5	2	3	1	3	0.12	0.06
PP6R1_HUMAN	Serine/threonine-protein phosphatase 6 regulatory subunit 1	31	96664	4.45	4	2	2	1	2	0.05	0.04
CT027_HUMAN	UPF0687 protein C20orf27	30	19279	6.34	2	1	2	1	2	0.16	0.24
IRGQ_HUMAN	Immunity-related GTPase family Q protein	30	62678	4.81	3	2	1	1	1	0.05	0.07
YIF1A_HUMAN	Protein YIF1A	30	31991	8.98	3	2	3	2	3	0.19	0.3
CARM1_HUMAN	Histone-arginine methyltransferase CARM1	30	65811	6.25	6	3	4	2	4	0.17	0.21
KBL_HUMAN	2-amino-3-ketobutyrate coenzyme A ligase, mitochondrial	30	45256	8.3	2	1	2	1	2	0.14	0.1
NAV1_HUMAN	Neuron navigator 1	30	202347	8.2	9	2	6	1	6	0.07	0.04
CASP9_HUMAN	Caspase-9	30	46251	5.73	1	1	1	1	1	0.06	0.09
GORS2_HUMAN	Golgi reassembly-stacking protein 2	30	47116	4.73	2	1	2	1	2	0.08	0.09
NIPS2_HUMAN	Protein NipSnap homolog 2	30	33721	9.42	3	1	2	1	2	0.15	0.13
SAHH2_HUMAN	S-adenosylhomocysteine hydrolase-like protein 1	30	58913	6.49	6	1	6	1	6	0.25	0.07
CSN3_HUMAN	COP9 signalosome complex subunit 3	30	47842	6.19	9	1	8	1	8	0.44	0.09
GSH0_HUMAN	Glutamate--cysteine ligase regulatory subunit	30	30708	5.69	1	1	1	1	1	0.11	0.14
MAP1S_HUMAN	Microtubule-associated protein 1S	29	112142	6.92	3	1	3	1	3	0.05	0.04
PGH1_HUMAN	Prostaglandin G/H synthase 1	29	68642	6.81	2	2	2	2	2	0.09	0.13
MOT4_HUMAN	Monocarboxylate transporter 4	29	49437	8.23	4	2	1	1	1	0.03	0.09
NAGK_HUMAN	N-acetyl-D-glucosamine kinase	29	37352	5.81	9	1	3	1	3	0.15	0.12
NUCB2_HUMAN	Nucleobindin-2	29	50164	5.03	3	2	2	1	2	0.08	0.09
RIPK2_HUMAN	Receptor-interacting serine/threonine-protein kinase 2	29	61156	6.63	2	1	2	1	2	0.09	0.07
LMO7_HUMAN	LIM domain only protein 7	29	192576	8.34	10	1	5	1	5	0.05	0.02
ARAID_HUMAN	All-trans retinoic acid-induced differentiation factor	29	24730	6.95	2	1	1	1	1	0.17	0.18
PDXD2_HUMAN	Putative pyridoxal-dependent decarboxylase domain-containing protein 2	29	51777	5.65	5	2	5	2	5	0.26	0.17
DNS2A_HUMAN	Deoxyribonuclease-2-alpha	29	39556	8.3	4	2	2	1	2	0.15	0.23
LEG3_HUMAN	Galectin-3	29	26136	8.57	5	1	4	1	4	0.22	0.17
PPIF_HUMAN	Peptidyl-prolyl cis-trans isomerase F, mitochondrial	28	22026	9.48	2	2	2	2	2	0.27	0.46
DYH10_HUMAN	Dynein heavy chain 10, axonemal	28	514512	5.64	13	1	10	1	10	0.06	0.01
LIPB1_HUMAN	Liprin-beta-1	28	113952	5.4	8	1	7	1	7	0.13	0.04
MALD1_HUMAN	MARVEL domain-containing protein 1	28	18902	9.69	2	1	1	1	1	0.17	0.24
EDIL3_HUMAN	EGF-like repeat and discoidin I-like domain-containing protein 3	28	53730	7.08	2	1	2	1	2	0.11	0.08
AIMP1_HUMAN	Aminoacyl tRNA synthase complex-interacting multifunctional protein 1	28	34331	8.61	3	2	3	2	3	0.17	0.27
SNX5_HUMAN	Sorting nexin-5	28	46787	6.31	1	1	1	1	1	0.05	0.09
MA1A2_HUMAN	Mannosyl-oligosaccharide 1,2-alpha-mannosidase IB	28	72957	7.17	2	1	1	1	1	0.03	0.06
FKBP4_HUMAN	Peptidyl-prolyl cis-trans isomerase FKBP4	28	51772	5.35	7	1	5	1	5	0.15	0.08
PSMG4_HUMAN	Proteasome assembly chaperone 4	28	13766	6.04	1	1	1	1	1	0.13	0.35
UBQL1_HUMAN	Ubiquilin-1	28	62479	5.02	4	1	3	1	3	0.16	0.07
XPOT_HUMAN	Exportin-T	28	109893	5.24	2	1	2	1	2	0.05	0.04
HEM6_HUMAN	Oxygen-dependent coproporphyrinogen-III oxidase, mitochondrial	28	50120	8.59	4	1	3	1	3	0.2	0.09
NIT2_HUMAN	Omega-amidase NIT2	28	30589	6.82	6	1	3	1	3	0.23	0.14
EMP3_HUMAN	Epithelial membrane protein 3	27	18417	8.18	1	1	1	1	1	0.21	0.25
WASC5_HUMAN	WASH complex subunit 5	27	134201	6.54	5	2	4	2	4	0.05	0.06
NU155_HUMAN	Nuclear pore complex protein Nup155	27	155100	5.78	8	1	4	1	4	0.09	0.03
UBP2L_HUMAN	Ubiquitin-associated protein 2-like	27	114465	6.61	3	1	3	1	3	0.05	0.04
WAC2A_HUMAN	WASH complex subunit 2A	27	147095	4.69	3	1	3	1	3	0.04	0.03

[a]Protein score is calculated from the score of the peptide attributed to the protein.
[b]pI is (Predicted) isoelectric point.
[c]Number of matches is spectrum number matched to protein[#1].
[d]Number of significant matches is spectrum number that matches protein and exceeds the identification criteria.
[e]Number of sequences is number of peptides matched to protein[#2].
[f]Number of significant sequences is number of peptides exceeding the identification criteria matched to proteins.
[g]Number of unique sequences is a unique[#3] number of peptides matched to proteins.
[h]Sequence coverage is the ratio of the total number of matched peptide residues to the total length of the protein.
[i]Exponentially Modified Protein Abundance Index (http://www.matrixscience.com/help/quant_empai_help.html).

Table S1. Cont 11.

UniProt/SWISS-PROT ID	Description	Protein score[a]	Protein mass (kDa)	pI[b]	Num. of matches[c]	Num. of significant matches[d]	Num. of sequences[e]	Num. of significant sequences[f]	Num. of unique sequences[g]	Sequence coverage[h]	emPAI[i]
SRRM1_HUMAN	Serine/arginine repetitive matrix protein 1	27	102274	11.84	6	2	3	1	3	0.09	0.09
CD97_HUMAN	CD97 antigen	27	91809	6.5	2	1	2	1	2	0.07	0.05
SORCN_HUMAN	Sorcin	27	21662	5.32	5	1	3	1	3	0.22	0.21
MTNA_HUMAN	Methylthioribose-1-phosphate isomerase	27	39125	5.89	2	1	2	1	2	0.21	0.11
GPI8_HUMAN	GPI-anchor transamidase	27	45223	5.76	2	1	1	1	1	0.07	0.1
NUP88_HUMAN	Nuclear pore complex protein Nup88	27	83489	5.47	3	1	2	1	2	0.06	0.05
GGH_HUMAN	Gamma-glutamyl hydrolase	27	35941	6.67	1	1	1	1	1	0.08	0.12
ANX11_HUMAN	Annexin A11	27	54355	7.53	2	1	2	1	2	0.07	0.08
IF2B_HUMAN	Eukaryotic translation initiation factor 2 subunit 2	27	38364	5.6	3	1	3	1	3	0.15	0.11
IFIT1_HUMAN	Interferon-induced protein with tetratricopeptide repeats 1	27	55325	6.75	3	1	3	1	3	0.13	0.08
RRAGC_HUMAN	Ras-related GTP-binding protein C	27	44196	4.94	1	1	1	1	1	0.06	0.1
NRBF2_HUMAN	Nuclear receptor-binding factor 2	27	32358	5.61	1	1	1	1	1	0.1	0.14
TARA_HUMAN	TRIO and F-actin-binding protein	26	261217	8.86	10	1	7	1	7	0.05	0.02
GCSH_HUMAN	Glycine cleavage system H protein, mitochondrial	26	18873	4.91	3	1	2	1	2	0.29	0.24
CHID1_HUMAN	Chitinase domain-containing protein 1	26	44912	8.74	6	2	4	2	4	0.26	0.2
NEK9_HUMAN	Serine/threonine-protein kinase Nek9	26	107100	5.51	8	2	6	1	6	0.13	0.04
ENAH_HUMAN	Protein enabled homolog	26	66470	6.51	14	2	3	1	3	0.08	0.13
PARP1_HUMAN	Poly [ADP-ribose] polymerase 1	26	113012	8.99	5	1	3	1	3	0.09	0.04
ODPB_HUMAN	Pyruvate dehydrogenase E1 component subunit beta, mitochondrial	26	39208	6.2	12	3	3	2	3	0.21	0.24
LONM_HUMAN	Lon protease homolog, mitochondrial	26	106422	6.01	10	2	5	1	5	0.16	0.04
FA98B_HUMAN	Protein FAM98B	26	37167	5.96	2	1	2	1	2	0.09	0.12
UB2G1_HUMAN	Ubiquitin-conjugating enzyme E2 G1	26	19497	5.21	2	1	2	1	2	0.35	0.23
COX2_HUMAN	Cytochrome c oxidase subunit 2	26	25548	4.67	8	1	3	1	3	0.3	0.18
KDM1A_HUMAN	Lysine-specific histone demethylase 1A	26	92845	6.11	5	2	4	1	4	0.12	0.05
ACDSB_HUMAN	Short/branched chain specific acyl-CoA dehydrogenase, mitochondrial	26	47455	6.53	2	1	2	1	2	0.09	0.09
TTL12_HUMAN	Tubulin--tyrosine ligase-like protein 12	26	74356	5.33	3	1	2	1	2	0.08	0.06
PRP8_HUMAN	Pre-mRNA-processing-splicing factor 8	26	273427	8.95	7	1	5	1	5	0.04	0.02
UMPS_HUMAN	Uridine 5'-monophosphate synthase	25	52189	6.81	1	1	1	1	1	0.07	0.08
BGIN_HUMAN	Bargin	25	73553	5.13	2	1	2	1	2	0.05	0.06
SCFD2_HUMAN	Sec1 family domain-containing protein 2	25	75079	6.21	4	1	3	1	3	0.12	0.06
SMD3_HUMAN	Small nuclear ribonucleoprotein Sm D3	25	13907	10.33	1	1	1	1	1	0.17	0.34
LYPL1_HUMAN	Lysophospholipase-like protein 1	25	26299	7.71	3	1	2	1	2	0.21	0.17
BET1L_HUMAN	BET1-like protein	25	12380	7.88	3	1	3	1	3	0.47	0.39
SNF8_HUMAN	Vacuolar-sorting protein SNF8	24	28846	6.2	1	1	1	1	1	0.14	0.15
SNAG_HUMAN	Gamma-soluble NSF attachment protein	24	34724	5.3	4	1	4	1	4	0.24	0.13
TPRGL_HUMAN	Tumor protein p63-regulated gene 1-like protein	24	30193	6.85	4	1	2	1	2	0.19	0.15
KPRA_HUMAN	Phosphoribosyl pyrophosphate synthase-associated protein 1	24	39369	6.73	2	1	2	1	2	0.09	0.11
LANC1_HUMAN	LanC-like protein 1	24	45254	7.86	3	1	2	1	2	0.16	0.1
DESI1_HUMAN	Desumoylating isopeptidase 1	24	18251	4.86	1	1	1	1	1	0.15	0.25
ABHDB_HUMAN	Protein ABHD11	24	34668	9.5	5	1	2	1	2	0.16	0.13
CRKL_HUMAN	Crk-like protein	24	33756	6.26	2	1	2	1	2	0.11	0.13
CCD80_HUMAN	Coiled-coil domain-containing protein 80	23	108106	9.72	11	2	5	1	5	0.09	0.04
DNPEP_HUMAN	Aspartyl aminopeptidase	23	52395	7.03	4	1	2	1	2	0.08	0.08
PARP4_HUMAN	Poly [ADP-ribose] polymerase 4	23	192472	5.43	11	1	7	1	7	0.07	0.02
ERG1_HUMAN	Squalene monooxygenase	23	63882	8.8	3	1	2	1	2	0.06	0.07
PLCG1_HUMAN	1-phosphatidylinositol 4,5-bisphosphate phosphodiesterase gamma-1	23	148438	5.73	5	1	4	1	4	0.06	0.03
ELP1_HUMAN	Elongator complex protein 1	23	150159	5.61	2	1	2	1	2	0.03	0.03
INT3_HUMAN	Integrator complex subunit 3	23	117994	5.53	5	1	4	1	4	0.1	0.04
UBP7_HUMAN	Ubiquitin carboxyl-terminal hydrolase 7	23	128220	5.33	6	2	5	1	5	0.11	0.03
YIPF3_HUMAN	Protein YIPF3	23	38223	5.47	1	1	1	1	1	0.07	0.11
CSPG2_HUMAN	Versican core protein	23	372590	4.43	5	1	3	1	3	0.02	0.01
TRIP6_HUMAN	Thyroid receptor-interacting protein 6	23	50255	7.19	3	1	1	1	1	0.05	0.09
TTC1_HUMAN	Tetratricopeptide repeat protein 1	23	33505	4.78	2	1	2	1	2	0.2	0.13
RANG_HUMAN	Ran-specific GTPase-activating protein	23	23296	5.19	5	1	2	1	2	0.15	0.19
COMD5_HUMAN	COMM domain-containing protein 5	22	24655	6.52	2	1	1	1	1	0.13	0.18
MA2B2_HUMAN	Epididymis-specific alpha-mannosidase	22	113908	6.74	2	1	2	1	2	0.05	0.04
PIGS_HUMAN	GPI transamidase component PIG-S	22	61617	6.05	2	1	1	1	1	0.05	0.07
MARE3_HUMAN	Microtubule-associated protein RP/EB family member 3	22	31962	5.33	2	1	1	1	1	0.09	0.14
HEAT1_HUMAN	HEAT repeat-containing protein 1	22	242215	6.11	7	1	6	1	6	0.05	0.02
VPS16_HUMAN	Vacuolar protein sorting-associated protein 16 homolog	22	94634	6.32	2	1	2	1	2	0.05	0.05
VMP1_HUMAN	Vacuole membrane protein 1	22	46208	6.47	3	1	1	1	1	0.07	0.09
TBC23_HUMAN	TBC1 domain family member 23	22	78272	5.23	2	1	1	1	1	0.04	0.05
PDLI1_HUMAN	PDZ and LIM domain protein 1	22	36049	6.56	7	1	2	1	2	0.14	0.12
GRB2_HUMAN	Growth factor receptor-bound protein 2	22	25190	5.89	2	1	1	1	1	0.13	0.18
PAXI_HUMAN	Paxillin	21	64464	5.8	6	1	5	1	5	0.2	0.07
WFS1_HUMAN	Wolframin	21	100227	8.34	2	1	2	1	2	0.03	0.04
CEP41_HUMAN	Centrosomal protein of 41 kDa	21	41343	8.46	2	1	2	1	2	0.14	0.11
GLTP_HUMAN	Glycolipid transfer protein	21	23834	6.9	3	1	2	1	2	0.29	0.19
VPS53_HUMAN	Vacuolar protein sorting-associated protein 53 homolog	21	79602	5.74	3	1	3	1	3	0.14	0.05
ACADM_HUMAN	Medium-chain specific acyl-CoA dehydrogenase, mitochondrial	20	46559	8.61	5	1	2	1	2	0.1	0.09
CX6B1_HUMAN	Cytochrome c oxidase subunit 6B1	20	10186	6.54	7	1	2	1	2	0.45	0.49
RM44_HUMAN	39S ribosomal protein L44, mitochondrial	20	37512	8.64	1	1	1	1	1	0.12	0.12
SNX9_HUMAN	Sorting nexin-9	20	66550	5.4	3	1	3	1	3	0.13	0.06
PP6R2_HUMAN	Serine/threonine-protein phosphatase 6 regulatory subunit 2	20	104876	4.74	4	1	4	1	4	0.11	0.04
CRLF3_HUMAN	Cytokine receptor-like factor 3	19	49735	5.01	3	1	2	1	2	0.13	0.09
MIC10_HUMAN	MICOS complex subunit MIC10	19	8802	8.68	3	1	1	1	1	0.41	0.58
SYDM_HUMAN	Aspartate--tRNA ligase, mitochondrial	19	73516	8.19	4	3	1	1	1	0.04	0.06
NU214_HUMAN	Nuclear pore complex protein Nup214	19	213488	7.15	1	1	1	1	1	0.02	0.02
GRM1A_HUMAN	GRAM domain-containing protein 1A	19	80630	6.29	2	1	1	1	1	0.05	0.05
EPS8_HUMAN	Epidermal growth factor receptor kinase substrate 8	19	91824	7.1	3	1	3	1	3	0.08	0.05
CPIN1_HUMAN	Anamorsin	19	33561	5.44	4	1	4	1	4	0.17	0.13
MGST3_HUMAN	Microsomal glutathione S-transferase 3	19	16506	9.46	3	3	1	1	1	0.18	0.28
RAVR1_HUMAN	Ribonucleoprotein PTB-binding 1	19	63837	8.79	4	1	2	1	2	0.11	0.07
MINP1_HUMAN	Multiple inositol polyphosphate phosphatase 1	19	55016	7.93	2	1	2	1	2	0.09	0.08
GOSR2_HUMAN	Golgi SNAP receptor complex member 2	19	24760	7.85	1	1	1	1	1	0.09	0.18
VP33A_HUMAN	Vacuolar protein sorting-associated protein 33A	18	67568	6.5	6	1	3	1	3	0.13	0.06
MCM2_HUMAN	DNA replication licensing factor MCM2	18	101832	5.34	5	1	4	1	4	0.09	0.04
CYBP_HUMAN	Calcyclin-binding protein	18	26194	8.28	3	1	2	1	2	0.19	0.17
EMC10_HUMAN	ER membrane protein complex subunit 10	18	27330	5.78	1	1	1	1	1	0.14	0.16
DHX15_HUMAN	Pre-mRNA-splicing factor ATP-dependent RNA helicase DHX15	18	90875	7.12	5	1	4	1	4	0.13	0.05
PARP9_HUMAN	Poly [ADP-ribose] polymerase 9	18	96281	8.12	2	1	2	1	2	0.04	0.04
SL9A7_HUMAN	Sodium/hydrogen exchanger 7	18	80080	5.97	1	1	1	1	1	0.03	0.05
SELO_HUMAN	Selenoprotein O	18	73444	5.69	2	1	2	1	2	0.08	0.06
NCDN_HUMAN	Neurochondrin	17	78814	5.34	7	1	6	1	6	0.13	0.05
PGLT1_HUMAN	Protein O-glucosyltransferase 1	17	46159	8.91	1	1	1	1	1	0.08	0.09
CLU_HUMAN	Clustered mitochondria protein homolog	17	146577	5.75	5	1	5	1	5	0.09	0.03
D2HDH_HUMAN	D-2-hydroxyglutarate dehydrogenase, mitochondrial	16	56380	8.21	2	1	1	1	1	0.05	0.08
DSRAD_HUMAN	Double-stranded RNA-specific adenosine deaminase	15	135981	8.86	4	1	4	1	4	0.06	0.03
PMGE_HUMAN	Bisphosphoglycerate mutase	14	29987	6.1	1	1	1	1	1	0.12	0.15
EXOS6_HUMAN	Exosome complex component MTR3	14	28218	6.06	2	1	1	1	1	0.13	0.16

[a]Protein score is calculated from the score of the peptide attributed to the protein.
[b]pI is (Predicted) isoelectric point.
[c]Number of matches is spectrum number matched to protein[#1].
[d]Number of significant matches is spectrum number that matches protein and exceeds the identification criteria.
[e]Number of sequences is number of peptides matched to protein[#2].
[f]Number of significant sequences is number of peptides exceeding the identification criteria matched to proteins.
[g]Number of unique sequences is a unique[#3] number of peptides matched to proteins.
[h]Sequence coverage is the ratio of the total number of matched peptide residues to the total length of the protein.
[i]Exponentially Modified Protein Abundance Index (http://www.matrixscience.com/help/quant_empai_help.html).

Table S2. Identification of endogenous proteins contained in hADSC cultured in medium, DMEM/10% FBS.

Table S2. Identification of endogenous proteins contained in hADSC cultured in medium, DMEM/10% FBS.

UniProt/SWISS-PROT ID	Description	Protein score[a]	Protein mass (kDa)	pI[b]	Num. of matches[c]	Num. of significant matches[d]	Num. of sequences[e]	Num. of significant sequences[f]	Num. of unique sequences[g]	Sequence coverage[h]	emPAI[i]
MYH9_HUMAN	Myosin-9	7632	226392	5.5	539	301	139	91	119	0.69	6.8
MYH10_HUMAN	Myosin-10	1255	228858	5.44	96	47	50	26	32	0.33	0.67
MYH14_HUMAN	Myosin-14	294	227732	5.52	33	14	19	7	12	0.11	0.14
FLNA_HUMAN	Filamin-A	6172	280564	5.7	620	309	114	85	105	0.63	3.2
FLNC_HUMAN	Filamin-C	2298	290841	5.65	194	123	86	61	20	0.52	1.44
FLNB_HUMAN	Filamin-B	1082	277990	5.47	96	49	59	32	49	0.41	0.64
VIME_HUMAN	Vimentin	6140	53619	5.06	804	380	61	50	15	0.89	132.98
TLN1_HUMAN	Talin-1	3604	269599	5.77	225	145	87	63	80	0.58	1.83
TLN2_HUMAN	Talin-2	161	271443	5.4	20	9	16	5	9	0.1	0.08
LDHA_HUMAN	L-lactate dehydrogenase A chain	3474	36665	8.44	248	156	22	15	15	0.8	6.7
LDHB_HUMAN	L-lactate dehydrogenase B chain	760	36615	5.71	81	40	15	11	13	0.6	3.37
ACTN1_HUMAN	Alpha-actinin-1	3222	102993	5.25	246	141	50	36	10	0.71	4.73
ACTN4_HUMAN	Alpha-actinin-4	1968	104788	5.27	185	94	48	31	28	0.67	2.59
ENOA_HUMAN	Alpha-enolase	3169	47139	7.01	182	103	27	18	17	0.74	7.32
ENOG_HUMAN	Gamma-enolase	510	47239	4.91	29	14	7	5	2	0.25	0.7
G3P_HUMAN	Glyceraldehyde-3-phosphate dehydrogenase	2932	36030	8.57	224	103	19	14	9	0.77	13.13
KPYM_HUMAN	Pyruvate kinase PKM	2871	57900	7.96	221	147	39	33	6	0.78	16.79
TBB2A_HUMAN	Tubulin beta-2A chain	2307	49875	4.78	222	132	28	23	5	0.81	14.77
TBB6_HUMAN	Tubulin beta-6 chain	1150	49825	4.77	130	60	28	20	13	0.82	7.08
ALDOA_HUMAN	Fructose-bisphosphate aldolase A	2582	39395	8.3	194	96	25	16	25	0.8	11.58
PLEC_HUMAN	Plectin	2184	531466	5.74	181	92	111	56	70	0.35	0.56
FINC_HUMAN	Fibronectin	1999	262460	5.46	154	93	60	40	60	0.45	0.96
CO6A3_HUMAN	Collagen alpha-3(VI) chain	1888	343457	6.26	100	73	55	40	55	0.31	0.67
IQGA1_HUMAN	Ras GTPase-activating-like protein IQGAP1	1827	189134	6.08	116	73	53	36	53	0.52	1.43
GRP78_HUMAN	78 kDa glucose-regulated protein	1815	72288	5.07	169	102	33	25	28	0.69	3.76
HS71A_HUMAN	Heat shock 70 kDa protein 1A	315	70009	5.48	37	17	20	11	16	0.56	0.93
DYHC1_HUMAN	Cytoplasmic dynein 1 heavy chain 1	1743	532072	6.01	135	80	84	53	84	0.3	0.52
EF2_HUMAN	Elongation factor 2	1628	95277	6.41	118	70	42	28	42	0.62	2.73
UBA1_HUMAN	Ubiquitin-like modifier-activating enzyme 1	1601	117774	5.49	96	65	33	26	10	0.61	2.01
GSTP1_HUMAN	Glutathione S-transferase P	1593	23341	5.43	74	57	15	13	15	0.92	16.03
PDIA1_HUMAN	Protein disulfide-isomerase	1552	57081	4.76	160	77	32	18	32	0.61	3.01
1433F_HUMAN	14-3-3 protein eta	214	28201	4.76	22	9	11	5	9	0.52	1.08
TBA1C_HUMAN	Tubulin alpha-1C chain	1127	49863	4.96	131	71	25	22	1	0.75	7.79
SERPH_HUMAN	Serpin H1	1461	46411	8.75	93	62	24	21	24	0.66	5.58
WDR1_HUMAN	WD repeat-containing protein 1	1454	66152	6.17	63	46	23	18	23	0.68	2.11
ENPL_HUMAN	Endoplasmin	1433	92411	4.76	126	68	42	28	12	0.55	2.88
TERA_HUMAN	Transitional endoplasmic reticulum ATPase	1363	89266	5.14	80	58	36	28	36	0.65	3.08
AHNK_HUMAN	Neuroblast differentiation-associated protein AHNAK	1329	628699	5.8	244	85	135	57	135	0.33	0.46
CO1A1_HUMAN	Collagen alpha-1(I) chain	1314	138857	5.6	165	78	40	30	12	0.53	1.55
GARS_HUMAN	Glycine--tRNA ligase	1311	83113	6.61	81	48	30	20	30	0.61	2.18
CKAP4_HUMAN	Cytoskeleton-associated protein 4	1302	65983	5.63	99	44	28	16	28	0.56	1.93
ANXA2_HUMAN	Annexin A2	1284	38580	7.57	167	86	29	20	29	0.73	8.58
MAP1B_HUMAN	Microtubule-associated protein 1B	1251	270468	4.73	87	57	51	37	51	0.36	0.8
VDAC1_HUMAN	Voltage-dependent anion-selective channel protein 1	1245	30754	8.62	47	34	16	12	16	0.76	4.77
LRP1_HUMAN	Prolow-density lipoprotein receptor-related protein 1	1218	504276	5.16	83	48	57	30	57	0.23	0.3
ANXA5_HUMAN	Annexin A5	1182	35914	4.94	103	54	22	14	12	0.74	6.99
PDIA3_HUMAN	Protein disulfide-isomerase A3	1177	56747	5.98	93	53	27	18	3	0.59	3.04
VINC_HUMAN	Vinculin	1153	123722	5.5	99	51	38	21	38	0.45	1.1
DPYL2_HUMAN	Dihydropyrimidinase-related protein 2	1146	62255	5.95	77	48	25	17	21	0.71	2.82
DPYL3_HUMAN	Dihydropyrimidinase-related protein 3	541	61924	6.04	39	21	21	12	17	0.69	1.4
CH60_HUMAN	60 kDa heat shock protein, mitochondrial	1100	61016	5.7	48	36	20	16	20	0.52	2.2
P4HA1_HUMAN	Prolyl 4-hydroxylase subunit alpha-1	1084	61011	5.7	51	32	19	14	19	0.52	1.6
PGK1_HUMAN	Phosphoglycerate kinase 1	1076	44586	8.3	75	40	21	13	5	0.68	3.45
HYOU1_HUMAN	Hypoxia up-regulated protein 1	1070	111266	5.16	52	35	20	13	20	0.36	0.69
MVP_HUMAN	Major vault protein	1051	99266	5.34	56	40	23	14	23	0.46	0.8
ATPA_HUMAN	ATP synthase subunit alpha, mitochondrial	1048	59714	9.16	55	37	21	13	21	0.54	1.85
TCPB_HUMAN	T-complex protein 1 subunit beta	1046	57452	6.01	48	34	22	17	22	0.62	2.43
CO1A2_HUMAN	Collagen alpha-2(I) chain	1038	129235	9.08	71	41	32	20	32	0.45	0.91
COCA1_HUMAN	Collagen alpha-1(XII) chain	1034	332941	5.38	73	40	47	23	47	0.26	0.34
RINI_HUMAN	Ribonuclease inhibitor	1004	49941	4.71	53	36	19	15	19	0.61	2.49
MOES_HUMAN	Moesin	967	67778	6.08	77	38	29	17	20	0.56	2.03
TSP1_HUMAN	Thrombospondin-1	958	129300	4.71	98	54	31	18	29	0.35	0.79
CAP1_HUMAN	Adenylyl cyclase-associated protein 1	831	51869	8.24	79	37	20	12	5	0.56	2.08
ACLY_HUMAN	ATP-citrate synthase	933	120762	6.95	68	39	34	22	34	0.49	1.22
MARCS_HUMAN	Myristoylated alanine-rich C-kinase substrate	928	31536	4.47	116	69	5	5	5	0.35	1.2
CO6A1_HUMAN	Collagen alpha-1(VI) chain	903	108462	5.26	45	32	21	16	21	0.37	0.85
LMO7_HUMAN	LIM domain only protein 7	894	192576	8.34	56	29	27	16	27	0.27	0.45
CAN2_HUMAN	Calpain-2 catalytic subunit	881	79945	4.87	58	34	26	18	20	0.54	1.7
LEG1_HUMAN	Galectin-1	877	14706	5.34	129	50	11	5	11	0.67	6.06
CALD1_HUMAN	Caldesmon	871	93175	5.62	90	39	22	13	22	0.3	0.79
FSCN1_HUMAN	Fascin	854	54496	6.84	44	28	17	13	17	0.56	1.92
COPG1_HUMAN	Coatomer subunit gamma-1	846	97655	5.32	42	23	25	13	23	0.5	0.74
COPG2_HUMAN	Coatomer subunit gamma-2	151	97560	5.56	10	5	7	3	5	0.12	0.14
STAT1_HUMAN	Signal transducer and activator of transcription 1-alpha/beta	845	87280	5.74	41	27	21	14	21	0.4	0.96
TRXR1_HUMAN	Thioredoxin reductase 1, cytoplasmic	842	70862	7.16	37	27	20	15	20	0.49	1.57
SYAC_HUMAN	Alanine--tRNA ligase, cytoplasmic	833	106743	5.34	48	35	26	20	26	0.47	1.19
COF1_HUMAN	Cofilin-1	832	18491	8.22	52	34	11	9	8	0.57	6.37
SERA_HUMAN	D-3-phosphoglycerate dehydrogenase	831	56614	6.29	40	34	19	16	3	0.58	2.77
GELS_HUMAN	Gelsolin	818	85644	5.9	57	39	19	14	19	0.52	1.18
PROF1_HUMAN	Profilin-1	808	15045	8.44	88	48	13	8	13	0.94	18.75
TAGL_HUMAN	Transgelin	804	22596	8.87	131	47	18	14	18	0.85	21.18
CATD_HUMAN	Cathepsin D	799	44524	6.1	57	35	18	12	18	0.6	3.07
TPM4_HUMAN	Tropomyosin alpha-4 chain	789	28504	4.67	87	44	18	10	10	0.54	3.27
TPM1_HUMAN	Tropomyosin alpha-1 chain	222	32689	4.69	85	22	21	11	12	0.46	3.05
TKT_HUMAN	Transketolase	783	67835	7.58	55	36	21	16	21	0.55	1.85
TCPH_HUMAN	T-complex protein 1 subunit eta	761	59329	7.55	44	29	20	16	20	0.58	2.08
GANAB_HUMAN	Neutral alpha-glucosidase AB	760	106807	5.74	63	36	32	19	32	0.6	1.28
SYWC_HUMAN	Tryptophan--tRNA ligase, cytoplasmic	760	53132	5.83	43	27	19	15	2	0.62	2.5
ANXA1_HUMAN	Annexin A1	759	38690	6.57	55	31	18	12	18	0.65	2.62
PEBP1_HUMAN	Phosphatidylethanolamine-binding protein 1	754	21044	7.01	24	17	7	6	7	0.62	2.23
AMPN_HUMAN	Aminopeptidase N	741	109471	5.31	66	29	34	18	34	0.49	1.07
ANXA6_HUMAN	Annexin A6	739	75826	5.42	89	38	40	20	40	0.66	2.01
SPB6_HUMAN	Serpin B6	737	42594	5.18	30	22	16	11	16	0.55	1.93
GDIB_HUMAN	Rab GDP dissociation inhibitor beta	733	50631	6.11	53	30	23	14	17	0.71	2.44
GDIA_HUMAN	Rab GDP dissociation inhibitor alpha	286	50550	5	31	22	15	14	9	0.58	2.17
PFKAP_HUMAN	ATP-dependent 6-phosphofructokinase, platelet type	733	85542	7.5	48	28	27	16	23	0.59	1.18
PFKAL_HUMAN	ATP-dependent 6-phosphofructokinase, liver type	264	84964	7.26	15	8	9	5	8	0.21	0.28
ECHA_HUMAN	Trifunctional enzyme subunit alpha, mitochondrial	733	82947	9.16	38	26	17	12	17	0.4	0.93
SND1_HUMAN	Staphylococcal nuclease domain-containing protein 1	730	101934	6.74	52	33	30	18	30	0.44	1.09
SEPT2_HUMAN	Septin-2	729	41461	6.15	36	29	14	12	13	0.57	2.68

[a]Protein score is calculated from the score of the peptide attributed to the protein.
[b]pI is (Predicted) isoelectric point.
[c]Number of matches is spectrum number matched to protein[#1].
[d]Number of significant matches is spectrum number that matches protein and exceeds the identification criteria.
[e]Number of sequences is number of peptides matched to protein[#2].
[f]Number of significant sequences is number of peptides exceeding the identification criteria matched to proteins.
[g]Number of unique sequences is a unique[#3] number of peptides matched to proteins.
[h]Sequence coverage is the ratio of the total number of matched peptide residues to the total length of the protein.
[i]Exponentially Modified Protein Abundance Index (http://www.matrixscience.com/help/quant_empai_help.html).

Table S2. Cont 1.

UniProt/SWISS-PROT ID	Description	Protein score[a]	Protein mass (kDa)	pI[b]	Num. of matches[c]	Num. of significant matches[d]	Num. of sequences[e]	Num. of significant sequences[f]	Num. of unique sequences[g]	Sequence coverage[h]	emPAI[i]
SYEP_HUMAN	Bifunctional glutamate/proline--tRNA ligase	725	170483	7.02	58	32	35	20	35	0.39	0.63
FAS_HUMAN	Fatty acid synthase	716	273254	6.01	67	40	42	25	42	0.31	0.47
TCPZ_HUMAN	T-complex protein 1 subunit zeta	712	57988	6.23	39	27	22	15	22	0.65	1.94
MYOF_HUMAN	Myoferlin	708	234561	5.84	78	39	51	26	51	0.37	0.68
TPIS_HUMAN	Triosephosphate isomerase	705	30772	5.65	64	39	16	12	11	0.7	4.77
CTNA1_HUMAN	Catenin alpha-1	691	100009	5.95	34	19	21	13	19	0.38	0.72
SEPR_HUMAN	Prolyl endopeptidase FAP	690	87657	6.21	46	32	26	19	26	0.46	1.47
CPNS1_HUMAN	Calpain small subunit 1	682	28298	5.05	27	20	14	11	4	0.8	4.79
ATPB_HUMAN	ATP synthase subunit beta, mitochondrial	681	56525	5.26	59	40	27	22	8	0.78	5.81
CAVN1_HUMAN	Caveolae-associated protein 1	670	43450	5.51	26	19	9	5	9	0.33	0.78
ML12B_HUMAN	Myosin regulatory light chain 12B	658	19767	4.71	38	27	13	11	1	0.84	11.19
ML12A_HUMAN	Myosin regulatory light chain 12A	640	19781	4.67	37	27	13	11	1	0.84	11.19
UGPA_HUMAN	UTP--glucose-1-phosphate uridylyltransferase	650	56905	8.16	44	28	18	15	4	0.6	2
PLIN3_HUMAN	Perilipin-3	550	47046	5,3	32	23	16	11	3	0,59	1,65
PTBP1_HUMAN	Polypyrimidine tract-binding protein 1	652	57186	9,22	25	19	9	7	9	0,32	0,67
PSMD1_HUMAN	26S proteasome non-ATPase regulatory subunit 1	651	105769	5,25	33	24	18	14	18	0,34	0,74
MAP4_HUMAN	Microtubule-associated protein 4	651	120930	5,32	66	22	22	10	22	0,36	0,46
THIO_HUMAN	Thioredoxin	649	11730	4,82	39	27	8	5	8	0,78	4,63
CNN3_HUMAN	Calponin-3	640	36391	5,69	50	30	14	8	13	0,51	1,79
CNN2_HUMAN	Calponin-2	480	33675	6,95	44	27	11	9	10	0,6	3,4
IMB1_HUMAN	Importin subunit beta-1	631	97108	4,68	46	28	25	16	25	0,52	1,08
IPO5_HUMAN	Importin-5	616	123550	4,83	60	36	36	26	36	0,55	1,41
TENA_HUMAN	Tenascin	611	240700	4,79	45	30	29	18	29	0,29	0,37
TAGL2_HUMAN	Transgelin-2	597	22377	8,41	65	30	15	10	15	0,84	9,94
PRDX6_HUMAN	Peroxiredoxin-6	589	25019	6	38	20	14	8	14	0,76	2,76
COPB_HUMAN	Coatomer subunit beta	583	107074	5,72	33	20	22	14	22	0,38	0,73
SYIC_HUMAN	Isoleucine--tRNA ligase, cytoplasmic	579	144406	5,82	53	31	29	17	28	0,37	0,64
SPTN1_HUMAN	Spectrin alpha chain, non-erythrocytic 1	578	284364	5,22	50	17	39	12	39	0,25	0,19
COPA_HUMAN	Coatomer subunit alpha	571	138258	7,7	40	26	21	15	21	0,26	0,57
PDIA6_HUMAN	Protein disulfide-isomerase A6	570	48091	4,95	30	21	11	9	11	0,48	1,59
GBB4_HUMAN	Guanine nucleotide-binding protein subunit beta-4	120	37543	5,6	11	5	7	4	3	0,39	0,56
P4HA2_HUMAN	Prolyl 4-hydroxylase subunit alpha-2	560	60864	5,49	44	29	20	12	20	0,47	1,28
SC31A_HUMAN	Protein transport protein Sec31A	555	132931	6,43	35	19	19	10	19	0,31	0,37
UGGG1_HUMAN	UDP-glucose:glycoprotein glucosyltransferase 1	550	177078	5,42	41	27	25	17	25	0,32	0,5
PPIB_HUMAN	Peptidyl-prolyl cis-trans isomerase B	545	23728	9,42	60	25	17	6	17	0,64	3,02
PGAM1_HUMAN	Phosphoglycerate mutase 1	535	28786	6,67	47	28	11	8	11	0,65	3,22
CLIC4_HUMAN	Chloride intracellular channel protein 4	531	28754	5,45	43	25	14	8	13	0,72	2,66
COPB2_HUMAN	Coatomer subunit beta'	528	102422	5,15	42	26	24	17	24	0,46	1,08
FKB10_HUMAN	Peptidyl-prolyl cis-trans isomerase FKBP10	524	64204	5,36	39	23	20	11	20	0,43	1,05
EF1G_HUMAN	Elongation factor 1-gamma	523	50087	6,25	41	24	19	12	19	0,55	1,95
TCPE_HUMAN	T-complex protein 1 subunit epsilon	520	59633	5,45	34	25	16	12	16	0,54	1,66
VAT1_HUMAN	Synaptic vesicle membrane protein VAT-1 homolog	510	41893	5,88	36	24	16	15	16	0,59	3,44
VIGLN_HUMAN	Vigilin	500	141368	6,43	44	20	25	13	3	0,32	0,47
TCPA_HUMAN	T-complex protein 1 subunit alpha	503	60306	5,8	31	18	19	12	7	0,58	1,29
GFPT1_HUMAN	Glutamine--fructose-6-phosphate aminotransferase [isomerizing] 1	501	78756	6,66	29	18	22	13	18	0,51	0,99
GFPT2_HUMAN	Glutamine--fructose-6-phosphate aminotransferase [isomerizing] 2	167	76882	7,03	12	5	9	3	5	0,21	0,18
RRBP1_HUMAN	Ribosome-binding protein 1	492	152381	8,69	52	30	24	12	24	0,24	0,39
HSPB1_HUMAN	Heat shock protein beta-1	489	22768	5,98	54	38	13	8	13	0,86	9,57
2AAA_HUMAN	Serine/threonine-protein phosphatase 2A 65 kDa regulatory subunit A alpha isoform	487	65267	5	23	14	15	10	15	0,42	0,9
MDHM_HUMAN	Malate dehydrogenase, mitochondrial	486	35481	8,92	31	18	12	7	12	0,48	1,27
VATA_HUMAN	V-type proton ATPase catalytic subunit A	485	68260	5,35	35	26	18	12	18	0,46	1,08
G6PD_HUMAN	Glucose-6-phosphate 1-dehydrogenase	484	59219	6,39	38	25	19	12	19	0,57	1,68
PRDX5_HUMAN	Peroxiredoxin-5, mitochondrial	483	22073	8,93	26	16	10	6	10	0,66	2,07
RPN2_HUMAN	Dolichyl-diphosphooligosaccharide--protein glycosyltransferase subunit 2	483	69241	5,44	30	22	17	14	17	0,55	1,33
PSMD2_HUMAN	26S proteasome non-ATPase regulatory subunit 2	475	100136	5,08	37	22	19	13	19	0,41	0,72
SYCC_HUMAN	Cysteine--tRNA ligase, cytoplasmic	475	85419	6,31	23	13	15	7	15	0,34	0,41
S10AB_HUMAN	Protein S100-A11	473	11733	6,56	42	17	6	5	6	0,74	21,41
PARK7_HUMAN	Protein/nucleic acid deglycase DJ-1	468	19878	6,33	32	19	9	6	9	0,72	3,27
PLOD3_HUMAN	Procollagen-lysine,2-oxoglutarate 5-dioxygenase 3	467	84731	5,69	28	21	11	9	11	0,26	0,72
PLOD2_HUMAN	Procollagen-lysine,2-oxoglutarate 5-dioxygenase 2	308	84632	6,24	31	13	19	9	19	0,36	0,56
PLOD1_HUMAN	Procollagen-lysine,2-oxoglutarate 5-dioxygenase 1	307	83497	6,47	26	17	17	9	17	0,35	0,57
HSP74_HUMAN	Heat shock 70 kDa protein 4	466	94271	5,11	37	24	23	15	22	0,43	0,95
HS105_HUMAN	Heat shock protein 105 kDa	89	96804	5,28	18	4	14	3	13	0,25	0,14
UGDH_HUMAN	UDP-glucose 6-dehydrogenase	463	54989	6,73	35	26	19	16	19	0,68	2,37
NEP_HUMAN	Neprilysin	460	85460	5,54	35	18	21	11	21	0,4	0,71
G6PI_HUMAN	Glucose-6-phosphate isomerase	447	63107	8,43	30	16	14	9	10	0,44	0,94
STIP1_HUMAN	Stress-induced-phosphoprotein 1	446	62599	6,4	22	14	12	7	12	0,33	0,59
NNMT_HUMAN	Nicotinamide N-methyltransferase	439	29555	5,56	36	17	11	6	11	0,66	1,32
ADHX_HUMAN	Alcohol dehydrogenase class-3	438	39698	7,45	18	13	8	5	8	0,39	0,69
BGH3_HUMAN	Transforming growth factor-beta-induced protein ig-h3	437	74634	7,62	26	13	15	7	15	0,37	0,48
CSRP1_HUMAN	Cysteine and glycine-rich protein 1	430	20554	8,9	30	19	9	7	9	0,59	3,07
CALU_HUMAN	Calumenin	428	37084	4,47	50	18	18	9	18	0,52	1,74
HNRPU_HUMAN	Heterogeneous nuclear ribonucleoprotein U	428	90528	5,76	29	19	16	10	16	0,34	0,66
PSB5_HUMAN	Proteasome subunit beta type-5	423	28462	6,43	15	12	6	5	6	0,38	1,07
TCPG_HUMAN	T-complex protein 1 subunit gamma	422	60495	6,1	28	13	15	8	15	0,42	0,74
GRHPR_HUMAN	Glyoxylate reductase/hydroxypyruvate reductase	420	35646	7,01	9	7	4	3	4	0,28	0,42
4F2_HUMAN	4F2 cell-surface antigen heavy chain	417	67952	4,89	25	16	15	10	15	0,37	0,85
OST48_HUMAN	Dolichyl-diphosphooligosaccharide--protein glycosyltransferase 48 kDa subunit	416	50769	6,09	19	13	9	6	9	0,4	0,78
DREB_HUMAN	Drebrin	415	71385	4,41	19	15	9	6	9	0,29	0,51
AP1B1_HUMAN	AP-1 complex subunit beta-1	230	104570	4,94	22	8	16	7	11	0,28	0,32
PDC6I_HUMAN	Programmed cell death 6-interacting protein	405	95963	6,13	32	18	19	10	8	0,38	0,69
P3H1_HUMAN	Prolyl 3-hydroxylase 1	405	83341	5,05	34	17	18	9	18	0,45	0,65
TMEDA_HUMAN	Transmembrane emp24 domain-containing protein 10	404	24960	6,97	14	9	6	4	6	0,38	0,94
SYSC_HUMAN	Serine--tRNA ligase, cytoplasmic	403	58740	6,05	27	16	16	7	16	0,42	0,64
CALX_HUMAN	Calnexin	403	67526	4,47	33	19	13	8	13	0,33	0,97
CO5A1_HUMAN	Collagen alpha-1(V) chain	403	183447	4,94	18	13	12	8	12	0,14	0,2
NIBL1_HUMAN	Niban-like protein 1	402	84085	5,82	34	22	15	13	15	0,32	0,91
ATLA3_HUMAN	Atlastin-3	401	60503	5,43	32	19	15	10	15	0,51	1,14
GCN1_HUMAN	eIF-2-alpha kinase activator GCN1	399	292572	7,29	40	22	22	14	22	0,15	0,22
CLIC1_HUMAN	Chloride intracellular channel protein 1	399	26906	5,09	44	22	14	9	13	0,69	3
TX1B3_HUMAN	Tax1-binding protein 3	398	13726	8,04	13	11	5	4	5	0,65	2,28
PLST_HUMAN	Plastin-3	395	70766	5,41	45	24	29	20	5	0,63	2,25
FERM2_HUMAN	Fermitin family homolog 2	393	77811	6,26	26	19	15	13	15	0,39	1,01
NB5R3_HUMAN	NADH-cytochrome b5 reductase 3	389	34213	7,18	24	14	13	8	13	0,62	1,64
IDHC_HUMAN	Isocitrate dehydrogenase [NADP] cytoplasmic	387	46630	6,53	34	21	16	9	15	0,56	1,44
ARF4_HUMAN	ADP-ribosylation factor 4	384	20498	6,59	33	15	13	7	9	0,83	3,07
PPCE_HUMAN	Prolyl endopeptidase	380	80648	5,53	18	11	12	7	12	0,33	0,44
TCPD_HUMAN	T-complex protein 1 subunit delta	377	57888	7,96	32	20	19	13	19	0,58	1,55
ITAV_HUMAN	Integrin alpha-V	375	115964	5,45	29	20	18	12	18	0,3	0,54
SPTB2_HUMAN	Spectrin beta chain, non-erythrocytic 1	372	274439	5,39	34	13	22	9	22	0,17	0,17

[a]Protein score is calculated from the score of the peptide attributed to the protein.
[b]pI is (Predicted) isoelectric point.
[c]Number of matches is spectrum number matched to protein[#1].
[d]Number of significant matches is spectrum number that matches protein and exceeds the identification criteria.
[e]Number of sequences is number of peptides matched to protein[#2].
[f]Number of significant sequences is number of peptides exceeding the identification criteria matched to proteins.
[g]Number of unique sequences is a unique[#3] number of peptides matched to proteins.
[h]Sequence coverage is the ratio of the total number of matched peptide residues to the total length of the protein.
[i]Exponentially Modified Protein Abundance Index (http://www.matrixscience.com/help/quant_empai_help.html).

Table S2. Cont 2.

UniProt/SWISS-PROT ID	Description	Protein score[a]	Protein mass (kDa)	pI[b]	Num. of matches[c]	Num. of significant matches[d]	Num. of sequences[e]	Num. of significant sequences[f]	Num. of unique sequences[g]	Sequence coverage[h]	emPAI[i]
6PGD_HUMAN	6−phosphogluconate dehydrogenase, decarboxylating	371	53106	6,8	20	16	11	8	11	0,38	0,87
AP2A1_HUMAN	AP−2 complex subunit alpha−1	370	107478	6,63	37	19	23	12	18	0,38	0,6
AP2A2_HUMAN	AP−2 complex subunit alpha−2	207	103895	6,53	21	8	13	5	8	0,24	0,22
OAT_HUMAN	Ornithine aminotransferase, mitochondrial	370	48504	6,57	16	12	8	7	8	0,32	0,82
MMP14_HUMAN	Matrix metalloproteinase−14	367	65852	7,63	36	12	10	5	10	0,2	0,37
HNRPF_HUMAN	Heterogeneous nuclear ribonucleoprotein F	366	45643	5,38	16	12	6	5	4	0,3	0,73
HNRH1_HUMAN	Heterogeneous nuclear ribonucleoprotein H	209	49198	5,89	18	9	10	7	6	0,38	0,81
HNRH2_HUMAN	Heterogeneous nuclear ribonucleoprotein H2	116	49232	5,89	11	6	9	6	6	0,38	0,66
AATC_HUMAN	Aspartate aminotransferase, cytoplasmic	366	46219	6,52	22	12	12	7	12	0,53	0,88
DPP4_HUMAN	Dipeptidyl peptidase 4	365	88222	5,67	19	13	9	5	9	0,19	0,27
LAMC1_HUMAN	Laminin subunit gamma−1	363	177489	5,01	22	18	15	11	15	0,18	0,3
PICAL_HUMAN	Phosphatidylinositol−binding clathrin assembly protein	359	70710	7,7	16	11	8	6	8	0,24	0,51
SERC_HUMAN	Phosphoserine aminotransferas	357	40397	7,56	37	18	17	10	17	0,67	1,8
MDHC_HUMAN	Malate dehydrogenase, cytoplasmic	354	36403	6,91	21	13	9	5	9	0,55	0,77
LRC59_HUMAN	Leucine−rich repeat−containing protein 59	353	34909	9,61	23	15	11	6	11	0,39	1,04
ERP29_HUMAN	Endoplasmic reticulum resident protein 29	350	28975	6,77	18	12	8	7	8	0,45	1,72
COPE_HUMAN	Coatomer subunit epsilon	349	34460	4,97	18	15	8	7	8	0,44	1,33
RL5_HUMAN	60S ribosomal protein L5	343	34341	9,73	19	10	8	4	8	0,4	0,62
VATB2_HUMAN	V−type proton ATPase subunit B, brain isoform	342	56465	5,57	25	16	11	9	11	0,41	0,94
RCN3_HUMAN	Reticulocalbin−3	339	37470	4,74	16	10	6	5	2	0,35	0,94
FKBP9_HUMAN	Peptidyl−prolyl cis−trans isomerase FKBP9	339	63044	4,91	20	15	8	6	8	0,22	0,59
YBOX3_HUMAN	Y−box−binding protein 3	278	40066	9,77	11	6	5	2	3	0,23	0,23
DDX1_HUMAN	ATP−dependent RNA helicase DDX1	335	82380	6,81	23	13	14	7	14	0,32	0,43
AGM1_HUMAN	Phosphoacetylglucosamine mutase	335	59814	5,84	19	12	13	8	13	0,4	0,75
LPPRC_HUMAN	Leucine−rich PPR motif−containing protein, mitochondrial	333	157805	5,81	25	14	16	8	16	0,22	0,24
PRDX4_HUMAN	Peroxiredoxin−4	327	30521	5,86	38	22	15	10	13	0,7	2,89
PRDX1_HUMAN	Peroxiredoxin−1	229	22096	8,27	53	22	20	11	17	0,88	6,8
PRDX2_HUMAN	Peroxiredoxin−2	165	21878	5,66	16	8	7	5	6	0,44	1,57
ESTD_HUMAN	S−formylglutathione hydrolase	324	31442	6,54	16	11	9	6	9	0,57	1,21
PALLD_HUMAN	Palladin	322	150470	6,67	36	17	20	9	20	0,24	0,28
PYGB_HUMAN	Glycogen phosphorylase, brain form	322	96635	6,4	28	14	19	10	15	0,33	0,54
PYGL_HUMAN	Glycogen phosphorylase, liver form	68	97087	6,71	10	3	7	3	3	0,14	0,14
IF2A_HUMAN	Eukaryotic translation initiation factor 2 subunit 1	321	36089	5,02	20	15	10	7	10	0,47	1,24
GSLG1_HUMAN	Golgi apparatus protein 1	321	134464	6,52	38	12	18	6	18	0,26	0,21
DDX3X_HUMAN	ATP−dependent RNA helicase DDX3X	319	73198	6,73	15	11	10	7	10	0,25	0,49
IPO7_HUMAN	Importin−7	318	119440	4,7	26	14	13	8	13	0,23	0,32
NUCL_HUMAN	Nucleolin	318	76568	4,6	23	13	11	6	11	0,21	0,39
ITB1_HUMAN	Integrin beta−1	316	88357	5,27	41	19	21	10	18	0,45	0,68
SYVC_HUMAN	Valine−−tRNA ligase	316	140387	7,53	19	12	14	9	14	0,2	0,31
CATB_HUMAN	Cathepsin B	314	37797	5,88	33	12	12	7	12	0,42	1,41
RTN4_HUMAN	Reticulon−4	312	129851	4,43	19	10	7	4	7	0,14	0,17
SYRC_HUMAN	Arginine−−tRNA ligase, cytoplasmic	305	75331	6,26	24	11	17	8	17	0,35	0,56
SAP_HUMAN	Prosaposin	304	58074	5,06	46	17	17	7	17	0,5	0,91
FHL2_HUMAN	Four and a half LIM domains protein 2	304	32171	7,8	20	9	12	5	12	0,6	0,9
FBLN1_HUMAN	Fibulin−1	267	77162	5,07	23	6	12	3	7	0,19	0,24
DCTN2_HUMAN	Dynactin subunit 2	304	44204	5,1	9	8	4	4	4	0,15	0,46
ANXA4_HUMAN	Annexin A4	299	35860	5,84	30	11	13	5	7	0,56	1
COMT_HUMAN	Catechol O−methyltransferase	302	30018	5,26	20	13	10	6	10	0,53	1,29
NOMO1_HUMAN	Nodal modulator 1	302	134239	5,54	20	11	16	8	16	0,27	0,28
ECHM_HUMAN	Enoyl−CoA hydratase, mitochondrial	302	31367	8,34	11	9	7	6	1	0,42	1,21
EHD2_HUMAN	EH domain−containing protein 2	301	61123	6,03	22	14	14	10	14	0,38	0,98
IPYR_HUMAN	Inorganic pyrophosphatase	301	32639	5,54	21	11	11	6	11	0,55	1,14
MYO1C_HUMAN	Unconventional myosin−Ic	300	121606	9,46	29	12	20	9	20	0,3	0,36
TCPQ_HUMAN	T−complex protein 1 subunit theta	299	59583	5,42	38	17	20	10	20	0,46	1,01
IMA7_HUMAN	Importin subunit alpha−7	298	59991	4,89	13	11	7	5	6	0,28	0,42
PUR9_HUMAN	Bifunctional purine biosynthesis protein PURH	297	64575	6,27	25	15	14	10	14	0,45	0,91
FHL1_HUMAN	Four and a half LIM domains protein 1	295	36239	9,25	17	12	7	6	7	0,32	0,99
RL9_HUMAN	60S ribosomal protein L9	294	21850	9,96	26	17	8	6	8	0,62	2,75
LIMA1_HUMAN	LIM domain and actin−binding protein 1	293	85173	6,41	19	9	11	5	11	0,19	0,28
AMPL_HUMAN	Cytosol aminopeptidase	293	56131	8,03	19	8	11	5	11	0,33	0,45
AL7A1_HUMAN	Alpha−aminoadipic semialdehyde dehydrogenase	292	58450	8,21	15	10	10	7	10	0,35	0,65
TXND5_HUMAN	Thioredoxin domain−containing protein 5	292	47599	5,63	40	17	13	9	13	0,43	1,2
THIC_HUMAN	Acetyl−CoA acetyltransferase, cytosolic	292	41324	6,47	13	10	8	8	8	0,51	1,24
STRAP_HUMAN	Serine−threonine kinase receptor−associated protein	289	38414	4,98	17	11	10	5	10	0,53	0,72
PSB4_HUMAN	Proteasome subunit beta type−4	288	29185	5,72	11	7	7	6	7	0,47	1,34
DHX9_HUMAN	ATP−dependent RNA helicase A	283	140869	6,41	17	10	8	3	8	0,14	0,09
AL1L2_HUMAN	Mitochondrial 10−formyltetrahydrofolate dehydrogenase	281	101681	6,13	33	18	21	12	21	0,37	0,64
GLSK_HUMAN	Glutaminase kidney isoform, mitochondrial	280	73414	7,85	29	10	14	5	14	0,32	0,33
APT_HUMAN	Adenine phosphoribosyltransferase	279	19595	5,78	7	5	6	4	6	0,53	1,31
SDCB1_HUMAN	Syntenin−1	279	32424	7,05	16	13	5	5	5	0,39	1,16
SCRN1_HUMAN	Secernin−1	274	46353	4,66	19	11	8	4	8	0,37	0,43
USO1_HUMAN	General vesicular transport factor p115	274	107828	4,85	28	19	13	10	13	0,23	0,47
ECHB_HUMAN	Trifunctional enzyme subunit beta, mitochondrial	273	51262	9,45	14	10	6	4	6	0,23	0,38
ESYT1_HUMAN	Extended synaptotagmin−1	272	122780	5,57	23	14	10	6	10	0,18	0,23
ASNS_HUMAN	Asparagine synthetase [glutamine−hydrolyzing]	271	64329	6,39	28	12	17	8	17	0,51	0,68
RPN1_HUMAN	Dolichyl−diphosphooligosaccharide−−protein glycosyltransferase subunit 1	270	68527	5,96	46	25	22	16	21	0,5	1,65
RD23B_HUMAN	UV excision repair protein RAD23 homolog B	270	43145	4,79	12	11	6	5	6	0,31	0,78
CO6A2_HUMAN	Collagen alpha−2(VI) chain	269	108512	5,85	22	15	13	9	13	0,25	0,47
CNDP2_HUMAN	Cytosolic non−specific dipeptidase	268	52845	5,66	16	10	11	6	11	0,43	0,61
MRC2_HUMAN	C−type mannose receptor 2	267	166568	5,54	26	15	15	9	15	0,23	0,25
XRCC5_HUMAN	X−ray repair cross−complementing protein 5	266	82652	5,55	27	16	18	11	18	0,45	0,74
RAB23_HUMAN	Ras−related protein Rab−23	266	26643	6,22	9	7	4	3	4	0,36	0,59
NXP20_HUMAN	Protein NOXP20	266	60704	4,61	12	9	9	6	9	0,26	0,51
IF5A2_HUMAN	Eukaryotic translation initiation factor 5A−2	119	16782	5,38	10	9	6	6	1	0,42	3,35
TMX3_HUMAN	Protein disulfide−isomerase TMX3	265	51839	4,8	11	6	8	4	8	0,25	0,38
EF1B_HUMAN	Elongation factor 1−beta	263	24748	4,5	24	13	9	8	8	0,59	3,5
EF1D_HUMAN	Elongation factor 1−delta	151	31103	4,9	19	9	10	7	9	0,52	1,54
BASP1_HUMAN	Brain acid soluble protein 1	262	22680	4,64	41	15	7	5	7	0,54	1,99
PSB6_HUMAN	Proteasome subunit beta type−6	261	25341	4,8	7	6	3	3	3	0,33	0,63
PSME2_HUMAN	Proteasome activator complex subunit 2	261	27384	5,54	7	5	5	3	5	0,33	0,57
CAN1_HUMAN	Calpain−1 catalytic subunit	260	81838	5,49	21	14	16	10	16	0,42	0,67
NDKB_HUMAN	Nucleoside diphosphate kinase B	259	17287	8,52	34	10	9	4	5	0,72	3,15
NDKA_HUMAN	Nucleoside diphosphate kinase A	90	17138	5,83	31	9	11	5	7	0,88	3,23
PSMD3_HUMAN	26S proteasome non−ATPase regulatory subunit 3	259	60939	8,47	19	14	10	7	10	0,23	0,61
ARC1B_HUMAN	Actin−related protein 2/3 complex subunit 1B	258	40923	8,69	25	13	10	6	9	0,41	1,04
ANX11_HUMAN	Annexin A11	255	54355	7,53	12	9	6	3	6	0,17	0,26
ITA5_HUMAN	Integrin alpha−5	255	114465	5,5	15	10	9	7	9	0,2	0,29
CBR3_HUMAN	Carbonyl reductase [NADPH] 3	135	30831	5,82	11	7	5	3	2	0,27	0,5
ITB5_HUMAN	Integrin beta−5	248	87996	5,72	13	11	10	8	10	0,29	0,46
SCRB2_HUMAN	Lysosome membrane protein 2	245	54255	5	21	11	10	4	10	0,35	0,36

[a]Protein score is calculated from the score of the peptide attributed to the protein.
[b]pI is (Predicted) isoelectric point.
[c]Number of matches is spectrum number matched to protein[#1].
[d]Number of significant matches is spectrum number that matches protein and exceeds the identification criteria.
[e]Number of sequences is number of peptides matched to protein[#2].
[f]Number of significant sequences is number of peptides exceeding the identification criteria matched to proteins.
[g]Number of unique sequences is a unique[#3] number of peptides matched to proteins.
[h]Sequence coverage is the ratio of the total number of matched peptide residues to the total length of the protein.
[i]Exponentially Modified Protein Abundance Index (http://www.matrixscience.com/help/quant_empai_help.html).

Table S2. Cont 3.

UniProt/SWISS-PROT ID	Description	Protein score[a]	Protein mass (kDa)	pI[b]	Num. of matches[c]	Num. of significant matches[d]	Num. of sequences[e]	Num. of significant sequences[f]	Num. of unique sequences[g]	Sequence coverage[h]	emPAI[i]
PPGB_HUMAN	Lysosomal protective protein	244	54431	6,16	8	6	5	3	5	0,17	0,26
C1QBP_HUMAN	Complement component 1 Q subcomponent-binding protein, mitochondrial	243	31343	4,74	15	9	7	3	7	0,47	0,7
HINT1_HUMAN	Histidine triad nucleotide-binding protein 1	242	13793	6,43	13	9	4	3	4	0,47	2,28
EHD1_HUMAN	EH domain-containing protein 1	242	60589	6,35	15	9	10	5	10	0,33	0,51
PGM1_HUMAN	Phosphoglucomutase-1	241	61411	6,3	17	11	13	9	13	0,37	0,84
SRC8_HUMAN	Src substrate cortactin	240	61549	5,24	13	8	9	5	9	0,22	0,4
PAI1_HUMAN	Plasminogen activator inhibitor 1	240	45031	6,68	12	8	8	6	8	0,36	0,91
NP1L1_HUMAN	Nucleosome assembly protein 1-like 1	240	45346	4,36	18	11	8	6	7	0,43	0,73
CAPG_HUMAN	Macrophage-capping protein	239	38474	5,82	23	14	9	6	9	0,45	1,13
ARPC5_HUMAN	Actin-related protein 2/3 complex subunit 5	238	16310	5,47	10	5	5	2	5	0,56	0,65
NACAM_HUMAN	Nascent polypeptide-associated complex subunit alpha, muscle-specific form	238	205295	9,6	13	8	7	5	7	0,04	0,11
NACA2_HUMAN	Nascent polypeptide-associated complex subunit alpha-2	109	23209	4,68	3	3	1	1	1	0,07	0,19
LKHA4_HUMAN	Leukotriene A-4 hydrolase	237	69241	5,8	16	11	6	6	6	0,16	0,44
BZW1_HUMAN	Basic leucine zipper and W2 domain-containing protein 1	236	48013	5,75	13	8	9	5	8	0,35	0,54
PA2G4_HUMAN	Proliferation-associated protein 2G4	236	43759	6,13	11	5	8	3	8	0,32	0,33
DHB12_HUMAN	Very-long-chain 3-oxoacyl-CoA reductase	233	34302	9,34	12	7	6	4	6	0,38	0,62
GSHB_HUMAN	Glutathione synthetase	232	52352	5,67	10	8	5	4	5	0,2	0,37
IMDH2_HUMAN	Inosine-5'-monophosphate dehydrogenase 2	231	55770	6,44	12	6	5	3	5	0,21	0,25
ASSY_HUMAN	Argininosuccinate synthase	230	46501	8,08	15	12	8	7	8	0,4	1,05
MK01_HUMAN	Mitogen-activated protein kinase 1	230	41363	6,5	14	9	10	7	6	0,51	1,02
MK03_HUMAN	Mitogen-activated protein kinase 3	91	43108	6,28	10	2	7	2	3	0,3	0,21
SEC13_HUMAN	Protein SEC13 homolog	230	35518	5,22	20	10	6	4	6	0,23	0,79
RHG01_HUMAN	Rho GTPase-activating protein 1	229	50404	5,85	32	11	14	6	14	0,44	0,64
UCHL1_HUMAN	Ubiquitin carboxyl-terminal hydrolase isozyme L1	229	24808	5,33	43	17	15	11	15	0,85	5,28
RTRAF_HUMAN	RNA transcription, translation and transport factor protein	222	28051	6,19	12	6	7	5	7	0,43	1,09
GDS1_HUMAN	Rap1 GTPase-GDP dissociation stimulator 1	222	66275	5,17	19	9	11	7	11	0,4	0,55
CCD47_HUMAN	Coiled-coil domain-containing protein 47	220	55838	4,76	13	8	5	2	5	0,2	0,16
AMPB_HUMAN	Aminopeptidase B	219	72549	5,51	15	9	9	5	9	0,29	0,41
EIF3A_HUMAN	Eukaryotic translation initiation factor 3 subunit A	218	166468	6,38	21	11	12	5	12	0,14	0,13
RL3_HUMAN	60S ribosomal protein L3	218	46080	10,19	18	9	9	4	9	0,32	0,72
GNAI2_HUMAN	Guanine nucleotide-binding protein G(i) subunit alpha-2	217	40425	5,34	28	10	12	6	5	0,44	0,85
GNAS1_HUMAN	Guanine nucleotide-binding protein G(s) subunit alpha isoforms Xlas	123	110956	4,91	9	5	4	4	3	0,06	0,16
SPD2A_HUMAN	SH3 and PX domain-containing protein 2A	216	125213	9,01	11	7	5	3	5	0,08	0,11
GLYM_HUMAN	Serine hydroxymethyltransferase, mitochondrial	215	55958	8,76	12	10	7	5	7	0,21	0,45
TPD54_HUMAN	Tumor protein D54	214	22224	5,26	22	10	8	5	8	0,56	2,05
LMAN1_HUMAN	Protein ERGIC-53	213	57513	6,3	11	6	5	4	5	0,15	0,34
EGLN_HUMAN	Endoglin	213	70533	6,14	21	11	11	9	11	0,3	0,7
THIK_HUMAN	3-ketoacyl-CoA thiolase, peroxisomal	212	44264	8,76	11	9	6	4	6	0,33	0,46
CIRBP_HUMAN	Cold-inducible RNA-binding protein	212	18637	9,51	5	4	2	1	2	0,19	0,25
KINH_HUMAN	Kinesin-1 heavy chain	212	109617	6,12	24	12	19	10	19	0,33	0,46
PDXK_HUMAN	Pyridoxal kinase	211	35080	5,75	13	8	7	4	7	0,38	0,61
TCTP_HUMAN	Translationally-controlled tumor protein	211	19583	4,84	16	11	5	5	5	0,43	2,54
ALDR_HUMAN	Aldose reductase	210	35830	6,51	17	8	10	5	10	0,54	0,78
CAZA1_HUMAN	F-actin-capping protein subunit alpha-1	143	32902	5,45	18	8	9	5	7	0,55	0,88
EIF3F_HUMAN	Eukaryotic translation initiation factor 3 subunit F	208	37540	5,24	8	5	6	4	6	0,28	0,56
CNN1_HUMAN	Calponin-1	208	33150	9,14	12	9	5	3	5	0,35	0,46
DCTN1_HUMAN	Dynactin subunit 1	207	141607	5,61	13	8	8	4	8	0,13	0,13
SAHH_HUMAN	Adenosylhomocysteinase	207	47685	5,92	17	7	10	5	10	0,29	0,55
RCN1_HUMAN	Reticulocalbin-1	207	38866	4,86	17	8	9	5	9	0,37	0,9
5NTD_HUMAN	5'-nucleotidase	207	63327	6,58	13	7	9	4	9	0,33	0,3
PAPS2_HUMAN	Bifunctional 3'-phosphoadenosine 5'-phosphosulfate synthase 2	206	69457	8,18	19	10	9	7	9	0,28	0,52
HTRA1_HUMAN	Serine protease HTRA1	206	51255	8,09	5	5	3	3	3	0,19	0,28
PNPH_HUMAN	Purine nucleoside phosphorylase	205	32097	6,45	13	10	9	6	9	0,45	1,17
CD81_HUMAN	CD81 antigen	205	25792	5,09	8	6	3	3	3	0,25	0,62
PHB2_HUMAN	Prohibitin-2	203	33276	9,83	12	9	8	5	8	0,37	0,86
RLA0_HUMAN	60S acidic ribosomal protein P0	201	34252	5,71	30	11	11	8	11	0,56	1,97
TXD17_HUMAN	Thioredoxin domain-containing protein 17	201	13932	5,4	11	9	6	5	6	0,7	3,31
GSTO1_HUMAN	Glutathione S-transferase omega-1	201	27548	6,23	24	13	12	6	12	0,63	1,47
PDLI1_HUMAN	PDZ and LIM domain protein 1	200	36049	6,56	15	9	8	5	8	0,43	1
ZYX_HUMAN	Zyxin	199	61238	6,22	34	11	12	6	12	0,4	0,72
SRPRA_HUMAN	Signal recognition particle receptor subunit alpha	199	69767	9,07	11	5	8	4	8	0,22	0,27
FKB1A_HUMAN	Peptidyl-prolyl cis-trans isomerase FKBP1A	197	11943	7,88	11	9	4	4	4	0,49	2,89
MAP1A_HUMAN	Microtubule-associated protein 1A	195	305298	4,85	18	8	14	5	14	0,1	0,07
HNRPM_HUMAN	Heterogeneous nuclear ribonucleoprotein M	195	77464	8,84	16	7	12	5	12	0,26	0,31
PDIA4_HUMAN	Protein disulfide-isomerase A4	194	72887	4,96	18	8	13	6	13	0,27	0,49
QORX_HUMAN	Quinone oxidoreductase PIG3	194	35514	6,67	8	6	5	4	5	0,24	0,6
OLA1_HUMAN	Obg-like ATPase 1	193	44715	7,64	9	6	7	4	7	0,26	0,45
LMAN2_HUMAN	Vesicular integral-membrane protein VIP36	192	40203	6,46	12	5	7	3	7	0,31	0,36
PEPD_HUMAN	Xaa-Pro dipeptidase	191	54513	5,64	9	5	7	5	7	0,28	0,47
CTHR1_HUMAN	Collagen triple helix repeat-containing protein 1	191	26207	8,31	16	8	7	4	7	0,46	0,88
EIF3I_HUMAN	Eukaryotic translation initiation factor 3 subunit I	191	36479	5,38	17	11	7	6	7	0,44	0,98
PCYOX_HUMAN	Prenylcysteine oxidase 1	190	56604	5,8	8	7	6	5	6	0,24	0,45
PSB7_HUMAN	Proteasome subunit beta type-7	190	29946	7,57	6	4	3	2	3	0,21	0,32
LPP_HUMAN	Lipoma-preferred partner	190	65704	7,18	9	6	7	5	7	0,28	0,46
ICAL_HUMAN	Calpastatin	187	76526	4,98	10	8	7	6	7	0,2	0,39
TMM43_HUMAN	Transmembrane protein 43	186	44847	7,86	12	9	6	5	6	0,29	0,59
CDC37_HUMAN	Hsp90 co-chaperone Cdc37	186	44440	5,17	14	8	7	5	7	0,26	0,6
SNX9_HUMAN	Sorting nexin-9	185	66550	5,4	8	5	5	3	5	0,21	0,21
GDIR1_HUMAN	Rho GDP-dissociation inhibitor 1	185	23193	5,02	18	11	7	5	7	0,51	1,44
RABP2_HUMAN	Cellular retinoic acid-binding protein 2	185	15683	5,42	5	4	2	2	2	0,27	0,69
LAMP1_HUMAN	Lysosome-associated membrane glycoprotein 1	184	44854	9	12	7	6	3	6	0,12	0,32
MYO1B_HUMAN	Unconventional myosin-Ib	182	131902	9,43	25	11	16	9	16	0,23	0,33
P5CS_HUMAN	Delta-1-pyrroline-5-carboxylate synthase	181	87248	6,66	13	6	7	2	7	0,15	0,1
SYNC_HUMAN	Asparagine--tRNA ligase, cytoplasmic	179	62903	5,9	14	11	10	9	10	0,32	0,82
PRS6A_HUMAN	26S proteasome regulatory subunit 6A	179	49172	5,13	18	14	10	8	9	0,42	0,97
PUR6_HUMAN	Multifunctional protein ADE2	177	47049	6,95	13	7	10	5	10	0,5	0,56
UBP14_HUMAN	Ubiquitin carboxyl-terminal hydrolase 14	174	56033	5,2	11	4	8	3	8	0,27	0,25
GLU2B_HUMAN	Glucosidase 2 subunit beta	172	59388	4,33	20	11	10	8	10	0,26	0,88
HSPB6_HUMAN	Heat shock protein beta-6	170	17125	5,95	14	8	8	5	8	0,86	2,32
PSD13_HUMAN	26S proteasome non-ATPase regulatory subunit 13	169	42918	5,53	11	6	7	3	7	0,29	0,34
GLCM_HUMAN	Glucosylceramidase	169	59678	7,29	9	8	7	6	7	0,26	0,52
IF4G1_HUMAN	Eukaryotic translation initiation factor 4 gamma 1	166	175382	5,25	13	7	11	5	10	0,13	0,13
IF4G3_HUMAN	Eukaryotic translation initiation factor 4 gamma 3	38	176542	5,27	5	2	5	2	4	0,05	0,05
EIF3D_HUMAN	Eukaryotic translation initiation factor 3 subunit D	166	63932	5,79	9	6	5	3	5	0,16	0,22
SDHA_HUMAN	Succinate dehydrogenase [ubiquinone] flavoprotein subunit, mitochondrial	165	72645	7,06	12	6	5	2	5	0,19	0,12
E41L3_HUMAN	Band 4.1-like protein 3	164	120603	5,09	17	7	9	5	9	0,16	0,19
FABP5_HUMAN	Fatty acid-binding protein, epidermal	163	15155	6,6	12	4	5	1	5	0,46	0,31
RALA_HUMAN	Ras-related protein Ral-A	163	23552	6,66	10	7	7	4	7	0,57	1,02
CBSL_HUMAN	Cystathionine beta-synthase-like protein	162	60548	6,2	7	4	4	3	4	0,18	0,23
AKA12_HUMAN	A-kinase anchor protein 12	162	191367	4,37	11	5	9	3	9	0,09	0,07

[a]Protein score is calculated from the score of the peptide attributed to the protein.
[b]pI is (Predicted) isoelectric point.
[c]Number of matches is spectrum number matched to protein[#1].
[d]Number of significant matches is spectrum number that matches protein and exceeds the identification criteria.
[e]Number of sequences is number of peptides matched to protein[#2].
[f]Number of significant sequences is number of peptides exceeding the identification criteria matched to proteins.
[g]Number of unique sequences is a unique[#3] number of peptides matched to proteins.
[h]Sequence coverage is the ratio of the total number of matched peptide residues to the total length of the protein.
[i]Exponentially Modified Protein Abundance Index (http://www.matrixscience.com/help/quant_empai_help.html).

Table S2. Cont 4.

UniProt/SWISS-PROT ID	Description	Protein score[a]	Protein mass (kDa)	pI[b]	Num. of matches[c]	Num. of significant matches[d]	Num. of sequences[e]	Num. of significant sequences[f]	Num. of unique sequences[g]	Sequence coverage[h]	emPAI[i]
PRS4_HUMAN	26S proteasome regulatory subunit 4	162	49154	5.87	6	5	6	5	6	0.22	0.53
PSME1_HUMAN	Proteasome activator complex subunit 1	161	28705	5.78	9	6	7	4	7	0.44	0.78
CD109_HUMAN	CD109 antigen	161	161587	5.59	12	6	7	3	7	0.11	0.11
IMA3_HUMAN	Importin subunit alpha-3	160	57851	4.8	10	6	7	3	6	0.31	0.24
IMA4_HUMAN	Importin subunit alpha-4	93	57775	4.8	9	5	4	3	3	0.19	0.24
IMPA3_HUMAN	Inositol monophosphatase 3	159	38657	6.38	8	5	3	1	3	0.22	0.11
PARVA_HUMAN	Alpha-parvin	159	42217	5.69	5	4	3	3	3	0.12	0.34
HCDH_HUMAN	Hydroxyacyl-coenzyme A dehydrogenase, mitochondrial	158	34272	8.88	9	7	6	5	6	0.35	0.83
SPEE_HUMAN	Spermidine synthase	158	33803	5.3	16	12	11	8	11	0.65	1.67
PSMD4_HUMAN	26S proteasome non-ATPase regulatory subunit 4	158	40711	4.68	10	5	7	3	7	0.34	0.36
TGFI1_HUMAN	Transforming growth factor beta-1-induced transcript 1 protein	157	49782	6.62	12	6	7	5	7	0.3	0.52
QCR2_HUMAN	Cytochrome b-c1 complex subunit 2, mitochondrial	155	48413	8.74	10	6	6	4	6	0.25	0.54
SODC_HUMAN	Superoxide dismutase [Cu-Zn]	155	15926	5.7	9	4	5	2	5	0.45	0.68
NLTP_HUMAN	Non-specific lipid-transfer protein	154	58956	6.44	13	5	8	3	8	0.22	0.24
DHB4_HUMAN	Peroxisomal multifunctional enzyme type 2	154	79636	8.96	18	10	14	9	13	0.35	0.6
DDAH2_HUMAN	N(G),N(G)-dimethylarginine dimethylaminohydrolase 2	153	29625	5.66	8	4	6	2	6	0.44	0.52
TMM33_HUMAN	Transmembrane protein 33	153	27960	9.75	14	6	5	2	5	0.28	0.56
RL7_HUMAN	60S ribosomal protein L7	153	29207	10.66	22	7	15	5	14	0.58	1.03
RL6_HUMAN	60S ribosomal protein L6	153	32708	10.59	17	7	8	4	8	0.34	0.66
MIF_HUMAN	Macrophage migration inhibitory factor	151	12468	7.74	16	12	4	4	4	0.4	4.14
ERF3A_HUMAN	Eukaryotic peptide chain release factor GTP-binding subunit ERF3A	150	55720	5.45	11	5	5	4	2	0.19	0.35
LYAG_HUMAN	Lysosomal alpha-glucosidase	149	105257	5.62	13	6	10	4	10	0.18	0.17
P3H3_HUMAN	Prolyl 3-hydroxylase 3	149	81786	5.93	7	6	3	3	3	0.08	0.17
SC24D_HUMAN	Protein transport protein Sec24D	149	112936	6.91	19	10	12	7	12	0.22	0.3
SYHC_HUMAN	Histidine--tRNA ligase, cytoplasmic	148	57374	5.72	11	9	8	7	8	0.27	0.66
PPIC_HUMAN	Peptidyl-prolyl cis-trans isomerase C	148	22749	8.48	5	4	2	1	2	0.18	0.44
S10A6_HUMAN	Protein S100-A6	147	10173	5.33	20	5	8	2	8	0.67	1.21
PSB1_HUMAN	Proteasome subunit beta type-1	146	26472	8.27	10	6	6	3	6	0.41	0.6
PGRC2_HUMAN	Membrane-associated progesterone receptor component 2	146	23804	4.76	6	5	4	3	4	0.32	0.68
SIAS_HUMAN	Sialic acid synthase	146	40281	6.29	19	10	12	7	12	0.58	1.06
SAE2_HUMAN	SUMO-activating enzyme subunit 2	146	71179	5.15	8	8	3	3	3	0.12	0.26
SRPRB_HUMAN	Signal recognition particle receptor subunit beta	145	29684	9.17	9	5	6	4	6	0.28	0.75
GSDME_HUMAN	Gasdermin-E	144	54520	5.03	13	7	6	3	6	0.2	0.26
IKIP_HUMAN	Inhibitor of nuclear factor kappa-B kinase-interacting protein	143	39285	9.21	9	3	6	2	6	0.25	0.24
IF4H_HUMAN	Eukaryotic translation initiation factor 4H	143	27368	6.67	11	8	5	4	5	0.38	0.83
ENAH_HUMAN	Protein enabled homolog	142	66470	6.51	12	6	6	3	6	0.17	0.21
HCD2_HUMAN	3-hydroxyacyl-CoA dehydrogenase type-2	142	26906	7.66	9	4	7	4	7	0.52	0.85
HNRPQ_HUMAN	Heterogeneous nuclear ribonucleoprotein Q	141	69560	8.68	15	7	9	4	9	0.26	0.35
SPSY_HUMAN	Spermine synthase	141	41242	4.87	10	5	5	3	5	0.33	0.35
NAGK_HUMAN	N-acetyl-D-glucosamine kinase	140	37352	5.81	14	8	10	6	10	0.53	0.95
NPM_HUMAN	Nucleophosmin	140	32555	4.64	19	8	8	4	8	0.33	0.67
SYYC_HUMAN	Tyrosine--tRNA ligase, cytoplasmic	140	59106	6.61	24	11	20	9	20	0.46	0.89
ADT3_HUMAN	ADP/ATP translocase 3	140	32845	9.76	20	5	13	4	7	0.57	0.66
MYO1D_HUMAN	Unconventional myosin-Id	140	116129	9.44	14	6	11	5	11	0.17	0.2
COTL1_HUMAN	Coactosin-like protein	139	15935	5.54	10	3	6	2	6	0.59	0.67
CO3A1_HUMAN	Collagen alpha-1(III) chain	139	138479	6.21	12	6	10	5	10	0.12	0.16
CPSF7_HUMAN	Cleavage and polyadenylation specificity factor subunit 7	138	52018	7.82	4	2	2	1	2	0.08	0.08
NAMPT_HUMAN	Nicotinamide phosphoribosyltransferase	138	55487	6.69	7	3	6	2	6	0.22	0.16
LASP1_HUMAN	LIM and SH3 domain protein 1	137	29698	6.61	19	7	5	3	5	0.25	0.52
CD44_HUMAN	CD44 antigen	137	81487	5.13	10	5	6	4	6	0.1	0.23
MYPT1_HUMAN	Protein phosphatase 1 regulatory subunit 12A	137	115211	5.31	14	6	10	5	10	0.14	0.2
AN32A_HUMAN	Acidic leucine-rich nuclear phosphoprotein 32 family member A	136	28568	3.99	10	4	4	1	4	0.3	0.34
CSN2_HUMAN	COP9 signalosome complex subunit 2	136	51564	5.36	9	4	6	2	6	0.26	0.27
GT251_HUMAN	Procollagen galactosyltransferase 1	135	71590	6.85	13	7	9	6	9	0.26	0.42
UBP5_HUMAN	Ubiquitin carboxyl-terminal hydrolase 5	135	95725	4.91	8	4	7	3	7	0.15	0.14
RBP56_HUMAN	TATA-binding protein-associated factor 2N	50	61793	8.04	4	2	3	2	2	0.1	0.14
UAP1_HUMAN	UDP-N-acetylhexosamine pyrophosphorylase	134	58732	5.92	13	5	10	4	10	0.37	0.43
AHSA1_HUMAN	Activator of 90 kDa heat shock protein ATPase homolog 1	133	38250	5.41	7	6	4	4	4	0.24	0.54
SMD3_HUMAN	Small nuclear ribonucleoprotein Sm D3	133	13907	10.33	8	5	4	3	4	0.48	1.42
MAOX_HUMAN	NADP-dependent malic enzyme	132	64109	5.79	12	7	9	6	9	0.36	0.48
GLOD4_HUMAN	Glyoxalase domain-containing protein 4	132	34771	5.4	7	6	4	3	4	0.26	0.61
DESP_HUMAN	Desmoplakin	131	331569	6.44	23	3	19	2	19	0.11	0.03
KTN1_HUMAN	Kinectin	131	156179	5.52	21	6	16	5	16	0.21	0.14
MTAP_HUMAN	S-methyl-5'-thioadenosine phosphorylase	131	31216	6.75	3	2	3	2	3	0.21	0.3
UBR4_HUMAN	E3 ubiquitin-protein ligase UBR4	130	573476	5.7	30	7	28	7	28	0.13	0.05
HMOX1_HUMAN	Heme oxygenase 1	130	32798	7.88	5	4	4	3	4	0.22	0.46
PTK7_HUMAN	Inactive tyrosine-protein kinase 7	129	118317	6.67	17	7	11	4	11	0.18	0.15
HXK1_HUMAN	Hexokinase-1	128	102420	6.36	10	4	9	3	9	0.16	0.13
PPP6_HUMAN	Serine/threonine-protein phosphatase 6 catalytic subunit	128	35121	5.43	3	2	2	1	2	0.16	0.13
TFG_HUMAN	Protein TFG	127	43421	4.94	14	5	6	3	6	0.3	0.33
LAT1_HUMAN	Large neutral amino acids transporter small subunit 1	127	54974	7.9	8	5	4	2	4	0.11	0.16
CSN7A_HUMAN	COP9 signalosome complex subunit 7a	127	30258	8.33	8	3	6	2	6	0.32	0.31
EIF3L_HUMAN	Eukaryotic translation initiation factor 3 subunit L	126	66684	5.93	8	6	6	4	6	0.15	0.28
DLDH_HUMAN	Dihydrolipoyl dehydrogenase, mitochondrial	126	54143	7.95	17	5	10	4	10	0.32	0.36
PIMT_HUMAN	Protein-L-isoaspartate(D-aspartate) O-methyltransferase	126	24621	6.7	7	3	5	2	4	0.43	0.65
XRCC6_HUMAN	X-ray repair cross-complementing protein 6	125	69799	6.23	13	5	11	4	11	0.22	0.27
POSTN_HUMAN	Periostin	125	93255	7.27	14	8	9	6	9	0.22	0.31
DOPD_HUMAN	D-dopachrome decarboxylase	125	12704	6.71	7	5	4	3	4	0.69	1.63
PDLI4_HUMAN	PDZ and LIM domain protein 4	124	35376	8.07	13	4	9	3	9	0.45	0.6
XRP2_HUMAN	Protein XRP2	124	39615	5	4	4	1	1	1	0.08	0.23
MP2K2_HUMAN	Dual specificity mitogen-activated protein kinase kinase 2	123	44396	6.12	5	3	3	2	3	0.15	0.21
MRP1_HUMAN	Multidrug resistance-associated protein 1	123	171481	6.71	7	3	5	2	4	0.06	0.05
MIC60_HUMAN	MICOS complex subunit MIC60	123	83626	6.08	7	5	5	3	5	0.16	0.16
ABCE1_HUMAN	ATP-binding cassette sub-family E member 1	122	67271	8.63	5	4	4	3	4	0.11	0.2
PDLI7_HUMAN	PDZ and LIM domain protein 7	122	49813	8.76	20	11	12	6	12	0.42	0.65
BLMH_HUMAN	Bleomycin hydrolase	122	52528	5.87	6	5	3	2	2	0.16	0.17
DLRB1_HUMAN	Dynein light chain roadblock-type 1	120	10915	6.58	7	6	5	4	5	0.74	3.44
RBM3_HUMAN	RNA-binding protein 3	120	17160	8.86	7	5	3	2	3	0.43	0.61
CATK_HUMAN	Cathepsin K	120	36942	8.72	9	6	5	3	5	0.37	0.4
SEPT9_HUMAN	Septin-9	120	65361	9.06	18	7	12	6	12	0.28	0.47
UBXN1_HUMAN	UBX domain-containing protein 1	120	33305	5.23	4	3	2	1	2	0.18	0.28
GLRX1_HUMAN	Glutaredoxin-1	120	11768	8.33	3	2	1	1	1	0.2	0.41
PSDE_HUMAN	26S proteasome non-ATPase regulatory subunit 14	119	34555	6.06	7	4	4	2	4	0.3	0.27
TXNL1_HUMAN	Thioredoxin-like protein 1	118	32231	4.84	9	5	6	2	6	0.34	0.29
MARE1_HUMAN	Microtubule-associated protein RP/EB family member 1	118	29980	5.02	13	6	10	5	10	0.6	1
CDV3_HUMAN	Protein CDV3 homolog	117	27318	6.06	4	1	3	1	3	0.32	0.16
SORCN_HUMAN	Sorcin	117	21662	5.32	6	5	4	4	4	0.33	1.14
ENPP1_HUMAN	Ectonucleotide pyrophosphatase/phosphodiesterase family member 1	117	104857	6.76	7	6	5	4	5	0.11	0.17
MYDGF_HUMAN	Myeloid-derived growth factor	117	18783	6.2	6	4	3	2	3	0.18	0.55
ATP5H_HUMAN	ATP synthase subunit d, mitochondrial	116	18480	5.21	7	3	4	2	4	0.42	0.56

[a]Protein score is calculated from the score of the peptide attributed to the protein.

[b]pI is (Predicted) isoelectric point.

[c]Number of matches is spectrum number matched to protein[#1].

[d]Number of significant matches is spectrum number that matches protein and exceeds the identification criteria.

[e]Number of sequences is number of peptides matched to protein[#2].

[f]Number of significant sequences is number of peptides exceeding the identification criteria matched to proteins.

[g]Number of unique sequences is a unique[#3] number of peptides matched to proteins.

[h]Sequence coverage is the ratio of the total number of matched peptide residues to the total length of the protein.

[i]Exponentially Modified Protein Abundance Index (http://www.matrixscience.com/help/quant_empai_help.html).

Table S2. Cont 5.

UniProt/SWISS-PROT ID	Description	Protein score[a]	Protein mass (kDa)	pI[b]	Num. of matches[c]	Num. of significant matches[d]	Num. of sequences[e]	Num. of significant sequences[f]	Num. of unique sequences[g]	Sequence coverage[h]	emPAI[i]
DHRS7_HUMAN	Dehydrogenase/reductase SDR family member 7	116	38274	8.59	9	5	5	3	5	0.31	0.38
CBX3_HUMAN	Chromobox protein homolog 3	115	20798	5.23	6	5	5	4	5	0.45	1.21
ITA2_HUMAN	Integrin alpha-2	115	129214	5.16	13	6	12	6	12	0.19	0.21
NAA15_HUMAN	N-alpha-acetyltransferase 15, NatA auxiliary subunit	115	101208	7.23	11	5	7	3	7	0.14	0.13
LGUL_HUMAN	Lactoylglutathione lyase	115	20764	5.12	8	3	5	2	5	0.39	0.49
LAMP2_HUMAN	Lysosome-associated membrane glycoprotein 2	114	44932	5.35	6	5	3	3	3	0.09	0.32
PDCD6_HUMAN	Programmed cell death protein 6	114	21855	5.16	5	3	3	2	3	0.28	0.46
LICH_HUMAN	Lysosomal acid lipase/cholesteryl ester hydrolase	114	45390	6.42	1	1	1	1	1	0.05	0.1
PTTG_HUMAN	Pituitary tumor-transforming gene 1 protein-interacting protein	114	20310	9.14	3	3	1	1	1	0.1	0.22
PRRC1_HUMAN	Protein PRRC1	114	46672	5.56	4	3	2	2	2	0.07	0.2
F10A1_HUMAN	Hsc70-interacting protein	113	41305	5.18	11	5	6	3	6	0.21	0.35
ATPD_HUMAN	ATP synthase subunit delta, mitochondrial	113	17479	5.38	6	5	3	2	3	0.33	0.6
STX12_HUMAN	Syntaxin-12	113	31622	5.45	7	3	6	3	6	0.36	0.48
LA_HUMAN	Lupus La protein	113	46808	6.68	9	3	8	3	8	0.28	0.31
BCAT1_HUMAN	Branched-chain-amino-acid aminotransferase, cytosolic	112	42939	5.17	15	6	10	4	10	0.51	0.47
PDLI5_HUMAN	PDZ and LIM domain protein 5	112	63904	8.55	25	10	14	7	9	0.34	0.58
RCN2_HUMAN	Reticulocalbin-2	112	36854	4.26	14	9	9	5	9	0.38	0.76
PUR2_HUMAN	Trifunctional purine biosynthetic protein adenosine-3	112	107699	6.26	14	4	11	4	11	0.23	0.17
PYR1_HUMAN	CAD protein	112	242829	6.02	16	6	10	4	10	0.1	0.07
ATPO_HUMAN	ATP synthase subunit O, mitochondrial	112	23263	9.97	10	4	5	2	5	0.37	0.43
HEBP2_HUMAN	Heme-binding protein 2	112	22861	4.58	3	2	3	2	3	0.24	0.43
DJB11_HUMAN	DnaJ homolog subfamily B member 11	111	40489	5.81	10	3	4	2	4	0.22	0.23
DNJA2_HUMAN	DnaJ homolog subfamily A member 2	111	45717	6.06	4	4	3	3	3	0.18	0.31
TMOD3_HUMAN	Tropomodulin-3	111	39570	5.08	6	4	5	3	5	0.32	0.37
AATM_HUMAN	Aspartate aminotransferase, mitochondrial	111	47487	9.14	6	3	5	2	5	0.18	0.19
TADBP_HUMAN	TAR DNA-binding protein 43	111	44711	5.85	5	5	4	4	4	0.18	0.45
OCAD1_HUMAN	OCIA domain-containing protein 1	111	27609	7.02	3	2	2	1	2	0.11	0.16
VATE1_HUMAN	V-type proton ATPase subunit E 1	110	26129	7.71	9	6	5	3	5	0.34	0.61
LIMS1_HUMAN	LIM and senescent cell antigen-like-containing domain protein 1	110	37226	8.43	6	5	3	3	3	0.15	0.4
PEF1_HUMAN	Peflin	109	30361	6.1	2	2	1	1	1	0.06	0.15
SGCD_HUMAN	Delta-sarcoglycan	109	32051	9.24	2	2	2	2	2	0.11	0.3
DCTN3_HUMAN	Dynactin subunit 3	109	21106	5.37	8	3	5	3	5	0.52	0.8
DDRGK_HUMAN	DDRGK domain-containing protein 1	109	35589	5.12	5	5	4	4	4	0.26	0.79
FUBP2_HUMAN	Far upstream element-binding protein 2	109	73070	6.85	17	4	14	3	14	0.32	0.19
PACN2_HUMAN	Protein kinase C and casein kinase substrate in neurons protein 2	109	55704	5.08	8	4	5	2	5	0.17	0.16
TWF1_HUMAN	Twinfilin-1	108	40258	6.48	6	6	3	3	3	0.14	0.36
RSU1_HUMAN	Ras suppressor protein 1	108	31521	8.57	13	8	8	5	8	0.64	0.93
WDR11_HUMAN	WD repeat-containing protein 11	108	136598	6.48	5	3	3	1	3	0.05	0.03
GNA1_HUMAN	Glucosamine 6-phosphate N-acetyltransferase	107	20736	8.17	3	3	3	3	3	0.29	0.81
PROF2_HUMAN	Profilin-2	107	15036	6.55	8	6	3	3	3	0.38	1.27
GOLP3_HUMAN	Golgi phosphoprotein 3	107	33790	6.05	8	4	4	2	4	0.26	0.28
TGM2_HUMAN	Protein-glutamine gamma-glutamyltransferase 2	107	77280	5.11	15	5	10	4	10	0.31	0.24
CX6A1_HUMAN	Cytochrome c oxidase subunit 6A1, mitochondrial	107	12147	9.3	5	4	2	1	2	0.46	0.4
EIF3B_HUMAN	Eukaryotic translation initiation factor 3 subunit B	107	92424	4.89	18	7	11	4	9	0.25	0.2
BLVRB_HUMAN	Flavin reductase (NADPH)	106	22105	7.13	7	4	3	1	3	0.28	0.21
LYOX_HUMAN	Protein-lysine 6-oxidase	106	46915	8.36	7	2	4	2	4	0.16	0.19
CHMP5_HUMAN	Charged multivesicular body protein 5	106	24555	4.68	3	3	2	2	2	0.22	0.4
MIC10_HUMAN	MICOS complex subunit MIC10	105	8802	8.68	2	2	1	1	1	0.22	0.58
CCD80_HUMAN	Coiled-coil domain-containing protein 80	104	108106	9.72	5	4	3	2	3	0.09	0.08
STOM_HUMAN	Erythrocyte band 7 integral membrane protein	104	31711	7.71	7	5	2	1	2	0.13	0.14
AK1C1_HUMAN	Aldo-keto reductase family 1 member C1	104	36765	8.02	10	5	5	4	3	0.26	0.57
AK1C3_HUMAN	Aldo-keto reductase family 1 member C3	81	36830	8.06	9	3	4	3	2	0.2	0.4
PCKGM_HUMAN	Phosphoenolpyruvate carboxykinase [GTP], mitochondrial	104	70685	7.57	13	7	9	4	9	0.28	0.27
MGST1_HUMAN	Microsomal glutathione S-transferase 1	104	17587	9.41	6	3	3	2	3	0.25	1.02
6PGL_HUMAN	6-phosphogluconolactonase	104	27530	5.7	9	3	6	2	6	0.3	0.35
PRKDC_HUMAN	DNA-dependent protein kinase catalytic subunit	103	468788	6.75	23	6	18	4	18	0.08	0.04
HNRH3_HUMAN	Heterogeneous nuclear ribonucleoprotein H3	101	36903	6.37	4	2	3	1	3	0.21	0.12
SYTC_HUMAN	Threonine--tRNA ligase, cytoplasmic	101	83382	6.23	21	8	13	4	13	0.3	0.22
TOIP1_HUMAN	Torsin-1A-interacting protein 1	101	66208	8.22	8	5	4	2	4	0.17	0.13
AR6P1_HUMAN	ADP-ribosylation factor-like protein 6-interacting protein 1	101	23347	9.38	4	4	1	1	1	0.14	0.19
PTN11_HUMAN	Tyrosine-protein phosphatase non-receptor type 11	100	68393	6.87	11	5	7	4	7	0.23	0.28
TOM70_HUMAN	Mitochondrial import receptor subunit TOM70	100	67412	6.75	5	4	4	3	4	0.12	0.2
NP1L4_HUMAN	Nucleosome assembly protein 1-like 4	100	42797	4.6	8	3	5	1	4	0.26	0.1
IF1AX_HUMAN	Eukaryotic translation initiation factor 1A, X-chromosomal	100	16450	5.07	3	3	1	1	1	0.1	0.28
SYPL1_HUMAN	Synaptophysin-like protein 1	99	28547	8.69	2	2	1	1	1	0.07	0.16
PP1R7_HUMAN	Protein phosphatase 1 regulatory subunit 7	99	41539	4.84	8	4	7	4	7	0.31	0.49
CRTAP_HUMAN	Cartilage-associated protein	99	46532	5.5	10	4	6	4	5	0.22	0.43
COPD_HUMAN	Coatomer subunit delta	98	57174	5.89	19	5	16	5	16	0.48	0.44
ECM29_HUMAN	Proteasome-associated protein ECM29 homolog	98	204160	6.74	16	5	10	3	10	0.12	0.06
CTGF_HUMAN	Connective tissue growth factor	98	38065	8.43	16	6	10	4	10	0.43	0.55
ARF6_HUMAN	ADP-ribosylation factor 6	98	20069	9.04	11	4	7	2	6	0.58	0.51
DCNL1_HUMAN	DCN1-like protein 1	98	30105	5.18	4	2	3	1	3	0.2	0.15
VAPA_HUMAN	Vesicle-associated membrane protein-associated protein A	98	27875	8.8	10	4	7	2	7	0.4	0.35
CTND1_HUMAN	Catenin delta-1	97	108103	5.86	20	7	13	4	13	0.24	0.21
UTRO_HUMAN	Utrophin	97	394220	5.2	10	4	7	1	7	0.04	0.01
ULA1_HUMAN	NEDD8-activating enzyme E1 regulatory subunit	97	60209	5.25	4	3	3	2	3	0.13	0.15
MON2_HUMAN	Protein MON2 homolog	97	190237	5.73	4	3	1	1	1	0.02	0.02
PGM2_HUMAN	Phosphoglucomutase-2	97	68240	6.28	8	6	4	2	4	0.13	0.2
CKAP5_HUMAN	Cytoskeleton-associated protein 5	97	225352	7.95	13	5	7	3	7	0.07	0.06
KLC1_HUMAN	Kinesin light chain 1	97	65269	5.87	10	3	7	3	7	0.2	0.21
ERP44_HUMAN	Endoplasmic reticulum resident protein 44	96	46941	5.09	9	4	6	2	6	0.16	0.19
FAAA_HUMAN	Fumarylacetoacetase	96	46344	6.46	3	2	2	1	2	0.12	0.09
GCP60_HUMAN	Golgi resident protein GCP60	96	60556	5.02	7	3	6	3	6	0.21	0.23
ANXA7_HUMAN	Annexin A7	95	52706	5.52	8	4	5	3	5	0.14	0.27
C1TC_HUMAN	C-1-tetrahydrofolate synthase, cytoplasmic	96	101495	6.89	15	7	12	6	12	0.21	0.28
SRP72_HUMAN	Signal recognition particle subunit SRP72	96	74560	9.31	13	7	8	4	8	0.25	0.25
DPM1_HUMAN	Dolichol-phosphate mannosyltransferase subunit 1	95	29616	9.57	5	2	4	1	4	0.34	0.15
CRYAB_HUMAN	Alpha-crystallin B chain	95	20146	6.76	6	4	3	2	3	0.29	0.51
CTR1_HUMAN	High affinity cationic amino acid transporter 1	95	67594	5.29	10	6	4	2	4	0.1	0.13
PSA_HUMAN	Puromycin-sensitive aminopeptidase	95	103211	5.49	16	4	13	3	13	0.21	0.13
OTUB1_HUMAN	Ubiquitin thioesterase OTUB1	95	31264	4.85	7	5	4	3	4	0.33	0.7
PYRG1_HUMAN	CTP synthase 1	94	66648	6.02	10	6	6	3	6	0.17	0.21
ATP5L_HUMAN	ATP synthase subunit g, mitochondrial	94	11421	9.65	9	5	3	2	3	0.5	1.03
DYN2_HUMAN	Dynamin-2	94	98003	7.04	9	4	7	4	7	0.13	0.19
CNPY2_HUMAN	Protein canopy homolog 2	94	20639	4.81	10	4	6	3	6	0.45	0.82
ERLN2_HUMAN	Erlin-2	93	37815	5.47	8	3	5	2	5	0.25	0.25
GPNMB_HUMAN	Transmembrane glycoprotein NMB	93	63882	6.17	4	2	2	1	2	0.05	0.07
LARP1_HUMAN	La-related protein 1	93	123434	8.91	5	3	4	2	4	0.06	0.07
DC1L1_HUMAN	Cytoplasmic dynein 1 light intermediate chain 1	93	56544	6.01	3	2	2	1	2	0.1	0.08
ANM5_HUMAN	Protein arginine N-methyltransferase 5	93	72638	5.88	9	5	6	4	6	0.25	0.26

[a]Protein score is calculated from the score of the peptide attributed to the protein.
[b]pI is (Predicted) isoelectric point.
[c]Number of matches is spectrum number matched to protein[#1].
[d]Number of significant matches is spectrum number that matches protein and exceeds the identification criteria.
[e]Number of sequences is number of peptides matched to protein[#2].
[f]Number of significant sequences is number of peptides exceeding the identification criteria matched to proteins.
[g]Number of unique sequences is a unique[#3] number of peptides matched to proteins.
[h]Sequence coverage is the ratio of the total number of matched peptide residues to the total length of the protein.
[i]Exponentially Modified Protein Abundance Index (http://www.matrixscience.com/help/quant_empai_help.html).

Table S2. Cont 6.

UniProt/SWISS-PROT ID	Description	Protein score[a]	Protein mass (kDa)	pI[b]	Num. of matches[c]	Num. of significant matches[d]	Num. of sequences[e]	Num. of significant sequences[f]	Num. of unique sequences[g]	Sequence coverage[h]	emPAI[i]
CYFP1_HUMAN	Cytoplasmic FMR1-interacting protein 1	93	145089	6.46	8	3	8	3	8	0.1	0.09
PAK2_HUMAN	Serine/threonine-protein kinase PAK 2	92	58006	5.69	7	4	4	3	4	0.13	0.24
PIGS_HUMAN	GPI transamidase component PIG-S	92	61617	6.05	3	3	2	2	2	0.09	0.15
IF5_HUMAN	Eukaryotic translation initiation factor 5	91	49192	5.41	8	3	5	2	5	0.18	0.18
ARMC9_HUMAN	LisH domain-containing protein ARMC9	91	91704	5.83	8	3	7	3	7	0.15	0.15
CD166_HUMAN	CD166 antigen	91	65061	5.92	9	5	5	4	5	0.2	0.29
IF2B_HUMAN	Eukaryotic translation initiation factor 2 subunit 2	91	38364	5.6	7	5	4	3	4	0.2	0.38
CRK_HUMAN	Adapter molecule crk	91	33810	5.38	1	1	1	1	1	0.06	0.13
GRN_HUMAN	Granulins	91	63500	6.43	6	3	4	2	4	0.11	0.22
SRP09_HUMAN	Signal recognition particle 9 kDa protein	91	10105	7.77	2	2	1	1	1	0.13	0.49
UMPS_HUMAN	Uridine 5'-monophosphate synthase	90	52189	6.81	4	3	3	2	3	0.09	0.17
RO60_HUMAN	60 kDa SS-A/Ro ribonucleoprotein	90	60631	8.27	6	4	4	3	4	0.14	0.23
FRIH_HUMAN	Ferritin heavy chain	90	21212	5.3	12	4	8	3	8	0.7	0.79
NDRG1_HUMAN	Protein NDRG1	90	42808	5.49	6	3	6	3	6	0.24	0.34
RL13_HUMAN	60S ribosomal protein L13	89	24247	11.65	11	4	6	2	6	0.27	0.41
HEXB_HUMAN	Beta-hexosaminidase subunit beta	89	63071	6.29	15	5	10	4	10	0.26	0.3
SGTB_HUMAN	Small glutamine-rich tetratricopeptide repeat-containing protein beta	89	33408	4.8	3	2	2	2	2	0.17	0.28
HNRL2_HUMAN	Heterogeneous nuclear ribonucleoprotein U-like protein 2	88	85052	4.85	8	3	6	1	6	0.17	0.05
ERO1A_HUMAN	ERO1-like protein alpha	88	54358	5.48	6	2	6	2	4	0.24	0.17
SYIM_HUMAN	Isoleucine--tRNA ligase, mitochondrial	88	113719	6.78	6	3	5	2	5	0.12	0.08
MMS19_HUMAN	MMS19 nucleotide excision repair protein homolog	88	113217	5.92	5	2	5	2	5	0.11	0.08
PCNA_HUMAN	Proliferating cell nuclear antigen	87	28750	4.57	9	4	5	2	5	0.43	0.33
TOM1_HUMAN	Target of Myb protein 1	87	53785	4.59	10	3	6	2	6	0.29	0.17
PGRC1_HUMAN	Membrane-associated progesterone receptor component 1	87	21658	4.56	3	3	1	1	1	0.12	0.21
S10AG_HUMAN	Protein S100-A16	87	11794	6.28	7	2	4	2	4	0.53	1
COMD1_HUMAN	COMM domain-containing protein 1	87	21165	5.85	2	2	2	2	2	0.18	0.48
HIP1_HUMAN	Huntingtin-interacting protein 1	87	116148	5.2	11	3	5	1	5	0.11	0.04
XPO1_HUMAN	Exportin-1	86	123306	5.71	17	5	10	3	10	0.16	0.11
CPNE1_HUMAN	Copine-1	86	59022	5.52	5	4	3	3	3	0.11	0.24
KCY_HUMAN	UMP-CMP kinase	86	22208	5.44	8	6	5	4	5	0.3	1.1
GNA11_HUMAN	Guanine nucleotide-binding protein subunit alpha-11	86	42097	5.51	5	3	4	3	2	0.2	0.35
HEBP1_HUMAN	Heme-binding protein 1	86	21084	5.71	2	1	2	1	2	0.15	0.22
HEXA_HUMAN	Beta-hexosaminidase subunit alpha	85	60664	5.04	8	4	5	2	5	0.12	0.15
SODM_HUMAN	Superoxide dismutase [Mn], mitochondrial	85	24735	8.35	6	2	3	2	3	0.25	0.4
EP15R_HUMAN	Epidermal growth factor receptor substrate 15-like 1	84	94197	4.93	4	3	4	3	4	0.1	0.14
CA198_HUMAN	Uncharacterized protein C1orf198	84	36324	5.6	6	5	2	2	2	0.15	0.26
SULF1_HUMAN	Extracellular sulfatase Sulf-1	83	100962	9.23	8	4	5	1	5	0.14	0.04
MAT2B_HUMAN	Methionine adenosyltransferase 2 subunit beta	83	37528	6.9	3	2	2	1	2	0.16	0.12
IPO4_HUMAN	Importin-4	83	118640	4.88	5	3	4	2	4	0.06	0.07
TRI25_HUMAN	E3 ubiquitin/ISG15 ligase TRIM25	82	70928	8.44	5	4	3	2	3	0.11	0.12
STAT3_HUMAN	Signal transducer and activator of transcription 3	82	88011	5.94	9	3	8	2	8	0.18	0.1
DBNL_HUMAN	Drebrin-like protein	82	48178	5.02	9	3	6	3	6	0.18	0.3
TIF1B_HUMAN	Transcription intermediary factor 1-beta	82	88493	5.52	12	4	8	3	8	0.21	0.15
PSMD5_HUMAN	26S proteasome non-ATPase regulatory subunit 5	82	56160	5.35	5	3	3	2	3	0.1	0.25
GLGB_HUMAN	1,4-alpha-glucan-branching enzyme	82	80423	5.87	17	5	8	2	8	0.17	0.11
ITA11_HUMAN	Integrin alpha-11	81	133386	6.24	15	2	11	2	11	0.15	0.06
RL29_HUMAN	60S ribosomal protein L29	81	17741	11.66	16	5	2	1	2	0.17	0.26
STML2_HUMAN	Stomatin-like protein 2, mitochondrial	81	38510	6.88	9	5	6	2	6	0.35	0.24
CSPG2_HUMAN	Versican core protein	81	372590	4.43	19	4	14	3	14	0.07	0.03
DTD1_HUMAN	D-aminoacyl-tRNA deacylase 1	80	23409	8.35	8	4	4	3	4	0.35	0.7
PRDX3_HUMAN	Thioredoxin-dependent peroxide reductase, mitochondrial	80	27675	7.67	7	2	6	2	6	0.34	0.35
USMG5_HUMAN	Up-regulated during skeletal muscle growth protein 5	80	6453	9.78	2	2	1	1	1	0.26	0.85
AIMP2_HUMAN	Aminoacyl tRNA synthase complex-interacting multifunctional protein 2	80	35326	8.45	11	5	5	3	5	0.3	0.42
PXDN_HUMAN	Peroxidasin homolog	80	165170	6.79	8	4	7	4	7	0.09	0.11
ANM1_HUMAN	Protein arginine N-methyltransferase 1	80	41489	5.24	6	4	6	4	6	0.25	0.49
EFTU_HUMAN	Elongation factor Tu, mitochondrial	79	49510	7.26	7	4	5	4	5	0.24	0.4
PEX19_HUMAN	Peroxisomal biogenesis factor 19	79	32786	4.26	3	3	2	2	2	0.17	0.46
STX7_HUMAN	Syntaxin-7	79	29797	5.41	5	3	3	3	3	0.22	0.52
2ABA_HUMAN	Serine/threonine-protein phosphatase 2A 55 kDa regulatory subunit B alpha isoform	78	51659	5.82	8	3	6	2	6	0.25	0.18
ACADV_HUMAN	Very long-chain specific acyl-CoA dehydrogenase, mitochondrial	78	70345	8.92	8	5	8	5	8	0.23	0.35
RTCA_HUMAN	RNA 3'-terminal phosphate cyclase	76	39311	8.01	2	2	2	2	2	0.11	0.24
NUCB1_HUMAN	Nucleobindin-1	77	53846	5.15	10	3	8	3	8	0.23	0.26
MINP1_HUMAN	Multiple inositol polyphosphate phosphatase 1	77	55016	7.93	3	1	3	1	3	0.11	0.08
PARP4_HUMAN	Poly [ADP-ribose] polymerase 4	77	192472	5.43	2	2	2	2	2	0.03	0.04
MPV17_HUMAN	Protein Mpv17	77	19720	9.54	2	2	1	1	1	0.13	0.23
TALDO_HUMAN	Transaldolase	77	37516	6.36	8	5	7	4	7	0.24	0.56
FKBP3_HUMAN	Peptidyl-prolyl cis-trans isomerase FKBP3	76	25161	9.29	4	4	2	2	2	0.18	0.39
STK24_HUMAN	Serine/threonine-protein kinase 24	76	49277	5.49	4	2	3	2	3	0.12	0.18
CD63_HUMAN	CD63 antigen	76	25619	8.14	10	5	4	2	4	0.2	0.62
SNX6_HUMAN	Sorting nexin-6	76	46620	5.81	7	3	5	3	5	0.24	0.31
NCPR_HUMAN	NADPH--cytochrome P450 reductase	76	76641	5.38	4	2	3	1	3	0.09	0.06
IVD_HUMAN	Isovaleryl-CoA dehydrogenase, mitochondrial	76	46290	8.45	2	1	2	1	2	0.09	0.09
GSTK1_HUMAN	Glutathione S-transferase kappa 1	76	25480	8.5	8	6	5	5	5	0.42	1.25
S12A4_HUMAN	Solute carrier family 12 member 4	76	120572	6.02	4	2	3	1	3	0.05	0.04
UBA6_HUMAN	Ubiquitin-like modifier-activating enzyme 6	75	117895	5.76	8	5	6	3	6	0.09	0.11
SERC1_HUMAN	Serine incorporator 1	75	50461	5.59	2	1	2	1	2	0.06	0.09
ASAH1_HUMAN	Acid ceramidase	74	44631	7.52	5	2	4	2	4	0.23	0.21
KAD1_HUMAN	Adenylate kinase isoenzyme 1	74	21621	8.73	8	3	4	2	4	0.34	0.46
VATL_HUMAN	V-type proton ATPase 16 kDa proteolipid subunit	74	15725	7.98	1	1	1	1	1	0.2	0.3
SYK_HUMAN	Lysine--tRNA ligase	73	68005	5.94	7	6	5	4	5	0.16	0.28
RRAS_HUMAN	Ras-related protein R-Ras	73	23466	6.43	9	4	7	3	7	0.57	0.7
JIP4_HUMAN	C-Jun-amino-terminal kinase-interacting protein 4	73	146115	5.05	8	2	6	2	6	0.1	0.06
TWF2_HUMAN	Twinfilin-2	73	39523	6.37	6	4	4	2	4	0.21	0.23
FA98A_HUMAN	Protein FAM98A	73	55366	9.15	13	3	5	2	4	0.16	0.16
ILVBL_HUMAN	Acetolactate synthase-like protein	73	67825	8.43	12	6	7	4	7	0.24	0.28
E2AK2_HUMAN	Interferon-induced, double-stranded RNA-activated protein kinase	73	62056	8.58	1	1	1	1	1	0.02	0.07
DJB12_HUMAN	DnaJ homolog subfamily B member 12	72	41793	8.67	5	2	3	1	3	0.13	0.1
OXSR1_HUMAN	Serine/threonine-protein kinase OSR1	72	57986	6.03	5	3	5	3	5	0.17	0.24
SYQ_HUMAN	Glutamine--tRNA ligase	72	87743	6.71	8	3	5	2	5	0.1	0.1
AAAT_HUMAN	Neutral amino acid transporter B(0)	72	56562	5.34	3	3	3	3	3	0.11	0.25
GNS_HUMAN	N-acetylglucosamine-6-sulfatase	72	62042	8.6	7	2	5	2	5	0.16	0.14
TBCE_HUMAN	Tubulin-specific chaperone E	72	59309	6.32	6	3	5	2	5	0.16	0.15
CARM1_HUMAN	Histone-arginine methyltransferase CARM1	72	65811	6.25	9	5	6	3	6	0.19	0.21
CC90B_HUMAN	Coiled-coil domain-containing protein 90B, mitochondrial	72	29487	6.99	2	2	1	1	1	0.09	0.15
NIT2_HUMAN	Omega-amidase NIT2	71	30589	6.82	6	4	4	3	4	0.28	0.5
AK1A1_HUMAN	Alcohol dehydrogenase [NADP(+)]	71	36550	6.32	11	7	7	5	7	0.36	0.77
T126A_HUMAN	Transmembrane protein 126A	71	21513	9.36	4	2	3	1	3	0.38	0.21
RWDD1_HUMAN	RWD domain-containing protein 1	71	27922	4.11	3	3	2	2	2	0.23	0.34
TFR1_HUMAN	Transferrin receptor protein 1	71	84818	6.18	12	6	9	4	9	0.19	0.22
BGAL_HUMAN	Beta-galactosidase	70	76027	6.1	9	4	8	3	8	0.14	0.18

[a]Protein score is calculated from the score of the peptide attributed to the protein.
[b]pI is (Predicted) isoelectric point.
[c]Number of matches is spectrum number matched to protein[#1].
[d]Number of significant matches is spectrum number that matches protein and exceeds the identification criteria.
[e]Number of sequences is number of peptides matched to protein[#2].
[f]Number of significant sequences is number of peptides exceeding the identification criteria matched to proteins.
[g]Number of unique sequences is a unique[#3] number of peptides matched to proteins.
[h]Sequence coverage is the ratio of the total number of matched peptide residues to the total length of the protein.
[i]Exponentially Modified Protein Abundance Index (http://www.matrixscience.com/help/quant_empai_help.html).

Table S2. Cont 7.

UniProt/SWISS-PROT ID	Description	Protein score[a]	Protein mass (kDa)	pI[b]	Num. of matches[c]	Num. of significant matches[d]	Num. of sequences[e]	Num. of significant sequences[f]	Num. of unique sequences[g]	Sequence coverage[h]	emPAI[i]
CSTFT_HUMAN	Cleavage stimulation factor subunit 2 tau variant	70	64396	6.79	5	2	4	1	4	0.15	0.07
SAM50_HUMAN	Sorting and assembly machinery component 50 homolog	70	51943	6.44	3	2	3	2	3	0.14	0.17
ASPH_HUMAN	Aspartyl/asparaginyl beta-hydroxylase	70	85809	4.92	3	1	3	1	3	0.07	0.05
GSTM3_HUMAN	Glutathione S-transferase Mu 3	70	26542	5.37	2	2	2	2	2	0.14	0.37
MPRI_HUMAN	Cation-independent mannose-6-phosphate receptor	70	274199	5.64	16	2	13	1	13	0.1	0.02
EEA1_HUMAN	Early endosome antigen 1	70	162367	5.55	14	4	13	4	13	0.17	0.11
AT2B1_HUMAN	Plasma membrane calcium-transporting ATPase 1	69	138668	5.73	8	3	6	2	6	0.08	0.06
ROA0_HUMAN	Heterogeneous nuclear ribonucleoprotein A0	69	30822	9.34	4	2	4	2	4	0.25	0.31
LEG3_HUMAN	Galectin-3	69	26136	8.57	12	3	7	2	7	0.35	0.37
HNRPD_HUMAN	Heterogeneous nuclear ribonucleoprotein D0	69	38410	7.62	10	6	4	3	3	0.13	0.38
EIF3H_HUMAN	Eukaryotic translation initiation factor 3 subunit H	69	39905	6.09	3	3	3	3	3	0.17	0.37
RAB13_HUMAN	Ras-related protein Rab-13	68	22760	9.27	5	1	4	1	3	0.28	0.2
MMP2_HUMAN	72 kDa type IV collagenase	68	73835	5.26	5	3	2	1	2	0.09	0.06
CUTA_HUMAN	Protein CutA	68	19104	5.42	6	1	6	1	6	0.48	0.24
RAB21_HUMAN	Ras-related protein Rab-21	68	24332	8.11	5	1	4	1	4	0.28	0.19
XPOT_HUMAN	Exportin-T	68	109893	5.24	11	2	9	2	9	0.17	0.08
CPNE3_HUMAN	Copine-3	68	60092	5.6	6	4	5	3	5	0.2	0.23
MYADM_HUMAN	Myeloid-associated differentiation marker	68	35250	8.53	3	2	1	1	1	0.07	0.12
VATH_HUMAN	V-type proton ATPase subunit H	67	55847	6.07	7	2	7	2	7	0.29	0.16
NICA_HUMAN	Nicastrin	67	78362	5.67	6	3	4	1	4	0.1	0.05
CX7A2_HUMAN	Cytochrome c oxidase subunit 7A2, mitochondrial	67	9390	9.75	3	1	2	1	2	0.45	0.53
IPO9_HUMAN	Importin-9	67	115889	4.71	3	2	3	2	3	0.06	0.07
INF2_HUMAN	Inverted formin-2	67	135540	5.26	7	3	6	3	6	0.11	0.1
KCD12_HUMAN	BTB/POZ domain-containing protein KCTD12	67	35679	5.51	4	2	4	2	4	0.25	0.26
RBGPR_HUMAN	Rab3 GTPase-activating protein non-catalytic subunit	67	155886	5.4	9	3	9	3	9	0.13	0.08
KAD2_HUMAN	Adenylate kinase 2, mitochondrial	67	26461	7.67	3	2	2	1	2	0.16	0.17
HUWE1_HUMAN	E3 ubiquitin-protein ligase HUWE1	67	481589	5.1	19	4	15	3	15	0.07	0.03
MP2K3_HUMAN	Dual specificity mitogen-activated protein kinase kinase 3	66	39293	7.05	5	2	4	1	4	0.21	0.11
PPIL4_HUMAN	Peptidyl-prolyl cis-trans isomerase-like 4	66	57189	5.63	3	2	2	2	2	0.11	0.16
VPS25_HUMAN	Vacuolar protein-sorting-associated protein 25	66	20735	5.97	3	2	2	2	2	0.28	0.49
NDUAA_HUMAN	NADH dehydrogenase [ubiquinone] 1 alpha subcomplex subunit 10, mitochondrial	66	40725	8.67	4	3	3	3	3	0.16	0.36
DBLOH_HUMAN	Diablo homolog, mitochondrial	66	27114	5.68	3	2	1	1	1	0.07	0.17
SEC63_HUMAN	Translocation protein SEC63 homolog	66	87942	5.21	6	4	4	2	4	0.1	0.1
TSPO_HUMAN	Translocator protein	66	18816	9.43	5	4	2	2	2	0.29	0.55
DNJB4_HUMAN	DnaJ homolog subfamily B member 4	66	37783	8.65	9	4	5	3	5	0.23	0.39
PLXB2_HUMAN	Plexin-B2	65	204997	5.85	6	2	6	2	6	0.08	0.04
ACSL3_HUMAN	Long-chain-fatty-acid--CoA ligase 3	65	80368	8.65	3	3	2	2	2	0.07	0.11
VASN_HUMAN	Vasorin	65	71668	7.16	1	1	1	1	1	0.02	0.06
PTGR1_HUMAN	Prostaglandin reductase 1	65	35847	8.45	6	3	4	3	4	0.3	0.42
NAGAB_HUMAN	Alpha-N-acetylgalactosaminidase	65	46534	4.98	3	2	2	1	2	0.1	0.09
ARFG1_HUMAN	ADP-ribosylation factor GTPase-activating protein 1	65	44640	5.46	5	3	3	3	3	0.15	0.32
TMED7_HUMAN	Transmembrane emp24 domain-containing protein 7	65	25156	6.43	13	7	8	4	8	0.6	0.93
GPDM_HUMAN	Glycerol-3-phosphate dehydrogenase, mitochondrial	64	80802	7.57	10	2	7	2	7	0.19	0.11
LIPB1_HUMAN	Liprin-beta-1	64	113952	5.4	9	3	7	3	7	0.13	0.12
LTOR3_HUMAN	Ragulator complex protein LAMTOR3	64	13614	6.73	4	2	3	2	3	0.54	0.82
TBCB_HUMAN	Tubulin-folding cofactor B	64	27308	5.06	4	3	3	2	3	0.25	0.35
FA98B_HUMAN	Protein FAM98B	64	37167	5.96	3	2	3	2	2	0.17	0.25
IF2P_HUMAN	Eukaryotic translation initiation factor 5B	64	138742	5.39	7	3	5	1	5	0.09	0.03
GBP1_HUMAN	Guanylate-binding protein 1	64	67888	5.97	4	2	4	2	4	0.13	0.13
SP16H_HUMAN	FACT complex subunit SPT16	64	119838	5.5	6	3	4	1	4	0.07	0.04
SSBP_HUMAN	Single-stranded DNA-binding protein, mitochondrial	64	17249	9.59	3	2	3	2	3	0.32	0.61
OSTF1_HUMAN	Osteoclast-stimulating factor 1	64	23772	5.46	1	1	1	1	1	0.06	0.19
S23IP_HUMAN	SEC23-interacting protein	63	111007	5.35	4	3	3	2	3	0.06	0.08
IFT27_HUMAN	Intraflagellar transport protein 27 homolog	63	20467	5.28	2	1	2	1	2	0.21	0.22
PUR8_HUMAN	Adenylosuccinate lyase	63	54854	6.69	11	4	6	3	6	0.25	0.26
UFL1_HUMAN	E3 UFM1-protein ligase 1	63	89540	6.35	7	3	6	2	6	0.17	0.1
RAVR1_HUMAN	Ribonucleoprotein PTB-binding 1	63	63837	8.79	3	2	1	1	1	0.05	0.14
THIL_HUMAN	Acetyl-CoA acetyltransferase, mitochondrial	63	45171	8.98	2	1	1	1	1	0.04	0.1
DC1L2_HUMAN	Cytoplasmic dynein 1 light intermediate chain 2	63	54066	5.97	6	2	5	2	5	0.14	0.17
SEM7A_HUMAN	Semaphorin-7A	63	74776	7.57	2	1	1	1	1	0.05	0.06
GPC6_HUMAN	Glypican-6	63	62695	5.29	6	2	5	2	5	0.16	0.14
LMNA_HUMAN	Prelamin-A/C	63	74095	6.57	7	3	6	3	6	0.17	0.18
LMF2_HUMAN	Lipase maturation factor 2	63	79647	10.1	4	3	3	2	3	0.08	0.11
STAT2_HUMAN	Signal transducer and activator of transcription 2	63	97855	5.34	6	2	5	1	5	0.09	0.04
RL14_HUMAN	60S ribosomal protein L14	62	23417	10.94	11	5	6	3	6	0.28	0.7
CD9_HUMAN	CD9 antigen	62	25399	6.8	3	2	2	1	2	0.18	0.18
NIBAN_HUMAN	Protein Niban	62	103070	4.74	9	3	8	3	8	0.16	0.13
GORS2_HUMAN	Golgi reassembly-stacking protein 2	62	47116	4.73	7	3	6	3	6	0.25	0.3
SE1L1_HUMAN	Protein sel-1 homolog 1	62	88698	5.23	3	2	2	2	2	0.06	0.1
TIGAR_HUMAN	Fructose-2,6-bisphosphatase TIGAR	62	30043	7.6	5	3	3	2	3	0.24	0.32
MPPA_HUMAN	Mitochondrial-processing peptidase subunit alpha	61	58216	6.45	6	3	4	1	4	0.12	0.07
UBA7_HUMAN	Ubiquitin-like modifier-activating enzyme 7	61	111624	5.64	2	2	1	1	1	0.03	0.04
AP3D1_HUMAN	AP-3 complex subunit delta-1	61	130076	8.69	10	2	8	2	8	0.11	0.07
TSP2_HUMAN	Thrombospondin-2	61	129908	4.62	13	4	9	2	7	0.12	0.07
BTF3_HUMAN	Transcription factor BTF3	61	22154	9.41	7	3	4	1	4	0.34	0.2
LONM_HUMAN	Lon protease homolog, mitochondrial	61	106422	6.01	6	3	4	1	4	0.08	0.04
FRIL_HUMAN	Ferritin light chain	61	20007	5.51	7	2	5	2	5	0.43	0.51
STAM1_HUMAN	Signal transducing adapter molecule 1	60	59142	4.7	6	1	4	1	4	0.14	0.07
BIEA_HUMAN	Biliverdin reductase A	60	33407	6.06	7	2	5	2	5	0.34	0.28
SNAA_HUMAN	Alpha-soluble NSF attachment protein	60	33211	5.23	6	5	4	4	4	0.2	0.65
APMAP_HUMAN	Adipocyte plasma membrane-associated protein	60	46451	5.82	9	3	6	2	6	0.25	0.2
ACON_HUMAN	Aconitate hydratase, mitochondrial	60	85372	7.36	3	1	3	1	3	0.08	0.05
ARFG3_HUMAN	ADP-ribosylation factor GTPase-activating protein 3	60	56893	7.04	4	2	4	2	4	0.13	0.16
GSH0_HUMAN	Glutamate--cysteine ligase regulatory subunit	60	30708	5.69	2	1	2	1	2	0.18	0.14
FOCAD_HUMAN	Focadhesin	60	199944	6.17	7	3	6	2	6	0.07	0.04
TTC37_HUMAN	Tetratricopeptide repeat protein 37	60	175375	7.47	10	1	9	1	9	0.1	0.02
PSMD8_HUMAN	26S proteasome non-ATPase regulatory subunit 8	60	39587	9.73	9	2	7	2	7	0.33	0.23
PREP_HUMAN	Presequence protease, mitochondrial	60	117338	6.45	9	1	8	1	8	0.13	0.04
CETN2_HUMAN	Centrin-2	60	19726	4.91	5	1	4	1	4	0.45	0.23
PSMD6_HUMAN	26S proteasome non-ATPase regulatory subunit 6	60	45502	5.45	4	2	4	2	4	0.14	0.2
ACADM_HUMAN	Medium-chain specific acyl-CoA dehydrogenase, mitochondrial	60	46559	8.61	3	2	2	1	2	0.13	0.09
PHOCN_HUMAN	MOB-like protein phocein	60	26016	5.5	6	3	4	2	4	0.32	0.38
ECI1_HUMAN	Enoyl-CoA delta isomerase 1, mitochondrial	60	32795	8.8	5	2	3	2	3	0.23	0.29
APEX1_HUMAN	DNA-(apurinic or apyrimidinic site) lyase	60	35532	8.33	7	1	6	1	6	0.26	0.12
CLIP1_HUMAN	CAP-Gly domain-containing linker protein 1	59	162147	5.29	14	3	11	2	11	0.13	0.05
GREM1_HUMAN	Gremlin-1	58	20683	9.53	3	3	2	2	2	0.17	0.49
PPID_HUMAN	Peptidyl-prolyl cis-trans isomerase D	59	40738	6.77	2	1	2	1	2	0.07	0.11
ATP5J_HUMAN	ATP synthase-coupling factor 6, mitochondrial	59	12580	9.52	3	2	3	2	3	0.41	0.91
WDFY1_HUMAN	WD repeat and FYVE domain-containing protein 1	53	46293	6.99	2	2	2	2	2	0.13	0.2
NRP1_HUMAN	Neuropilin-1	59	103069	5.58	2	1	2	1	2	0.04	0.04

[a]Protein score is calculated from the score of the peptide attributed to the protein.
[b]pI is (Predicted) isoelectric point.
[c]Number of matches is spectrum number matched to protein[#1].
[d]Number of significant matches is spectrum number that matches protein and exceeds the identification criteria.
[e]Number of sequences is number of peptides matched to protein[#2].
[f]Number of significant sequences is number of peptides exceeding the identification criteria matched to proteins.
[g]Number of unique sequences is a unique[#3] number of peptides matched to proteins.
[h]Sequence coverage is the ratio of the total number of matched peptide residues to the total length of the protein.
[i]Exponentially Modified Protein Abundance Index (http://www.matrixscience.com/help/quant_empai_help.html).

Table S2. Cont 8.

UniProt/SWISS-PROT ID	Description	Protein score[a]	Protein mass (kDa)	pI[b]	Num. of matches[c]	Num. of significant matches[d]	Num. of sequences[e]	Num. of significant sequences[f]	Num. of unique sequences[g]	Sequence coverage[h]	emPAI[i]
TSN4_HUMAN	Tetraspanin-4	59	26100	6.07	2	1	2	1	2	0.15	0.17
IBP7_HUMAN	Insulin-like growth factor-binding protein 7	59	29111	8.25	9	3	5	3	5	0.29	0.53
ADK_HUMAN	Adenosine kinase	59	40520	6.24	5	2	4	2	4	0.2	0.23
DUS3_HUMAN	Dual specificity protein phosphatase 3	59	20465	7.66	2	1	2	1	2	0.1	0.22
RECQ1_HUMAN	ATP-dependent DNA helicase Q1	59	73410	8.12	14	4	8	2	8	0.27	0.12
2A5D_HUMAN	Serine/threonine-protein phosphatase 2A 56 kDa regulatory subunit delta isoform	59	69947	8.24	3	2	2	1	2	0.06	0.06
THIM_HUMAN	3-ketoacyl-CoA thiolase, mitochondrial	58	41898	8.32	9	4	8	3	1	0.34	0.35
STX4_HUMAN	Syntaxin-4	58	34159	5.92	4	2	3	1	3	0.2	0.13
UB2R2_HUMAN	Ubiquitin-conjugating enzyme E2 R2	58	27149	4.26	6	3	3	1	3	0.34	0.36
PSB2_HUMAN	Proteasome subunit beta type-2	58	22822	6.51	15	3	6	3	6	0.36	0.72
SCMC1_HUMAN	Calcium-binding mitochondrial carrier protein SCaMC-1	57	53320	6	5	2	5	2	5	0.21	0.17
DCTN4_HUMAN	Dynactin subunit 4	57	52304	7.1	1	1	1	1	1	0.08	0.08
VMP1_HUMAN	Vacuole membrane protein 1	57	46208	6.47	2	2	1	1	1	0.07	0.09
AP1G1_HUMAN	AP-1 complex subunit gamma-1	57	91293	6.36	8	3	7	3	7	0.17	0.15
NPC1_HUMAN	Niemann-Pick C1 protein	57	142074	5.17	5	1	3	1	3	0.05	0.03
TM9S2_HUMAN	Transmembrane 9 superfamily member 2	57	75725	7.23	5	2	3	1	3	0.08	0.06
FKBP7_HUMAN	Peptidyl-prolyl cis-trans isomerase FKBP7	57	29990	6.09	4	3	4	3	4	0.22	0.51
VASP_HUMAN	Vasodilator-stimulated phosphoprotein	56	39805	9.05	13	3	8	3	8	0.24	0.37
AIFM1_HUMAN	Apoptosis-inducing factor 1, mitochondrial	56	66859	9.04	3	1	3	1	3	0.09	0.06
COX1_HUMAN	Cytochrome c oxidase subunit 1	56	57003	6.19	3	3	2	2	2	0.06	0.16
HIG2A_HUMAN	HIG1 domain family member 2A, mitochondrial	56	11521	10.21	3	3	1	1	1	0.2	0.42
NXN_HUMAN	Nucleoredoxin	56	48362	4.88	1	1	1	1	1	0.06	0.09
CSDE1_HUMAN	Cold shock domain-containing protein E1	56	88829	5.88	5	2	4	2	4	0.09	0.1
DERL2_HUMAN	Derlin-2	56	27549	6.73	2	2	1	1	1	0.13	0.16
COMD2_HUMAN	COMM domain-containing protein 2	56	22731	6.24	7	2	3	2	3	0.31	0.44
ERGI3_HUMAN	Endoplasmic reticulum-Golgi intermediate compartment protein 3	56	43194	5.68	4	2	2	1	2	0.09	0.1
QCR6_HUMAN	Cytochrome b-c1 complex subunit 6, mitochondrial	56	10732	4.39	2	2	1	1	1	0.2	0.46
MCFD2_HUMAN	Multiple coagulation factor deficiency protein 2	55	16380	4.51	1	1	1	1	1	0.16	0.28
RFIP5_HUMAN	Rab11 family-interacting protein 5	55	70372	9.28	4	3	3	2	3	0.1	0.13
NUCB2_HUMAN	Nucleobindin-2	55	50164	5.03	7	2	4	1	4	0.16	0.09
PLD3_HUMAN	Phospholipase D3	55	54671	6.02	5	1	4	1	4	0.21	0.08
TSNAX_HUMAN	Translin-associated protein X	55	33092	6.1	5	2	4	1	4	0.21	0.13
CYB5B_HUMAN	Cytochrome b5 type B	54	16322	4.88	2	1	2	1	2	0.29	0.29
NPC2_HUMAN	Epididymal secretory protein E1	54	16559	7.56	6	3	2	2	2	0.26	0.64
TPR_HUMAN	Nucleoprotein TPR	54	267131	4.97	14	2	13	2	13	0.1	0.03
ARSA_HUMAN	Arylsulfatase A	54	53554	5.65	1	1	1	1	1	0.03	0.08
RAI14_HUMAN	Ankycorbin	54	109973	5.87	6	1	5	1	5	0.08	0.04
XPO7_HUMAN	Exportin-7	54	123828	5.91	7	1	5	1	5	0.06	0.03
OSGEP_HUMAN	Probable tRNA N6-adenosine threonylcarbamoyltransferase	54	36403	5.94	1	1	1	1	1	0.08	0.12
MFGM_HUMAN	Lactadherin	54	43095	8.47	11	3	9	3	9	0.41	0.34
AT131_HUMAN	Manganese-transporting ATPase 13A1	54	132870	8.46	12	1	6	1	6	0.07	0.03
NUDC_HUMAN	Nuclear migration protein nudC	54	38219	5.27	4	2	4	2	4	0.15	0.24
TXLNA_HUMAN	Alpha-taxilin	54	61853	6.15	8	2	6	2	6	0.18	0.14
PRAF3_HUMAN	PRA1 family protein 3	54	21600	9.77	2	1	2	1	2	0.14	0.21
TR112_HUMAN	Multifunctional methyltransferase subunit TRM112-like protein	54	14190	5.21	3	1	2	1	2	0.21	0.33
5NTC_HUMAN	Cytosolic purine 5'-nucleotidase	53	64928	5.75	6	4	5	4	5	0.19	0.29
NU4M_HUMAN	NADH-ubiquinone oxidoreductase chain 4	53	51547	9.4	2	1	2	1	2	0.09	0.08
ARP5L_HUMAN	Actin-related protein 2/3 complex subunit 5-like protein	53	16931	6.15	2	2	2	2	2	0.22	0.62
TTL12_HUMAN	Tubulin--tyrosine ligase-like protein 12	53	74356	5.33	4	3	4	3	4	0.15	0.18
ISOC2_HUMAN	Isochorismatase domain-containing protein 2	53	22323	7.67	6	2	5	2	5	0.48	0.45
FPPS_HUMAN	Farnesyl pyrophosphate synthase	53	48245	5.83	5	3	3	2	3	0.17	0.19
MIC13_HUMAN	MICOS complex subunit MIC13	53	13079	9.44	4	2	2	2	2	0.44	0.86
SF01_HUMAN	Splicing factor 1	53	68286	9.07	6	1	4	1	4	0.1	0.06
SAP3_HUMAN	Ganglioside GM2 activator	52	20825	5.17	3	2	2	2	2	0.18	0.48
USP9X_HUMAN	Probable ubiquitin carboxyl-terminal hydrolase FAF-X	52	292094	5.52	15	3	14	3	14	0.12	0.04
PEBB_HUMAN	Core-binding factor subunit beta	52	21495	6.23	3	1	3	1	3	0.41	0.21
CUL5_HUMAN	Cullin-5	52	90897	8.08	8	1	8	1	8	0.17	0.05
PLIN2_HUMAN	Perilipin-2	52	48045	6.34	7	2	6	2	6	0.26	0.19
DPP3_HUMAN	Dipeptidyl peptidase 3	52	82538	5.02	9	2	8	2	8	0.23	0.11
MVD1_HUMAN	Diphosphomevalonate decarboxylase	52	43377	6.79	4	3	4	3	4	0.26	0.33
ORN_HUMAN	Oligoribonuclease, mitochondrial	52	26816	6.41	6	3	6	3	6	0.49	0.59
MLEC_HUMAN	Malectin	51	32214	5.27	2	1	1	1	1	0.08	0.14
TCP4_HUMAN	Activated RNA polymerase II transcriptional coactivator p15	51	14386	9.6	5	3	3	2	3	0.24	0.76
S10AD_HUMAN	Protein S100-A13	51	11464	5.91	10	3	5	1	5	0.39	0.43
UBS3B_HUMAN	Ubiquitin-associated and SH3 domain-containing protein B	51	72649	6.48	2	2	1	1	1	0.03	0.06
TBK1_HUMAN	Serine/threonine-protein kinase TBK1	51	83589	6.32	5	1	4	1	4	0.08	0.05
RIR1_HUMAN	Ribonucleoside-diphosphate reductase large subunit	51	90013	6.76	5	1	5	1	5	0.15	0.05
DHE3_HUMAN	Glutamate dehydrogenase 1, mitochondrial	50	61359	7.66	8	2	6	2	6	0.21	0.15
STXB3_HUMAN	Syntaxin-binding protein 3	50	67721	7.98	4	2	3	1	3	0.09	0.06
TM9S3_HUMAN	Transmembrane 9 superfamily member 3	50	67843	6.83	2	1	2	1	2	0.08	0.06
PTGES_HUMAN	Prostaglandin E synthase	50	17091	9.59	2	2	1	1	1	0.16	0.27
DGKA_HUMAN	Diacylglycerol kinase alpha	49	82577	6.28	5	2	4	1	4	0.09	0.05
CSN3_HUMAN	COP9 signalosome complex subunit 3	49	47842	6.19	6	2	6	2	6	0.27	0.19
FKBP4_HUMAN	Peptidyl-prolyl cis-trans isomerase FKBP4	49	51772	5.35	8	2	4	2	4	0.17	0.17
ODO2_HUMAN	Dihydrolipoyllysine-residue succinyltransferase component of 2-oxoglutarate dehydrogenase complex, mitochondrial	49	48724	9.11	3	1	3	1	3	0.11	0.09
PDIA5_HUMAN	Protein disulfide-isomerase A5	49	59556	8.08	2	1	2	1	2	0.11	0.07
ADPGK_HUMAN	ADP-dependent glucokinase	49	54055	5.77	3	3	2	2	2	0.08	0.17
SATT_HUMAN	Neutral amino acid transporter A	49	55688	5.88	4	3	2	2	2	0.1	0.16
COX2_HUMAN	Cytochrome c oxidase subunit 2	49	25548	4.67	5	3	2	1	2	0.13	0.38
NU155_HUMAN	Nuclear pore complex protein Nup155	49	155100	5.78	6	2	5	1	5	0.1	0.03
PAXI_HUMAN	Paxillin	49	64464	5.8	8	2	6	1	6	0.24	0.14
SPNS1_HUMAN	Protein spinster homolog 1	49	56594	6.19	3	2	3	2	3	0.13	0.16
RIR2B_HUMAN	Ribonucleoside-diphosphate reductase subunit M2 B	48	40710	4.89	1	1	1	1	1	0.07	0.11
TMED5_HUMAN	Transmembrane emp24 domain-containing protein 5	48	25988	4.71	1	1	1	1	1	0.12	0.17
ARPIN_HUMAN	Arpin	48	24927	5.57	2	2	1	1	1	0.12	0.18
LYPA1_HUMAN	Acyl-protein thioesterase 1	48	24653	6.29	2	1	2	1	2	0.18	0.18
HIBCH_HUMAN	3-hydroxyisobutyryl-CoA hydrolase, mitochondrial	48	43454	8.38	4	2	3	1	3	0.16	0.1
DHX15_HUMAN	Pre-mRNA-splicing factor ATP-dependent RNA helicase DHX15	48	90875	7.12	5	3	4	2	4	0.09	0.1
MXRA8_HUMAN	Matrix remodeling-associated protein 8	47	49101	6.75	1	1	1	1	1	0.04	0.09
LSM2_HUMAN	U6 snRNA-associated Sm-like protein LSm2	47	10828	6.04	2	1	2	1	2	0.41	0.45
CP062_HUMAN	UPF0505 protein C16orf62	47	109492	6.82	6	2	6	2	6	0.12	0.08
SYFB_HUMAN	Phenylalanine--tRNA ligase beta subunit	39	66074	6.4	8	3	4	3	1	0.15	0.21
QOR_HUMAN	Quinone oxidoreductase	47	35185	8.56	2	1	2	1	2	0.12	0.13
MICA1_HUMAN	[F-actin]-monooxygenase MICAL1	47	117801	6	6	1	5	1	5	0.12	0.04
G6PE_HUMAN	GDH/6PGL endoplasmic bifunctional protein	47	88836	6.84	6	1	4	1	4	0.08	0.05
DDX17_HUMAN	Probable ATP-dependent RNA helicase DDX17	47	80222	8.53	5	1	5	1	4	0.11	0.05
CHIP_HUMAN	E3 ubiquitin-protein ligase CHIP	47	34834	5.61	3	1	3	1	3	0.17	0.13
1A03_HUMAN	HLA class I histocompatibility antigen, A-3 alpha chain	47	40815	5.66	5	2	5	2	5	0.25	0.23
CRIP2_HUMAN	Cysteine-rich protein 2	47	22478	9.01	1	1	1	1	1	0.08	0.2
TYPH_HUMAN	Thymidine phosphorylase	46	49924	5.36	6	3	4	2	4	0.18	0.18

[a]Protein score is calculated from the score of the peptide attributed to the protein.
[b]pI is (Predicted) isoelectric point.
[c]Number of matches is spectrum number matched to protein[#1].
[d]Number of significant matches is spectrum number that matches protein and exceeds the identification criteria.
[e]Number of sequences is number of peptides matched to protein[#2].
[f]Number of significant sequences is number of peptides exceeding the identification criteria matched to proteins.
[g]Number of unique sequences is a unique[#3] number of peptides matched to proteins.
[h]Sequence coverage is the ratio of the total number of matched peptide residues to the total length of the protein.
[i]Exponentially Modified Protein Abundance Index (http://www.matrixscience.com/help/quant_empai_help.html).

Table S2. Cont 9.

UniProt/SWISS-PROT ID	Description	Protein score[a]	Protein mass (kDa)	pI[b]	Num. of matches[c]	Num. of significant matches[d]	Num. of sequences[e]	Num. of significant sequences[f]	Num. of unique sequences[g]	Sequence coverage[h]	emPAI[i]
ARHG2_HUMAN	Rho guanine nucleotide exchange factor 2	46	111473	6.89	1	1	1	1	1	0.02	0.04
VPS4A_HUMAN	Vacuolar protein sorting-associated protein 4A	46	48867	7.64	5	3	3	2	3	0.15	0.19
RS27L_HUMAN	40S ribosomal protein S27-like	46	9471	9.57	2	1	2	1	2	0.3	0.53
EXOS5_HUMAN	Exosome complex component RRP46	46	25233	7.53	1	1	1	1	1	0.1	0.18
RIPK1_HUMAN	Receptor-interacting serine/threonine-protein kinase 1	46	75883	5.92	4	1	3	1	3	0.08	0.06
SKIV2_HUMAN	Helicase SKI2W	46	137668	5.72	4	1	3	1	3	0.05	0.03
IMA5_HUMAN	Importin subunit alpha-5	46	60184	4.94	3	2	3	2	2	0.14	0.15
NEK7_HUMAN	Serine/threonine-protein kinase Nek7	46	34528	8.49	7	3	6	3	6	0.36	0.43
RRF2M_HUMAN	Ribosome-releasing factor 2, mitochondrial	46	86546	6.08	2	1	2	1	2	0.06	0.05
AMRP_HUMAN	Alpha-2-macroglobulin receptor-associated protein	46	41441	8.73	5	3	3	2	3	0.12	0.22
MIC19_HUMAN	MICOS complex subunit MIC19	46	26136	8.48	6	1	2	1	2	0.09	0.17
YAP1_HUMAN	Transcriptional coactivator YAP1	46	54427	5	4	1	3	1	3	0.15	0.08
FKBP8_HUMAN	Peptidyl-prolyl cis-trans isomerase FKBP8	46	44534	4.78	8	3	4	1	4	0.21	0.1
RANG_HUMAN	Ran-specific GTPase-activating protein	45	23296	5.19	5	2	4	1	4	0.26	0.19
NSF1C_HUMAN	NSFL1 cofactor p47	45	40548	4.99	5	2	4	2	4	0.18	0.23
NUDC1_HUMAN	NudC domain-containing protein 1	45	66713	4.99	5	1	5	1	5	0.14	0.06
ACDSB_HUMAN	Short/branched chain specific acyl-CoA dehydrogenase, mitochondrial	45	47455	6.53	3	1	2	1	2	0.15	0.09
C1TM_HUMAN	Monofunctional C1-tetrahydrofolate synthase, mitochondrial	45	105724	8.32	3	1	2	1	2	0.04	0.04
CD59_HUMAN	CD59 glycoprotein	45	14168	6.02	6	2	3	2	3	0.49	0.78
WASC4_HUMAN	WASH complex subunit 4	45	136316	7.1	6	1	5	1	5	0.08	0.03
UPAR_HUMAN	Urokinase plasminogen activator surface receptor	45	36953	6.2	5	2	4	1	4	0.3	0.12
RABX5_HUMAN	Rab5 GDP/GTP exchange factor	45	79321	6.42	8	2	6	1	6	0.17	0.05
PLBL2_HUMAN	Putative phospholipase B-like 2	45	65430	6.34	5	2	3	2	3	0.08	0.14
LYRIC_HUMAN	Protein LYRIC	45	63799	9.33	5	3	3	3	3	0.15	0.22
TPBG_HUMAN	Trophoblast glycoprotein	45	46003	6.35	3	1	2	1	2	0.06	0.09
PPM1F_HUMAN	Protein phosphatase 1F	44	49800	4.99	3	1	3	1	3	0.1	0.09
FKB11_HUMAN	Peptidyl-prolyl cis-trans isomerase FKBP11	44	22166	9.44	2	1	2	1	2	0.14	0.2
NUP62_HUMAN	Nuclear pore glycoprotein p62	44	53222	5.21	2	1	2	1	2	0.08	0.08
CLP1L_HUMAN	Cleft lip and palate transmembrane protein 1-like protein	39	62189	8.71	3	3	2	2	2	0.07	0.14
LC7L2_HUMAN	Putative RNA-binding protein Luc7-like 2	44	46486	10.02	4	1	4	1	4	0.19	0.09
SPRE_HUMAN	Sepiapterin reductase	44	28031	8.24	1	1	1	1	1	0.08	0.16
CK5P3_HUMAN	CDK5 regulatory subunit-associated protein 3	44	56885	4.68	4	2	2	1	2	0.09	0.08
HM13_HUMAN	Minor histocompatibility antigen H13	44	41462	6.01	3	1	2	1	2	0.13	0.11
DAD1_HUMAN	Dolichyl-diphosphooligosaccharide--protein glycosyltransferase subunit DAD1	44	12489	6.52	2	2	1	1	1	0.11	0.38
RAB3B_HUMAN	Ras-related protein Rab-3B	44	24742	4.85	7	1	2	1	1	0.13	0.18
PTPA_HUMAN	Serine/threonine-protein phosphatase 2A activator	44	40641	5.63	4	2	3	2	3	0.2	0.23
MAVS_HUMAN	Mitochondrial antiviral-signaling protein	44	56493	5.36	2	1	2	1	2	0.11	0.08
SCUB3_HUMAN	Signal peptide, CUB and EGF-like domain-containing protein 3	44	109210	7.85	4	2	3	1	3	0.06	0.04
EFHD2_HUMAN	EF-hand domain-containing protein D2	44	26680	5.15	2	1	2	1	2	0.12	0.17
SCPDL_HUMAN	Saccharopine dehydrogenase-like oxidoreductase	44	47121	9.24	4	3	2	2	2	0.13	0.19
TM9S4_HUMAN	Transmembrane 9 superfamily member 4	44	74470	6.1	7	1	5	1	5	0.16	0.06
NONO_HUMAN	Non-POU domain-containing octamer-binding protein	44	54197	9.01	5	1	4	1	4	0.21	0.08
ARK72_HUMAN	Aflatoxin B1 aldehyde reductase member 2	43	39564	6.7	4	1	3	1	3	0.18	0.11
NU5M_HUMAN	NADH-ubiquinone oxidoreductase chain 5	43	66982	9.14	2	1	2	1	2	0.09	0.06
AMPD2_HUMAN	AMP deaminase 2	43	100624	6.46	3	1	2	1	2	0.04	0.04
IPYR2_HUMAN	Inorganic pyrophosphatase 2, mitochondrial	43	37896	7.07	2	1	2	1	2	0.14	0.12
TFIP8_HUMAN	Tumor necrosis factor alpha-induced protein 8	43	22989	7.74	4	1	3	1	3	0.3	0.2
MTX2_HUMAN	Metaxin-2	43	29744	5.9	4	2	2	1	2	0.19	0.15
TMED3_HUMAN	Transmembrane emp24 domain-containing protein 3	43	24761	5.4	7	1	3	1	3	0.3	0.18
PTN23_HUMAN	Tyrosine-protein phosphatase non-receptor type 23	43	178861	6.45	1	1	1	1	1	0.01	0.02
RAB35_HUMAN	Ras-related protein Rab-35	43	23011	8.52	10	1	3	1	2	0.2	0.2
SYFA_HUMAN	Phenylalanine--tRNA ligase alpha subunit	43	57528	7.31	3	1	3	1	3	0.13	0.08
FAHD1_HUMAN	Acylpyruvase FAHD1, mitochondrial	43	24827	6.96	3	3	2	2	2	0.25	0.39
RBP2_HUMAN	E3 SUMO-protein ligase RanBP2	43	357974	5.85	17	1	15	1	15	0.09	0.01
MBOA7_HUMAN	Lysophospholipid acyltransferase 7	43	52730	9.17	4	1	3	1	3	0.09	0.08
RHG17_HUMAN	Rho GTPase-activating protein 17	43	95377	7.23	3	1	3	1	3	0.07	0.04
ARMT1_HUMAN	Protein-glutamate O-methyltransferase	42	51140	5.48	3	1	3	1	3	0.13	0.08
CATA_HUMAN	Catalase	42	59719	6.9	5	1	4	1	4	0.13	0.07
TB182_HUMAN	182 kDa tankyrase-1-binding protein	42	181685	4.77	6	1	3	1	3	0.02	0.02
DCUP_HUMAN	Uroporphyrinogen decarboxylase	42	40761	5.77	3	2	2	2	2	0.15	0.23
BDH2_HUMAN	3-hydroxybutyrate dehydrogenase type 2	42	26707	7.56	3	1	3	1	3	0.21	0.17
PLXD1_HUMAN	Plexin-D1	42	211871	6.8	5	1	4	1	4	0.04	0.02
HNRLL_HUMAN	Heterogeneous nuclear ribonucleoprotein L-like	42	60045	7.83	2	2	2	2	2	0.08	0.15
HERC4_HUMAN	Probable E3 ubiquitin-protein ligase HERC4	41	118487	5.8	9	3	6	2	6	0.14	0.07
VPS51_HUMAN	Vacuolar protein sorting-associated protein 51 homolog	41	85988	6.06	2	1	2	1	2	0.06	0.05
PHS_HUMAN	Pterin-4-alpha-carbinolamine dehydratase	41	11992	6.28	3	1	3	1	3	0.41	0.4
RECK_HUMAN	Reversion-inducing cysteine-rich protein with Kazal motifs	41	106386	6.35	9	1	7	1	7	0.17	0.04
CA123_HUMAN	UPF0587 protein C1orf123	41	18037	4.93	2	1	2	1	2	0.17	0.26
IMPA1_HUMAN	Inositol monophosphatase 1	41	30169	5.16	3	1	3	1	3	0.27	0.15
SNX2_HUMAN	Sorting nexin-2	41	58435	5.04	8	2	5	2	5	0.17	0.15
TF_HUMAN	Tissue factor	41	33047	6.64	4	1	3	1	3	0.18	0.13
HIF1N_HUMAN	Hypoxia-inducible factor 1-alpha inhibitor	41	40260	5.39	1	1	1	1	1	0.07	0.11
METK2_HUMAN	S-adenosylmethionine synthase isoform type-2	41	43633	6.02	7	1	5	1	5	0.21	0.1
BAG3_HUMAN	BAG family molecular chaperone regulator 3	40	61557	6.46	3	2	3	2	3	0.13	0.15
UBE2Z_HUMAN	Ubiquitin-conjugating enzyme E2 Z	40	38186	5.37	2	1	2	1	2	0.11	0.11
ABHEB_HUMAN	Protein ABHD14B	40	22332	5.94	3	2	2	2	2	0.15	0.45
KAP2_HUMAN	cAMP-dependent protein kinase type II-alpha regulatory subunit	40	45490	4.96	2	1	2	1	2	0.07	0.1
UBFD1_HUMAN	Ubiquitin domain-containing protein UBFD1	40	33361	5.55	4	2	3	1	3	0.17	0.13
OSBL8_HUMAN	Oxysterol-binding protein-related protein 8	40	101132	6.52	10	1	8	1	8	0.14	0.04
PARPT_HUMAN	TCDD-inducible poly [ADP-ribose] polymerase	40	76178	6.19	69	1	3	1	3	0.08	0.06
ARMX2_HUMAN	Armadillo repeat-containing X-linked protein 2	40	65643	8.68	2	1	2	1	2	0.06	0.07
GLRX3_HUMAN	Glutaredoxin-3	40	37408	5.31	7	2	5	1	5	0.26	0.12
CNRP1_HUMAN	CB1 cannabinoid receptor-interacting protein 1	40	18637	7.72	4	1	4	1	4	0.53	0.25
BID_HUMAN	BH3-interacting domain death agonist	40	21981	5.27	4	3	2	1	2	0.25	0.21
SSRD_HUMAN	Translocon-associated protein subunit delta	40	18987	5.76	4	2	2	2	2	0.18	0.54
CAST2_HUMAN	Cytosolic arginine sensor for mTORC1 subunit 2	40	36034	5.02	1	1	1	1	1	0.11	0.12
HDHD1_HUMAN	Pseudouridine-5'-phosphatase	40	25233	5.18	1	1	1	1	1	0.08	0.18
TOLIP_HUMAN	Toll-interacting protein	39	30262	5.68	3	1	3	1	3	0.18	0.15
GSTM2_HUMAN	Glutathione S-transferase Mu 2	39	25728	6	6	2	4	1	4	0.28	0.17
FIS1_HUMAN	Mitochondrial fission 1 protein	39	16927	8.84	3	2	2	1	2	0.18	0.28
GOGA2_HUMAN	Golgin subfamily A member 2	39	113017	5	7	1	5	1	5	0.09	0.04
LZIC_HUMAN	Protein LZIC	39	21481	4.88	3	1	2	1	2	0.26	0.21
AUP1_HUMAN	Ancient ubiquitous protein 1	39	52995	8.37	2	1	2	1	2	0.09	0.08
GNPI1_HUMAN	Glucosamine-6-phosphate isomerase 1	39	32648	6.42	4	2	3	1	3	0.25	0.14
ANO10_HUMAN	Anoctamin-10	39	76280	7.1	2	1	2	1	2	0.07	0.06
LAP2A_HUMAN	Lamina-associated polypeptide 2, isoform alpha	39	75446	7.56	3	3	2	2	2	0.05	0.12
MYLK_HUMAN	Myosin light chain kinase, smooth muscle	39	210583	5.85	10	1	8	1	8	0.09	0.02
CATZ_HUMAN	Cathepsin Z	39	33846	6.7	3	1	2	1	2	0.11	0.13
HDDC2_HUMAN	HD domain-containing protein 2	39	23376	5.33	3	2	2	1	2	0.21	0.19
HPRT_HUMAN	Hypoxanthine-guanine phosphoribosyltransferase	39	24564	6.21	4	2	3	1	3	0.25	0.18

[a]Protein score is calculated from the score of the peptide attributed to the protein.
[b]pI is (Predicted) isoelectric point.
[c]Number of matches is spectrum number matched to protein[#1].
[d]Number of significant matches is spectrum number that matches protein and exceeds the identification criteria.
[e]Number of sequences is number of peptides matched to protein[#2].
[f]Number of significant sequences is number of peptides exceeding the identification criteria matched to proteins.
[g]Number of unique sequences is a unique[#3] number of peptides matched to proteins.
[h]Sequence coverage is the ratio of the total number of matched peptide residues to the total length of the protein.
[i]Exponentially Modified Protein Abundance Index (http://www.matrixscience.com/help/quant_empai_help.html).

Table S2. Cont 10.

UniProt/SWISS-PROT ID	Description	Protein score[a]	Protein mass (kDa)	pI[b]	Num. of matches[c]	Num. of significant matches[d]	Num. of sequences[e]	Num. of significant sequences[f]	Num. of unique sequences[g]	Sequence coverage[h]	emPAI[i]
BIN1_HUMAN	Myc box-dependent-interacting protein 1	39	64659	4.97	2	1	2	1	2	0.07	0.07
QCR8_HUMAN	Cytochrome b-c1 complex subunit 8	38	9900	10.07	3	3	1	1	1	0.26	0.51
HEM3_HUMAN	Porphobilinogen deaminase	38	39306	6.68	4	1	3	1	3	0.15	0.11
NT5D1_HUMAN	5'-nucleotidase domain-containing protein 1	38	51812	5.94	1	1	1	1	1	0.04	0.08
AT5F1_HUMAN	ATP synthase F(0) complex subunit B1, mitochondrial	38	28890	9.37	3	1	3	1	3	0.14	0.15
CPPED_HUMAN	Serine/threonine-protein phosphatase CPPED1	38	35526	5.78	2	1	2	1	2	0.12	0.12
RUVB1_HUMAN	RuvB-like 1	38	50196	6.02	3	2	3	2	3	0.12	0.18
CK068_HUMAN	UPF0696 protein C11orf68	38	27338	5.28	4	2	2	1	2	0.16	0.16
SUMF2_HUMAN	Sulfatase-modifying factor 2	38	33822	7.78	1	1	1	1	1	0.03	0.13
PCY2_HUMAN	Ethanolamine-phosphate cytidylyltransferase	38	43808	6.44	4	1	3	1	3	0.13	0.1
PK3C3_HUMAN	Phosphatidylinositol 3-kinase catalytic subunit type 3	38	101485	6.37	4	1	4	1	4	0.06	0.04
CATL1_HUMAN	Cathepsin L1	38	37540	5.31	2	1	2	1	2	0.11	0.12
GDE_HUMAN	Glycogen debranching enzyme	38	174652	6.31	4	2	3	2	3	0.04	0.05
CPT1A_HUMAN	Carnitine O-palmitoyltransferase 1, liver isoform	37	88311	8.85	2	1	2	1	2	0.07	0.05
PAPS1_HUMAN	Bifunctional 3'-phosphoadenosine 5'-phosphosulfate synthase 1	37	70788	6.4	7	1	6	1	6	0.22	0.06
TPPC8_HUMAN	Trafficking protein particle complex subunit 8	37	160896	6.41	6	1	4	1	4	0.05	0.03
PLAP_HUMAN	Phospholipase A-2-activating protein	37	87101	5.96	3	1	3	1	3	0.09	0.05
CLCA_HUMAN	Clathrin light chain A	37	27060	4.43	1	1	1	1	1	0.03	0.17
GMPPA_HUMAN	Mannose-1-phosphate guanyltransferase alpha	37	46262	6.73	2	2	1	1	1	0.04	0.09
CSPG4_HUMAN	Chondroitin sulfate proteoglycan 4	37	250382	5.27	4	1	4	1	4	0.04	0.02
SAHH2_HUMAN	S-adenosylhomocysteine hydrolase-like protein 1	37	58913	6.49	7	3	6	2	6	0.17	0.15
SERB_HUMAN	Phosphoserine phosphatase	37	24992	5.53	2	1	2	1	2	0.12	0.18
GIPC1_HUMAN	PDZ domain-containing protein GIPC1	37	36027	5.9	2	1	2	1	2	0.08	0.12
SUCA_HUMAN	Succinate--CoA ligase [ADP/GDP-forming] subunit alpha, mitochondrial	36	36227	9.01	3	1	2	1	2	0.12	0.12
GBF1_HUMAN	Golgi-specific brefeldin A-resistance guanine nucleotide exchange factor 1	36	206315	5.48	12	1	10	1	9	0.1	0.02
MXRA7_HUMAN	Matrix-remodeling-associated protein 7	36	21452	4.24	1	1	1	1	1	0.14	0.21
SCFD1_HUMAN	Sec1 family domain-containing protein 1	36	72334	5.89	8	5	6	5	6	0.17	0.33
K1C19_HUMAN	Keratin, type I cytoskeletal 19	36	44079	5.04	1	1	1	1	1	0.06	0.1
ALG5_HUMAN	Dolichyl-phosphate beta-glucosyltransferase	36	36922	9.34	1	1	1	1	1	0.05	0.12
OPA1_HUMAN	Dynamin-like 120 kDa protein, mitochondrial	36	111561	7.88	3	1	3	1	3	0.07	0.04
CN37_HUMAN	2',3'-cyclic-nucleotide 3'-phosphodiesterase	36	47549	9.17	3	2	2	1	2	0.09	0.09
GUAA_HUMAN	GMP synthase [glutamine-hydrolyzing]	36	76667	6.42	2	1	2	1	2	0.05	0.06
ANX13_HUMAN	Annexin A13	36	35393	5.47	3	1	2	1	2	0.09	0.12
LCAP_HUMAN	Leucyl-cystinyl aminopeptidase	36	117274	5.5	4	1	2	1	2	0.04	0.04
CUL2_HUMAN	Cullin-2	36	86927	6.45	3	1	3	1	3	0.09	0.05
SCAM1_HUMAN	Secretory carrier-associated membrane protein 1	35	37896	7.03	3	2	1	1	1	0.07	0.12
CD248_HUMAN	Endosialin	35	80807	5.18	3	2	2	2	2	0.05	0.11
UBA3_HUMAN	NEDD8-activating enzyme E1 catalytic subunit	35	51819	5.3	1	1	1	1	1	0.05	0.08
MCA3_HUMAN	Eukaryotic translation elongation factor 1 epsilon-1	35	19798	8.54	2	1	1	1	1	0.14	0.23
PTER_HUMAN	Phosphotriesterase-related protein	35	38993	6.07	1	1	1	1	1	0.06	0.11
CHCH2_HUMAN	Coiled-coil-helix-coiled-coil-helix domain-containing protein 2	35	15503	9.43	1	1	1	1	1	0.19	0.3
CD99_HUMAN	CD99 antigen	35	18836	4.58	1	1	1	1	1	0.1	0.24
EIF3J_HUMAN	Eukaryotic translation initiation factor 3 subunit J	35	29045	4.72	3	1	3	1	3	0.21	0.15
WFS1_HUMAN	Wolframin	35	100227	8.34	3	1	3	1	3	0.06	0.04
TXD12_HUMAN	Thioredoxin domain-containing protein 12	35	19194	5.24	2	1	2	1	2	0.31	0.24
ITIH2_HUMAN	Inter-alpha-trypsin inhibitor heavy chain H2	35	106397	6.4	9	1	7	1	7	0.14	0.04
ELOB_HUMAN	Elongin-B	35	13125	4.73	8	2	4	1	4	0.66	0.37
CMBL_HUMAN	Carboxymethylenebutenolidase homolog	34	28030	6.71	6	2	4	2	4	0.33	0.34
SQOR_HUMAN	Sulfide:quinone oxidoreductase, mitochondrial	34	49929	9.18	4	2	3	1	3	0.11	0.09
EI2BA_HUMAN	Translation initiation factor eIF-2B subunit alpha	34	33691	6.9	4	1	3	1	3	0.22	0.13
SPP2A_HUMAN	Signal peptide peptidase-like 2A	34	58104	8.65	1	1	1	1	1	0.02	0.07
SNX18_HUMAN	Sorting nexin-18	34	68851	5.44	2	1	2	1	2	0.07	0.06
RB22A_HUMAN	Ras-related protein Rab-22A	34	21841	8.32	2	1	2	1	2	0.15	0.21
UB2R1_HUMAN	Ubiquitin-conjugating enzyme E2 R1	34	26720	4.41	2	1	1	1	1	0.12	0.17
SQSTM_HUMAN	Sequestosome-1	34	47657	5.1	8	2	8	2	8	0.38	0.19
ACBP_HUMAN	Acyl-CoA-binding protein	34	10038	6.12	4	2	3	2	3	0.51	1.25
EWS_HUMAN	RNA-binding protein EWS	34	68436	9.37	4	1	4	1	4	0.1	0.06
SAR1B_HUMAN	GTP-binding protein SAR1b	34	22396	5.76	7	2	6	2	5	0.46	0.45
UBXN4_HUMAN	UBX domain-containing protein 4	34	56743	6.1	4	3	3	2	3	0.15	0.16
SFXN3_HUMAN	Sideroflexin-3	34	35481	9.26	6	1	4	1	4	0.24	0.12
MGST3_HUMAN	Microsomal glutathione S-transferase 3	34	16506	9.46	6	4	2	2	2	0.32	1.1
TRIPB_HUMAN	Thyroid receptor-interacting protein 11	33	227447	5.18	15	1	13	1	13	0.12	0.02
ATPG_HUMAN	ATP synthase subunit gamma, mitochondrial	33	32975	9.23	3	1	3	1	3	0.17	0.13
SGT1_HUMAN	Protein SGT1 homolog	33	40998	5.07	3	1	3	1	3	0.15	0.11
BOLA2_HUMAN	BolA-like protein 2	33	10110	6.07	4	1	3	1	3	0.51	0.49
CRKL_HUMAN	Crk-like protein	33	33756	6.26	3	1	1	1	1	0.06	0.13
STX17_HUMAN	Syntaxin-17	33	33383	6.14	3	1	2	1	2	0.15	0.13
PDLI2_HUMAN	PDZ and LIM domain protein 2	33	37436	9	2	2	1	1	1	0.06	0.12
APOB_HUMAN	Apolipoprotein B-100	33	515283	6.58	13	2	11	2	10	0.05	0.02
APOL2_HUMAN	Apolipoprotein L2	33	37069	6.28	2	1	2	1	2	0.12	0.12
SYMC_HUMAN	Methionine--tRNA ligase, cytoplasmic	33	101052	5.82	7	1	6	1	6	0.14	0.04
SNX17_HUMAN	Sorting nexin-17	33	52868	7.07	1	1	1	1	1	0.03	0.08
CDR2L_HUMAN	Cerebellar degeneration-related protein 2-like	33	52978	5.7	2	1	2	1	2	0.08	0.08
HYEP_HUMAN	Epoxide hydrolase 1	33	52915	6.77	7	2	5	2	5	0.24	0.17
ILF3_HUMAN	Interleukin enhancer-binding factor 3	32	95279	8.86	3	2	3	2	3	0.07	0.09
TBCEL_HUMAN	Tubulin-specific chaperone cofactor E-like protein	32	48164	5.23	2	1	1	1	1	0.06	0.09
CCDC6_HUMAN	Coiled-coil domain-containing protein 6	32	53258	6.87	9	2	4	1	4	0.16	0.08
MOGS_HUMAN	Mannosyl-oligosaccharide glucosidase	32	91861	8.97	5	2	5	2	5	0.14	0.1
COR1B_HUMAN	Coronin-1B	32	54200	5.61	6	2	5	2	5	0.26	0.17
WDR26_HUMAN	WD repeat-containing protein 26	32	72079	5.74	2	1	2	1	2	0.06	0.06
TM165_HUMAN	Transmembrane protein 165	32	34883	6.54	1	1	1	1	1	0.06	0.13
DJC10_HUMAN	DnaJ homolog subfamily C member 10	32	91021	6.78	6	1	6	1	6	0.14	0.05
SRP68_HUMAN	Signal recognition particle subunit SRP68	32	70686	8.75	7	1	4	1	4	0.1	0.06
ACAD9_HUMAN	Acyl-CoA dehydrogenase family member 9, mitochondrial	32	68717	8.16	2	1	2	1	2	0.07	0.06
ACPH_HUMAN	Acylamino-acid-releasing enzyme	32	81173	5.29	5	1	5	1	5	0.17	0.05
GSK3B_HUMAN	Glycogen synthase kinase-3 beta	31	46715	8.98	3	2	2	1	2	0.13	0.09
API5_HUMAN	Apoptosis inhibitor 5	31	58968	6.99	2	1	2	1	2	0.08	0.07
ACOC_HUMAN	Cytoplasmic aconitate hydratase	31	98337	6.23	6	3	2	2	2	0.06	0.09
BZW2_HUMAN	Basic leucine zipper and W2 domain-containing protein 2	31	48132	6.26	6	1	5	1	4	0.23	0.09
ENDD1_HUMAN	Endonuclease domain-containing 1 protein	31	54981	5.55	3	1	2	1	2	0.12	0.08
PDCL3_HUMAN	Phosducin-like protein 3	31	27597	4.78	3	1	3	1	3	0.26	0.16
PCLI1_HUMAN	PTB-containing, cubilin and LRP1-interacting protein	31	28253	6.53	5	1	4	1	4	0.24	0.16
MALD1_HUMAN	MARVEL domain-containing protein 1	31	18902	9.69	1	1	1	1	1	0.17	0.24
KDEL2_HUMAN	KDEL motif-containing protein 2	31	58535	8.42	4	1	4	1	4	0.11	0.07
FUBP1_HUMAN	Far upstream element-binding protein 1	31	67518	7.18	6	1	6	1	6	0.15	0.06
MOCOS_HUMAN	Molybdenum cofactor sulfurase	31	98058	6.23	2	2	1	1	1	0.03	0.04
PTH2_HUMAN	Peptidyl-tRNA hydrolase 2, mitochondrial	31	19181	8.95	3	1	2	1	2	0.16	0.24
PFD5_HUMAN	Prefoldin subunit 5	31	17317	5.93	2	1	2	1	2	0.31	0.27
SPTC1_HUMAN	Serine palmitoyltransferase 1	31	52710	5.72	4	1	4	1	4	0.14	0.08
FUBP3_HUMAN	Far upstream element-binding protein 3	31	61602	8.6	5	3	3	2	3	0.12	0.15

[a]Protein score is calculated from the score of the peptide attributed to the protein.
[b]pI is (Predicted) isoelectric point.
[c]Number of matches is spectrum number matched to protein[#1].
[d]Number of significant matches is spectrum number that matches protein and exceeds the identification criteria.
[e]Number of sequences is number of peptides matched to protein[#2].
[f]Number of significant sequences is number of peptides exceeding the identification criteria matched to proteins.
[g]Number of unique sequences is a unique[#3] number of peptides matched to proteins.
[h]Sequence coverage is the ratio of the total number of matched peptide residues to the total length of the protein.
[i]Exponentially Modified Protein Abundance Index (http://www.matrixscience.com/help/quant_empai_help.html).

Table S2. Cont 11.

UniProt/SWISS-PROT ID	Description	Protein score[a]	Protein mass (kDa)	pI[b]	Num. of matches[c]	Num. of significant matches[d]	Num. of sequences[e]	Num. of significant sequences[f]	Num. of unique sequences[g]	Sequence coverage[h]	emPAI[i]
TM214_HUMAN	Transmembrane protein 214	30	77101	9.28	3	1	3	1	3	0.1	0.06
DHB11_HUMAN	Estradiol 17-beta-dehydrogenase 11	30	32915	9.2	4	1	4	1	4	0.18	0.13
TI23B_HUMAN	Putative mitochondrial import inner membrane translocase subunit Tim23B	30	28030	9.47	2	1	2	1	2	0.21	0.16
TBL2_HUMAN	Transducin beta-like protein 2	30	49766	9.52	3	1	2	1	2	0.08	0.09
TXTP_HUMAN	Tricarboxylate transport protein, mitochondrial	30	33991	9.91	6	2	4	1	4	0.2	0.28
RHEB_HUMAN	GTP-binding protein Rheb	30	20485	5.65	2	2	1	1	1	0.14	0.22
SC16A_HUMAN	Protein transport protein Sec16A	30	233373	5.4	5	1	4	1	4	0.05	0.02
TBCD_HUMAN	Tubulin-specific chaperone D	30	132515	5.8	2	1	1	1	1	0.02	0.03
NU214_HUMAN	Nuclear pore complex protein Nup214	29	213488	7.15	4	1	3	1	3	0.03	0.02
GLIP1_HUMAN	Glioma pathogenesis-related protein 1	29	30346	8.8	1	1	1	1	1	0.09	0.15
DHRS1_HUMAN	Dehydrogenase/reductase SDR family member 1	29	33887	8.01	2	2	1	1	1	0.06	0.13
KPCA_HUMAN	Protein kinase C alpha type	29	76700	6.61	3	1	3	1	3	0.1	0.06
RRAS2_HUMAN	Ras-related protein R-Ras2	29	23385	5.74	4	1	3	1	3	0.25	0.19
MBNL1_HUMAN	Muscleblind-like protein 1	29	41790	9.16	2	1	2	1	2	0.13	0.1
ERAP1_HUMAN	Endoplasmic reticulum aminopeptidase 1	29	107166	6.02	4	1	3	1	3	0.06	0.04
ACO13_HUMAN	Acyl-coenzyme A thioesterase 13	29	14951	9.23	1	1	1	1	1	0.13	0.31
TP8L3_HUMAN	Tumor necrosis factor alpha-induced protein 8-like protein 3	29	32639	8.75	3	1	2	1	2	0.15	0.14
EMD_HUMAN	Emerin	29	28976	5.29	3	2	3	2	3	0.24	0.33
LAMB1_HUMAN	Laminin subunit beta-1	29	197909	4.83	15	2	11	2	11	0.15	0.04
ITM2B_HUMAN	Integral membrane protein 2B	29	30318	5	2	1	2	1	2	0.15	0.15
PUR4_HUMAN	Phosphoribosylformylglycinamidine synthase	29	144643	5.5	6	1	6	1	6	0.13	0.03
BAG2_HUMAN	BAG family molecular chaperone regulator 2	29	23757	6.25	5	1	4	1	4	0.34	0.19
IAH1_HUMAN	Isoamyl acetate-hydrolyzing esterase 1 homolog	29	27581	5.13	3	1	2	1	2	0.17	0.16
GCSH_HUMAN	Glycine cleavage system H protein, mitochondrial	28	18873	4.91	2	1	2	1	2	0.29	0.24
MGLL_HUMAN	Monoglyceride lipase	28	33240	6.49	3	1	2	1	2	0.13	0.13
GGH_HUMAN	Gamma-glutamyl hydrolase	28	35941	6.67	1	1	1	1	1	0.08	0.12
AN13A_HUMAN	Ankyrin repeat domain-containing protein 13A	28	67577	4.94	1	1	1	1	1	0.04	0.06
CSN8_HUMAN	COP9 signalosome complex subunit 8	28	23211	5.25	1	1	1	1	1	0.14	0.19
LPXN_HUMAN	Leupaxin	28	43304	5.62	1	1	1	1	1	0.05	0.1
MACF1_HUMAN	Microtubule-actin cross-linking factor 1, isoforms 1/2/3/5	28	837787	5.28	31	2	26	2	26	0.08	0.01
LMCD1_HUMAN	LIM and cysteine-rich domains protein 1	28	40806	8.27	6	1	6	1	6	0.28	0.11
DNPH1_HUMAN	2'-deoxynucleoside 5'-phosphate N-hydrolase 1	28	19097	4.97	3	1	2	1	2	0.26	0.24
DJC11_HUMAN	DnaJ homolog subfamily C member 11	28	63239	8.54	7	1	4	1	4	0.13	0.07
PTCD3_HUMAN	Pentatricopeptide repeat domain-containing protein 3, mitochondrial	28	78500	6	4	1	3	1	3	0.07	0.05
ZFYV1_HUMAN	Zinc finger FYVE domain-containing protein 1	28	87120	7.29	4	1	4	1	4	0.1	0.05
COG3_HUMAN	Conserved oligomeric Golgi complex subunit 3	27	94036	5.39	2	1	2	1	2	0.07	0.05
KGUA_HUMAN	Guanylate kinase	27	21712	6.11	2	2	2	2	2	0.17	0.46
EMAL4_HUMAN	Echinoderm microtubule-associated protein-like 4	27	108848	5.96	3	1	3	1	3	0.08	0.04
CSN1_HUMAN	COP9 signalosome complex subunit 1	27	55501	6.3	5	2	3	2	3	0.12	0.16
FADD_HUMAN	FAS-associated death domain protein	27	23265	5.48	3	1	3	1	3	0.37	0.19
MA2B1_HUMAN	Lysosomal alpha-mannosidase	27	113672	6.84	9	2	7	2	7	0.14	0.08
DDX58_HUMAN	Probable ATP-dependent RNA helicase DDX58	27	106531	6.03	7	1	6	1	6	0.11	0.04
SRP14_HUMAN	Signal recognition particle 14 kDa protein	27	14561	10.05	3	1	2	1	2	0.34	0.32
VP33A_HUMAN	Vacuolar protein sorting-associated protein 33A	27	67568	6.5	4	2	3	1	3	0.08	0.06
CLN3_HUMAN	Battenin	26	47592	5.93	1	1	1	1	1	0.05	0.09
GMPR2_HUMAN	GMP reductase 2	26	37850	6.79	5	1	4	1	4	0.21	0.12
PLS3_HUMAN	Phospholipid scramblase 3	26	31628	6.22	2	1	2	1	2	0.19	0.14
PCOC1_HUMAN	Procollagen C-endopeptidase enhancer 1	26	47942	7.41	2	2	2	2	2	0.12	0.19
MUC18_HUMAN	Cell surface glycoprotein MUC18	26	71563	5.58	4	1	4	1	4	0.13	0.06
YES_HUMAN	Tyrosine-protein kinase Yes	26	60763	6.32	5	1	4	1	4	0.12	0.07
HDAC6_HUMAN	Histone deacetylase 6	26	131336	5.14	1	1	1	1	1	0.02	0.03
PMM2_HUMAN	Phosphomannomutase 2	26	28064	6.35	4	1	2	1	2	0.1	0.16
DYST_HUMAN	Dystonin	26	860127	5.14	30	2	27	2	27	0.07	0.01
QCR1_HUMAN	Cytochrome b-c1 complex subunit 1, mitochondrial	26	52612	5.94	6	1	5	1	5	0.19	0.08
DDX5_HUMAN	Probable ATP-dependent RNA helicase DDX5	26	69105	9.06	7	2	5	2	4	0.14	0.13
DAB2_HUMAN	Disabled homolog 2	26	82397	5.34	3	1	3	1	3	0.08	0.05
HSDL2_HUMAN	Hydroxysteroid dehydrogenase-like protein 2	26	45366	8.07	7	1	6	1	6	0.26	0.1
TR16L_HUMAN	Tripartite motif-containing protein 16-like protein	25	40280	5.86	1	1	1	1	1	0.07	0.11
F122B_HUMAN	Protein FAM122B	25	26911	5.98	1	1	1	1	1	0.09	0.17
FBN1_HUMAN	Fibrillin-1	25	312022	4.81	23	1	18	1	18	0.13	0.01
PEPL1_HUMAN	Probable aminopeptidase NPEPL1	25	55825	6.41	1	1	1	1	1	0.06	0.08
GOGB1_HUMAN	Golgin subfamily B member 1	25	375790	4.96	25	1	21	1	21	0.1	0.01
ABC3C_HUMAN	DNA dC->dU-editing enzyme APOBEC-3C	25	22811	7.52	2	1	2	1	2	0.16	0.2
SDF2_HUMAN	Stromal cell-derived factor 2	25	23011	6.83	3	2	2	1	2	0.26	0.2
CO8A1_HUMAN	Collagen alpha-1(VIII) chain	25	73317	9.62	4	1	3	1	3	0.11	0.06
DHRS4_HUMAN	Dehydrogenase/reductase SDR family member 4	25	29518	8.81	1	1	1	1	1	0.1	0.15
PGFRB_HUMAN	Platelet-derived growth factor receptor beta	25	123889	4.88	6	1	5	1	4	0.08	0.03
TIA1_HUMAN	Nucleolysin TIA-1 isoform p40	25	42936	7.62	1	1	1	1	1	0.05	0.1
CPIN1_HUMAN	Anamorsin	25	33561	5.44	1	1	1	1	1	0.09	0.13
ATOX1_HUMAN	Copper transport protein ATOX1	24	7397	6.71	7	1	4	1	4	0.66	0.71
MCAT_HUMAN	Mitochondrial carnitine/acylcarnitine carrier protein	24	32922	9.48	4	1	3	1	3	0.29	0.13
MPPB_HUMAN	Mitochondrial-processing peptidase subunit beta	24	54331	6.38	6	1	4	1	4	0.17	0.08
ROA3_HUMAN	Heterogeneous nuclear ribonucleoprotein A3	24	39571	9.1	7	1	6	1	5	0.24	0.11
EPG5_HUMAN	Ectopic P granules protein 5 homolog	24	292293	5.99	5	1	5	1	5	0.04	0.01
QPCTL_HUMAN	Glutaminyl-peptide cyclotransferase-like protein	24	42897	9.84	1	1	1	1	1	0.09	0.1
ADAS_HUMAN	Alkyldihydroxyacetonephosphate synthase, peroxisomal	24	72866	6.99	2	1	2	1	2	0.07	0.06
MAP1S_HUMAN	Microtubule-associated protein 1S	24	112142	6.92	3	1	2	1	2	0.03	0.04
ABCD3_HUMAN	ATP-binding cassette sub-family D member 3	24	75428	9.41	6	1	4	1	4	0.12	0.06
MPRIP_HUMAN	Myosin phosphatase Rho-interacting protein	24	116461	5.89	10	1	8	1	8	0.13	0.04
DCTD_HUMAN	Deoxycytidylate deaminase	24	20003	7.49	1	1	1	1	1	0.1	0.23
ACOT1_HUMAN	Acyl-coenzyme A thioesterase 1	23	46248	6.9	2	2	2	2	2	0.11	0.2
AIMP1_HUMAN	Aminoacyl tRNA synthase complex-interacting multifunctional protein 1	23	34331	8.61	7	1	6	1	6	0.37	0.13
BICD2_HUMAN	Protein bicaudal D homolog 2	23	93476	5.35	5	1	4	1	4	0.08	0.05
MTCH2_HUMAN	Mitochondrial carrier homolog 2	23	33309	8.25	2	1	2	1	2	0.1	0.13
MAOM_HUMAN	NAD-dependent malic enzyme, mitochondrial	23	65402	7.53	6	1	6	1	6	0.27	0.07
PSD10_HUMAN	26S proteasome non-ATPase regulatory subunit 10	23	24412	5.71	8	1	4	1	4	0.34	0.18
PCAT2_HUMAN	Lysophosphatidylcholine acyltransferase 2	23	60169	6.14	4	1	3	1	3	0.06	0.07
RU2A_HUMAN	U2 small nuclear ribonucleoprotein A'	23	28398	8.72	1	1	1	1	1	0.1	0.16
LTOR1_HUMAN	Ragulator complex protein LAMTOR1	23	17734	5.01	5	1	4	1	4	0.49	0.26
CYR61_HUMAN	Protein CYR61	23	41998	8.64	6	1	5	1	5	0.19	0.1
DHX29_HUMAN	ATP-dependent RNA helicase DHX29	22	155139	8.3	7	1	6	1	6	0.08	0.03
IDE_HUMAN	Insulin-degrading enzyme	22	117893	6.16	1	1	1	1	1	0.02	0.04
PGH1_HUMAN	Prostaglandin G/H synthase 1	22	68642	6.81	3	1	3	1	3	0.1	0.06
NNRE_HUMAN	NAD(P)H-hydrate epimerase	22	31654	7.56	1	1	1	1	1	0.13	0.14
LG3BP_HUMAN	Galectin-3-binding protein	22	65289	5.13	2	1	1	1	1	0.03	0.07
SLFN5_HUMAN	Schlafen family member 5	22	100990	8.54	3	1	3	1	3	0.09	0.04
ELMD2_HUMAN	ELMO domain-containing protein 2	22	34938	8.11	9	1	6	1	6	0.31	0.13
RAGP1_HUMAN	Ran GTPase-activating protein 1	22	63502	4.63	4	1	4	1	4	0.11	0.07
NCEH1_HUMAN	Neutral cholesterol ester hydrolase 1	22	45779	6.76	2	1	1	1	1	0.07	0.1
EDC4_HUMAN	Enhancer of mRNA-decapping protein 4	21	151567	5.55	5	1	5	1	5	0.09	0.03

[a]Protein score is calculated from the score of the peptide attributed to the protein.
[b]pI is (Predicted) isoelectric point.
[c]Number of matches is spectrum number matched to protein[#1].
[d]Number of significant matches is spectrum number that matches protein and exceeds the identification criteria.
[e]Number of sequences is number of peptides matched to protein[#2].
[f]Number of significant sequences is number of peptides exceeding the identification criteria matched to proteins.
[g]Number of unique sequences is a unique[#3] number of peptides matched to proteins.
[h]Sequence coverage is the ratio of the total number of matched peptide residues to the total length of the protein.
[i]Exponentially Modified Protein Abundance Index (http://www.matrixscience.com/help/quant_empai_help.html).

Table S2. Cont 12.

UniProt/SWISS-PROT ID	Description	Protein score[a]	Protein mass (kDa)	pI[b]	Num. of matches[c]	Num. of significant matches[d]	Num. of sequences[e]	Num. of significant sequences[f]	Num. of unique sequences[g]	Sequence coverage[h]	emPAI[i]
PPR18_HUMAN	Phostensin	21	67902	5.38	7	1	4	1	4	0.11	0.06
MANF_HUMAN	Mesencephalic astrocyte-derived neurotrophic factor	21	20687	8.93	5	1	2	1	2	0.16	0.22
SGCB_HUMAN	Beta-sarcoglycan	21	34755	8.86	2	1	2	1	2	0.11	0.13
YIPF3_HUMAN	Protein YIPF3	20	38223	5.47	1	1	1	1	1	0.08	0.11
UBP2L_HUMAN	Ubiquitin-associated protein 2-like	20	114465	6.61	9	1	5	1	5	0.1	0.04
TM181_HUMAN	Transmembrane protein 181	19	69280	9.1	2	1	2	1	2	0.08	0.06
PAWR_HUMAN	PRKC apoptosis WT1 regulator protein	19	36545	5.35	1	1	1	1	1	0.05	0.12
MCU_HUMAN	Calcium uniporter protein, mitochondrial	19	39842	8.83	4	1	3	1	3	0.16	0.11
S2546_HUMAN	Solute carrier family 25 member 46	19	46145	6.97	3	1	1	1	1	0.06	0.09
HIG1A_HUMAN	HIG1 domain family member 1A, mitochondrial	19	10136	9.79	1	1	1	1	1	0.24	0.49
CBPQ_HUMAN	Carboxypeptidase Q	19	51855	5.79	2	1	1	1	1	0.07	0.08
FND3B_HUMAN	Fibronectin type III domain-containing protein 3B	18	132803	5.64	7	1	6	1	6	0.1	0.03
VTA1_HUMAN	Vacuolar protein sorting-associated protein VTA1 homolog	18	33858	5.87	5	1	3	1	3	0.16	0.13
MARE3_HUMAN	Microtubule-associated protein RP/EB family member 3	17	31962	5.33	2	1	2	1	2	0.11	0.14
DHX36_HUMAN	ATP-dependent RNA helicase DHX36	15	114688	7.58	3	1	3	1	3	0.05	0.04

[a]Protein score is calculated from the score of the peptide attributed to the protein.
[b]pI is (Predicted) isoelectric point.
[c]Number of matches is spectrum number matched to protein[#1].
[d]Number of significant matches is spectrum number that matches protein and exceeds the identification criteria.
[e]Number of sequences is number of peptides matched to protein[#2].
[f]Number of significant sequences is number of peptides exceeding the identification criteria matched to proteins.
[g]Number of unique sequences is a unique[#3] number of peptides matched to proteins.
[h]Sequence coverage is the ratio of the total number of matched peptide residues to the total length of the protein.
[i]Exponentially Modified Protein Abundance Index (http://www.matrixscience.com/help/quant_empai_help.html).

Table S3. Identification of endogenous proteins (Biological Precess: growth) in hADSC; group CDM & DMEM.

Table S3. Identification of endogenous proteins (Biological Precess: growth) in hADSC; group CDM & DMEM.

UniProt/SWISS-Alternate ID	Biological Process growth	emPAI[a] CDM	emPAI[a] DMEM
VIM	skeletal muscle tissue regeneration	588.33	1108.52
TUBB6	seed trichome elongation	488.41	682.32
IQGAP1	neuron projection extension	101.79	191.57
VCL	axon extension	47.63	161.43
ANXA1	myoblast migration involved in skeletal muscle regeneration	51.36	88.25
MAP1B	axon extension	20.55	139.91
ATP5A1	growth	44.83	92.56
MYL9	cell elongation involved in imaginal disc-derived wing morphogenesis	40.16	92.56
PSAP	prostate gland growth	38.29	55.96
CLIC4	multicellular organism growth	19.61	71.03
GNAI2	multicellular organism growth	26.15	55.96
GNAS	developmental growth, multicellular organism growth	1.87	17.22
ITGB1	axon extension	24.28	55.96
MAPK1	endochondral bone growth	22.41	34.44
RAB10	pollen tube growth	15.88	64.57
ATP6V1A	imaginal disc growth	10.27	66.73
TNC	bud outgrowth involved in lung branching	5.60	75.34
ITGAV	cell growth	3.74	43.05
EIF4H	developmental growth	14.94	21.52
MTPN	cell growth, skeletal muscle tissue regeneration	18.68	10.76
POSTN	bone regeneration, neuron projection extension	14.01	23.68
SLC3A2	cell growth	1.87	43.05
PAK2	budding cell apical bud growth	12.14	10.76
SEC61A1	cell growth	14.94	10.76
CDC42	multicellular organism growth	10.27	17.22
CPNE1	neuron projection extension	12.14	10.76
GPX1	skeletal muscle tissue regeneration	3.74	12.91
PTK7	axis elongation	0.93	21.52
TRIM28	convergent extension involved in axis elongation	6.54	10.76
TMED2	multicellular organism growth	4.67	12.91
PRKAR1A	cardiac muscle cell proliferation	5.60	10.76

[a]Exponentially Modified Protein Abundance Index (http://www.matrixscience.com/help/quant_empai_help.html).

Table S4. Identification of endogenous proteins (Molecular Function: antioxidant activity) in hADSC; group CDM & DMEM.

Table S4. Identification of endogenous proteins (Molecular Function: antioxidant activity) in hADSC; group CDM & DMEM.

UniProt/SWISS-Alternate ID	Molecular Function antioxidant activity	emPAI[a]	
		CDM	DMEM
ALB	antioxidant activity	698.52	47.35
GSTP1	glutathione peroxidase activity	58.83	124.84
PRDX6	antioxidant activity, glutathione peroxidase activity, peroxidase activity, peroxiredoxin activity	48.56	64.57
PARK7	peroxiredoxin activity	30.82	51.66
PRDX1	peroxidase activity, peroxiredoxin activity, thioredoxin peroxidase activity	38.29	60.27
GSTO1	glutathione dehydrogenase (ascorbate) activity	26.15	30.13
PRDX4	antioxidant activity, peroxidase activity, peroxiredoxin activity, thioredoxin peroxidase activity	20.55	60.27
PRDX5	peroxidase activity, peroxynitrite reductase activity, thioredoxin peroxidase activity	14.01	49.51
TXNRD1	thioredoxin-disulfide reductase activity	2.80	66.73
PRDX2	antioxidant activity, peroxidase activity, peroxiredoxin activity, thioredoxin peroxidase activity	11.21	19.37
TXNDC17	peroxidase activity	5.60	21.52
GSTK1	glutathione peroxidase activity	6.54	15.07
PRDX3	peroxidase activity, peroxiredoxin activity, thioredoxin peroxidase activity	7.47	12.91
MGST1	glutathione peroxidase activity	8.40	10.76
GPX1	glutathione peroxidase activity	3.74	12.91
SOD1	superoxide dismutase activity	4.67	15.07

[a]Exponentially Modified Protein Abundance Index (http://www.matrixscience.com/help/quant_empai_help.html).

Table S5. Identification of endogenous proteins (Biological Process: immune system process) in hADSC; group CDM & DMEM.

Table S5. Identification of endogenous proteins (Biological Process: immune system process) in hADSC; group CDM & DMEM.

UniProt/SWISS-Alternate ID	Biological Process immune system process	emPAI[a] CDM	emPAI[a] DMEM
TUBB6	natural killer cell mediated cytotoxicity	488.41	682.32
TUBB	natural killer cell mediated cytotoxicity	304.44	396.06
TUBB4B	natural killer cell mediated cytotoxicity	286.69	380.98
MYH9	establishment of T cell polarity, leukocyte migration, monocyte differentiation, platelet formation	300.70	901.88
HSP90AB1	Fc-gamma receptor signaling pathway involved in phagocytosis, toll-like receptor signaling pathway	439.85	454.16
HSP90B1	toll-like receptor signaling pathway	87.78	180.81
HSP90AA1	Fc-gamma receptor signaling pathway involved in phagocytosis	229.73	165.74
ACTN4	platelet formation	135.41	514.44
ACTN1	platelet formation	104.59	370.22
CLTC	antigen processing and presentation of exogenous peptide antigen via MHC class II	163.42	243.22
EEF2	hematopoietic progenitor cell differentiation	162.49	189.42
ANXA2	osteoclast development	116.73	271.20
LGALS1	T cell costimulation, plasma cell differentiation	118.60	180.81
FN1	leukocyte migration	97.12	251.84
DYNC1H1	antigen processing and presentation of exogenous peptide antigen via MHC class II	103.66	213.10
COL1A1	leukocyte migration	76.58	226.00
CALR	antigen processing and presentation of peptide antigen via MHC class I, peptide antigen assembly with MHC class I protein complex	115.80	120.54
PDIA3	antigen processing and presentation of peptide antigen via MHC class I	84.98	142.06
THBS1	immune response	82.18	148.52
MSN	leukocyte migration	56.03	139.91
HSPD1	B cell cytokine production, B cell proliferation, MyD88-dependent toll-like receptor signaling pathway, T cell activation, isotype switching to IgG isotypes	79.38	81.79
ARF4	antigen processing and presentation of exogenous peptide antigen via MHC class II	79.38	60.27
ARF1	antigen processing and presentation of exogenous peptide antigen via MHC class II	46.69	23.68
CTSD	antigen processing and presentation of exogenous peptide antigen via MHC class II	40.16	103.32
ANXA1	adaptive immune response, alpha-beta T cell differentiation, granulocyte chemotaxis, innate immune response, monocyte chemotaxis, neutrophil clearance, neutrophil homeostasis	51.36	88.25
COL1A2	leukocyte migration	30.82	118.39
STAT1	defense response to virus, interferon-gamma-mediated signaling pathway, type I interferon signaling pathway	56.97	64.57
EPRS	cellular response to interferon-gamma	31.75	73.18
CTSB	toll-like receptor signaling pathway	42.02	40.90
ACTR3	Fc-gamma receptor signaling pathway involved in phagocytosis	31.75	58.12
PRKDC	B cell lineage commitment, T cell differentiation in thymus, T cell lineage commitment, T cell receptor V(D)J recombination, V(D)J recombination, immunoglobulin V(D)J recombination, immunoglobulin production, pro-B cell differentiation, spleen development, thymus development	51.36	25.83
HSPA9	erythrocyte differentiation	28.95	68.88
CANX	antigen processing and presentation of exogenous peptide antigen via MHC class II, antigen processing and presentation of peptide antigen via MHC class I	30.82	53.81
GPI	erythrocyte homeostasis, humoral immune response	41.09	40.90
XRCC5	hematopoietic stem cell differentiation	34.55	43.05
PRDX1	erythrocyte homeostasis, natural killer cell mediated cytotoxicity	38.29	60.27
G6PD	erythrocyte maturation	24.28	60.27
PSMD2	T cell receptor signaling pathway, antigen processing and presentation of exogenous peptide antigen via MHC class I, TAP-dependent, stimulatory C-type lectin receptor signaling pathway	26.15	58.12
PSMD1	T cell receptor signaling pathway, antigen processing and presentation of exogenous peptide antigen via MHC class I, TAP-dependent, stimulatory C-type lectin receptor signaling pathway	24.28	60.27
ITGB1	B cell differentiation, leukocyte tethering or rolling	24.28	55.96
MAPK1	B cell receptor signaling pathway, Fc-gamma receptor signaling pathway involved in phagocytosis, T cell receptor signaling pathway, embryonic hemopoiesis, innate immune response, macrophage chemotaxis, microglia differentiation, monocyte chemotaxis, osteoclast development, thymus	22.41	34.44
MAPK3	Fc-gamma receptor signaling pathway involved in phagocytosis, thymus development	5.60	10.76
RAB10	antigen processing and presentation	15.88	64.57
RAB35	antigen processing and presentation	5.60	12.91
ACTR2	Fc-gamma receptor signaling pathway involved in phagocytosis	29.88	40.90
ARPC2	Fc-gamma receptor signaling pathway involved in phagocytosis	25.21	45.20
MIF	innate immune response, leukocyte migration	31.75	30.13
AP2A1	antigen processing and presentation of exogenous peptide antigen via MHC class II	11.21	66.73
AP2A2	antigen processing and presentation of exogenous peptide antigen via MHC class II	4.67	27.98
SEC31A	antigen processing and presentation of exogenous peptide antigen via MHC class II, antigen processing and presentation of peptide antigen via MHC class I	16.81	49.51
AP2B1	antigen processing and presentation of exogenous peptide antigen via MHC class II	20.55	62.42
DDOST	T cell activation	21.48	36.59
RPS7	hemopoiesis	26.15	27.98
DYNC1I2	antigen processing and presentation of exogenous peptide antigen via MHC class II	26.15	23.68
IPO7	innate immune response	18.68	36.59
CTSK	toll-like receptor signaling pathway	28.95	12.91
GLG1	leukocyte migration	14.01	36.59
MYO1C	Fc-gamma receptor signaling pathway involved in phagocytosis	17.74	40.90
ACTR1A	antigen processing and presentation of exogenous peptide antigen via MHC class II	15.88	34.44
PSMA4	T cell receptor signaling pathway, antigen processing and presentation of exogenous peptide antigen via MHC class I, TAP-dependent, stimulatory C-type lectin receptor signaling pathway	15.88	38.74
ARPC1B	Fc-gamma receptor signaling pathway involved in phagocytosis	15.88	38.74
C1QBP	adaptive immune response, complement activation, classical pathway, innate immune response	18.68	25.83
RAB5C	antigen processing and presentation	9.34	43.05
PGM3	hemopoiesis	9.34	36.59
DDX3X	innate immune response	18.68	23.68
PSME2	T cell receptor signaling pathway, antigen processing and presentation of exogenous peptide antigen via MHC class I, TAP-dependent, stimulatory C-type lectin receptor signaling pathway	20.55	12.91
PCBP2	defense response to virus, innate immune response	25.21	34.44
PSMC4	T cell receptor signaling pathway, antigen processing and presentation of exogenous peptide antigen via MHC class I, TAP-dependent, stimulatory C-type lectin receptor signaling pathway	13.07	25.83
ITGAV	antigen processing and presentation of exogenous peptide antigen via MHC class I, TAP-dependent, leukocyte migration	3.74	43.05
AP1B1	antigen processing and presentation of exogenous peptide antigen via MHC class II	18.68	23.68
PSMA7	T cell receptor signaling pathway, antigen processing and presentation of exogenous peptide antigen via MHC class I, TAP-dependent, stimulatory C-type lectin receptor signaling pathway	14.94	19.37
PSMD3	T cell receptor signaling pathway, antigen processing and presentation of exogenous peptide antigen via MHC class I, TAP-dependent, stimulatory C-type lectin receptor signaling pathway	8.40	36.59
CAPZA1	innate immune response	15.88	23.68
PSMA6	T cell receptor signaling pathway, antigen processing and presentation of exogenous peptide antigen via MHC class I, TAP-dependent, stimulatory C-type lectin receptor signaling pathway	11.21	23.68
RAB7A	antigen processing and presentation of exogenous peptide antigen via MHC class II	7.47	32.29
SLC3A2	leukocyte migration	1.87	43.05
PRKACA	stimulatory C-type lectin receptor signaling pathway	8.40	19.37
PRKACB	stimulatory C-type lectin receptor signaling pathway	8.40	15.07
PSMB2	T cell receptor signaling pathway, antigen processing and presentation of exogenous peptide antigen via MHC class I, TAP-dependent, stimulatory C-type lectin receptor signaling pathway	13.07	15.07
SEC13	antigen processing and presentation of exogenous peptide antigen via MHC class II, antigen processing and presentation of peptide antigen via MHC class I	6.54	27.98
UBE2V1	T cell receptor signaling pathway, nucleotide-binding oligomerization domain containing signaling pathway, stimulatory C-type lectin receptor signaling pathway	9.34	23.68
FKBP1A	T cell activation	10.27	21.52
ARPC4	Fc-gamma receptor signaling pathway involved in phagocytosis	10.27	17.22
DCTN1	antigen processing and presentation of exogenous peptide antigen via MHC class II	9.34	23.68
SHMT2	response to type I interferon	10.27	21.52
PAK2	T cell costimulation, T cell receptor signaling pathway, stimulatory C-type lectin receptor signaling pathway	12.14	10.76
PSMC3	T cell receptor signaling pathway, antigen processing and presentation of exogenous peptide antigen via MHC class I, TAP-dependent, stimulatory C-type lectin receptor signaling pathway	3.74	36.59
SEC24D	antigen processing and presentation of exogenous peptide antigen via MHC class II, antigen processing and presentation of peptide antigen via MHC class I	6.54	23.68
PSMA5	T cell receptor signaling pathway, antigen processing and presentation of exogenous peptide antigen via MHC class I, TAP-dependent, stimulatory C-type lectin receptor signaling pathway	0.93	38.74
CD109	osteoclast fusion	13.07	12.91
PSMD14	T cell receptor signaling pathway, antigen processing and presentation of exogenous peptide antigen via MHC class I, TAP-dependent, stimulatory C-type lectin receptor signaling pathway	12.14	10.76
SEC61A1	response to interferon-gamma	14.94	10.76
AHCY	chronic inflammatory response to antigenic stimulus	12.14	19.37
PSMB5	T cell receptor signaling pathway, antigen processing and presentation of exogenous peptide antigen via MHC class I, TAP-dependent, stimulatory C-type lectin receptor signaling pathway	6.54	27.98
PICALM	hemopoiesis	5.60	23.68
AP2M1	antigen processing and presentation of exogenous peptide antigen via MHC class II	8.40	12.91
ARPC3	Fc-gamma receptor signaling pathway involved in phagocytosis	8.40	19.37
PPP1R14B	innate immune response	15.88	2.15
PSMA2	T cell receptor signaling pathway, antigen processing and presentation of exogenous peptide antigen via MHC class I, TAP-dependent, stimulatory C-type lectin receptor signaling pathway	14.94	8.61
PSMB4	T cell receptor signaling pathway, antigen processing and presentation of exogenous peptide antigen via MHC class I, TAP-dependent, stimulatory C-type lectin receptor signaling pathway	7.47	17.22

[a]Exponentially Modified Protein Abundance Index (http://www.matrixscience.com/help/quant_empai_help.html).

Table S5. Cont 1.

UniProt/SWISS-Alternate ID	Biological Process immune system process	emPAI[a] CDM	emPAI[a] DMEM
PSMD13	T cell receptor signaling pathway, antigen processing and presentation of exogenous peptide antigen via MHC class I, TAP-dependent, stimulatory C-type lectin receptor signaling pathway	13.07	12.91
PSMC2	T cell receptor signaling pathway, antigen processing and presentation of exogenous peptide antigen via MHC class I, TAP-dependent, stimulatory C-type lectin receptor signaling pathway	7.47	23.68
CDC42	Fc-gamma receptor signaling pathway involved in phagocytosis, T cell costimulation, dendritic cell migration, macrophage differentiation	10.27	17.22
DPP4	T cell activation, T cell costimulation	0.93	36.59
CAPZA2	innate immune response	7.47	30.13
PAFAH1B1	osteoclast development	9.34	12.91
PNP	immune response	4.67	27.98
PSMC5	T cell receptor signaling pathway, antigen processing and presentation of exogenous peptide antigen via MHC class I, TAP-dependent, stimulatory C-type lectin receptor signaling pathway	7.47	17.22
PSMB3	T cell receptor signaling pathway, antigen processing and presentation of exogenous peptide antigen via MHC class I, TAP-dependent, stimulatory C-type lectin receptor signaling pathway	8.40	17.22
RPS17	erythrocyte homeostasis	8.40	19.37
UBE2N	T cell receptor signaling pathway, nucleotide-binding oligomerization domain containing signaling pathway, stimulatory C-type lectin receptor signaling pathway	9.34	15.07
SYNCRIP	cellular response to interferon-gamma	3.74	17.22
IMPDH2	lymphocyte proliferation	7.47	19.37
GLO1	osteoclast differentiation	9.34	8.61
PSMB6	T cell receptor signaling pathway, antigen processing and presentation of exogenous peptide antigen via MHC class I, TAP-dependent, stimulatory C-type lectin receptor signaling pathway	8.40	15.07
PSMD7	T cell receptor signaling pathway, antigen processing and presentation of exogenous peptide antigen via MHC class I, TAP-dependent, stimulatory C-type lectin receptor signaling pathway	4.67	19.37
PPP3CA	T cell activation	1.87	17.22
PSMB1	T cell receptor signaling pathway, antigen processing and presentation of exogenous peptide antigen via MHC class I, TAP-dependent, stimulatory C-type lectin receptor signaling pathway	6.54	17.22
PSMB7	T cell receptor signaling pathway, antigen processing and presentation of exogenous peptide antigen via MHC class I, TAP-dependent, stimulatory C-type lectin receptor signaling pathway	9.34	12.91
PSMC6	T cell receptor signaling pathway, antigen processing and presentation of exogenous peptide antigen via MHC class I, TAP-dependent, stimulatory C-type lectin receptor signaling pathway	7.47	10.76
ITGB5	antigen processing and presentation of exogenous peptide antigen via MHC class I, TAP-dependent	3.74	23.68
OTUB1	adaptive immune response	6.54	12.91
RPL22	alpha-beta T cell differentiation	8.40	12.91
STAT2	defense response to virus, type I interferon signaling pathway	7.47	6.46
ENPP1	immune response	6.54	12.91
PRDX3	myeloid cell differentiation	7.47	12.91
PSMD4	T cell receptor signaling pathway, antigen processing and presentation of exogenous peptide antigen via MHC class I, TAP-dependent, stimulatory C-type lectin receptor signaling pathway	4.67	19.37
SKP1	T cell receptor signaling pathway, stimulatory C-type lectin receptor signaling pathway	3.74	21.52
AP3B1	antigen processing and presentation, antigen processing and presentation, exogenous lipid antigen via MHC class Ib	4.67	12.91
CTPS1	B cell proliferation, T cell proliferation	7.47	12.91
RAC1	Fc-gamma receptor signaling pathway involved in phagocytosis, T cell costimulation, mast cell chemotaxis	7.47	15.07
CTNNB1	T cell differentiation, T cell differentiation in thymus, osteoclast differentiation, thymus development	0.93	21.52
PSMD11	T cell receptor signaling pathway, antigen processing and presentation of exogenous peptide antigen via MHC class I, TAP-dependent, stimulatory C-type lectin receptor signaling pathway	3.74	19.37
PSMD12	T cell receptor signaling pathway, antigen processing and presentation of exogenous peptide antigen via MHC class I, TAP-dependent, stimulatory C-type lectin receptor signaling pathway	3.74	12.91
TRIM28	innate immune response	6.54	10.76
DYNLL1	antigen processing and presentation of exogenous peptide antigen via MHC class II	5.60	12.91
TMOD3	erythrocyte development	7.47	8.61
STOML2	CD4-positive, alpha-beta T cell activation, T cell receptor signaling pathway	3.74	12.91
PSMD6	T cell receptor signaling pathway, antigen processing and presentation of exogenous peptide antigen via MHC class I, TAP-dependent, stimulatory C-type lectin receptor signaling pathway	7.47	6.46
DCTN2	antigen processing and presentation of exogenous peptide antigen via MHC class II	1.87	17.22
PSME1	T cell receptor signaling pathway, antigen processing and presentation of exogenous antigen, antigen processing and presentation of exogenous peptide antigen via MHC class I, TAP-dependent, stimulatory C-type lectin receptor signaling pathway	2.80	15.07
TROVE2	immune system development	6.54	8.61
SOD1	myeloid cell homeostasis, thymus development	4.67	15.07
LGALS3	eosinophil chemotaxis, innate immune response, macrophage chemotaxis, monocyte chemotaxis, mononuclear cell migration, neutrophil chemotaxis	2.80	15.07
UBE2D2	T cell receptor signaling pathway, TRIF-dependent toll-like receptor signaling pathway, stimulatory C-type lectin receptor signaling pathway	8.40	4.31
ARPC1A	Fc-gamma receptor signaling pathway involved in phagocytosis	3.74	17.22

[a]Exponentially Modified Protein Abundance Index (http://www.matrixscience.com/help/quant_empai_help.html).

Table S6. Identification of endogenous proteins (Biological Process: biological regulation) in hADSC; group CDM & DMEM.

Table S6. Identification of endogenous proteins (Biological Process: biological regulation) in hADSC; group CDM & DMEM.

UniProt/SWISS-Alternate ID	Biological Process<br>biological regulation	emPAI[a] CDM	emPAI[a] DMEM
ACTA2	positive regulation of gene expression, regulation of blood pressure, vascular smooth muscle contraction	285.76	439.10
POTEE	retina homeostasis	246.54	208.78
VIM	SMAD protein signal transduction, negative regulation of neuron projection development, positive regulation of gene expression, positive regulation of glial cell proliferation, regulation of Schwann cell migration, regulation of axonogenesis	588.33	1108.52
MYH9	integrin-mediated signaling pathway, negative regulation of actin filament polymerization, negative regulation of actin filament severing, platelet aggregation, positive regulation of protein processing in phagocytic vesicle, regulation of cell shape, regul	300.70	901.88
MYH10	regulation of cell shape	18.68	124.84
FLNA	adenylate cyclase-inhibiting dopamine receptor signaling pathway, cytoplasmic sequestering of protein, negative regulation of apoptotic process, negative regulation of protein catabolic process, negative regulation of sequence-specific DNA binding transcri	223.19	884.66
HSP90AB1	ATF6-mediated unfolded protein response, ERBB2 signaling pathway, Fc-gamma receptor signaling pathway involved in phagocytosis, negative regulation of complement-dependent cytotoxicity, negative regulation of neuron apoptotic process, negative regulation o	439.85	454.16
HSP90B1	ATF6-mediated unfolded protein response, negative regulation of apoptotic process, regulation of phosphoprotein phosphatase activity, sequestering of calcium ion, toll-like receptor signaling pathway	87.78	180.81
HSP90AA1	ERBB2 signaling pathway, Fc-gamma receptor signaling pathway involved in phagocytosis, positive regulation of cardiac muscle contraction, positive regulation of cell size, positive regulation of lamellipodium assembly, positive regulation of nitric oxide bi	229.73	165.74
ALB	hemolysis by symbiont of host erythrocytes, maintenance of mitochondrion location, negative regulation of apoptotic process, negative regulation of programmed cell death, retina homeostasis	698.52	47.35
EEF1A1	calcium-mediated signaling, regulation of chaperone-mediated autophagy, regulation of transcription, DNA-templated	382.88	376.68
HSPA8	ATF6-mediated unfolded protein response, ER overload response, IRE1-mediated unfolded protein response, PERK-mediated unfolded protein response, RNA interference, activation of signaling protein activity involved in unfolded protein response, endoplasmic r	303.50	559.64
HSPA5	maintenance of protein localization in endoplasmic reticulum, positive regulation of cell migration	132.61	247.54
HSPA1A	negative regulation of cell death, negative regulation of endoplasmic reticulum stress-induced intrinsic apoptotic signaling pathway, negative regulation of extrinsic apoptotic signaling pathway in absence of ligand, negative regulation of inclusion body a	15.88	51.66
HSPA2	negative regulation of inclusion body assembly, positive regulation of calcium-transporting ATPase activity, positive regulation of cyclin-dependent protein serine/threonine kinase activity involved in G2/M transition of mitotic cell cycle, positive regulation of protein phosphorylation	19.61	36.59
TUBA1B	establishment or maintenance of cell polarity regulating cell shape	330.58	292.74
YWHAQ	negative regulation of ion transmembrane transport, negative regulation of transcription, DNA-templated, positive regulation of protein insertion into mitochondrial membrane involved in apoptotic signaling pathway, small GTPase mediated signal transduction	29.88	60.27
YWHAG	negative regulation of protein kinase activity, negative regulation of protein serine/threonine kinase activity, positive regulation of protein insertion into mitochondrial membrane involved in apoptotic signaling pathway, regulation of neuron differentiation, regulation of signal transduction, regulation of synaptic plastici	30.82	47.35
YWHAE	hippo signaling, intracellular signal transduction, membrane repolarization during cardiac muscle cell action potential, negative regulation of cysteine-type endopeptidase activity involved in apoptotic process, negative regulation of peptidyl-serine dephos	42.96	51.66
YWHAH	glucocorticoid receptor signaling pathway, membrane depolarization during action potential, negative regulation of dendrite morphogenesis, positive regulation of protein insertion into mitochondrial membrane involved in apoptotic signaling pathway, positive	10.27	30.13
ENO1	negative regulation of cell growth, negative regulation of transcription, DNA-templated	254.94	273.36
ACTN4	MAPK cascade, actin filament capping, cardiac conduction, cytoskeletal anchoring at plasma membrane, negative regulation of cellular component movement, negative regulation of heart rate, negative regulation of substrate adhesion-dependent cell spreading, peroxisome proliferator acti	135.41	514.44
ACTN1	negative regulation of cellular component movement, platelet aggregation, regulation of apoptotic process, regulation of nucleic acid-templated transcription	104.59	370.22
TLN1	IRE1-mediated unfolded protein response, cytoskeletal anchoring at plasma membrane, platelet aggregation	131.67	374.52
TLN2	cytoskeletal anchoring at plasma membrane	7.47	23.68
CLTC	Wnt signaling pathway, planar cell polarity pathway, negative regulation of hyaluronan biosynthetic process, negative regulation of protein localization to plasma membrane, positive regulation of glucose import, regulation of mitotic spindle organization, si	163.42	243.22
CLTCL1	positive regulation of glucose import, signal transduction	20.55	30.13
P4HB	cell redox homeostasis, positive regulation of viral entry into host cell, regulation of oxidative stress-induced intrinsic apoptotic signaling pathway	136.34	236.78
LDHA	positive regulation of apoptotic process	107.39	458.48
EEF2	positive regulation of cytoplasmic translation, positive regulation of translation	162.49	189.42
ALDOA	mitotic G2 DNA damage checkpoint, muscle cell cellular homeostasis, regulation of cell shape	110.19	279.82
ANXA2	body fluid secretion, fibrinolysis, negative regulation of catalytic activity, negative regulation of low-density lipoprotein particle receptor catabolic process, negative regulation of receptor binding, negative regulation of receptor internalization, posi	116.73	271.20
PLEC	negative regulation of cell migration, negative regulation of epithelial cell proliferation, negative regulation of keratinocyte migration, negative regulation of keratinocyte proliferation, regulation of epithelium regeneration	82.18	284.12
LGALS1	T cell costimulation, cellular response to glucose stimulus, negative regulation of cell-substrate adhesion, negative regulation of neuron projection development, positive regulation of I-kappaB kinase/NF-kappaB signaling, positive regulation of dendritic cell differentiation, positive regulation of erythrocyte aggregatic	118.60	180.81
FN1	cellular response to glucose stimulus, negative regulation of apoptotic process, negative regulation of transforming growth factor-beta secretion, positive regulation of axon extension, positive regulation of cell migration, positive regulation of cell proliferation, positive regulation of chemotaxis, positive regulation of f	97.12	251.84
COL6A3	negative regulation of endopeptidase activity, negative regulation of peptidase activity	126.07	180.81
COL1A1	blood coagulation, negative regulation of cell-substrate adhesion, platelet activation, positive regulation of canonical Wnt signaling pathway, positive regulation of cell migration, positive regulation of epithelial to mesenchymal transition, positive regulation of transcription, DNA-templated, regulation of immune respor	76.58	226.00
SERPINH1	negative regulation of endopeptidase activity	127.94	159.28
AHNAK	regulation of RNA splicing, regulation of voltage-gated calcium channel activity	42.02	331.48
IQGAP1	Arp2/3 complex-mediated actin nucleation, ERK1 and ERK2 cascade, Ras protein signal transduction, activation of MAPK activity, epidermal growth factor receptor signaling pathway, fibroblast growth factor receptor signaling pathway, negative regulation of G	101.79	191.57
FASN	positive regulation of cellular metabolic process	130.74	96.86
PFN1	Wnt signaling pathway, planar cell polarity pathway, negative regulation of actin filament polymerization, negative regulation of stress fiber assembly, positive regulation of ATPase activity, positive regulation of DNA metabolic process, positive regulatio	103.66	157.13
CALR	ATF6-mediated unfolded protein response, cell cycle arrest, cellular calcium ion homeostasis, glucocorticoid receptor signaling pathway, negative regulation of intracellular steroid hormone receptor signaling pathway, negative regulation of neuron differen	115.80	120.54
MARCKS	regulation of insulin secretion	88.72	178.65
ATP5B	negative regulation of cell adhesion involved in substrate-bound cell migration, regulation of intracellular pH	83.11	101.17
PDIA3	cell redox homeostasis, positive regulation of extrinsic apoptotic signaling pathway, protein retention in ER lumen, signal transduction	84.98	142.06
THBS1	activation of MAPK activity, cell cycle arrest, negative regulation of angiogenesis, negative regulation of antigen processing and presentation of peptide or polysaccharide antigen via MHC class II, negative regulation of blood vessel endothelial cell migration, negative regulation of cGMP-mediated signaling, negative	82.18	148.52
CAP1	activation of adenylate cyclase activity, regulation of adenylate cyclase activity, signal transduction	98.99	120.54
VCP	activation of cysteine-type endopeptidase activity involved in apoptotic process, endoplasmic reticulum unfolded protein response, positive regulation of ATP biosynthetic process, positive regulation of Lys63-specific deubiquitinase activity, positive regulation of mitochondrial membrane potential, positive regulatio	85.92	133.45
CFL1	Rho protein signal transduction, negative regulation of apoptotic process, positive regulation by host of viral process, regulation of dendritic spine morphogenesis	128.87	96.86
GSN	actin nucleation, barbed-end actin filament capping, negative regulation of viral entry into host cell, positive regulation of actin nucleation, positive regulation of cysteine-type endopeptidase activity involved in apoptotic signaling pathway, positive r	89.65	99.01
ANXA5	blood coagulation, hemostasis, negative regulation of apoptotic process, negative regulation of catalytic activity, negative regulation of coagulation, negative regulation of sequestering of calcium ion, positive regulation of defense response to virus by	70.97	159.28
MSN	actin filament capping, barbed-end actin filament capping, cytoskeletal anchoring at plasma membrane, negative regulation of ERK1 and ERK2 cascade, negative regulation of GTPase activity, negative regulation of T cell receptor signaling pathway, negative r	56.03	139.91
EZR	cytoskeletal anchoring at plasma membrane, negative regulation of ERK1 and ERK2 cascade, negative regulation of T cell receptor signaling pathway, negative regulation of interleukin-2 secretion, negative regulation of p38MAPK cascade, negative regulation o	14.94	32.29
RDX	actin filament capping, barbed-end actin filament capping, negative regulation of GTPase activity, negative regulation of adherens junction organization, negative regulation of cell size, negative regulation of homotypic cell-cell adhesion, positive regulati	12.14	34.44
VCL	negative regulation of cell migration, platelet aggregation	47.63	161.43
HSPD1	MyD88-dependent toll-like receptor signaling pathway, activation of cysteine-type endopeptidase activity involved in apoptotic process, negative regulation of apoptotic process, negative regulation of apoptotic process in bone marrow, negative regulation o	79.38	81.79
TAGLN2	negative regulation of transcription from RNA polymerase II promoter	69.11	99.01
ACLY	positive regulation of cellular metabolic process	70.97	105.47
S100A6	positive regulation of fibroblast proliferation, signal transduction	101.79	12.91
GSTP1	negative regulation of ERK1 and ERK2 cascade, negative regulation of I-kappaB kinase/NF-kappaB signaling, negative regulation of JUN kinase activity, negative regulation of MAPK cascade, negative regulation of acute inflammatory response, negative regulation of apoptotic process, negative regulation of extrins	58.83	124.84
EIF4A1	negative regulation of RNA-directed 5'-3' RNA polymerase activity, negative regulation of excitatory postsynaptic potential, negative regulation of selenocysteine incorporation, negative regulation of selenocysteine insertion sequence binding, negative regulation of translation, nuclear-transcribed mRNA catabolic p	70.97	86.10
EIF4A2	negative regulation of RNA-directed 5'-3' RNA polymerase activity, nuclear-transcribed mRNA poly(A) tail shortening, regulation of translational initiation	29.88	38.74
TKT	regulation of growth	70.04	83.95
ARF4	activation of phospholipase D activity, cellular copper ion homeostasis, epidermal growth factor receptor signaling pathway, long term synaptic depression, negative regulation of apoptotic process, positive regulation of ER to Golgi vesicle-mediated transp	79.38	60.27
ARF1	cellular copper ion homeostasis, long term synaptic depression, positive regulation of ER to Golgi vesicle-mediated transport, positive regulation of calcium ion-dependent exocytosis, positive regulation of catalytic activity, positive regulation of dendri	46.69	23.68
ARF3	small GTPase mediated signal transduction	40.16	17.22
ARF5	small GTPase mediated signal transduction	15.88	15.07
CAPN2	regulation of cytoskeleton organization	75.64	86.10
LRP1	negative regulation of Wnt signaling pathway, negative regulation of neuron apoptotic process, negative regulation of neuron projection development, negative regulation of platelet-derived growth factor receptor-beta signaling pathway, negative regulation	46.69	124.84
S100A11	negative regulation of DNA replication, negative regulation of cell proliferation, regulation of cell proliferation, signal transduction	79.38	55.96
PGAM1	positive regulation of flagellated sperm motility, regulation of glycolytic process, regulation of pentose-phosphate shunt	60.70	73.18
PGAM4	positive regulation of flagellated sperm motility, regulation of pentose-phosphate shunt	32.69	15.07
WDR1	positive regulation of actin filament depolymerization, regulation of oligodendrocyte differentiation, regulation of ventricular cardiac muscle cell membrane repolarization	45.76	118.39
KPNB1	Ran protein signal transduction, modulation by virus of host process	59.77	79.64
ANXA1	G-protein coupled receptor signaling pathway, coupled to cyclic nucleotide second messenger, cell surface receptor signaling pathway, insulin secretion, negative regulation of T-helper 2 cell differentiation, negative regulation of apoptotic process, negative regulation of catalytic activity, negative regulation of exocy	51.36	88.25
EIF5A	polyamine homeostasis, positive regulation of cell proliferation, positive regulation of translational elongation, positive regulation of translational termination	82.18	30.13
EIF5A2	polyamine homeostasis, positive regulation of cell proliferation, positive regulation of translational elongation, positive regulation of translational termination	15.88	19.37
COL1A2	Rho protein signal transduction, blood coagulation, platelet activation, regulation of blood pressure, regulation of immune response, transforming growth factor beta receptor signaling pathway	30.82	118.39
MYOF	regulation of vascular endothelial growth factor receptor signaling pathway	37.35	111.93
PPIB	positive regulation by host of viral genome replication, positive regulation of multicellular organism growth, protein stabilization, regulation of post-translational protein modification	49.49	77.49
STAT1	JAK-STAT cascade, activation of cysteine-type endopeptidase activity involved in apoptotic process, interferon-gamma-mediated signaling pathway, negative regulation by virus of viral protein levels in host cell, negative regulation of I-kappaB kinase/NF-kappaB signaling, negative regulation of angiogenesis, negati	56.97	64.57
ANXA6	apoptotic signaling pathway, mitochondrial calcium ion homeostasis, regulation of muscle contraction	28.02	114.08
MAP1B	induction of synaptic plasticity by chemical substance, negative regulation of intracellular transport, negative regulation of microtubule depolymerization, positive regulation of axon extension, positive regulation of microtubule polymerization, positive re	20.55	139.91
MVP	ERBB signaling pathway, negative regulation of protein autophosphorylation, negative regulation of protein tyrosine kinase activity, negative regulation of signaling	46.69	96.86
ATP5A1	lipid storage, negative regulation of endothelial cell proliferation, regulation of choline O-acetyltransferase activity	44.83	92.56
MYL9	ephrin receptor signaling pathway, platelet aggregation, regulation of actin cytoskeleton organization, regulation of cell shape, regulation of muscle contraction, regulation of tube length, open tracheal system	40.16	92.56
MYL12B	regulation of cell shape	37.35	73.18
MYL12A	ephrin receptor signaling pathway, platelet aggregation	31.75	73.18
IARS	regulation of translational fidelity	45.76	88.25
TXN	activation of protein kinase B activity, cell redox homeostasis, negative regulation of hydrogen peroxide-induced cell death, negative regulation of protein export from nucleus, negative regulation of transcription from RNA polymerase II promoter, positive regulation of DNA binding, positive regulation of peptidyl-seri	51.36	79.64
RNH1	mRNA catabolic process, negative regulation of catalytic activity, regulation of angiogenesis	42.02	96.86
HSPB1	intracellular signal transduction, negative regulation of apoptotic process, negative regulation of apoptotic signaling pathway, negative regulation of oxidative stress-induced intrinsic apoptotic signaling pathway, negative regulation of protein serine/threonine kinase activity, platelet aggregation, positive regulation of	42.02	94.71
CLIC1	platelet aggregation, positive regulation of osteoblast differentiation, regulation of cell cycle, regulation of ion transmembrane transport, regulation of mitochondrial membrane potential, signal transduction	44.83	64.57
DPYSL2	positive regulation of glutamate secretion, regulation of axon extension, regulation of neuron differentiation, regulation of neuron projection development, signal transduction	42.02	129.15
SND1	gene silencing by RNA, regulation of transcription, DNA-templated	42.02	83.95
FLNB	cytoskeletal anchoring at plasma membrane, signal transduction	40.16	139.91
PRDX6	cell redox homeostasis	48.56	64.57
CCT2	positive regulation of establishment of protein localization to telomere, positive regulation of protein localization to Cajal body, positive regulation of telomerase RNA localization to Cajal body, positive regulation of telomerase activity, positive regulat	34.55	88.25
FSCN1	positive regulation of extracellular matrix disassembly, positive regulation of filopodium assembly, positive regulation of lamellipodium assembly, positive regulation of podosome assembly, regulation of actin cytoskeleton organization, regulation of microv	45.76	66.73

[a]Exponentially Modified Protein Abundance Index (http://www.matrixscience.com/help/quant_empai_help.html).

Table S6. Cont 1.

UniProt/SWISS-Alternate ID	Biological Process<br>biological regulation	emPAI[a]<br>CDM	 DMEM
HYOU1	IRE1-mediated unfolded protein response, negative regulation of apoptotic process, negative regulation of endoplasmic reticulum stress-induced neuron intrinsic apoptotic signaling pathway, negative regulation of hypoxia-induced intrinsic apoptotic signaling pathway	34.55	83.95
IDH1	regulation of phospholipid biosynthetic process, regulation of phospholipid catabolic process	38.29	64.57
IPO5	negative regulation of GTPase activity, positive regulation of protein import into nucleus	31.75	103.32
EPRS	negative regulation of translation, regulation of translation	31.75	73.18
PSAP	adenylate cyclase-inhibiting G-protein coupled receptor signaling pathway, negative regulation of hydrogen peroxide-induced cell death, positive regulation of MAPK cascade, positive regulation of catalytic activity, regulation of MAPK cascade, regulation of autophagy, regulation of lipid metabolic process	38.29	55.96
CTSB	regulation of apoptotic process, regulation of catalytic activity, toll-like receptor signaling pathway	42.02	40.90
HSPA4	negative regulation of apoptotic signaling pathway, negative regulation of establishment of protein localization to mitochondrion, negative regulation of intrinsic apoptotic signaling pathway in response to hydrogen peroxide, negative regulation of p38MAPK	31.75	73.18
ACTR3	Arp2/3 complex-mediated actin nucleation, Fc-gamma receptor signaling pathway involved in phagocytosis, ephrin receptor signaling pathway, positive regulation of Notch signaling pathway, positive regulation of filopodium assembly, positive regulation of lamellipodium assembly	31.75	58.12
AARS	endoplasmic reticulum unfolded protein response, negative regulation of neuron apoptotic process, regulation of translational fidelity	28.02	86.10
TXNDC5	cell redox homeostasis, negative regulation of apoptotic process	35.49	49.51
PDIA6	IRE1-mediated unfolded protein response, cell redox homeostasis, platelet aggregation	37.35	60.27
PRKDC	intrinsic apoptotic signaling pathway in response to DNA damage, negative regulation of apoptotic process, negative regulation of cellular senescence, negative regulation of immunoglobulin production, negative regulation of protein phosphorylation, negativ	51.36	25.83
UCHL1	negative regulation of MAP kinase activity, regulation of macroautophagy	28.95	53.81
CALU	negative regulation of catalytic activity	28.95	53.81
CCT7	positive regulation of establishment of protein localization to telomere, positive regulation of protein localization to Cajal body, positive regulation of telomerase RNA localization to Cajal body, positive regulation of telomere maintenance via telomerase,	28.02	75.34
CCT6A	positive regulation of establishment of protein localization to telomere, positive regulation of protein localization to Cajal body, positive regulation of telomerase RNA localization to Cajal body, positive regulation of telomere maintenance via telomerase,	38.29	64.57
HSPA9	erythrocyte differentiation, negative regulation of apoptotic process, negative regulation of erythrocyte differentiation	28.95	68.88
MME	angiotensin maturation	31.75	62.42
RRBP1	signal transduction	22.41	77.49
TGFBI	negative regulation of cell adhesion	39.22	36.59
GNB2	G-protein coupled acetylcholine receptor signaling pathway, Ras protein signal transduction, Wnt signaling pathway, calcium modulating pathway, adenylate cyclase-activating dopamine receptor signaling pathway, phospholipase C-activating G-protein coupled receptor signaling pathway, platelet activation, positive	25.21	73.18
GNB1	G-protein coupled acetylcholine receptor signaling pathway, Ras protein signal transduction, Wnt signaling pathway, calcium modulating pathway, adenylate cyclase-activating dopamine receptor signaling pathway, phospholipase C-activating G-protein coupled receptor signaling pathway, platelet activation, positive	12.14	38.74
GNB4	signal transduction	4.67	10.76
PARK7	Ras protein signal transduction, activation of protein kinase B activity, dopamine uptake involved in synaptic transmission, membrane depolarization, membrane hyperpolarization, negative regulation of TRAIL-activated apoptotic signaling pathway, negative reg	30.82	51.66
BASP1	negative regulation of transcription, DNA-templated, positive regulation of heart growth, positive regulation of metanephric ureteric bud development	28.02	47.35
FAP	mitotic cell cycle arrest, negative regulation of cell proliferation involved in contact inhibition, negative regulation of extracellular matrix disassembly, negative regulation of extracellular matrix organization, positive regulation of cell cycle arrest,	19.61	83.95
UGGT1	endoplasmic reticulum unfolded protein response	25.21	66.73
VAT1	negative regulation of mitochondrial fusion	26.15	66.73
GPI	erythrocyte homeostasis, glucose homeostasis, hemostasis, negative regulation of cysteine-type endopeptidase activity involved in apoptotic process, negative regulation of neuron apoptotic process	41.09	40.90
CCT5	positive regulation of establishment of protein localization to telomere, positive regulation of protein localization to Cajal body, positive regulation of telomerase RNA localization to Cajal body, positive regulation of telomere maintenance via telomerase,	29.88	58.12
XRCC5	negative regulation of t-circle formation, negative regulation of transcription, DNA-templated, positive regulation of neurogenesis, positive regulation of type I interferon production, regulation of smooth muscle cell proliferation, regulation of transcription, DNA-templated, telomere maintenance	34.55	43.05
CAPG	barbed-end actin filament capping, positive regulation of podosome assembly	37.35	36.59
PRDX1	cell redox homeostasis, erythrocyte homeostasis, regulation of NF-kappaB import into nucleus, regulation of stress-activated MAPK cascade, retina homeostasis	38.29	60.27
COPA	pancreatic juice secretion	21.48	66.73
G6PD	erythrocyte maturation, negative regulation of cell growth involved in cardiac muscle cell development, negative regulation of protein glutathionylation, negative regulation of reactive oxygen species metabolic process, positive regulation of calcium ion transmembrane transport via high voltage-gated calcium chann	24.28	60.27
PSMD2	Fc-epsilon receptor signaling pathway, MAPK cascade, NIK/NF-kappaB signaling, T cell receptor signaling pathway, Wnt signaling pathway, planar cell polarity pathway, negative regulation of canonical Wnt signaling pathway, negative regulation of ubiquitin-protein ligase activity involved in mitotic cell cycle, positive	26.15	58.12
RPSA	nuclear-transcribed mRNA catabolic process, nonsense-mediated decay	28.02	51.66
CLIC4	negative regulation of cell migration, regulation of cytoskeleton organization, regulation of ion transmembrane transport, vacuolar acidification	19.61	71.03
CNN3	negative regulation of ATPase activity	22.41	81.79
LRPPRC	negative regulation of mitochondrial RNA catabolic process, regulation of mitochondrial translation, regulation of transcription, DNA-templated	32.69	34.44
RACK1	activation of cysteine-type endopeptidase activity involved in apoptotic process, cellular response to glucose stimulus, negative regulation of Wnt signaling pathway, negative regulation of cell growth, negative regulation of endoplasmic reticulum unfolded protein response, negative regulation of gene expression, ne	21.48	71.03
PSMD1	Fc-epsilon receptor signaling pathway, MAPK cascade, NIK/NF-kappaB signaling, T cell receptor signaling pathway, Wnt signaling pathway, planar cell polarity pathway, negative regulation of canonical Wnt signaling pathway, negative regulation of ubiquitin-protein ligase activity involved in mitotic cell cycle, positive	24.28	60.27
PPP2R1A	inactivation of MAPK activity, negative regulation of cell growth, negative regulation of tyrosine phosphorylation of Stat3 protein, nuclear-transcribed mRNA catabolic process, nonsense-mediated decay, positive regulation of extrinsic apoptotic signaling pathway in absence of ligand, regulation of DNA replication, re	33.62	38.74
CAPNS1	positive regulation of cell proliferation, regulation of macroautophagy	28.02	47.35
GDI2	positive regulation of GTPase activity, regulation of small GTPase mediated signal transduction, signal transduction, small GTPase mediated signal transduction	45.76	81.79
GNAI2	G-protein coupled acetylcholine receptor signaling pathway, activation of MAPKK activity, adaptation of signaling pathway by response to pheromone involved in conjugation with cellular fusion, adenosine receptor signaling pathway, adenylate cyclase-activating G-protein coupled receptor signaling pathway, adenyl	26.15	55.96
GNAS	adenylate cyclase-activating dopamine receptor signaling pathway, genetic imprinting, platelet aggregation, positive regulation of osteoblast differentiation, positive regulation of osteoclast differentiation, regulation of parathyroid hormone secretion, tissue homeostasis	1.87	17.22
ITGB1	integrin-mediated signaling pathway, negative regulation of Rho protein signal transduction, negative regulation of anoikis, negative regulation of cell differentiation, positive regulation of GTPase activity, positive regulation of apoptotic process, posi	24.28	55.96
MAPK1	B cell receptor signaling pathway, BMP signaling pathway, ERBB signaling pathway, ERK1 and ERK2 cascade, Fc-epsilon receptor signaling pathway, Fc-gamma receptor signaling pathway involved in phagocytosis, G-protein coupled receptor signaling pathway, JAK-STAT cascade involved i	22.41	34.44
MAPK3	BMP signaling pathway, ERK1 and ERK2 cascade, Fc-epsilon receptor signaling pathway, Fc-gamma receptor signaling pathway involved in phagocytosis, JAK-STAT cascade involved in growth hormone signaling pathway, MAPK import into nucleus, activation of MAPK activity, fibroblast growt	5.60	10.76
PDCD6IP	positive regulation of exosomal secretion, positive regulation of extracellular exosome assembly, regulation of centrosome duplication, regulation of extracellular exosome assembly, regulation of mitotic spindle assembly	28.95	45.20
WARS	negative regulation of cell proliferation, regulation of angiogenesis	13.07	73.18
PPP1CA	beta-catenin destruction complex disassembly, circadian regulation of gene expression, entrainment of circadian clock by photoperiod, negative regulation of protein binding, positive regulation of establishment of cell polarity regulating cell shape, positive regulation of extrinsic apoptotic signaling pathway in absen	30.82	27.98
PPP1CB	circadian regulation of gene expression, entrainment of circadian clock by photoperiod, regulation of cell adhesion, regulation of glycogen biosynthetic process, regulation of glycogen catabolic process	25.21	25.83
RAB10	bone resorption, fibroblast growth factor receptor signaling pathway, growth hormone secretion, positive regulation of dopamine uptake involved in synaptic transmission, positive regulation of glycoprotein metabolic process, positive regulation of regulate	15.88	64.57
RAB35	small GTPase mediated signal transduction	5.60	12.91
RAB1B	positive regulation of glycoprotein metabolic process, regulation of autophagosome assembly, small GTPase mediated signal transduction	6.54	27.98
RAB1A	growth hormone secretion, positive regulation of glycoprotein metabolic process, small GTPase mediated signal transduction	5.60	32.29
SPTAN1	MAPK cascade, actin filament capping, positive regulation of GTPase activity	19.61	51.66
CCT4	positive regulation of establishment of protein localization to telomere, positive regulation of protein localization to Cajal body, positive regulation of telomerase RNA localization to Cajal body, positive regulation of telomerase activity, positive regulat	25.21	47.35
ACTR2	Arp2/3 complex-mediated actin nucleation, Fc-gamma receptor signaling pathway involved in phagocytosis, ephrin receptor signaling pathway	29.88	40.90
ARPC2	Arp2/3 complex-mediated actin nucleation, Fc-gamma receptor signaling pathway involved in phagocytosis, ephrin receptor signaling pathway, positive regulation of lamellipodium assembly, positive regulation of substrate adhesion-dependent cell spreading	25.21	45.20
CNN2	regulation of actin filament-based process	18.68	77.49
GCN1	positive regulation of kinase activity, positive regulation of transcription from RNA polymerase II promoter in response to stress, regulation of protein kinase activity, regulation of translation	23.35	55.96
P3H1	negative regulation of cell proliferation, negative regulation of post-translational protein modification, protein stabilization, regulation of protein secretion	23.35	47.35
MIF	DNA damage response, signal transduction by p53 class mediator, cell surface receptor signaling pathway, negative regulation of DNA damage response, signal transduction by p53 class mediator, negative regulation of apoptotic process, negative regulation of cell aging, negative re	31.75	30.13
FAM129B	negative regulation of apoptotic process	19.61	60.27
VPS35	Wnt signaling pathway, negative regulation of late endosome to lysosome transport, positive regulation of Wnt protein secretion, positive regulation of canonical Wnt signaling pathway, positive regulation of dopamine receptor signaling pathway, regulation of macroautophagy, regulation of protein stability	19.61	62.42
ATP6V1A	endosomal lumen acidification, insulin receptor signaling pathway, lumen formation, open tracheal system, phagosome acidification, regulation of macroautophagy	10.27	66.73
VDAC1	negative regulation of reactive oxygen species metabolic process, regulation of anion transmembrane transport, regulation of anion transport, regulation of mitophagy	8.40	90.40
AP2A1	Wnt signaling pathway, planar cell polarity pathway, ephrin receptor signaling pathway, negative regulation of epidermal growth factor receptor signaling pathway, negative regulation of hyaluronan biosynthetic process, neurotransmitter secretion, positive regulation of endocytosis, regulation of defense response to	11.21	66.73
AP2A2	Wnt signaling pathway, planar cell polarity pathway, ephrin receptor signaling pathway, negative regulation of epidermal growth factor receptor signaling pathway, regulation of defense response to virus by virus	4.67	27.98
HNRNPK	negative regulation of apoptotic process, negative regulation of mRNA splicing, via spliceosome, positive regulation of low-density lipoprotein particle receptor biosynthetic process, positive regulation of receptor-mediated endocytosis, regulation of intrinsic apoptotic signaling pathway in response to DNA damage b	25.21	38.74
PYGB	glucose homeostasis	16.81	38.74
PYGL	glucose homeostasis	6.54	8.61
TCP1	positive regulation of establishment of protein localization to telomere, positive regulation of protein localization to Cajal body, positive regulation of telomerase RNA localization to Cajal body, positive regulation of telomerase activity, positive regulat	21.48	51.66
ZYX	integrin-mediated signaling pathway, regulation of inflammatory response, signal transduction, transforming growth factor beta receptor signaling pathway	21.48	45.20
MAP4	negative regulation of non-motile cilium assembly	10.27	68.88
TNC	negative regulation of cell adhesion, positive regulation of cell proliferation, positive regulation of gene expression	5.60	75.34
PHGDH	regulation of gene expression	1.87	77.49
PDIA4	cell redox homeostasis	26.15	32.29
SEC31A	IRE1-mediated unfolded protein response	16.81	49.51
XRCC6	negative regulation of transcription, DNA-templated, positive regulation of transcription from RNA polymerase II promoter, positive regulation of transcription, DNA-templated, positive regulation of type I interferon production, regulation of smooth muscle cell proliferation, telomere maintenance	32.69	19.37
AP2B1	Wnt signaling pathway, planar cell polarity pathway, ephrin receptor signaling pathway, negative regulation of epidermal growth factor receptor signaling pathway, regulation of defense response to virus by virus	20.55	62.42
GSTO1	negative regulation of ryanodine-sensitive calcium-release channel activity, positive regulation of ryanodine-sensitive calcium-release channel activity, positive regulation of skeletal muscle contraction by regulation of release of sequestered calcium ion, regulation of cardiac muscle contraction by regulation of the	26.15	30.13
LMO7	regulation of cell adhesion, regulation of signaling	0.93	86.10
NPM1	DNA damage response, signal transduction by p53 class mediator resulting in cell cycle arrest, negative regulation of apoptotic process, negative regulation of cell proliferation, negative regulation of centrosome duplication, negative regulation of protein kinase activity by	26.15	27.98
CCT8	positive regulation of establishment of protein localization to telomere, positive regulation of protein localization to Cajal body, positive regulation of telomerase RNA localization to Cajal body, positive regulation of telomere maintenance via telomerase,	15.88	43.05
CAND1	negative regulation of catalytic activity, positive regulation of RNA polymerase II transcriptional preinitiation complex assembly, positive regulation of transcription, DNA-templated	18.68	38.74
PEBP1	MAPK cascade, negative regulation of endopeptidase activity	20.55	38.74
RPL9	nuclear-transcribed mRNA catabolic process, nonsense-mediated decay	18.68	49.51
RPS3	negative regulation of DNA repair, negative regulation of protein ubiquitination, negative regulation of translation, nuclear-transcribed mRNA catabolic process, nonsense-mediated decay, positive regulation of DNA N-glycosylase activity, positive regulatio	15.88	51.66
RPL12	nuclear-transcribed mRNA catabolic process, nonsense-mediated decay	28.02	25.83
RPS7	nuclear-transcribed mRNA catabolic process, nonsense-mediated decay, regulation of cell cycle	26.15	27.98
CAPN1	positive regulation of cell proliferation, regulation of macroautophagy	20.55	34.44
IPO7	regulation of catalytic activity, signal transduction	18.68	36.59
PRDX4	I-kappaB phosphorylation, cell redox homeostasis, negative regulation of male germ cell proliferation	20.55	60.27
RHOA	Rho protein signal transduction, Roundabout signaling pathway, Wnt signaling pathway, planar cell polarity pathway, apolipoprotein A-I-mediated signaling pathway, ephrin receptor signaling pathway, negative regulation of axonogenesis, negative regulation of cell migration	21.48	34.44
RHOC	positive regulation of I-kappaB kinase/NF-kappaB signaling, positive regulation of lipase activity, regulation of small GTPase mediated signal transduction, small GTPase mediated signal transduction	16.81	25.83
VARS	regulation of translational fidelity	22.41	34.44

[a]Exponentially Modified Protein Abundance Index (http://www.matrixscience.com/help/quant_empai_help.html).

Table S6. Cont 2.

UniProt/SWISS-<br>Alternate ID	Biological Process<br>biological regulation	emPAI[a]<br>CDM	 DMEM
RPL7A	nuclear-transcribed mRNA catabolic process, nonsense-mediated decay	24.28	30.13
CTSK	bone resorption, positive regulation of protein targeting to mitochondrion, regulation of mitophagy, toll-like receptor signaling pathway	28.95	12.91
FERMT2	Wnt signaling pathway, integrin-mediated signaling pathway, regulation of cell shape, transforming growth factor beta receptor signaling pathway	13.07	49.51
GLG1	negative regulation of protein processing, negative regulation of transforming growth factor beta receptor signaling pathway, regulation of chondrocyte differentiation	14.01	36.59
MYO1C	Fc-gamma receptor signaling pathway involved in phagocytosis, positive regulation of actin filament polymerization, positive regulation of cell migration, positive regulation of cell migration by vascular endothelial growth factor signaling pathway, positiv	17.74	40.90
NME1	cellular response to glucose stimulus, negative regulation of cell proliferation, negative regulation of gene expression, negative regulation of myeloid leukocyte differentiation, positive regulation of DNA binding, positive regulation of epithelial cell proliferation, positive regulation of neuron projection development, reg	48.56	36.59
PRDX5	cell redox homeostasis, negative regulation of apoptotic process, negative regulation of cysteine-type endopeptidase activity involved in apoptotic process, negative regulation of oxidoreductase activity, negative regulation of transcription from RNA polymerase III promoter, positive regulation of collagen biosynthe	14.01	49.51
RAP1B	Rap protein signal transduction, activation of MAPKK activity, cellular response to glucose stimulus, negative regulation of calcium ion-dependent exocytosis, negative regulation of collagen biosynthetic process, negative regulation of synaptic vesicle exocytosis, nerve	22.41	32.29
RAP1A	Rap protein signal transduction, activation of MAPKK activity, cellular response to glucose stimulus, negative regulation of collagen biosynthetic process, negative regulation of synaptic vesicle exocytosis, nerve growth factor signaling pathway, positive regulation of ERK1	16.81	25.83
SPTBN1	MAPK cascade, SMAD protein import into nucleus, actin filament capping, common-partner SMAD protein phosphorylation, positive regulation of GTPase activity, positive regulation of interleukin-2 secretion, positive regulation of protein localization to plasm	14.94	38.74
RNPEP	negative regulation of blood pressure	20.55	21.52
ARHGAP1	Rho protein signal transduction, positive regulation of GTPase activity, positive regulation of signal transduction, regulation of small GTPase mediated signal transduction, small GTPase mediated signal transduction	15.88	36.59
SPARC	negative regulation of angiogenesis, negative regulation of endothelial cell proliferation, positive regulation of endothelial cell migration, regulation of cell morphogenesis, regulation of cell proliferation, signal transduction	14.94	43.05
ATP1A1	cell communication by electrical coupling involved in cardiac conduction, cellular potassium ion homeostasis, cellular sodium ion homeostasis, membrane hyperpolarization, membrane repolarization during cardiac muscle cell action potential, negative regulatio	25.21	17.22
ARHGDIA	negative regulation of apoptotic process, negative regulation of axonogenesis, negative regulation of cell adhesion, positive regulation of GTPase activity, positive regulation of axonogenesis, regulation of Rho protein signal transduction, regulation of a	16.81	30.13
NAP1L1	positive regulation of cell proliferation	16.81	32.29
PTBP1	fibroblast growth factor receptor signaling pathway, negative regulation of mRNA splicing, via spliceosome, negative regulation of muscle cell differentiation, regulation of alternative mRNA splicing, via spliceosome	13.07	49.51
CCT3	positive regulation of protein localization to Cajal body, positive regulation of telomerase RNA localization to Cajal body, positive regulation of telomere maintenance via telomerase, protein stabilization	16.81	38.74
ATP6V1B2	insulin receptor signaling pathway, phagosome acidification, regulation of macroautophagy	11.21	49.51
VDAC2	negative regulation of intrinsic apoptotic signaling pathway, negative regulation of protein polymerization, regulation of anion transmembrane transport, regulation of anion transport	6.54	58.12
PCBP1	positive regulation of transcription from RNA polymerase II promoter	29.88	49.51
RPS3A	negative regulation of apoptotic process, nuclear-transcribed mRNA catabolic process, nonsense-mediated decay	6.54	43.05
TXNRD1	cell redox homeostasis, signal transduction	2.80	66.73
ADH5	positive regulation of blood pressure	17.74	32.29
COMT	estrogen metabolic process, negative regulation of dopamine metabolic process, negative regulation of renal sodium excretion, negative regulation of smooth muscle cell proliferation, neurotransmitter catabolic process, positive regulation of homocysteine metabolic process, regulation of sensory perception of pai	14.01	34.44
NME2	cellular response to glucose stimulus, integrin-mediated signaling pathway, negative regulation of apoptotic process, negative regulation of myeloid leukocyte differentiation, positive regulation of cAMP biosynthetic process, positive regulation of epithelial cell proliferation, positive regulation of keratinocyte different	45.76	36.59
PLOD3	vasodilation	13.07	47.35
PSMA4	Fc-epsilon receptor signaling pathway, MAPK cascade, NIK/NF-kappaB signaling, T cell receptor signaling pathway, Wnt signaling pathway, planar cell polarity pathway, negative regulation of canonical Wnt signaling pathway, negative regulation of ubiquitin-protein ligase activity involved in mitotic cell cycle, positive	15.88	38.74
SERPINB6	negative regulation of endopeptidase activity	5.60	55.96
SLC25A6	active induction of host immune response by virus, negative regulation of mitochondrial outer membrane permeabilization involved in apoptotic signaling pathway, positive regulation of cell proliferation, regulation of insulin secretion	14.94	25.83
SLC25A5	negative regulation of mitochondrial outer membrane permeabilization involved in apoptotic signaling pathway, positive regulation of cell proliferation, regulation of insulin secretion	9.34	15.07
ARPC1B	Arp2/3 complex-mediated actin nucleation, Fc-gamma receptor signaling pathway involved in phagocytosis, ephrin receptor signaling pathway, regulation of actin filament polymerization	15.88	38.74
C1QBP	blood coagulation, intrinsic pathway, complement activation, classical pathway, negative regulation of MDA-5 signaling pathway, negative regulation of RIG-I signaling pathway, negative regulation of defense response to virus, negative regulation of interferon-gamma production, negative regulation of interleukin-12 p	18.68	25.83
CSRP1	platelet aggregation	5.60	47.35
GFPT1	IRE1-mediated unfolded protein response, circadian regulation of gene expression	15.88	47.35
EIF4G1	nuclear-transcribed mRNA catabolic process, nonsense-mediated decay, nuclear-transcribed mRNA poly(A) tail shortening, positive regulation of neuron differentiation, regulation of mRNA stability, regulation of translation, regulation of translational initiation	21.48	17.22
RAB5C	blood coagulation, positive regulation of exocytosis, receptor internalization involved in canonical Wnt signaling pathway, regulation of autophagosome assembly, regulation of endocytosis, regulation of endosome size, regulation of filopodium assembly, regul	9.34	43.05
CD63	positive regulation of integrin-mediated signaling pathway, positive regulation of receptor internalization, regulation of rubidium ion transport, regulation of vascular endothelial growth factor signaling pathway	20.55	12.91
ATP2A2	ER-nucleus signaling pathway, cellular calcium ion homeostasis, negative regulation of heart contraction, negative regulation of striated muscle contraction, positive regulation of endoplasmic reticulum calcium ion concentration, positive regulation of fast	7.47	45.20
CORO1C	activation of GTPase activity, negative regulation of epithelial cell migration, negative regulation of focal adhesion assembly, negative regulation of protein kinase activity by regulation of protein phosphorylation, negative regulation of substrate adhesion-dependent cell spreading, positive regulation of lamellipodium	14.94	25.83
DDX3X	Wnt signaling pathway, extrinsic apoptotic signaling pathway via death domain receptors, intracellular signal transduction, intrinsic apoptotic signaling pathway, negative regulation of apoptotic process, negative regulation of cell growth, negative regulation of cysteine-type endopeptidase activity involved in apoptoti	18.68	23.68
DBN1	maintenance of protein location in cell, regulation of dendrite development, regulation of neuronal synaptic plasticity	12.14	36.59
PAPSS2	blood coagulation	14.01	32.29
RAN	androgen receptor signaling pathway, gene silencing by RNA, positive regulation of protein binding, positive regulation of transcription, DNA-templated, pre-miRNA export from nucleus, signal transduction, small GTPase mediated signal transduction	15.88	25.83
RPL4	nuclear-transcribed mRNA catabolic process, nonsense-mediated decay	9.34	38.74
TMSB4X	sequestering of actin monomers	17.74	17.22
EEF1D	positive regulation of I-kappaB kinase/NF-kappaB signaling, regulation of cell death, regulation of transcription, DNA-templated, signal transduction	19.61	34.44
FH	homeostasis of number of cells within a tissue	14.01	32.29
LAMC1	positive regulation of epithelial cell proliferation	10.27	40.90
NCL	positive regulation of transcription from RNA polymerase II promoter, positive regulation of transcription of nuclear large rRNA transcript from RNA polymerase I promoter	12.14	34.44
RPL5	nuclear-transcribed mRNA catabolic process, nonsense-mediated decay	9.34	30.13
RPS2	nuclear-transcribed mRNA catabolic process, nonsense-mediated decay, positive regulation of transferase activity	15.88	23.68
TPM2	regulation of ATPase activity	20.55	133.45
COL3A1	integrin-mediated signaling pathway, negative regulation of immune response, negative regulation of neuron migration, platelet activation, positive regulation of Rho protein signal transduction, regulation of immune response, transforming growth factor beta receptor signaling pathway	17.74	21.52
PRKCSH	intracellular signal transduction	14.01	30.13
MRC2	signal transduction	8.40	40.90
PSME2	Fc-epsilon receptor signaling pathway, MAPK cascade, NIK/NF-kappaB signaling, T cell receptor signaling pathway, Wnt signaling pathway, planar cell polarity pathway, negative regulation of canonical Wnt signaling pathway, negative regulation of ubiquitin-protein ligase activity involved in mitotic cell cycle, positive	20.55	12.91
RPS5	nuclear-transcribed mRNA catabolic process, nonsense-mediated decay, regulation of translational fidelity	16.81	23.68
SDCBP	Ras protein signal transduction, ephrin receptor signaling pathway, negative regulation of proteasomal ubiquitin-dependent protein catabolic process, negative regulation of receptor internalization, positive regulation of JNK cascade, positive regulation of	14.94	30.13
YBX1	CRD-mediated mRNA stabilization, negative regulation of striated muscle cell differentiation, negative regulation of transcription from RNA polymerase II promoter, positive regulation of cell division, positive regulation of transcription from RNA polymeras	17.74	25.83
LAMB1	positive regulation of cell migration, positive regulation of epithelial cell proliferation	25.21	4.31
AKR1B1	C21-steroid hormone biosynthetic process, positive regulation of JAK-STAT cascade, positive regulation of smooth muscle cell proliferation, stress-activated protein kinase signaling cascade, tissue homeostasis	16.81	25.83
DPYSL3	negative regulation of cell migration, negative regulation of neuron projection development, positive regulation of filopodium assembly, positive regulation of neuron projection development	20.55	64.57
GLS	regulation of respiratory gaseous exchange by neurological system process	6.54	36.59
PCBP2	negative regulation of defense response to virus, negative regulation of type I interferon production	25.21	34.44
RPS8	nuclear-transcribed mRNA catabolic process, nonsense-mediated decay	7.47	34.44
PSMC4	Fc-epsilon receptor signaling pathway, MAPK cascade, NIK/NF-kappaB signaling, T cell receptor signaling pathway, Wnt signaling pathway, planar cell polarity pathway, negative regulation of canonical Wnt signaling pathway, negative regulation of ubiquitin-protein ligase activity involved in mitotic cell cycle, positive	13.07	25.83
RPS15	nuclear-transcribed mRNA catabolic process, nonsense-mediated decay	18.68	19.37
RPS4X	nuclear-transcribed mRNA catabolic process, nonsense-mediated decay, positive regulation of cell proliferation, positive regulation of translation	13.07	27.98
XPO1	negative regulation of transcription from RNA polymerase II promoter, regulation of centrosome duplication, regulation of mRNA stability, regulation of protein catabolic process, regulation of protein export from nucleus	16.81	15.07
ITGAV	ERK1 and ERK2 cascade, apolipoprotein A-I-mediated signaling pathway, entry of symbiont into host cell by promotion of host phagocytosis, extrinsic apoptotic signaling pathway in absence of ligand, integrin-mediated signaling pathway, negative regulation of	3.74	43.05
EIF3E	negative regulation of translational initiation, nuclear-transcribed mRNA catabolic process, nonsense-mediated decay	21.48	10.76
AP1B1	regulation of defense response to virus by virus	18.68	23.68
BZW1	regulation of transcription, DNA-templated	16.81	19.37
EHD2	blood coagulation, positive regulation of endocytic recycling, positive regulation of myoblast fusion	9.34	36.59
ERP29	activation of MAPK activity, negative regulation of gene expression, negative regulation of protein secretion, positive regulation of gene expression, regulation of endoplasmic reticulum stress-induced intrinsic apoptotic signaling pathway	11.21	30.13
EIF2S1	PERK-mediated unfolded protein response, negative regulation of guanyl-nucleotide exchange factor activity, negative regulation of translational initiation in response to stress, positive regulation of defense response to virus by host, positive regulation	10.27	34.44
EIF4H	regulation of translational initiation	14.94	21.52
MAPRE1	negative regulation of microtubule binding, negative regulation of microtubule polymerization, positive regulation of cell migration, positive regulation of microtubule plus-end binding	20.55	12.91
PABPC4	blood coagulation, gene silencing by RNA, mRNA stabilization, negative regulation of nuclear-transcribed mRNA catabolic process, nonsense-mediated decay, negative regulation of nuclear-transcribed mRNA poly(A) tail shortening, nuclear-transcribed mRNA catab	17.74	25.83
PABPC1	gene silencing by RNA, mRNA stabilization, negative regulation of nuclear-transcribed mRNA catabolic process, nonsense-mediated decay, nuclear-transcribed mRNA catabolic process, nonsense-mediated decay, nuclear-transcribed mRNA poly(A) tail shortening, pos	17.74	23.68
PSMA7	Fc-epsilon receptor signaling pathway, MAPK cascade, NIK/NF-kappaB signaling, T cell receptor signaling pathway, Wnt signaling pathway, planar cell polarity pathway, negative regulation of canonical Wnt signaling pathway, negative regulation of ubiquitin-protein ligase activity involved in mitotic cell cycle, positive	14.94	19.37
RPL3	nuclear-transcribed mRNA catabolic process, nonsense-mediated decay	14.94	27.98
YARS	signal transduction	10.27	32.29
HSD17B4	androgen metabolic process, estrogen metabolic process	9.34	32.29
ERO1A	cell redox homeostasis, endoplasmic reticulum unfolded protein response, intrinsic apoptotic signaling pathway in response to endoplasmic reticulum stress, release of sequestered calcium ion into cytosol	17.74	12.91
KPNA6	positive regulation of transcription from RNA polymerase II promoter	14.01	23.68
PSMD3	Fc-epsilon receptor signaling pathway, MAPK cascade, NIK/NF-kappaB signaling, T cell receptor signaling pathway, Wnt signaling pathway, planar cell polarity pathway, negative regulation of canonical Wnt signaling pathway, negative regulation of ubiquitin-protein ligase activity involved in mitotic cell cycle, positive	8.40	36.59
RSU1	positive regulation of GTPase activity, positive regulation of cell-substrate adhesion, signal transduction	13.07	23.68
TMED10	regulation of beta-amyloid formation	12.14	21.52
MTPN	positive regulation of NF-kappaB transcription factor activity, positive regulation of cardiac muscle hypertrophy, positive regulation of cell growth, positive regulation of macromolecule biosynthetic process, positive regulation of protein metabolic process, regulation of barbed-end actin filament capping, regulation o	18.68	10.76
POSTN	negative regulation of cell-matrix adhesion, negative regulation of substrate adhesion-dependent cell spreading, positive regulation of chemokine (C-C motif) ligand 2 secretion, positive regulation of smooth muscle cell migration, regulation of Notch signaling pathway, regulation of systemic arterial blood pressure	14.01	23.68
RAD23B	regulation of proteasomal ubiquitin-dependent protein catabolic process	12.14	23.68
TNPO1	regulation of mRNA stability	20.55	8.61
UBE2L3	positive regulation of protein targeting to mitochondrion, positive regulation of protein ubiquitination, positive regulation of ubiquitin-protein transferase activity, regulation of transcription, DNA-templated	15.88	17.22
CAPZA1	barbed-end actin filament capping, blood coagulation	15.88	23.68
EIF3I	regulation of translational initiation	6.54	30.13
ELOC	positive regulation of transcription elongation from RNA polymerase II promoter, regulation of transcription from RNA polymerase II promoter in response to hypoxia	9.34	25.83
KIF5B	positive regulation of establishment of protein localization to plasma membrane, positive regulation of insulin secretion involved in cellular response to glucose stimulus, positive regulation of intracellular protein transport, positive regulation of potas	6.54	32.29
PSMA6	Fc-epsilon receptor signaling pathway, MAPK cascade, NIK/NF-kappaB signaling, T cell receptor signaling pathway, Wnt signaling pathway, planar cell polarity pathway, negative regulation of canonical Wnt signaling pathway, negative regulation of ubiquitin-protein ligase activity involved in mitotic cell cycle, positive	11.21	23.68

[a]Exponentially Modified Protein Abundance Index (http://www.matrixscience.com/help/quant_empai_help.html).

Table S6. Cont 3.

UniProt/SWISS-Alternate ID	Biological Process<br>biological regulation	emPAI[a]<br>CDM	 DMEM
RAB7A	bone resorption, epidermal growth factor catabolic process, negative regulation of exosomal secretion, negative regulation of intralumenal vesicle formation, phagosome acidification, positive regulation of exosomal secretion, positive regulation of protein catabolic process, positive regulation of viral process, regul	7.47	32.29
RPS10	nuclear-transcribed mRNA catabolic process, nonsense-mediated decay	14.94	10.76
STRAP	maintenance of gastrointestinal epithelium, negative regulation of epithelial cell migration, negative regulation of epithelial cell proliferation, negative regulation of epithelial to mesenchymal transition, negative regulation of pathway-restricted SMAD protein phosphorylation, negative regulation of transcription from R	8.40	30.13
HSPE1	activation of cysteine-type endopeptidase activity involved in apoptotic process	16.81	10.76
DSTN	positive regulation of actin filament depolymerization	11.21	19.37
ETF1	nuclear-transcribed mRNA catabolic process, nonsense-mediated decay, regulation of translational termination	12.14	15.07
GDI1	Rab protein signal transduction, negative regulation of axonogenesis, negative regulation of protein targeting to membrane, positive regulation of GTPase activity, regulation of small GTPase mediated signal transduction, signal transduction	28.95	55.96
PRKACA	activation of protein kinase A activity, adenylate cyclase-modulating G-protein coupled receptor signaling pathway, blood coagulation, calcium-mediated signaling using intracellular calcium source, cell communication by electrical coupling involved in cardiac conduction, cellular response to glucose stimulus, modu	8.40	19.37
PRKACB	activation of protein kinase A activity, adenylate cyclase-modulating G-protein coupled receptor signaling pathway, blood coagulation, negative regulation of meiotic cell cycle, negative regulation of smoothened signaling pathway involved in dorsal/ventral neural tube patterning, regulation of protein processing, rena	8.40	15.07
PSMB2	Fc-epsilon receptor signaling pathway, MAPK cascade, NIK/NF-kappaB signaling, T cell receptor signaling pathway, Wnt signaling pathway, planar cell polarity pathway, negative regulation of canonical Wnt signaling pathway, negative regulation of ubiquitin-protein ligase activity involved in mitotic cell cycle, positive	13.07	15.07
HNRNPA1	fibroblast growth factor receptor signaling pathway, negative regulation of telomere maintenance via telomerase, positive regulation of telomere maintenance via telomerase	8.40	21.52
SEC13	positive regulation of TOR signaling	6.54	27.98
UBE2V1	Fc-epsilon receptor signaling pathway, T cell receptor signaling pathway, negative regulation of neuron apoptotic process, nucleotide-binding oligomerization domain containing signaling pathway, positive regulation of DNA repair, positive regulation of I-ka	9.34	23.68
UBE2V2	negative regulation of neuron apoptotic process, positive regulation of DNA repair, positive regulation of neuron projection development, positive regulation of proteasomal ubiquitin-dependent protein catabolic process, positive regulation of synapse assembly, regulation of DNA repair	6.54	19.37
HINT1	intrinsic apoptotic signaling pathway by p53 class mediator, positive regulation of calcium-mediated signaling, regulation of transcription, DNA-templated	7.47	19.37
CTNNA1	negative regulation of apoptotic process, negative regulation of cell motility, negative regulation of extrinsic apoptotic signaling pathway in absence of ligand, negative regulation of integrin-mediated signaling pathway, negative regulation of neuroblast proliferation, positive regulation of extrinsic apoptotic signaling p	0.93	45.20
ALDH9A1	hormone metabolic process, neurotransmitter biosynthetic process	15.88	10.76
GLUD1	positive regulation of insulin secretion	15.88	4.31
DHX9	CRD-mediated mRNA stabilization, positive regulation of type I interferon production	8.40	27.98
EIF3K	regulation of translational initiation	13.07	19.37
FKBP1A	SMAD protein complex assembly, negative regulation of phosphoprotein phosphatase activity, negative regulation of release of sequestered calcium ion into cytosol, negative regulation of ryanodine-sensitive calcium-release channel activity, positive regulat	10.27	21.52
RALA	Ras protein signal transduction, positive regulation of filopodium assembly, regulation of exocytosis	11.21	15.07
RPL6	nuclear-transcribed mRNA catabolic process, nonsense-mediated decay, regulation of transcription, DNA-templated	9.34	21.52
RPL7	nuclear-transcribed mRNA catabolic process, nonsense-mediated decay	7.47	17.22
LARS	regulation of translational fidelity	8.40	25.83
TPT1	cellular calcium ion homeostasis, negative regulation of apoptotic process, negative regulation of intrinsic apoptotic signaling pathway in response to DNA damage, regulation of apoptotic process	10.27	25.83
USP14	negative regulation of ER-associated ubiquitin-dependent protein catabolic process, negative regulation of endopeptidase activity, regulation of chemotaxis, regulation of proteasomal protein catabolic process	13.07	12.91
ANXA4	Notch signaling pathway, negative regulation of NF-kappaB transcription factor activity, negative regulation of apoptotic process, negative regulation of catalytic activity, negative regulation of interleukin-8 secretion, regulation of transcription from RNA polymerase II promoter,	2.80	40.90
RPL29	nuclear-transcribed mRNA catabolic process, nonsense-mediated decay	7.47	27.98
PRMT5	circadian regulation of gene expression, negative regulation of cell differentiation, negative regulation of nucleic acid-templated transcription, positive regulation of adenylate cyclase-inhibiting dopamine receptor signaling pathway, positive regulation of oligodendrocyte differentiation, regulation of DNA methylation,	12.14	12.91
ARPC4	Arp2/3 complex-mediated actin nucleation, Fc-gamma receptor signaling pathway involved in phagocytosis, actin nucleation, ephrin receptor signaling pathway	10.27	17.22
DCTN1	IRE1-mediated unfolded protein response, positive regulation of intracellular protein transport, positive regulation of microtubule nucleation, positive regulation of microtubule polymerization	9.34	23.68
DDX1	regulation of transcription, DNA-templated, regulation of translational initiation	5.60	32.29
ENG	BMP signaling pathway, negative regulation of cell migration, negative regulation of endothelial cell proliferation, negative regulation of gene expression, negative regulation of nitric-oxide synthase activity, negative regulation of pathway-restricted SMAD protein phosph	1.87	32.29
GSPT1	nuclear-transcribed mRNA catabolic process, nonsense-mediated decay	3.74	17.22
SHMT2	positive regulation of cell proliferation	10.27	21.52
LAMP1	granzyme-mediated apoptotic signaling pathway, positive regulation of natural killer cell degranulation, positive regulation of natural killer cell mediated cytotoxicity, protein stabilization, regulation of organelle transport along microtubule	10.27	17.22
PAK2	Fc-epsilon receptor signaling pathway, MAPK cascade, T cell costimulation, T cell receptor signaling pathway, activation of MAPKKK activity, gamma-aminobutyric acid secretion, neurotransmission, glutamate secretion, neurotransmission, negative regulation of apoptotic process, negative regulation of cell prolife	12.14	10.76
PFKL	negative regulation of insulin secretion	13.07	23.68
PSMC3	Fc-epsilon receptor signaling pathway, MAPK cascade, NIK/NF-kappaB signaling, T cell receptor signaling pathway, Wnt signaling pathway, planar cell polarity pathway, negative regulation of canonical Wnt signaling pathway, negative regulation of nucleic acid-templated transcription, negative regulation of ubiquitin-	3.74	36.59
NPEPPS	positive regulation of protein targeting to mitochondrion	13.07	12.91
CTTN	negative regulation of extrinsic apoptotic signaling pathway, positive regulation of actin filament polymerization, positive regulation of smooth muscle contraction, regulation of axon extension, regulation of mitophagy	7.47	23.68
TPD52L2	regulation of cell proliferation	3.74	32.29
RAP1GDS1	negative regulation of endoplasmic reticulum calcium ion concentration, positive regulation of GTPase activity, positive regulation of mitochondrial calcium ion concentration, small GTPase mediated signal transduction, vascular smooth muscle contraction	3.74	25.83
PSMA5	Fc-epsilon receptor signaling pathway, MAPK cascade, NIK/NF-kappaB signaling, T cell receptor signaling pathway, Wnt signaling pathway, planar cell polarity pathway, negative regulation of canonical Wnt signaling pathway, negative regulation of ubiquitin-protein ligase activity involved in mitotic cell cycle, positive	0.93	38.74
GOT1	Notch signaling pathway, fatty acid homeostasis	4.67	32.29
CD109	negative regulation of endopeptidase activity, negative regulation of keratinocyte proliferation, negative regulation of protein phosphorylation, negative regulation of transforming growth factor beta receptor signaling pathway, negative regulation of wound healing, regulation of keratinocyte differentiation	13.07	12.91
COL5A1	negative regulation of endodermal cell differentiation, regulation of cellular component organization	3.74	34.44
CTHRC1	Wnt signaling pathway, planar cell polarity pathway, negative regulation of canonical Wnt signaling pathway, positive regulation of osteoblast differentiation, positive regulation of osteoblast proliferation, positive regulation of protein binding	6.54	30.13
HEXB	cellular calcium ion homeostasis, lipid storage, positive regulation of transcription from RNA polymerase II promoter, regulation of cell shape, regulation of cellular metabolic process	9.34	17.22
HNRNPM	fibroblast growth factor receptor signaling pathway	9.34	21.52
EIF5B	regulation of translational initiation	11.21	10.76
PAFAH1B2	positive regulation of macroautophagy	14.01	12.91
PSMD14	Fc-epsilon receptor signaling pathway, MAPK cascade, NIK/NF-kappaB signaling, T cell receptor signaling pathway, Wnt signaling pathway, planar cell polarity pathway, negative regulation of canonical Wnt signaling pathway, negative regulation of ubiquitin-protein ligase activity involved in mitotic cell cycle, positive	12.14	10.76
RPL8	nuclear-transcribed mRNA catabolic process, nonsense-mediated decay	10.27	15.07
SEC61A1	IRE1-mediated unfolded protein response	14.94	10.76
SET	negative regulation of catalytic activity, negative regulation of histone acetylation, negative regulation of neuron apoptotic process, negative regulation of transcription, DNA-templated, regulation of mRNA stability, regulation of phosphoprotein phosphatase activity	11.21	12.91
TPM1	negative regulation of cell migration, negative regulation of vascular associated smooth muscle cell migration, negative regulation of vascular smooth muscle cell proliferation, positive regulation of ATPase activity, positive regulation of cell adhesion, positive regulation of heart rate by epinephrine, positive regulatio	5.60	103.32
PSMB5	Fc-epsilon receptor signaling pathway, MAPK cascade, NIK/NF-kappaB signaling, T cell receptor signaling pathway, Wnt signaling pathway, planar cell polarity pathway, negative regulation of canonical Wnt signaling pathway, negative regulation of ubiquitin-protein ligase activity involved in mitotic cell cycle, positive	6.54	27.98
PICALM	iron ion homeostasis, negative regulation of gene expression, negative regulation of metalloendopeptidase activity involved in amyloid precursor protein catabolic process, negative regulation of receptor-mediated endocytosis, positive regulation of asparti	5.60	23.68
AP2M1	Wnt signaling pathway, planar cell polarity pathway, ephrin receptor signaling pathway, negative regulation of epidermal growth factor receptor signaling pathway, negative regulation of protein localization to plasma membrane, regulation of defense response	8.40	12.91
ARPC3	Arp2/3 complex-mediated actin nucleation, Fc-gamma receptor signaling pathway involved in phagocytosis, ephrin receptor signaling pathway, regulation of actin filament polymerization	8.40	19.37
ASNS	PERK-mediated unfolded protein response, negative regulation of apoptotic process, positive regulation of mitotic cell cycle	0.93	36.59
PHB2	negative regulation of apoptotic process, negative regulation of intracellular estrogen receptor signaling pathway, negative regulation of mammary gland epithelial cell proliferation, negative regulation of sequence-specific DNA binding transcription facto	9.34	19.37
PPP1R14B	negative regulation of catalytic activity, regulation of phosphorylation, regulation of protein dephosphorylation	15.88	2.15
PSMA2	Fc-epsilon receptor signaling pathway, MAPK cascade, NIK/NF-kappaB signaling, T cell receptor signaling pathway, Wnt signaling pathway, planar cell polarity pathway, negative regulation of canonical Wnt signaling pathway, negative regulation of ubiquitin-protein ligase activity involved in mitotic cell cycle, positive	14.94	8.61
PSMB4	Fc-epsilon receptor signaling pathway, MAPK cascade, NIK/NF-kappaB signaling, T cell receptor signaling pathway, Wnt signaling pathway, planar cell polarity pathway, negative regulation of canonical Wnt signaling pathway, negative regulation of inflammatory response to antigenic stimulus, negative regulation of u	7.47	17.22
PSMD13	Fc-epsilon receptor signaling pathway, MAPK cascade, NIK/NF-kappaB signaling, T cell receptor signaling pathway, Wnt signaling pathway, planar cell polarity pathway, negative regulation of canonical Wnt signaling pathway, negative regulation of ubiquitin-protein ligase activity involved in mitotic cell cycle, positive	13.07	12.91
CAD	lactation	8.40	15.07
RPL11	nuclear-transcribed mRNA catabolic process, nonsense-mediated decay	8.40	19.37
PSMC2	Fc-epsilon receptor signaling pathway, MAPK cascade, NIK/NF-kappaB signaling, T cell receptor signaling pathway, Wnt signaling pathway, planar cell polarity pathway, negative regulation of canonical Wnt signaling pathway, negative regulation of ubiquitin-protein ligase activity involved in mitotic cell cycle, positive	7.47	23.68
APRT	lactation	10.27	10.76
CDC42	Fc-gamma receptor signaling pathway involved in phagocytosis, T cell costimulation, Wnt signaling pathway, planar cell polarity pathway, blood coagulation, canonical Wnt signaling pathway, ephrin receptor signaling pathway, negative regulation of epidermal growth factor receptor signaling pathway, negative regula	10.27	17.22
LMAN2	positive regulation of phagocytosis	6.54	19.37
MMP14	negative regulation of Notch signaling pathway, negative regulation of focal adhesion assembly, positive regulation of B cell differentiation, positive regulation of cell growth, positive regulation of cell migration, positive regulation of myotube differentiation, positive regulation of peptidase activity	0.93	34.44
PPP2CA	inactivation of MAPK activity, negative regulation of cell growth, negative regulation of epithelial to mesenchymal transition, negative regulation of tyrosine phosphorylation of Stat3 protein, nuclear-transcribed mRNA catabolic process, nonsense-mediated decay, positive regulation of protein serine/threonine kinase	4.67	12.91
NAPA	regulation of synaptic vesicle priming	12.14	10.76
TWF2	barbed-end actin filament capping, negative regulation of actin filament polymerization, positive regulation of axon extension, positive regulation of lamellipodium assembly, positive regulation of neuron projection development, regulation of actin cytoskel	11.21	12.91
RPL15	nuclear-transcribed mRNA catabolic process, nonsense-mediated decay	8.40	17.22
EHD1	blood coagulation, cholesterol homeostasis, low-density lipoprotein particle clearance, positive regulation of cholesterol storage, positive regulation of endocytic recycling, positive regulation of myoblast fusion, positive regulation of voltage-gated cal	5.60	27.98
SH3BGRL3	cell redox homeostasis, positive regulation of GTPase activity, regulation of actin cytoskeleton organization, regulation of actin filament depolymerization, regulation of blood vessel endothelial cell migration	10.27	6.46
DPP4	T cell costimulation, negative regulation of extracellular matrix disassembly, positive regulation of cell proliferation, regulation of cell-cell adhesion mediated by integrin	0.93	36.59
CAPZA2	barbed-end actin filament capping, blood coagulation	7.47	30.13
CDC37	ERBB2 signaling pathway, positive regulation of macromitophagy in response to mitochondrial depolarization, posttranscriptional regulation of gene expression, protein stabilization, regulation of cyclin-dependent protein serine/threonine kinase activity, reg	4.67	21.52
CPNE1	negative regulation of DNA binding, negative regulation of NIK/NF-kappaB signaling, negative regulation of gene expression, positive regulation of neuron differentiation, positive regulation of protein kinase B signaling, positive regulation of tumor necrosis factor-mediated signaling pathway, regulation of I-kappaB kir	12.14	10.76
KHSRP	3'-UTR-mediated mRNA destabilization, mRNA catabolic process, negative regulation of nitric oxide biosynthetic process, positive regulation of mRNA catabolic process, regulation of mRNA stability, regulation of miRNA metabolic process, regulation of transcr	6.54	10.76
HNRNPF	fibroblast growth factor receptor signaling pathway, regulation of RNA splicing	5.60	32.29
LASP1	positive regulation of signal transduction	6.54	23.68
PAFAH1B1	negative regulation of JNK cascade, negative regulation of neuron projection development, positive regulation of axon extension, positive regulation of cellular component organization, positive regulation of cytokine-mediated signaling pathway, positive reg	9.34	12.91
PHB	negative regulation of ERK1 and ERK2 cascade, negative regulation of androgen receptor signaling pathway, negative regulation of cell growth, negative regulation of cell proliferation, negative regulation of glucocorticoid receptor signaling pathway, negat	9.34	12.91
PNP	positive regulation of T cell proliferation, positive regulation of alpha-beta T cell differentiation	4.67	27.98
PPP1R7	positive regulation of protein dephosphorylation, regulation of catalytic activity	10.27	12.91
PSMC5	Fc-epsilon receptor signaling pathway, MAPK cascade, NIK/NF-kappaB signaling, T cell receptor signaling pathway, Wnt signaling pathway, planar cell polarity pathway, negative regulation of canonical Wnt signaling pathway, negative regulation of programmed cell death, negative regulation of transcription, DNA-ter	7.47	17.22
PSMB3	Fc-epsilon receptor signaling pathway, MAPK cascade, NIK/NF-kappaB signaling, T cell receptor signaling pathway, Wnt signaling pathway, planar cell polarity pathway, negative regulation of canonical Wnt signaling pathway, negative regulation of ubiquitin-protein ligase activity involved in mitotic cell cycle, positive	8.40	17.22
RPS17	erythrocyte homeostasis, nuclear-transcribed mRNA catabolic process, nonsense-mediated decay	8.40	19.37
RPL17	nuclear-transcribed mRNA catabolic process, nonsense-mediated decay	7.47	15.07
EIF3M	regulation of translational initiation	6.54	17.22
EIF3L	regulation of translational initiation	6.54	15.07
CCDC47	ER overload response, calcium ion homeostasis	5.60	19.37
UBE2N	Fc-epsilon receptor signaling pathway, T cell receptor signaling pathway, nucleotide-binding oligomerization domain containing signaling pathway, positive regulation of DNA repair, positive regulation of I-kappaB kinase/NF-kappaB signaling, positive regulat	9.34	15.07

[a]Exponentially Modified Protein Abundance Index (http://www.matrixscience.com/help/quant_empai_help.html).

Table S6. Cont 4.

UniProt/SWISS-Alternate ID	Biological Process biological regulation	emPAI[a] CDM	emPAI[a] DMEM
AKAP12	G-protein coupled receptor signaling pathway, positive regulation of cAMP biosynthetic process, positive regulation of protein kinase A signaling, regulation of protein kinase C signaling, signal transduction	7.47	12.91
EIF3F	regulation of translational initiation	10.27	12.91
FHL2	androgen receptor signaling pathway, negative regulation of apoptotic process, negative regulation of transcription from RNA polymerase II promoter, negative regulation of transcription, DNA-templated, positive regulation of transcription, DNA-templated, regulation of transcription from RNA polymerase II promote	3.74	23.68
GBA	negative regulation of MAP kinase activity, negative regulation of inflammatory response, negative regulation of interleukin-6 production, negative regulation of neuron death, negative regulation of protein homooligomerization, positive regulation of macroa	7.47	17.22
GLRX3	cell redox homeostasis, negative regulation of cardiac muscle hypertrophy, regulation of the force of heart contraction	10.27	8.61
SYNCRIP	CRD-mediated mRNA stabilization, negative regulation of translation, regulation of translation	3.74	17.22
ILK	cell cycle arrest, integrin-mediated signaling pathway, negative regulation of apoptotic process, negative regulation of cardiac muscle cell apoptotic process, negative regulation of neural precursor cell proliferation, negative regulation of neuron apoptotic process, negative regulation of protein kinase activity, negat	3.74	23.68
GLO1	negative regulation of apoptotic process, regulation of transcription from RNA polymerase II promoter	9.34	8.61
PCNA	DNA damage response, signal transduction by p53 class mediator resulting in cell cycle arrest, mitotic telomere maintenance via semi-conservative replication, positive regulation of DNA repair, positive regulation of DNA replication, positive regulation of deoxyribonuclease activity, reg	9.34	10.76
PSMB6	Fc-epsilon receptor signaling pathway, MAPK cascade, NIK/NF-kappaB signaling, T cell receptor signaling pathway, Wnt signaling pathway, planar cell polarity pathway, negative regulation of canonical Wnt signaling pathway, negative regulation of ubiquitin-protein ligase activity involved in mitotic cell cycle, positive	8.40	15.07
PSMD7	Fc-epsilon receptor signaling pathway, MAPK cascade, NIK/NF-kappaB signaling, T cell receptor signaling pathway, Wnt signaling pathway, planar cell polarity pathway, negative regulation of canonical Wnt signaling pathway, negative regulation of ubiquitin-protein ligase activity involved in mitotic cell cycle, positive	4.67	19.37
RPL18	nuclear-transcribed mRNA catabolic process, nonsense-mediated decay	1.87	27.98
SURF4	positive regulation of organelle organization	5.60	23.68
TFG	positive regulation of I-kappaB kinase/NF-kappaB signaling, signal transduction	3.74	17.22
TSPO	maintenance of protein location in mitochondrion, negative regulation of ATP metabolic process, negative regulation of glial cell proliferation, negative regulation of mitochondrion organization, negative regulation of mitophagy, negative regulation of nitr	9.34	8.61
SERPINE1	fibrinolysis, negative regulation of cell adhesion mediated by integrin, negative regulation of cell migration, negative regulation of endopeptidase activity, negative regulation of endothelial cell apoptotic process, negative regulation of extrinsic apoptotic signaling pathway via de	4.67	21.52
RPL13	nuclear-transcribed mRNA catabolic process, nonsense-mediated decay	8.40	17.22
FBLN1	blood coagulation, fibrin clot formation, integrin-mediated signaling pathway, negative regulation of ERK1 and ERK2 cascade, negative regulation of cell adhesion, negative regulation of cell motility, negative regulation of protein phosphorylation, negative regulation of stem cell proliferation, negative regulation of sub	4.67	23.68
PPP3CA	Fc-epsilon receptor signaling pathway, Wnt signaling pathway, calcium modulating pathway, calcineurin-NFAT signaling cascade, calcium-mediated signaling, cellular response to glucose stimulus, excitatory postsynaptic potential, modulation of synaptic transmission, negative regulation of chromatin binding, negat	1.87	17.22
CRTAP	negative regulation of post-translational protein modification, protein stabilization	9.34	12.91
CTNND1	Wnt signaling pathway, negative regulation of canonical Wnt signaling pathway, regulation of transcription, DNA-templated	4.67	21.52
DNAJB11	IRE1-mediated unfolded protein response, positive regulation of ATPase activity	6.54	15.07
DNM1L	positive regulation of GTPase activity, positive regulation of apoptotic process, positive regulation of intrinsic apoptotic signaling pathway, positive regulation of mitochondrial fission, positive regulation of protein secretion, positive regulation of r	5.60	17.22
DTD1	regulation of translational fidelity	6.54	12.91
GPNMB	negative regulation of G1/S transition of mitotic cell cycle, negative regulation of T cell proliferation, negative regulation of cell proliferation, negative regulation of cytokine production, negative regulation of neuron death, negative regulation of tumor necrosis factor production, positive regulation of ERK1 and ERK2 c	12.14	4.31
PEA15	DNA damage checkpoint, MAPK cascade, activation of MAPK activity, negative regulation of extrinsic apoptotic signaling pathway via death domain receptors, negative regulation of glucose import, positive regulation of extrinsic apoptotic signaling pathway via death domain rece	6.54	19.37
PRDX2	cell redox homeostasis, negative regulation of apoptotic process	11.21	19.37
PSMB1	Fc-epsilon receptor signaling pathway, MAPK cascade, NIK/NF-kappaB signaling, T cell receptor signaling pathway, Wnt signaling pathway, planar cell polarity pathway, negative regulation of canonical Wnt signaling pathway, negative regulation of ubiquitin-protein ligase activity involved in mitotic cell cycle, positive	6.54	17.22
RTN4	negative regulation of axon extension, negative regulation of axonogenesis, negative regulation of cell growth, regulation of apoptotic process, regulation of branching morphogenesis of a nerve, regulation of cell migration, regulation of nervous system development	1.87	30.13
SCFD1	negative regulation of autophagosome assembly, regulation of ER to Golgi vesicle-mediated transport, regulation of protein transport	8.40	12.91
TXNDC17	tumor necrosis factor-mediated signaling pathway	5.60	21.52
USP5	positive regulation of proteasomal ubiquitin-dependent protein catabolic process	11.21	8.61
RPS15A	nuclear-transcribed mRNA catabolic process, nonsense-mediated decay, positive regulation of cell cycle, positive regulation of cell proliferation	5.60	19.37
MCTS1	positive regulation of cell proliferation, regulation of growth, regulation of transcription, DNA-templated	9.34	12.91
PSMB7	Fc-epsilon receptor signaling pathway, MAPK cascade, NIK/NF-kappaB signaling, T cell receptor signaling pathway, Wnt signaling pathway, planar cell polarity pathway, negative regulation of canonical Wnt signaling pathway, negative regulation of ubiquitin-protein ligase activity involved in mitotic cell cycle, positive	9.34	12.91
PSMC6	Fc-epsilon receptor signaling pathway, MAPK cascade, NIK/NF-kappaB signaling, T cell receptor signaling pathway, Wnt signaling pathway, planar cell polarity pathway, negative regulation of canonical Wnt signaling pathway, negative regulation of ubiquitin-protein ligase activity involved in mitotic cell cycle, positive	7.47	10.76
RAB2A	positive regulation of exocytosis, small GTPase mediated signal transduction	3.74	19.37
ITGB5	integrin-mediated signaling pathway, transforming growth factor beta receptor signaling pathway	3.74	23.68
EIF6	gene silencing by miRNA, miRNA mediated inhibition of translation, positive regulation of translation, regulation of fatty acid biosynthetic process, regulation of glycolytic process, regulation of megakaryocyte differentiation, regulation of reactive oxygen species metabolic process	7.47	10.76
EIF3A	negative regulation of ERK1 and ERK2 cascade, regulation of translational initiation	2.80	30.13
PPP1R12A	negative regulation of catalytic activity, positive regulation of myosin-light-chain-phosphatase activity, positive regulation of transcription from RNA polymerase II promoter, regulation of cell adhesion, regulation of establishment of endothelial barrier, regulation of myosin-light-chain-phosphatase activity, regulation	5.60	15.07
SH3GL1	signal transduction	11.21	4.31
AIMP2	negative regulation of cell proliferation, positive regulation of neuron death, positive regulation of protein ubiquitination	8.40	12.91
CNPY2	enzyme linked receptor protein signaling pathway, negative regulation of gene expression, positive regulation of low-density lipoprotein particle receptor biosynthetic process, regulation of low-density lipoprotein particle clearance	5.60	15.07
CYCS	activation of cysteine-type endopeptidase activity involved in apoptotic process by cytochrome c, intrinsic apoptotic signaling pathway	12.14	4.31
HSD17B12	estrogen biosynthetic process, positive regulation of cell-substrate adhesion	4.67	17.22
HADH	negative regulation of insulin secretion	5.60	15.07
CAST	negative regulation of endopeptidase activity, negative regulation of peptidase activity, negative regulation of type B pancreatic cell apoptotic process	3.74	21.52
MFGE8	positive regulation of apoptotic cell clearance, positive regulation of phagocytosis	4.67	17.22
NACA	regulation of transcription, DNA-templated	4.67	17.22
OTUB1	negative regulation of double-strand break repair, negative regulation of histone H2A K63-linked ubiquitination	6.54	12.91
CTSA	negative regulation of chaperone-mediated autophagy, positive regulation of catalytic activity, regulation of chaperone-mediated autophagy, regulation of protein stability	7.47	15.07
VASP	positive regulation of actin filament polymerization	3.74	12.91
DLD	cell redox homeostasis, regulation of acetyl-CoA biosynthetic process from pyruvate, regulation of membrane potential	4.67	17.22
KPNA4	modulation by virus of host process	9.34	15.07
RPL22	nuclear-transcribed mRNA catabolic process, nonsense-mediated decay	8.40	12.91
FSTL1	BMP signaling pathway	9.34	10.76
STAT2	JAK-STAT cascade, regulation of mitochondrial fission, regulation of protein phosphorylation, regulation of transcription from RNA polymerase II promoter, regulation of type I interferon-mediated signaling pathway, signal transduction, type I interferon signaling pathway	7.47	6.46
ASPH	activation of cysteine-type endopeptidase activity, activation of store-operated calcium channel activity, positive regulation of calcium ion transport into cytosol, positive regulation of intracellular protein transport, positive regulation of proteolysis, positive regulation of ryanodine-sensitive calcium-release channel a	10.27	2.15
ACSL3	positive regulation of Golgi to plasma membrane protein transport, positive regulation of phosphatidylcholine biosynthetic process, positive regulation of secretion, very-low-density lipoprotein particle assembly	11.21	6.46
APEX1	cell redox homeostasis, negative regulation of nucleic acid-templated transcription, negative regulation of smooth muscle cell migration, positive regulation of DNA repair, positive regulation of G1/S transition of mitotic cell cycle, regulation of mRNA stability, regulation of transcription, DNA-templated, telomere mair	12.14	2.15
ARF6	negative regulation of dendrite development, negative regulation of receptor-mediated endocytosis, positive regulation of actin filament polymerization, positive regulation of establishment of protein localization to plasma membrane, regulation of Rac protei	5.60	15.07
DDB1	Wnt signaling pathway, negative regulation of apoptotic process, nucleotide-excision repair, preincision complex stabilization, positive regulation by virus of viral protein levels in host cell, positive regulation of viral genome replication, positive regu	4.67	17.22
EIF3H	regulation of translational initiation	9.34	6.46
ENPP1	cellular phosphate ion homeostasis, negative regulation of cell growth, negative regulation of fat cell differentiation, negative regulation of glucose import, negative regulation of glycogen biosynthetic process, negative regulation of insulin receptor si	6.54	12.91
FLII	regulation of transcription, DNA-templated	7.47	8.61
HEXA	SMAD protein signal transduction, lipid storage	6.54	12.91
LIMA1	negative regulation of actin filament depolymerization	0.93	21.52
LMAN1	blood coagulation, positive regulation of organelle organization	5.60	12.91
POR	negative regulation of cysteine-type endopeptidase activity involved in apoptotic process, negative regulation of lipase activity, positive regulation of cholesterol biosynthetic process, positive regulation of chondrocyte differentiation, positive regulation of monooxygenase activity, positive regulation of smoothened	9.34	6.46
PDLIM1	regulation of transcription, DNA-templated	3.74	19.37
PRDX3	cell redox homeostasis, negative regulation of apoptotic process, negative regulation of cysteine-type endopeptidase activity involved in apoptotic process, negative regulation of kinase activity, positive regulation of NF-kappaB transcription factor activity, positive regulation of cell proliferation, regulation of mitoch	7.47	12.91
PFN2	negative regulation of actin filament polymerization, negative regulation of epithelial cell migration, negative regulation of ruffle assembly, positive regulation of ATPase activity, positive regulation of actin filament polymerization, positive regulation	7.47	12.91
PSMD4	Fc-epsilon receptor signaling pathway, MAPK cascade, NIK/NF-kappaB signaling, T cell receptor signaling pathway, Wnt signaling pathway, planar cell polarity pathway, negative regulation of canonical Wnt signaling pathway, negative regulation of ubiquitin-protein ligase activity involved in mitotic cell cycle, positive	4.67	19.37
SKP1	Fc-epsilon receptor signaling pathway, NIK/NF-kappaB signaling, T cell receptor signaling pathway, Wnt signaling pathway, maintenance of protein location in nucleus, positive regulation of ubiquitin-protein ligase activity involved in regulation of mitotic cell cycle transition, stimulatory C-type lectin receptor signaling	3.74	21.52
TWF1	barbed-end actin filament capping, regulation of actin phosphorylation, sequestering of actin monomers	6.54	12.91
TAX1BP3	Rho protein signal transduction, Wnt signaling pathway, activation of GTPase activity, negative regulation of Wnt signaling pathway, negative regulation of cell proliferation, negative regulation of protein localization to cell surface, regulation of Wnt si	1.87	23.68
UBE2K	free ubiquitin chain polymerization, intrinsic apoptotic signaling pathway in response to endoplasmic reticulum stress, positive regulation of peptidyl-threonine phosphorylation, positive regulation of type I interferon-mediated signaling pathway, regulatio	7.47	12.91
ATP6V1E1	insulin receptor signaling pathway, phagosome acidification, regulation of macroautophagy	5.60	12.91
AP3B1	blood coagulation, positive regulation of NK T cell differentiation	4.67	12.91
CD81	activation of MAPK activity, cell surface receptor signaling pathway, positive regulation of 1-phosphatidylinositol 4-kinase activity, positive regulation of B cell proliferation, positive regulation of cell proliferation, positive regulation of peptidyl-tyrosine phosphorylation, positive regulation of protein catabolic process i	5.60	17.22
RPL21	nuclear-transcribed mRNA catabolic process, nonsense-mediated decay	4.67	19.37
PCOLCE	positive regulation of peptidase activity	9.34	4.31
RAC1	Fc-epsilon receptor signaling pathway, Fc-gamma receptor signaling pathway involved in phagocytosis, G-protein coupled receptor signaling pathway, T cell costimulation, Wnt signaling pathway, planar cell polarity pathway, blood coagulation, bone resorption	7.47	15.07
GPX1	cell redox homeostasis, intrinsic apoptotic signaling pathway in response to oxidative stress, negative regulation of cysteine-type endopeptidase activity involved in apoptotic process, negative regulation of extrinsic apoptotic signaling pathway via death	3.74	12.91
RPL10	nuclear-transcribed mRNA catabolic process, nonsense-mediated decay	7.47	10.76
CTNNB1	Wnt signaling pathway, calcium modulating pathway, androgen receptor signaling pathway, beta-catenin destruction complex disassembly, bone resorption, bundle of His cell-Purkinje myocyte adhesion involved in cell communication, canonical Wnt signaling pathway, canonical Wnt signaling pathway involved in mi	0.93	21.52
CARM1	DNA damage response, signal transduction by p53 class mediator resulting in cell cycle arrest, intracellular estrogen receptor signaling pathway, negative regulation of dendrite development, negative regulation of protein binding, positive regulation of cell proliferation, positi	3.74	17.22
CFL2	muscle cell cellular homeostasis, positive regulation of actin filament depolymerization	24.28	55.96
DDX6	exonucleolytic nuclear-transcribed mRNA catabolic process involved in deadenylation-dependent decay, negative regulation of neuron differentiation, regulation of translation	7.47	6.46
ERP44	cell redox homeostasis	6.54	12.91
HNRNPH1	fibroblast growth factor receptor signaling pathway, regulation of RNA splicing	7.47	25.83
HSPB6	regulation of muscle contraction	1.87	21.52
LIMS1	negative regulation of transcription, DNA-templated, positive regulation of GTPase activity, positive regulation of cell-substrate adhesion, positive regulation of focal adhesion assembly, positive regulation of gene expression, positive regulation of substrate adhesion-dependent cell spreading, regulation of epithelial	6.54	12.91
ME1	regulation of NADP metabolic process	3.74	17.22
PSMD11	Fc-epsilon receptor signaling pathway, MAPK cascade, NIK/NF-kappaB signaling, T cell receptor signaling pathway, Wnt signaling pathway, planar cell polarity pathway, negative regulation of canonical Wnt signaling pathway, negative regulation of ubiquitin-protein ligase activity involved in mitotic cell cycle, positive	3.74	19.37
PSMD12	Fc-epsilon receptor signaling pathway, MAPK cascade, NIK/NF-kappaB signaling, T cell receptor signaling pathway, Wnt signaling pathway, planar cell polarity pathway, negative regulation of canonical Wnt signaling pathway, negative regulation of ubiquitin-protein ligase activity involved in mitotic cell cycle, positive	3.74	12.91
PTK7	canonical Wnt signaling pathway, planar cell polarity pathway involved in neural tube closure, positive regulation of neuron projection development, signal transduction	0.93	21.52
RPL18A	nuclear-transcribed mRNA catabolic process, nonsense-mediated decay	1.87	17.22
TGFB1I1	Wnt signaling pathway, androgen receptor signaling pathway, negative regulation of cell proliferation, negative regulation of transforming growth factor beta receptor signaling pathway, positive regulation of epithelial to mesenchymal transition, positive regulation of transcription, DNA-templated, positive regulation c	1.87	23.68

[a]Exponentially Modified Protein Abundance Index (http://www.matrixscience.com/help/quant_empai_help.html).

Table S6. Cont 5.

UniProt/SWISS-Alternate ID	Biological Process biological regulation	emPAI[a] CDM	emPAI[a] DMEM
TRIM28	Ras protein signal transduction, negative regulation of DNA demethylation, negative regulation of single stranded viral RNA replication via double stranded DNA intermediate, negative regulation of transcription from RNA polymerase II promoter, negative regulation of transcription, DNA-templated,	6.54	10.76
VPS29	Wnt signaling pathway	6.54	8.61
DYNLL1	negative regulation of phosphorylation, positive regulation of protein insertion into mitochondrial membrane involved in apoptotic signaling pathway, regulation of transcription, DNA-templated	5.60	12.91
SSR1	IRE1-mediated unfolded protein response, positive regulation of cell proliferation	4.67	17.22
BAG2	regulation of catalytic activity, regulation of cellular response to heat	8.40	6.46
ANKFY1	positive regulation of pinocytosis	11.21	2.15
DPY30	chromatin silencing at telomere, regulation of transcription, DNA-templated	7.47	10.76
PCK2	cellular response to glucose stimulus, positive regulation of insulin secretion	1.87	23.68
RPL19	nuclear-transcribed mRNA catabolic process, nonsense-mediated decay	4.67	15.07
DNAJA1	androgen receptor signaling pathway, negative regulation of JUN kinase activity, negative regulation of apoptotic process, negative regulation of establishment of protein localization to mitochondrion, negative regulation of nitrosative stress-induced intri	7.47	8.61
ERH	positive regulation of Notch signaling pathway	10.27	2.15
EXOC1	phosphatidylinositol-mediated signaling, positive regulation of protein secretion, regulation of macroautophagy	9.34	2.15
NUTF2	negative regulation of vascular endothelial growth factor production, positive regulation of protein import into nucleus	3.74	17.22
RPL14	nuclear-transcribed mRNA catabolic process, nonsense-mediated decay	2.80	15.07
RPS12	nuclear-transcribed mRNA catabolic process, nonsense-mediated decay	0.93	23.68
RPS20	nuclear-transcribed mRNA catabolic process, nonsense-mediated decay	0.93	23.68
PTGES3	regulation of cellular response to heat, signal transduction, telomere maintenance	3.74	17.22
TMED2	negative regulation of GTPase activity, positive regulation of gene expression	4.67	12.91
TMOD3	erythrocyte development, pointed-end actin filament capping, positive regulation of mitotic cell cycle phase transition	7.47	8.61
DNAJA2	positive regulation of cell proliferation	7.47	8.61
KPNA3	modulation by virus of host process	7.47	17.22
STOML2	T cell receptor signaling pathway, cellular calcium ion homeostasis, positive regulation of cardiolipin metabolic process, positive regulation of mitochondrial DNA replication, positive regulation of mitochondrial membrane potential	3.74	12.91
MACF1	Wnt signaling pathway, positive regulation of Wnt signaling pathway, positive regulation of axon extension, regulation of epithelial cell migration, regulation of focal adhesion assembly, regulation of microtubule-based process	4.67	12.91
PSMD6	Fc-epsilon receptor signaling pathway, MAPK cascade, NIK/NF-kappaB signaling, T cell receptor signaling pathway, Wnt signaling pathway, planar cell polarity pathway, negative regulation of canonical Wnt signaling pathway, negative regulation of ubiquitin-protein ligase activity involved in mitotic cell cycle, positive	7.47	6.46
ANP32B	activation of cysteine-type endopeptidase activity involved in apoptotic process, negative regulation of cell differentiation, positive regulation of protein export from nucleus	5.60	12.91
NT5E	negative regulation of inflammatory response	0.93	19.37
AKR1C1	cholesterol homeostasis, progesterone metabolic process	4.67	12.91
PRMT1	DNA damage response, signal transduction by p53 class mediator resulting in cell cycle arrest, cell surface receptor signaling pathway, negative regulation of megakaryocyte differentiation, positive regulation of erythrocyte differentiation, positive regulation of hemoglobin bios	6.54	8.61
DYNC1LI1	positive regulation of mitotic cell cycle spindle assembly checkpoint	8.40	4.31
FUS	regulation of nucleic acid-templated transcription	7.47	12.91
GNAQ	G-protein coupled acetylcholine receptor signaling pathway, action potential, activation of phospholipase C activity, adenylate cyclase-activating G-protein coupled receptor signaling pathway, blood coagulation, entrainment of circadian clock, glutamate re	3.74	12.91
PRKAR1A	activation of protein kinase A activity, blood coagulation, intracellular signal transduction, negative regulation of activated T cell proliferation, negative regulation of cAMP-dependent protein kinase activity, negative regulation of meiotic nuclear division, negative regulation of protein kinase activity, regulation of prote	5.60	10.76
MATR3	posttranscriptional regulation of gene expression	3.74	12.91
MESD	Wnt signaling pathway	6.54	8.61
PSME1	Fc-epsilon receptor signaling pathway, MAPK cascade, NIK/NF-kappaB signaling, T cell receptor signaling pathway, Wnt signaling pathway, planar cell polarity pathway, negative regulation of canonical Wnt signaling pathway, negative regulation of ubiquitin-protein ligase activity involved in mitotic cell cycle, positive	2.80	15.07
TP53I3	regulation of apoptotic process	2.80	15.07
RPL23A	nuclear-transcribed mRNA catabolic process, nonsense-mediated decay	2.80	15.07
TROVE2	smoothened signaling pathway	6.54	8.61
SOD1	activation of MAPK activity, cellular iron ion homeostasis, muscle cell cellular homeostasis, myeloid cell homeostasis, negative regulation of cholesterol biosynthetic process, negative regulation of neuron apoptotic process, positive regulation of apoptotic process, positive regulation of catalytic activity, positive re	4.67	15.07
SRPRB	IRE1-mediated unfolded protein response, small GTPase mediated signal transduction	2.80	15.07
TARDBP	3'-UTR-mediated mRNA stabilization, negative regulation by host of viral transcription, negative regulation of protein phosphorylation, positive regulation of insulin secretion, positive regulation of transcription from RNA polymerase II promoter, regulatio	5.60	10.76
ANXA7	cellular calcium ion homeostasis, cellular water homeostasis, hemostasis, negative regulation of gene expression, regulation of cell shape	4.67	12.91
P3H3	negative regulation of cell proliferation	3.74	15.07
UBA2	positive regulation of catalytic activity	2.80	17.22
TXNL1	cell redox homeostasis	2.80	15.07
GLRX	cell redox homeostasis, positive regulation of membrane potential, positive regulation of sodium ion transmembrane transporter activity	7.47	6.46
ANP32A	intracellular signal transduction, regulation of apoptotic process, regulation of mRNA stability, regulation of transcription, DNA-templated	2.80	19.37
EIF3B	regulation of translational initiation	1.87	19.37
LGALS3	negative regulation of T cell activation via T cell receptor contact with antigen bound to MHC molecule on antigen presenting cell, negative regulation of T cell receptor signaling pathway, negative regulation of endocytosis, negative regulation of extrinsic apoptotic signaling pathway, negative regulation of immunologi	2.80	15.07
MYO1D	negative regulation of phosphatase activity	3.74	12.91
PARVA	regulation of cell shape	4.67	10.76
SRPRA	IRE1-mediated unfolded protein response	4.67	10.76
TMX3	cell redox homeostasis	3.74	12.91
ST13	negative regulation of protein refolding	2.80	15.07
KDELR2	protein retention in ER lumen	10.27	8.61
YBX3	3'-UTR-mediated mRNA stabilization, negative regulation of apoptotic process, negative regulation of intrinsic apoptotic signaling pathway in response to osmotic stress, negative regulation of necroptotic process, negative regulation of skeletal muscle tiss	5.60	19.37
UBE2D2	Fc-epsilon receptor signaling pathway, T cell receptor signaling pathway, TRIF-dependent toll-like receptor signaling pathway, regulation of transcription from RNA polymerase II promoter in response to hypoxia, stimulatory C-type lectin receptor signaling pathway	8.40	4.31
ARPC1A	Arp2/3 complex-mediated actin nucleation, Fc-gamma receptor signaling pathway involved in phagocytosis, ephrin receptor signaling pathway, regulation of actin filament polymerization	3.74	17.22

[a]Exponentially Modified Protein Abundance Index (http://www.matrixscience.com/help/quant_empai_help.html).

Table S7. Identification of endogenous proteins (Biological Process: metabolic process) in hADSC; group CDM & DMEM.

Table S7. Identification of endogenous proteins (Biological Process: metabolic process) in hADSC; group CDM & DMEM.

UniProt/SWISS-Alternate ID	Biological Process metabolic process	emPAI[a] CDM	emPAI[a] DMEM
MYH9	ATP metabolic process, membrane protein ectodomain proteolysis	300.70	901.88
FLNA	mRNA transcription from RNA polymerase II promoter	223.19	884.66
HSP90AB1	chaperone-mediated autophagy, peptidyl-tyrosine phosphorylation, retrograde protein transport, ER to cytosol, ubiquitin-dependent ERAD pathway	439.85	454.16
HSP90B1	retrograde protein transport, ER to cytosol, ubiquitin-dependent ERAD pathway	87.78	180.81
HSP90AA1	chaperone-mediated autophagy, peptidyl-tyrosine phosphorylation	229.73	165.74
ALB	lipoprotein metabolic process	698.52	47.35
EEF1A1	transcription, DNA-templated, translation, translational elongation	382.88	376.68
HSPA8	ATP metabolic process, chaperone-mediated protein transport involved in chaperone-mediated autophagy, late endosomal microautophagy, mRNA processing, mRNA splicing, via spliceosome, protein targeting to lysosome involved in chaperone-mediated autophagy, proteolysis involved in cellula	303.50	559.64
HSPA1A	ATP metabolic process	15.88	51.66
PKM	canonical glycolysis, glycolytic process, metabolic process, phosphorylation	295.10	434.80
YWHAH	glucocorticoid catabolic process	10.27	30.13
ENO1	canonical glycolysis, gluconeogenesis, glycolytic process, transcription, DNA-templated	254.94	273.36
ENO2	canonical glycolysis, gluconeogenesis, glycolytic process	70.04	27.98
ACTN4	MAPK cascade	135.41	514.44
CLTC	receptor internalization	163.42	243.22
P4HB	lipoprotein biosynthetic process, peptidyl-proline hydroxylation, peptidyl-proline hydroxylation to 4-hydroxy-L-proline	136.34	236.78
LDHA	NAD metabolic process, carbohydrate metabolic process, glycolytic process, lactate metabolic process, oxidation-reduction process, pyruvate metabolic process	107.39	458.48
EEF2	peptidyl-diphthamide biosynthetic process from peptidyl-histidine, translation, translational elongation	162.49	189.42
ALDOA	ATP biosynthetic process, canonical glycolysis, fructose 1,6-bisphosphate metabolic process, fructose metabolic process, gluconeogenesis, glycolytic process	110.19	279.82
ALDOC	canonical glycolysis, fructose 1,6-bisphosphate metabolic process, fructose metabolic process, gluconeogenesis, glycolytic process	17.74	75.34
FN1	peptide cross-linking	97.12	251.84
COL6A3	collagen catabolic process	126.07	180.81
COL1A1	collagen biosynthetic process, collagen catabolic process	76.58	226.00
SERPINH1	collagen biosynthetic process, protein maturation	127.94	159.28
IQGAP1	ERK1 and ERK2 cascade	101.79	191.57
UBA1	cellular protein modification process, modification-dependent protein catabolic process, protein ubiquitination	103.66	163.59
FASN	acetyl-CoA metabolic process, biosynthetic process, fatty acid biosynthetic process, lipid biosynthetic process, long-chain fatty-acyl-CoA biosynthetic process, oxidation-reduction process, pantothenate metabolic process	130.74	96.86
CALR	protein maturation by protein folding	115.80	120.54
ATP5B	ATP metabolic process, ATP synthesis coupled proton transport, generation of precursor metabolites and energy, lipid metabolic process, mitochondrial ATP synthesis coupled proton transport	83.11	101.17
PDIA3	oxidation-reduction process, proteolysis	84.98	142.06
THBS1	peptide cross-linking, protein O-linked fucosylation	82.18	148.52
VCP	ATP metabolic process, ER-associated misfolded protein catabolic process, NADH metabolic process, autophagosome maturation, autophagy, double-strand break repair, error-free translesion synthesis, flavin adenine dinucleotide catabolic process, proteasome-mediated ubiquitin-dependent p	85.92	133.45
ANPEP	peptide catabolic process, proteolysis	100.86	96.86
GSN	amyloid fibril formation, cellular protein metabolic process	89.65	99.01
ANXA5	xenophagy	70.97	159.28
MSN	receptor internalization	56.03	139.91
EZR	receptor internalization	14.94	32.29
HSPD1	isotype switching to IgG isotypes, protein maturation	79.38	81.79
ACLY	acetyl-CoA biosynthetic process, cholesterol biosynthetic process, citrate metabolic process, fatty acid biosynthetic process, lipid biosynthetic process, lipid metabolic process, long-chain fatty-acyl-CoA biosynthetic process, oxaloacetate metabolic process	70.97	105.47
COL6A1	collagen catabolic process	75.64	81.79
GSTP1	glutathione derivative biosynthetic process, glutathione metabolic process, linoleic acid metabolic process, metabolic process, xenobiotic metabolic process	58.83	124.84
EIF4A1	RNA secondary structure unwinding, RNA splicing, mRNA 3'-end processing, mRNA export from nucleus, mRNA splicing, via spliceosome, nuclear-transcribed mRNA catabolic process, nonsense-mediated decay, nuclear-transcribed mRNA poly(A) tail shortening, rRNA processing, selenocysteine metabolic proc	70.97	86.10
EIF4A2	RNA secondary structure unwinding, nuclear-transcribed mRNA poly(A) tail shortening, translation, translational initiation	29.88	38.74
TPI1	canonical glycolysis, gluconeogenesis, glyceraldehyde-3-phosphate biosynthetic process, glyceraldehyde-3-phosphate metabolic process, glycerol catabolic process, glycolytic process, pentose-phosphate shunt	59.77	107.62
TKT	glyceraldehyde-3-phosphate biosynthetic process, pentose-phosphate shunt, pentose-phosphate shunt, non-oxidative branch, xylulose biosynthetic process	70.04	83.95
ARF4	phosphatidylinositol biosynthetic process, protein ADP-ribosylation	79.38	60.27
ARF1	phosphatidylinositol biosynthetic process	46.69	23.68
ARF3	phosphatidylinositol biosynthetic process	40.16	17.22
CAPN2	protein autoprocessing, proteolysis involved in cellular protein catabolic process	75.64	86.10
LDHB	NAD metabolic process, carbohydrate metabolic process, lactate metabolic process, oxidation-reduction process, pyruvate metabolic process	93.39	135.60
LRP1	lipoprotein metabolic process, retinoid metabolic process	46.69	124.84
CKAP4	cellular protein metabolic process	34.55	152.82
GANAB	carbohydrate metabolic process	51.36	105.47
PGAM1	canonical glycolysis, gluconeogenesis, glycolytic process, respiratory burst	60.70	73.18
PGAM4	gluconeogenesis, glycolytic process	32.69	15.07
KPNB1	apoptotic DNA fragmentation	59.77	79.64
CTSD	autophagy, collagen catabolic process, protein catabolic process, proteolysis	40.16	103.32
ANXA1	DNA strand renaturation, peptide cross-linking	51.36	88.25
EIF5A	mRNA export from nucleus, peptidyl-lysine modification to peptidyl-hypusine, translation, translational frameshifting	82.18	30.13
EIF5A2	peptidyl-lysine modification to peptidyl-hypusine, translation, translational elongation, translational frameshifting	15.88	19.37
COL1A2	collagen catabolic process	30.82	118.39
PPIB	protein peptidyl-prolyl isomerization	49.49	77.49
STAT1	transcription, DNA-templated	56.97	64.57
ATP5A1	ATP metabolic process, ATP synthesis coupled proton transport, electron transport chain, lipid metabolic process, mitochondrial ATP synthesis coupled proton transport	44.83	92.56
IARS	isoleucyl-tRNA aminoacylation, translation	45.76	88.25
TXN	glycerol ether metabolic process, nucleobase-containing small molecule interconversion, oxidation-reduction process, protein repair, sulfate assimilation, transcription, DNA-templated	51.36	79.64
RNH1	mRNA catabolic process	42.02	96.86
CLIC1	glutathione metabolic process	44.83	64.57
DPYSL2	nucleobase-containing compound metabolic process	42.02	129.15
SND1	RNA catabolic process, nucleic acid phosphodiester bond hydrolysis, transcription, DNA-templated	42.02	83.95
PRDX6	hydrogen peroxide catabolic process, lipid catabolic process, lipid metabolic process, oxidation-reduction process	48.56	64.57
EEF1G	glutathione metabolic process, translation, translational elongation	44.83	66.73
P4HA1	oxidation-reduction process, peptidyl-proline hydroxylation to 4-hydroxy-L-proline	32.69	83.95
HADHA	cardiolipin acyl-chain remodeling, fatty acid beta-oxidation, fatty acid metabolic process, lipid metabolic process, oxidation-reduction process	42.96	66.73
UGP2	UDP-glucose metabolic process, UDP-glucuronate biosynthetic process, glucose 1-phosphate metabolic process, glycogen biosynthetic process	26.15	71.03
IDH1	2-oxoglutarate metabolic process, NADPH regeneration, glutathione metabolic process, glyoxylate cycle, isocitrate metabolic process, oxidation-reduction process, tricarboxylic acid cycle	38.29	64.57
EPRS	glutamyl-tRNA aminoacylation, prolyl-tRNA aminoacylation, translation	31.75	73.18
PSAP	glycosphingolipid metabolic process, lipid metabolic process, sphingolipid metabolic process	38.29	55.96
CTSB	collagen catabolic process, proteolysis involved in cellular protein catabolic process	42.02	40.90
P4HA2	oxidation-reduction process, peptidyl-proline hydroxylation	32.69	75.34
AARS	alanyl-tRNA aminoacylation, tRNA aminoacylation, tRNA modification, translation	28.02	86.10
PRKDC	DNA recombination, T cell receptor V(D)J recombination, V(D)J recombination, cellular protein modification process, double-strand break repair, double-strand break repair via alternative nonhomologous end joining, immunoglobulin V(D)J recombination, peptidyl-serine phosphorylation, phospho	51.36	25.83
UCHL1	proteasome-mediated ubiquitin-dependent protein catabolic process, protein deubiquitination	28.95	53.81
NNMT	methylation	32.69	62.42
RPN2	protein N-linked glycosylation via asparagine	36.42	49.51
MME	angiotensin maturation, beta-amyloid metabolic process, creatinine metabolic process, proteolysis	31.75	62.42
RRBP1	translation	22.41	77.49
UGDH	UDP-glucose metabolic process, UDP-glucuronate biosynthetic process, carbohydrate metabolic process, glycosaminoglycan biosynthetic process, oxidation-reduction process	28.95	64.57
TGFBI	cellular protein metabolic process	39.22	36.59
PARK7	autophagy, enzyme active site formation via L-cysteine sulfinic acid, glutathione deglycation, glycolate biosynthetic process, glyoxal catabolic process, glyoxal metabolic process, hydrogen peroxide metabolic process, lactate biosynthetic process, methylgly	30.82	51.66
FAP	proteolysis involved in cellular protein catabolic process	19.61	83.95
UGGT1	ER-associated misfolded protein catabolic process, UDP-glucosylation, endoplasmic reticulum mannose trimming, protein N-linked glycosylation via asparagine	25.21	66.73
VAT1	oxidation-reduction process	26.15	66.73
GPI	aldehyde catabolic process, canonical glycolysis, carbohydrate metabolic process, gluconeogenesis, glucose 6-phosphate metabolic process, glycolytic process, methylglyoxal biosynthetic process	41.09	40.90
PFKP	canonical glycolysis, fructose 1,6-bisphosphate metabolic process, fructose 6-phosphate metabolic process, glycolytic process through fructose-6-phosphate, phosphorylation	23.35	81.79
RPN1	protein N-linked glycosylation via asparagine, protein glycosylation	17.74	73.18
XRCC5	DNA recombination, double-strand break repair, double-strand break repair via nonhomologous end joining, telomere maintenance, transcription, DNA-templated	34.55	43.05
PRDX1	hydrogen peroxide catabolic process, oxidation-reduction process, removal of superoxide radicals	38.29	60.27
G6PD	NADPH regeneration, carbohydrate metabolic process, cholesterol biosynthetic process, glucose 6-phosphate metabolic process, glucose metabolic process, glutathione metabolic process, oxidation-reduction process, pentose biosynthetic process, pentose-phosphate shunt, pentose-ph	24.28	60.27
PSMD2	MAPK cascade, anaphase-promoting complex-dependent catabolic process, protein polyubiquitination	26.15	58.12

[a]Exponentially Modified Protein Abundance Index (http://www.matrixscience.com/help/quant_empai_help.html).

Table S7. Cont 1.

UniProt/SWISS-Alternate ID	Biological Process biological regulation	emPAI[a] CDM	emPAI[a] DMEM
RPSA	endonucleolytic cleavage in ITS1 to separate SSU-rRNA from 5.8S rRNA and LSU-rRNA from tricistronic rRNA transcript (SSU-rRNA, 5.8S rRNA, LSU-rRNA), endonucleolytic cleavage to generate mature 3'-end of SSU-rRNA from (SSU-rRNA, 5.8S rRNA, LSU-rRNA), nuclear-transcribed mRNA catabolic process, n	51.66	83.95
CLIC4	glutathione metabolic process	71.03	64.57
LRPPRC	transcription, DNA-templated	34.44	103.32
MDH2	NADH metabolic process, carbohydrate metabolic process, gluconeogenesis, internal protein amino acid acetylation, malate metabolic process, oxaloacetate metabolic process, oxidation-reduction process, tricarboxylic acid cycle	58.12	73.18
COL12A1	collagen catabolic process	111.93	55.96
PSMD1	MAPK cascade, anaphase-promoting complex-dependent catabolic process, protein polyubiquitination	60.27	40.90
PPP2R1A	RNA splicing, ceramide metabolic process, nuclear-transcribed mRNA catabolic process, nonsense-mediated decay, peptidyl-serine dephosphorylation	38.74	73.18
PGD	D-gluconate metabolic process, oxidation-reduction process, pentose biosynthetic process, pentose-phosphate shunt, pentose-phosphate shunt, oxidative branch	34.44	58.12
CAPNS1	proteolysis	47.35	86.10
GDI2	oxidation-reduction process	81.79	49.51
GNAI2	DNA methylation, GTP metabolic process, energy reserve metabolic process, genetic imprinting	55.96	60.27
GNAS	DNA methylation, energy reserve metabolic process, genetic imprinting	17.22	25.83
ITGB1	receptor internalization	55.96	53.81
MAPK1	DNA damage induced protein phosphorylation, ERK1 and ERK2 cascade, MAPK cascade in response to starvation, MAPK cascade involved in osmosensory signaling pathway, MAPK import into nucleus, arachidonic acid metabolic process, cytosine metabolic process, glycosaminoglycan biosynthetic	34.44	53.81
MAPK3	DNA damage induced protein phosphorylation, ERK1 and ERK2 cascade, MAPK import into nucleus, arachidonic acid metabolic process, peptidyl-serine phosphorylation, peptidyl-tyrosine autophosphorylation, phosphorylation, transcription initiation from RNA polymerase I promoter, transcription, DNA-t	10.76	75.34
PDCD6IP	ubiquitin-independent protein catabolic process via the multivesicular body sorting pathway	45.20	64.57
WARS	tRNA aminoacylation for protein translation, translation, tryptophanyl-tRNA aminoacylation	73.18	68.88
MDH1	NAD metabolic process, NADH metabolic process, carbohydrate metabolic process, carboxylic acid metabolic process, gluconeogenesis, malate metabolic process, oxaloacetate metabolic process, oxidation-reduction process, tricarboxylic acid cycle	32.29	62.42
PPP1CA	carbohydrate metabolic process, dephosphorylation, glycogen metabolic process, regulation of translational initiation by eIF2 alpha dephosphorylation	27.98	77.49
PPP1CB	glycogen metabolic process, protein dephosphorylation	25.83	36.59
RAB10	autophagosome assembly, autophagy, peptidyl-cysteine methylation	64.57	73.18
RAB1B	autophagy	27.98	38.74
RAB1A	autophagosome assembly, autophagy	32.29	10.76
SPTAN1	MAPK cascade	51.66	51.66
GCN1	translation	55.96	47.35
P3H1	collagen metabolic process, oxidation-reduction process, peptidyl-proline hydroxylation, protein hydroxylation	47.35	83.95
COL6A2	collagen catabolic process	36.59	66.73
MIF	carboxylic acid metabolic process, prostaglandin biosynthetic process	30.13	66.73
PLOD1	cellular protein modification process, hydroxylysine biosynthetic process, oxidation-reduction process	43.05	40.90
ATP6V1A	ATP metabolic process	66.73	58.12
VDAC1	macroautophagy	90.40	43.05
HDLBP	cholesterol metabolic process	68.88	36.59
FKBP10	protein peptidyl-prolyl isomerization	66.73	60.27
HNRNPK	gene expression, mRNA processing, mRNA splicing, via spliceosome, protein sumoylation, transcription from RNA polymerase II promoter, transcription, DNA-templated	38.74	66.73
PYGB	5-phosphoribose 1-diphosphate biosynthetic process, glycogen catabolic process, metabolic process	38.74	60.27
PYGL	5-phosphoribose 1-diphosphate biosynthetic process, glycogen catabolic process	8.61	58.12
SARS	selenocysteine metabolic process, selenocysteinyl-tRNA(Sec) biosynthetic process, seryl-tRNA aminoacylation, tRNA processing, translation	43.05	51.66
PHGDH	L-serine biosynthetic process, cellular amino acid biosynthetic process, gamma-aminobutyric acid metabolic process, glutamine metabolic process, glycine metabolic process, oxidation-reduction process, serine family amino acid biosynthetic process, taurine metabolic process, threonine metabolic process	77.49	71.03
XRCC6	DNA ligation, DNA recombination, double-strand break repair via classical nonhomologous end joining, telomere maintenance, transcription, DNA-templated	19.37	81.79
GSTO1	L-ascorbic acid metabolic process, glutathione derivative biosynthetic process, glutathione metabolic process, methylation, oxidation-reduction process, xenobiotic catabolic process	30.13	34.44
LMO7	protein ubiquitination	86.10	71.03
NPM1	DNA repair	27.98	60.27
DDOST	protein N-linked glycosylation, protein N-linked glycosylation via asparagine	36.59	38.74
CAND1	protein ubiquitination	38.74	47.35
ESD	formaldehyde catabolic process, glutathione derivative biosynthetic process	34.44	81.79
PEBP1	MAPK cascade	38.74	55.96
RPL9	cytoplasmic translation, nuclear-transcribed mRNA catabolic process, nonsense-mediated decay, rRNA processing, translation, translational initiation, viral transcription	49.51	17.22
RPS3	DNA repair, nuclear-transcribed mRNA catabolic process, nonsense-mediated decay, rRNA processing, transcription, DNA-templated, translation, translational initiation, viral transcription	51.66	55.96
PSAT1	L-serine biosynthetic process, cellular amino acid biosynthetic process, pyridoxine biosynthetic process	64.57	34.44
RPL12	nuclear-transcribed mRNA catabolic process, nonsense-mediated decay, rRNA processing, translation, translational initiation, viral transcription	25.83	10.76
RPS7	nuclear-transcribed mRNA catabolic process, nonsense-mediated decay, rRNA processing, translation, translational initiation, viral transcription	27.98	45.20
CAPN1	proteolysis, receptor catabolic process	34.44	73.18
PRDX4	4-hydroxyproline metabolic process, I-kappaB phosphorylation, oxidation-reduction process, protein maturation by protein folding, reactive oxygen species metabolic process	60.27	27.98
VARS	tRNA aminoacylation for protein translation, translation, valyl-tRNA aminoacylation	34.44	25.83
EEF1B2	translation, translational elongation	43.05	64.57
RPL7A	maturation of LSU-rRNA, nuclear-transcribed mRNA catabolic process, nonsense-mediated decay, rRNA processing, translation, translational initiation, viral transcription	30.13	12.91
CTSK	collagen catabolic process, proteolysis involved in cellular protein catabolic process	12.91	27.98
NME1	CTP biosynthetic process, DNA metabolic process, GTP biosynthetic process, UTP biosynthetic process, nucleobase-containing small molecule interconversion, nucleoside diphosphate phosphorylation, phosphorylation, pyrimidine nucleotide metabolic process	36.59	32.29
PRDX5	NADPH oxidation, hydrogen peroxide catabolic process, oxidation-reduction process, reactive nitrogen species metabolic process	49.51	51.66
SPTBN1	MAPK cascade, common-partner SMAD protein phosphorylation	38.74	47.35
ALDH1L2	10-formyltetrahydrofolate catabolic process, biosynthetic process, folic acid metabolic process, metabolic process, one-carbon metabolic process, oxidation-reduction process	45.20	40.90
RNPEP	peptide catabolic process, proteolysis	21.52	45.20
ATP1A1	dephosphorylation	17.22	77.49
NAP1L1	DNA replication	32.29	55.96
PTBP1	IRES-dependent viral translational initiation, alternative mRNA splicing, via spliceosome, gene expression, mRNA processing, mRNA splicing, via spliceosome	49.51	47.35
ATP6V1B2	ATP metabolic process	49.51	30.13
PCBP1	gene expression, mRNA splicing, via spliceosome, viral RNA genome replication	49.51	60.27
PLOD2	cellular protein modification process, oxidation-reduction process	45.20	62.42
RPS3A	nuclear-transcribed mRNA catabolic process, nonsense-mediated decay, rRNA processing, translation, translational initiation, viral transcription	43.05	66.73
TXNRD1	nucleobase-containing small molecule interconversion, oxidation-reduction process, selenium compound metabolic process	66.73	90.40
ADH5	ethanol oxidation, formaldehyde catabolic process, oxidation-reduction process, peptidyl-cysteine S-nitrosylation, retinoid metabolic process	32.29	66.73
COMT	catecholamine metabolic process, dopamine catabolic process, estrogen metabolic process, methylation, neurotransmitter catabolic process	34.44	27.98
NME2	CTP biosynthetic process, GTP biosynthetic process, UTP biosynthetic process, nucleobase-containing small molecule interconversion, nucleoside diphosphate phosphorylation, nucleoside triphosphate biosynthetic process, nucleotide metabolic process, peptidyl-histidine pho	36.59	38.74
PLOD3	oxidation-reduction process	47.35	38.74
PSMA4	MAPK cascade, anaphase-promoting complex-dependent catabolic process, proteasome-mediated ubiquitin-dependent protein catabolic process, protein polyubiquitination, proteolysis involved in cellular protein catabolic process	38.74	8.61
C1QBP	RNA splicing, blood coagulation, intrinsic pathway, complement activation, classical pathway, mRNA processing, transcription, DNA-templated	25.83	51.66
GFPT1	UDP-N-acetylglucosamine biosynthetic process, carbohydrate derivative biosynthetic process, carbohydrate metabolic process, energy reserve metabolic process, fructose 6-phosphate metabolic process, glutamine metabolic process	47.35	45.20
GBE1	generation of precursor metabolites and energy, glycogen biosynthetic process	23.68	68.88
EIF4G1	nuclear-transcribed mRNA catabolic process, nonsense-mediated decay, nuclear-transcribed mRNA poly(A) tail shortening, translation, translational initiation	17.22	75.34
RAB5C	receptor internalization involved in canonical Wnt signaling pathway, viral RNA genome replication	43.05	77.49
PGM3	UDP-N-acetylglucosamine biosynthetic process, carbohydrate metabolic process, glucosamine metabolic process, glucose 1-phosphate metabolic process, protein N-linked glycosylation, protein O-linked glycosylation	36.59	32.29
CNDP2	glutathione biosynthetic process, peptide catabolic process, proteolysis, sulfur amino acid metabolic process	21.52	49.51
DDX3X	RNA secondary structure unwinding, transcription, DNA-templated, translational initiation	23.68	19.37
ECHS1	fatty acid beta-oxidation, fatty acid metabolic process, lipid metabolic process	21.52	62.42
FDPS	cholesterol biosynthetic process, farnesyl diphosphate biosynthetic process, geranyl diphosphate biosynthetic process, isoprenoid biosynthetic process, lipid metabolic process, steroid biosynthetic process, sterol biosynthetic process	8.61	30.13
CYB5R3	L-ascorbic acid metabolic process, cholesterol biosynthetic process, lipid metabolic process, oxidation-reduction process, steroid biosynthetic process, sterol biosynthetic process	40.90	86.10
PAPSS2	3'-phosphoadenosine 5'-phosphosulfate biosynthetic process, phosphorylation, sulfate assimilation	32.29	27.98
RAN	DNA metabolic process	25.83	43.05
RPL4	nuclear-transcribed mRNA catabolic process, nonsense-mediated decay, rRNA processing, translation, translational initiation, viral transcription	38.74	38.74
MTHFD1	folic acid metabolic process, folic acid-containing compound biosynthetic process, folic acid-containing compound metabolic process, formate metabolic process, one-carbon metabolic process, oxidation-reduction process, tetrahydrofolate interconversion, tetrahydrofolate metabolic proce	19.37	38.74
HADHB	cardiolipin acyl-chain remodeling, fatty acid beta-oxidation, fatty acid metabolic process, lipid metabolic process	27.98	49.51
EEF1D	mRNA transcription, transcription, DNA-templated, translation, translational elongation	34.44	51.66
FH	fumarate metabolic process, malate metabolic process, tricarboxylic acid cycle	32.29	25.83
PPA1	diphosphate metabolic process, phosphate-containing compound metabolic process, tRNA aminoacylation for protein translation	27.98	27.98
RPL5	nuclear-transcribed mRNA catabolic process, nonsense-mediated decay, rRNA processing, translation, translational initiation, viral transcription	30.13	34.44
RPS2	nuclear-transcribed mRNA catabolic process, nonsense-mediated decay, rRNA processing, translation, translational initiation, viral transcription	23.68	36.59
COL3A1	collagen catabolic process, peptide cross-linking	21.52	60.27
PRKCSH	N-glycan processing	30.13	34.44
MRC2	collagen catabolic process	40.90	25.83
PSME2	MAPK cascade, anaphase-promoting complex-dependent catabolic process, protein polyubiquitination	12.91	34.44

[a]Exponentially Modified Protein Abundance Index (http://www.matrixscience.com/help/quant_empai_help.html).

Table S7. Cont 2.

UniProt/SWISS-<br>Alternate ID	Biological Process<br>biological regulation	emPAI[a]<br>CDM	 DMEM
ATIC	'de novo' IMP biosynthetic process, cobalamin metabolic process, dihydrofolate metabolic process, nucleoside metabolic process, purine ribonucleoside monophosphate biosynthetic process, ribonucleotide metabolic process, tetrahydrofolate biosynthetic process	10.27	36.59
RPS5	nuclear-transcribed mRNA catabolic process, nonsense-mediated decay, rRNA processing, translation, translational initiation, viral transcription	16.81	23.68
ACAT2	fatty acid beta-oxidation, lipid metabolic process, metabolic process	17.74	21.52
YBX1	mRNA processing, mRNA splicing, via spliceosome, transcription from RNA polymerase II promoter	17.74	25.83
AKR1B1	C21-steroid hormone biosynthetic process, carbohydrate metabolic process, daunorubicin metabolic process, doxorubicin metabolic process, fructose biosynthetic process, monosaccharide metabolic process, naphthalene metabolic process, norepinephrine metabolic process, oxidation-reducti	16.81	25.83
GLS	cellular amino acid biosynthetic process, glutamate biosynthetic process, glutamine catabolic process	6.54	36.59
PCBP2	IRES-dependent viral translational initiation, gene expression, mRNA metabolic process, mRNA splicing, via spliceosome, proteasome-mediated ubiquitin-dependent protein catabolic process, viral RNA genome replication	25.21	34.44
RPS8	maturation of SSU-rRNA from tricistronic rRNA transcript (SSU-rRNA, 5.8S rRNA, LSU-rRNA), nuclear-transcribed mRNA catabolic process, nonsense-mediated decay, translation, translational initiation, viral transcription	7.47	34.44
SCRN1	proteolysis	14.94	27.98
SSB	IRES-dependent viral translational initiation, RNA processing, histone mRNA metabolic process, tRNA modification	18.68	12.91
PSMC4	MAPK cascade, anaphase-promoting complex-dependent catabolic process, proteasome-mediated ubiquitin-dependent protein catabolic process, protein polyubiquitination, proteolysis, ubiquitin-dependent ERAD pathway	13.07	25.83
RPS15	nuclear-transcribed mRNA catabolic process, nonsense-mediated decay, rRNA processing, translation, translational initiation, viral transcription	18.68	19.37
RPS4X	nuclear-transcribed mRNA catabolic process, nonsense-mediated decay, rRNA processing, translation, translational initiation, viral transcription	13.07	27.98
UAP1	UDP-N-acetylglucosamine biosynthetic process	14.01	17.22
ITGAV	ERK1 and ERK2 cascade	3.74	43.05
EIF3E	formation of translation preinitiation complex, nuclear-transcribed mRNA catabolic process, nonsense-mediated decay, translation	21.48	10.76
BZW1	transcription, DNA-templated	16.81	19.37
EIF2S1	protein autophosphorylation, translation, translational initiation, xenophagy	10.27	34.44
EIF4H	cytoplasmic translation, formation of translation preinitiation complex, translation	14.94	21.52
PABPC4	RNA catabolic process, mRNA polyadenylation, mRNA processing, mRNA splicing, via spliceosome, nuclear-transcribed mRNA catabolic process, nonsense-mediated decay, nuclear-transcribed mRNA poly(A) tail shortening, translation, translational initiation	17.74	25.83
PABPC1	mRNA polyadenylation, mRNA processing, mRNA splicing, via spliceosome, nuclear-transcribed mRNA catabolic process, nonsense-mediated decay, nuclear-transcribed mRNA poly(A) tail shortening, translational initiation	17.74	23.68
PSMA7	MAPK cascade, anaphase-promoting complex-dependent catabolic process, proteasome-mediated ubiquitin-dependent protein catabolic process, protein polyubiquitination, proteolysis, proteolysis involved in cellular protein catabolic process, ubiquitin-dependent protein catabolic proces	14.94	19.37
RPL3	nuclear-transcribed mRNA catabolic process, nonsense-mediated decay, rRNA processing, translation, translational initiation, viral transcription	14.94	27.98
YARS	tRNA aminoacylation for protein translation, translation, tyrosyl-tRNA aminoacylation, valyl-tRNA aminoacylation	10.27	32.29
HSD17B4	alpha-linolenic acid metabolic process, androgen metabolic process, bile acid biosynthetic process, estrogen metabolic process, fatty acid beta-oxidation, fatty acid beta-oxidation using acyl-CoA oxidase, medium-chain fatty-acyl-CoA metabolic process, oxidation-reduction process, very long-chain fatty acid metal	9.34	32.29
ERO1A	4-hydroxyproline metabolic process, cellular protein modification process, oxidation-reduction process, protein maturation by protein folding	17.74	12.91
PSMD3	MAPK cascade, anaphase-promoting complex-dependent catabolic process, proteasome-mediated ubiquitin-dependent protein catabolic process, protein polyubiquitination, ubiquitin-dependent protein catabolic process	8.40	36.59
LAP3	protein metabolic process, proteolysis	11.21	27.98
MTPN	catecholamine metabolic process	18.68	10.76
ALDH18A1	L-proline biosynthetic process, cellular amino acid biosynthetic process, citrulline biosynthetic process, glutamate metabolic process, ornithine biosynthetic process, oxidation-reduction process, phosphorylation	13.07	21.52
RAD23B	global genome nucleotide-excision repair, nucleotide-excision repair, DNA damage recognition, nucleotide-excision repair, DNA duplex unwinding, nucleotide-excision repair, preincision complex assembly, proteasome-mediated ubiquitin-dependent protein catabolic process	12.14	23.68
NANS	carbohydrate biosynthetic process, lipopolysaccharide biosynthetic process	10.27	27.98
UBE2L3	cellular protein modification process, protein K11-linked ubiquitination, protein polyubiquitination, protein ubiquitination, transcription, DNA-templated, ubiquitin-dependent protein catabolic process	15.88	17.22
EIF3I	formation of translation preinitiation complex, translation	6.54	30.13
ELOC	protein ubiquitination involved in ubiquitin-dependent protein catabolic process, transcription elongation from RNA polymerase II promoter, transcription from RNA polymerase II promoter, transcription, DNA-templated, ubiquitin-dependent protein catabolic process	9.34	25.83
LTA4H	leukotriene biosynthetic process, leukotriene metabolic process, peptide catabolic process, proteolysis	8.40	27.98
PSMA6	MAPK cascade, anaphase-promoting complex-dependent catabolic process, protein polyubiquitination, proteolysis involved in cellular protein catabolic process	11.21	23.68
RAB7A	epidermal growth factor catabolic process, lipid catabolic process, lipid metabolic process, lipophagy	7.47	32.29
RPS10	nuclear-transcribed mRNA catabolic process, nonsense-mediated decay, rRNA processing, translation, translational initiation, viral transcription	14.94	10.76
STRAP	RNA splicing, mRNA processing, spliceosomal snRNP assembly	8.40	30.13
CBR1	cyclooxygenase pathway, drug metabolic process, oxidation-reduction process, phylloquinone catabolic process, vitamin K metabolic process	4.67	38.74
CBR3	oxidation-reduction process, phylloquinone catabolic process	3.74	12.91
SLC3A2	carbohydrate metabolic process	1.87	43.05
ETF1	nuclear-transcribed mRNA catabolic process, nonsense-mediated decay, protein methylation, translational termination	12.14	15.07
FKBP9	protein peptidyl-prolyl isomerization	5.60	36.59
GDI1	oxidation-reduction process	28.95	55.96
EIF2S3	formation of translation preinitiation complex, translation	4.67	38.74
PRKACA	lipoprotein metabolic process, mRNA processing, peptidyl-serine phosphorylation, peptidyl-threonine phosphorylation, phosphorylation, protein autophosphorylation	8.40	19.37
PRKACB	lipoprotein metabolic process, phosphorylation, protein phosphorylation	8.40	15.07
PSMB2	MAPK cascade, anaphase-promoting complex-dependent catabolic process, proteasome-mediated ubiquitin-dependent protein catabolic process, protein polyubiquitination, proteolysis, proteolysis involved in cellular protein catabolic process	13.07	15.07
HNRNPA1	RNA splicing, gene expression, mRNA processing, mRNA splicing, via spliceosome	8.40	21.52
RARS	arginyl-tRNA aminoacylation, tRNA aminoacylation for protein translation, translation	6.54	36.59
TARS	tRNA aminoacylation for protein translation, threonyl-tRNA aminoacylation, translation	6.54	27.98
UBE2V1	DNA double-strand break processing, double-strand break repair via nonhomologous end joining, error-free postreplication DNA repair, global genome nucleotide-excision repair, postreplication repair, protein K63-linked ubiquitination	9.34	23.68
UBE2V2	DNA double-strand break processing, double-strand break repair via nonhomologous end joining, error-free postreplication DNA repair, global genome nucleotide-excision repair, protein K63-linked ubiquitination	6.54	19.37
HINT1	purine ribonucleotide catabolic process, transcription, DNA-templated	7.47	19.37
ALDH9A1	carnitine biosynthetic process, cellular aldehyde metabolic process, hormone metabolic process, neurotransmitter biosynthetic process, oxidation-reduction process	15.88	10.76
GLUD1	cellular amino acid biosynthetic process, glutamate biosynthetic process, glutamate catabolic process, glutamine metabolic process, oxidation-reduction process, tricarboxylic acid metabolic process	15.88	4.31
DHX9	RNA processing, mRNA splicing, via spliceosome	8.40	27.98
EIF3K	formation of translation preinitiation complex, translation	13.07	19.37
FKBP1A	amyloid fibril formation, protein maturation by protein folding, protein peptidyl-prolyl isomerization	10.27	21.52
RPL6	cytoplasmic translation, nuclear-transcribed mRNA catabolic process, nonsense-mediated decay, rRNA processing, translation, translational initiation, viral transcription	9.34	21.52
RPL7	cytoplasmic translation, maturation of LSU-rRNA from tricistronic rRNA transcript (SSU-rRNA, 5.8S rRNA, LSU-rRNA), nuclear-transcribed mRNA catabolic process, nonsense-mediated decay, translation, translational initiation, viral transcription	7.47	17.22
HARS	histidyl-tRNA aminoacylation, mitochondrial translation	11.21	21.52
LARS	leucyl-tRNA aminoacylation, translation	8.40	25.83
NARS	asparaginyl-tRNA aminoacylation, tRNA aminoacylation for protein translation, translation	6.54	27.98
USP14	protein deubiquitination, ubiquitin-dependent protein catabolic process	13.07	12.91
CARS	cysteinyl-tRNA aminoacylation, translation	3.74	34.44
RPL29	cytoplasmic translation, nuclear-transcribed mRNA catabolic process, nonsense-mediated decay, rRNA processing, translation, translational initiation, viral transcription	7.47	27.98
PRMT5	DNA-templated transcription, termination, covalent chromatin modification, histone H4-R3 methylation, methylation, peptidyl-arginine methylation, peptidyl-arginine methylation, to symmetrical-dimethyl arginine, spliceosomal snRNP assembly, transcription, DNA-templated	12.14	12.91
DDX1	RNA secondary structure unwinding, double-strand break repair, mRNA processing, nucleic acid phosphodiester bond hydrolysis, spliceosomal complex assembly, tRNA processing, tRNA splicing, via endonucleolytic cleavage and ligation, transcription, DNA-templated	5.60	32.29
GSPT1	cytoplasmic translational termination, nuclear-transcribed mRNA catabolic process, nonsense-mediated decay, protein methylation, translation	3.74	17.22
SHMT2	L-serine biosynthetic process, folic acid metabolic process, glycine biosynthetic process from serine, one-carbon metabolic process, protein K63-linked deubiquitination, tetrahydrofolate interconversion, tetrahydrofolate metabolic process	10.27	21.52
LAMP1	autophagy	10.27	17.22
PAK2	MAPK cascade, histone H2B-S14 phosphorylation, peptidyl-serine phosphorylation, phosphorylation, protein autophosphorylation	12.14	10.76
PFKL	canonical glycolysis, carbohydrate metabolic process, fructose 1,6-bisphosphate metabolic process, fructose 6-phosphate metabolic process, glycolytic process, glycolytic process through fructose-6-phosphate, metabolic process, phosphorylation	13.07	23.68
PSMC3	MAPK cascade, anaphase-promoting complex-dependent catabolic process, protein polyubiquitination, ubiquitin-dependent ERAD pathway	3.74	36.59
NPEPPS	peptide catabolic process, protein polyubiquitination, proteolysis	13.07	12.91
SRM	polyamine biosynthetic process, spermidine biosynthetic process	7.47	27.98
QARS	glutaminyl-tRNA aminoacylation, translation	11.21	10.76
SDHA	anaerobic respiration, cellular respiration, mitochondrial electron transport, succinate to ubiquinone, oxidation-reduction process, respiratory electron transport chain, succinate metabolic process, tricarboxylic acid cycle	5.60	19.37
FABP5	glucose metabolic process, phosphatidylcholine biosynthetic process, triglyceride catabolic process	13.07	12.91
PSMA5	MAPK cascade, RNA phosphodiester bond hydrolysis, anaphase-promoting complex-dependent catabolic process, proteasome-mediated ubiquitin-dependent protein catabolic process, protein polyubiquitination, proteolysis involved in cellular protein catabolic process	0.93	38.74
GOT1	2-oxoglutarate metabolic process, aspartate biosynthetic process, aspartate catabolic process, biosynthetic process, cellular amino acid biosynthetic process, dicarboxylic acid metabolic process, gluconeogenesis, glutamate catabolic process to 2-oxoglutarate, glutamate catabolic process to aspartate, glutamat	4.67	32.29
CS	carbohydrate metabolic process, tricarboxylic acid cycle	17.74	4.31
COL5A1	collagen biosynthetic process, collagen catabolic process, integrin biosynthetic process	3.74	34.44
HEXB	carbohydrate metabolic process, cellular protein metabolic process, chondroitin sulfate catabolic process, ganglioside catabolic process, glycosaminoglycan metabolic process, glycosphingolipid metabolic process, hyaluronan catabolic process, keratan sulfate catabolic process, oligosaccharide catabolic proces	9.34	17.22
HNRNPM	alternative mRNA splicing, via spliceosome, gene expression, mRNA processing, mRNA splicing, via spliceosome	9.34	21.52
EIF5B	translation, translational initiation	11.21	10.76
PAFAH1B2	lipid catabolic process	14.01	12.91
PSMD14	MAPK cascade, anaphase-promoting complex-dependent catabolic process, double-strand break repair via homologous recombination, double-strand break repair via nonhomologous end joining, proteasome-mediated ubiquitin-dependent protein catabolic process, protein K63-linked deubiq	12.14	10.76
RPL8	cytoplasmic translation, nuclear-transcribed mRNA catabolic process, nonsense-mediated decay, rRNA processing, translation, translational initiation, viral transcription	10.27	15.07
AHCY	S-adenosylhomocysteine catabolic process, S-adenosylmethionine cycle, homocysteine biosynthetic process, methylation, one-carbon metabolic process	12.14	19.37
SET	DNA replication	11.21	12.91
PSMB5	MAPK cascade, anaphase-promoting complex-dependent catabolic process, protein polyubiquitination, proteolysis, proteolysis involved in cellular protein catabolic process	6.54	27.98
IDH2	2-oxoglutarate metabolic process, carbohydrate metabolic process, glyoxylate cycle, isocitrate metabolic process, oxidation-reduction process, tricarboxylic acid cycle	8.40	21.52
PICALM	positive regulation of aspartic-type endopeptidase activity involved in amyloid precursor protein catabolic process, receptor internalization	5.60	23.68
ALDH7A1	cellular aldehyde metabolic process, choline catabolic process, glycine betaine biosynthetic process from choline, lysine catabolic process, metabolic process, oxidation-reduction process	5.60	30.13
ASNS	L-asparagine biosynthetic process, asparagine biosynthetic process, cellular amino acid biosynthetic process, glutamine metabolic process	0.93	36.59
OAT	L-proline biosynthetic process, arginine catabolic process to glutamate, arginine catabolic process to proline via ornithine, cellular amino acid biosynthetic process	4.67	30.13

[a]Exponentially Modified Protein Abundance Index (http://www.matrixscience.com/help/quant_empai_help.html).

Table S7. Cont 3.

UniProt/SWISS-Alternate ID	Biological Process<br>biological regulation	emPAI[a]<br>CDM	 DMEM
PGM1	galactose catabolic process, gluconeogenesis, glycogen biosynthetic process, glycogen catabolic process, glycolytic process, organic substance metabolic process	6.54	27.98
PHB2	transcription, DNA-templated	9.34	19.37
PSMA2	MAPK cascade, anaphase-promoting complex-dependent catabolic process, proteasome-mediated ubiquitin-dependent protein catabolic process, protein polyubiquitination, proteolysis involved in cellular protein catabolic process	14.94	8.61
PSMB4	MAPK cascade, anaphase-promoting complex-dependent catabolic process, proteasome-mediated ubiquitin-dependent protein catabolic process, protein polyubiquitination, proteolysis, proteolysis involved in cellular protein catabolic process	7.47	17.22
PSMD13	MAPK cascade, anaphase-promoting complex-dependent catabolic process, proteasome-mediated ubiquitin-dependent protein catabolic process, protein polyubiquitination, ubiquitin-dependent protein catabolic process	13.07	12.91
PAICS	'de novo' IMP biosynthetic process, adenine biosynthetic process, purine ribonucleoside monophosphate biosynthetic process	8.40	21.52
CAD	'de novo' UMP biosynthetic process, 'de novo' pyrimidine nucleobase biosynthetic process, UTP biosynthetic process, arginine biosynthetic process, citrulline biosynthetic process, drug metabolic process, glutamine metabolic process, nitrogen compound metabolic	8.40	15.07
RPL11	nuclear-transcribed mRNA catabolic process, nonsense-mediated decay, rRNA processing, translation, translational initiation, viral transcription	8.40	19.37
UBR4	protein ubiquitination, protein ubiquitination involved in ubiquitin-dependent protein catabolic process	7.47	17.22
PSMC2	MAPK cascade, anaphase-promoting complex-dependent catabolic process, protein polyubiquitination, ubiquitin-dependent ERAD pathway	7.47	23.68
APRT	AMP salvage, adenine metabolic process, adenine salvage, nucleoside metabolic process	10.27	10.76
CDC42	protein ubiquitination, viral RNA genome replication	10.27	17.22
TUFM	mitochondrial translational elongation, translation, translational elongation	10.27	12.91
MMP14	collagen catabolic process, protein processing, proteolysis, zymogen activation	0.93	34.44
OLA1	ATP metabolic process	10.27	12.91
PPP2CA	RNA splicing, ceramide metabolic process, nuclear-transcribed mRNA catabolic process, nonsense-mediated decay, protein dephosphorylation	4.67	12.91
RPL15	cytoplasmic translation, nuclear-transcribed mRNA catabolic process, nonsense-mediated decay, rRNA processing, translation, translational initiation, viral transcription	8.40	17.22
EHD1	receptor recycling	5.60	27.98
SH3BGRL3	oxidation-reduction process	10.27	6.46
DPP4	proteolysis	0.93	36.59
CDC37	peptidyl-tyrosine phosphorylation	4.67	21.52
CPNE1	glycerophospholipid biosynthetic process, lipid metabolic process, proteolysis, transcription, DNA-templated	12.14	10.76
DPP3	proteolysis	14.01	6.46
KHSRP	RNA splicing, via transesterification reactions, mRNA catabolic process, mRNA processing, transcription, DNA-templated	6.54	10.76
GRHPR	dicarboxylic acid metabolic process, glyoxylate metabolic process, oxidation-reduction process	7.47	17.22
HNRNPF	gene expression, mRNA processing, mRNA splicing, via spliceosome	5.60	32.29
PAFAH1B1	lipid catabolic process, platelet activating factor metabolic process	9.34	12.91
PHB	DNA biosynthetic process, histone deacetylation	9.34	12.91
PNP	NAD biosynthesis via nicotinamide riboside salvage pathway, inosine catabolic process, nicotinamide riboside catabolic process, purine nucleoside metabolic process, purine nucleotide catabolic process, purine-containing compound salvage, urate biosynthetic process	4.67	27.98
PSMC5	MAPK cascade, anaphase-promoting complex-dependent catabolic process, protein polyubiquitination, transcription from RNA polymerase II promoter, ubiquitin-dependent ERAD pathway	7.47	17.22
PSMB3	MAPK cascade, anaphase-promoting complex-dependent catabolic process, proteasome-mediated ubiquitin-dependent protein catabolic process, protein polyubiquitination, proteolysis involved in cellular protein catabolic process	8.40	17.22
PTGR1	leukotriene metabolic process, oxidation-reduction process, prostaglandin metabolic process	10.27	10.76
RPS17	nuclear-transcribed mRNA catabolic process, nonsense-mediated decay, rRNA processing, translation, translational initiation, viral transcription	8.40	19.37
KARS	diadenosine tetraphosphate biosynthetic process, lysyl-tRNA aminoacylation, tRNA processing, translation	10.27	12.91
RPL17	nuclear-transcribed mRNA catabolic process, nonsense-mediated decay, rRNA processing, translation, translational initiation, viral transcription	7.47	15.07
EIF3M	cytoplasmic translational initiation, formation of translation preinitiation complex, translation	6.54	17.22
EIF3L	formation of translation preinitiation complex, translation, viral translational termination-reinitiation	6.54	15.07
CCDC47	ubiquitin-dependent ERAD pathway	5.60	19.37
UBE2N	DNA double-strand break processing, double-strand break repair via homologous recombination, double-strand break repair via nonhomologous end joining, global genome nucleotide-excision repair, histone ubiquitination, postreplication repair, protein K63-linked ubiquitination, proteol	9.34	15.07
ALDH1B1	carbohydrate metabolic process, ethanol catabolic process, ethanol oxidation, oxidation-reduction process	13.07	4.31
BLVRA	heme catabolic process, oxidation-reduction process	11.21	10.76
EIF3F	IRES-dependent viral translational initiation, formation of translation preinitiation complex, protein deubiquitination, translation	10.27	12.91
FHL2	transcription, DNA-templated	3.74	23.68
GBA	carbohydrate metabolic process, ceramide biosynthetic process, glucosylceramide catabolic process, glycosphingolipid metabolic process, sphingosine biosynthetic process	7.47	17.22
GLRX3	oxidation-reduction process	10.27	8.61
SYNCRIP	RNA splicing, mRNA processing, mRNA splicing, via spliceosome	3.74	17.22
ILK	peptidyl-serine phosphorylation, phosphorylation	3.74	23.68
IMPDH2	GMP biosynthetic process, GTP biosynthetic process, oxidation-reduction process, purine ribonucleoside monophosphate biosynthetic process	7.47	19.37
GLO1	carbohydrate metabolic process, glutathione metabolic process, methylglyoxal metabolic process, pyruvate metabolic process	9.34	8.61
MOGS	oligosaccharide metabolic process, protein N-linked glycosylation	13.07	6.46
QPRT	NAD biosynthetic process, NAD metabolic process, quinolinate catabolic process	11.21	2.15
PCNA	DNA replication, error-free translesion synthesis, error-prone translesion synthesis, leading strand elongation, mismatch repair, mitotic telomere maintenance via semi-conservative replication, nucleotide-excision repair, DNA gap filling, nucleotide-excision repair, DNA incision, 5'-to lesio	9.34	10.76
PREP	proteolysis	4.67	25.83
PSMB6	MAPK cascade, anaphase-promoting complex-dependent catabolic process, proteasome-mediated ubiquitin-dependent protein catabolic process, protein polyubiquitination, proteolysis involved in cellular protein catabolic process	8.40	15.07
PSMD7	MAPK cascade, anaphase-promoting complex-dependent catabolic process, protein polyubiquitination	4.67	19.37
RPL18	nuclear-transcribed mRNA catabolic process, nonsense-mediated decay, rRNA processing, translation, translational initiation, viral transcription	1.87	27.98
RPS6	mitochondrial translation, mitochondrial translational elongation, mitochondrial translational termination	6.54	21.52
SMS	methionine metabolic process, spermine biosynthetic process, spermine metabolic process	5.60	17.22
TSPO	heme biosynthetic process, steroid biosynthetic process, steroid metabolic process	9.34	8.61
RPL13	nuclear-transcribed mRNA catabolic process, nonsense-mediated decay, rRNA processing, translation, translational initiation, viral transcription	8.40	17.22
ECM29	ubiquitin-dependent ERAD pathway	6.54	12.91
FBLN1	blood coagulation, fibrin clot formation	4.67	23.68
PPP3CA	dephosphorylation, protein dephosphorylation	1.87	17.22
CRTAP	peptidyl-proline hydroxylation to 3-hydroxy-L-proline	9.34	12.91
CTNND1	transcription, DNA-templated	4.67	21.52
DTD1	D-amino acid catabolic process, DNA replication, tRNA metabolic process	6.54	12.91
GNS	glycosaminoglycan metabolic process, keratan sulfate catabolic process, metabolic process	7.47	8.61
PDXK	phosphorylation, pyridoxal 5'-phosphate salvage, vitamin B6 metabolic process	3.74	19.37
PEA15	MAPK cascade	6.54	19.37
PRDX2	hydrogen peroxide catabolic process, oxidation-reduction process, removal of superoxide radicals	11.21	19.37
PSMB1	MAPK cascade, anaphase-promoting complex-dependent catabolic process, proteasome-mediated ubiquitin-dependent protein catabolic process, protein polyubiquitination, proteolysis, proteolysis involved in cellular protein catabolic process	6.54	17.22
TXNDC17	oxidation-reduction process	5.60	21.52
USP5	protein K48-linked deubiquitination, proteolysis, ubiquitin-dependent protein catabolic process	11.21	8.61
RPS15A	nuclear-transcribed mRNA catabolic process, nonsense-mediated decay, rRNA processing, translation, translational initiation, viral transcription	5.60	19.37
MCTS1	IRES-dependent translational initiation, formation of translation preinitiation complex, transcription, DNA-templated, translation	9.34	12.91
PSMB7	MAPK cascade, anaphase-promoting complex-dependent catabolic process, proteasome-mediated ubiquitin-dependent protein catabolic process, protein polyubiquitination, proteolysis, proteolysis involved in cellular protein catabolic process	9.34	12.91
PSMC6	MAPK cascade, anaphase-promoting complex-dependent catabolic process, proteasome-mediated ubiquitin-dependent protein catabolic process, protein catabolic process, protein polyubiquitination, ubiquitin-dependent ERAD pathway	7.47	10.76
PPIC	protein peptidyl-prolyl isomerization	10.27	8.61
EIF6	maturation of 5.8S rRNA, maturation of LSU-rRNA, translation, translational initiation	7.47	10.76
EIF3A	IRES-dependent viral translational initiation, formation of cytoplasmic translation initiation complex, formation of translation preinitiation complex, translation reinitiation, viral translational termination-reinitiation	2.80	30.13
PPP1R12A	protein dephosphorylation	5.60	15.07
AIMP2	tRNA aminoacylation for protein translation, translation	8.40	12.91
CYCS	cellular respiration, mitochondrial electron transport, cytochrome c to oxygen, mitochondrial electron transport, ubiquinol to cytochrome c, oxidation-reduction process, protein dephosphorylation	12.14	4.31
HSD17B12	estrogen biosynthetic process, fatty acid biosynthetic process, lipid metabolic process, long-chain fatty-acyl-CoA biosynthetic process, oxidation-reduction process, steroid biosynthetic process	4.67	17.22
GSS	cellular amino acid metabolic process, glutathione biosynthetic process	4.67	19.37
HADH	fatty acid beta-oxidation, lipid metabolic process, oxidation-reduction process	5.60	15.07
LMF2	protein maturation	10.27	6.46
MFGE8	cellular protein metabolic process	4.67	17.22
NACA	transcription, DNA-templated	4.67	17.22
NAGK	N-acetylglucosamine metabolic process, N-acetylmannosamine metabolic process, N-acetylneuraminate catabolic process, UDP-N-acetylglucosamine biosynthetic process, carbohydrate phosphorylation, phosphorylation	1.87	21.52
OTUB1	DNA repair, protein K48-linked deubiquitination, protein deubiquitination	6.54	12.91
PEPD	cellular amino acid metabolic process, collagen catabolic process, proteolysis	7.47	15.07
CTSA	glycosphingolipid metabolic process, proteolysis involved in cellular protein catabolic process	7.47	15.07
DLD	2-oxoglutarate metabolic process, branched-chain amino acid catabolic process, dihydrolipoamide metabolic process, glyoxylate metabolic process, lipoate metabolic process, lysine catabolic process, mitochondrial acetyl-CoA biosynthetic process from pyruvate, mitochondrial electron transport, NADH to ubiqui	4.67	17.22
RPL22	cytoplasmic translation, nuclear-transcribed mRNA catabolic process, nonsense-mediated decay, rRNA processing, translation, translational initiation, viral transcription	8.40	12.91
STAT2	transcription, DNA-templated	7.47	6.46
ASPH	activation of cysteine-type endopeptidase activity, oxidation-reduction process, peptidyl-amino acid modification	10.27	2.15
ACSL3	fatty acid biosynthetic process, lipid metabolic process, long-chain fatty acid metabolic process, long-chain fatty-acyl-CoA biosynthetic process, metabolic process	11.21	6.46
APEX1	DNA demethylation, DNA recombination, RNA phosphodiester bond hydrolysis, endonucleolytic, base-excision repair, base-excision repair, base-free sugar-phosphate removal, nucleic acid phosphodiester bond hydrolysis, oxidation-reduction process, telomere maintenance, transcription, DNA-templa	12.14	2.15
DDB1	UV-damage excision repair, global genome nucleotide-excision repair, histone H2A monoubiquitination, nucleotide-excision repair, DNA damage recognition, nucleotide-excision repair, DNA duplex unwinding, nucleotide-excision repair, DNA incision, 3'-to lesio	4.67	17.22

[a]Exponentially Modified Protein Abundance Index (http://www.matrixscience.com/help/quant_empai_help.html).

Table S7. Cont 4.

UniProt/SWISS-<br>Alternate ID	Biological Process<br>biological regulation	emPAI[a]<br>CDM	 DMEM
DDT	melanin biosynthetic process	4.67	15.07
EIF3H	formation of translation preinitiation complex, translation, translational initiation	9.34	6.46
ENPP1	3'-phosphoadenosine 5'-phosphosulfate metabolic process, generation of precursor metabolites and energy, nucleic acid phosphodiester bond hydrolysis, nucleoside triphosphate catabolic process, riboflavin metabolic process	6.54	12.91
FLII	transcription, DNA-templated	7.47	8.61
GSTK1	glutathione derivative biosynthetic process, glutathione metabolic process, oxidation-reduction process	6.54	15.07
HSD17B10	branched-chain amino acid catabolic process, lipid metabolic process, mitochondrial tRNA methylation, mitochondrial tRNA processing, oxidation-reduction process, tRNA processing	7.47	10.76
HEXA	carbohydrate metabolic process, chondroitin sulfate catabolic process, ganglioside catabolic process, glycosaminoglycan biosynthetic process, glycosaminoglycan metabolic process, glycosphingolipid metabolic process, hyaluronan catabolic process, keratan sulfate catabolic process	6.54	12.91
LMAN1	protein N-linked glycosylation via asparagine	5.60	12.91
POR	carnitine metabolic process, cellular organofluorine metabolic process, demethylation, fatty acid oxidation, flavonoid metabolic process, internal peptidyl-lysine acetylation, nitrate catabolic process, nitric oxide catabolic process, oxidation-reduction process, xenobiotic metabolic process	9.34	6.46
PRDX3	hydrogen peroxide catabolic process, oxidation-reduction process, peptidyl-cysteine oxidation	7.47	12.91
PRPS1	5-phosphoribose 1-diphosphate biosynthetic process, AMP biosynthetic process, cellular biosynthetic process, hypoxanthine biosynthetic process, nucleoside metabolic process, nucleotide biosynthetic process, phosphorylation, purine nucleobase metabolic process, pyrimidine nucleotide biosynthetic process, ri	2.80	17.22
PSMD4	MAPK cascade, anaphase-promoting complex-dependent catabolic process, proteasome-mediated ubiquitin-dependent protein catabolic process, protein polyubiquitination, ubiquitin-dependent protein catabolic process	4.67	19.37
GART	'de novo' IMP biosynthetic process, biosynthetic process, glycine metabolic process, purine nucleobase biosynthetic process, purine ribonucleoside monophosphate biosynthetic process, ribonucleoside monophosphate biosynthetic process, tetrahydrofolate biosynt	5.60	10.76
SKP1	SCF-dependent proteasomal ubiquitin-dependent protein catabolic process, histone H2A monoubiquitination, proteasome-mediated ubiquitin-dependent protein catabolic process, protein polyubiquitination, protein ubiquitination, stress-activated MAPK cascade, ubi	3.74	21.52
DARS	aspartyl-tRNA aminoacylation, translation	2.80	23.68
UBE2K	proteasome-mediated ubiquitin-dependent protein catabolic process, protein K48-linked ubiquitination	7.47	12.91
CD81	receptor internalization	5.60	17.22
MGST1	glutathione derivative biosynthetic process, glutathione metabolic process, oxidation-reduction process, xenobiotic metabolic process	8.40	10.76
CTPS1	'de novo' CTP biosynthetic process, CTP biosynthetic process, glutamine metabolic process, nucleobase-containing small molecule interconversion, pyrimidine nucleotide biosynthetic process	7.47	12.91
PLBD2	lipid catabolic process, lipid metabolic process	7.47	8.61
RPL21	nuclear-transcribed mRNA catabolic process, nonsense-mediated decay, rRNA processing, translation, translational initiation, viral transcription	4.67	19.37
PCOLCE	proteolysis	9.34	4.31
APMAP	biosynthetic process	7.47	8.61
GPX1	glutathione metabolic process, hydrogen peroxide catabolic process, lipid metabolic process, lipoxygenase pathway, oxidation-reduction process, protein oxidation, purine nucleotide catabolic process, triglyceride metabolic process	3.74	12.91
RPL10	nuclear-transcribed mRNA catabolic process, nonsense-mediated decay, rRNA processing, translation, translational initiation, viral transcription	7.47	10.76
OGDH	2-oxoglutarate metabolic process, NADH metabolic process, generation of precursor metabolites and energy, glycolytic process, glyoxylate metabolic process, lysine catabolic process, oxidation-reduction process, succinyl-CoA metabolic process, tricarboxylic acid cycle	9.34	4.31
CTNNB1	proteasome-mediated ubiquitin-dependent protein catabolic process, transcription, DNA-templated	0.93	21.52
AKR1A1	D-glucuronate catabolic process, L-ascorbic acid biosynthetic process, aldehyde catabolic process, cellular aldehyde metabolic process, glucose metabolic process, glucuronate catabolic process to xylulose 5-phosphate, glutathione derivative biosynthetic process, oxidatio	3.74	21.52
BCAT1	aspartate biosynthetic process, branched-chain amino acid biosynthetic process, branched-chain amino acid catabolic process, leucine biosynthetic process, valine biosynthetic process	2.80	21.52
CARM1	cellular lipid metabolic process, covalent chromatin modification, histone H3-R17 methylation, histone H3-R2 methylation, histone methylation, methylation, peptidyl-arginine methylation, to asymmetrical-dimethyl arginine, transcription, DNA-templated	3.74	17.22
DDX6	RNA secondary structure unwinding, exonucleolytic nuclear-transcribed mRNA catabolic process involved in deadenylation-dependent decay	7.47	6.46
ERP44	glycoprotein metabolic process	6.54	12.91
FAH	L-phenylalanine catabolic process, arginine catabolic process, aromatic amino acid family metabolic process, metabolic process, tyrosine catabolic process	8.40	4.31
HNRNPH1	gene expression, mRNA processing, mRNA splicing, via spliceosome	7.47	25.83
AK2	ADP biosynthetic process, AMP metabolic process, ATP metabolic process, nucleobase-containing compound metabolic process, nucleobase-containing small molecule interconversion, nucleotide phosphorylation, phosphorylation	9.34	6.46
ME1	NADP biosynthetic process, carbohydrate metabolic process, malate metabolic process, oxidation-reduction process	3.74	17.22
PSMD11	MAPK cascade, anaphase-promoting complex-dependent catabolic process, proteasome-mediated ubiquitin-dependent protein catabolic process, protein polyubiquitination, ubiquitin-dependent protein catabolic process	3.74	19.37
PSMD12	MAPK cascade, anaphase-promoting complex-dependent catabolic process, protein polyubiquitination	3.74	12.91
PTK7	protein phosphorylation	0.93	21.52
RPL18A	nuclear-transcribed mRNA catabolic process, nonsense-mediated decay, rRNA processing, translation, translational initiation, viral transcription	1.87	17.22
TGFB1I1	transcription from RNA polymerase II promoter, ubiquitin-dependent SMAD protein catabolic process	1.87	23.68
TRIM28	DNA methylation involved in embryo development, DNA repair, covalent chromatin modification, protein autophosphorylation, protein sumoylation, protein ubiquitination, transcription initiation from RNA polymerase II promoter, transcription, DNA-templated	6.54	10.76
DYNLL1	macroautophagy, transcription, DNA-templated	5.60	12.91
BAG2	protein metabolic process	8.40	6.46
DPY30	covalent chromatin modification, histone H3-K4 methylation, transcription, DNA-templated	7.47	10.76
PCK2	NADH oxidation, gluconeogenesis, oxaloacetate metabolic process, pyruvate metabolic process	1.87	23.68
RPL19	nuclear-transcribed mRNA catabolic process, nonsense-mediated decay, rRNA processing, translation, translational initiation, viral transcription	4.67	15.07
UROD	heme biosynthetic process, heme metabolic process, protoporphyrinogen IX biosynthetic process	7.47	6.46
ERH	nucleobase-containing compound metabolic process, pyrimidine nucleoside metabolic process, pyrimidine nucleotide biosynthetic process	10.27	2.15
H6PD	carbohydrate metabolic process, glucose metabolic process, oxidation-reduction process, pentose-phosphate shunt	9.34	2.15
ADSL	'de novo' AMP biosynthetic process, 'de novo' IMP biosynthetic process, aerobic respiration, purine nucleotide metabolic process, purine ribonucleoside monophosphate biosynthetic process, ribonucleoside monophosphate biosynthetic process	2.80	17.22
RPL14	nuclear-transcribed mRNA catabolic process, nonsense-mediated decay, rRNA processing, translation, translational initiation, viral transcription	2.80	15.07
RPS12	nuclear-transcribed mRNA catabolic process, nonsense-mediated decay, rRNA processing, translation, translational initiation, viral transcription	0.93	23.68
RPS20	nuclear-transcribed mRNA catabolic process, nonsense-mediated decay, rRNA processing, translation, translational initiation, viral transcription	0.93	23.68
TALDO1	carbohydrate metabolic process, fructose 6-phosphate metabolic process, glyceraldehyde-3-phosphate metabolic process, pentose-phosphate shunt, pentose-phosphate shunt, non-oxidative branch, xylulose biosynthetic process	6.54	10.76
PTGES3	RNA-dependent DNA biosynthetic process, cyclooxygenase pathway, fatty acid biosynthetic process, fatty acid metabolic process, lipid metabolic process, prostaglandin biosynthetic process, prostaglandin metabolic process, telomere maintenance	3.74	17.22
ATP5O	ATP biosynthetic process, ATP synthesis coupled proton transport, mitochondrial ATP synthesis coupled proton transport	5.60	12.91
DHRS7	oxidation-reduction process	3.74	15.07
STOML2	mitochondrial ATP synthesis coupled proton transport, mitochondrial protein processing	3.74	12.91
PSMD6	MAPK cascade, anaphase-promoting complex-dependent catabolic process, protein polyubiquitination, proteolysis	7.47	6.46
ATP5L	mitochondrial ATP synthesis coupled proton transport	2.80	15.07
NT5E	AMP catabolic process, DNA metabolic process, adenosine biosynthetic process, dephosphorylation, nucleotide catabolic process, purine nucleotide catabolic process, pyrimidine nucleoside catabolic process	0.93	19.37
AKR1C1	bile acid metabolic process, daunorubicin metabolic process, doxorubicin metabolic process, oxidation-reduction process, progesterone metabolic process, retinal metabolic process, xenobiotic metabolic process	4.67	12.91
PRMT1	histone H4-R3 methylation, methylation, peptidyl-arginine methylation, to asymmetrical-dimethyl arginine	6.54	8.61
CBSL	cellular amino acid biosynthetic process, cysteine biosynthetic process, cysteine biosynthetic process from serine, cysteine biosynthetic process via cystathionine	5.60	10.76
FUS	mRNA splicing, via spliceosome	7.47	12.91
PSME1	MAPK cascade, anaphase-promoting complex-dependent catabolic process, protein polyubiquitination	2.80	15.07
TP53I3	NADP metabolic process, oxidation-reduction process	2.80	15.07
RECQL	DNA recombination, DNA strand renaturation, double-strand break repair via homologous recombination	0.93	19.37
RPL23A	nuclear-transcribed mRNA catabolic process, nonsense-mediated decay, rRNA processing, translation, translational initiation, viral transcription	2.80	15.07
TROVE2	transcription from RNA polymerase III promoter	6.54	8.61
SOD1	glutathione metabolic process, hydrogen peroxide biosynthetic process, oxidation-reduction process, removal of superoxide radicals, superoxide anion generation, superoxide metabolic process	4.67	15.07
TARDBP	RNA splicing, mRNA processing, transcription from RNA polymerase II promoter	5.60	10.76
UBA6	cellular protein modification process, protein ubiquitination, protein ubiquitination involved in ubiquitin-dependent protein catabolic process, ubiquitin-dependent protein catabolic process	2.80	15.07
ANXA7	autophagy	4.67	12.91
P3H3	collagen metabolic process, oxidation-reduction process, peptidyl-proline hydroxylation	3.74	15.07
UBA2	protein sumoylation	2.80	17.22
TXNL1	glycerol ether metabolic process, oxidation-reduction process, sulfate assimilation	2.80	15.07
ABHD10	cellular glucuronidation, glucuronoside catabolic process	6.54	8.61
GLRX	nucleobase-containing small molecule interconversion, oxidation-reduction process, protein deglutathionylation	7.47	6.46
ANP32A	transcription, DNA-templated	2.80	19.37
DECR1	fatty acid beta-oxidation, lipid metabolic process, oxidation-reduction process	8.40	4.31
EIF3B	IRES-dependent viral translational initiation, formation of translation preinitiation complex, translation, viral translational termination-reinitiation	1.87	19.37
GFPT2	UDP-N-acetylglucosamine biosynthetic process, carbohydrate derivative biosynthetic process, carbohydrate metabolic process, energy reserve metabolic process, fructose 6-phosphate metabolic process, glutamine metabolic process	11.21	10.76
LGALS3	RNA splicing, mRNA processing	2.80	15.07
ACAA1	alpha-linolenic acid metabolic process, bile acid metabolic process, fatty acid beta-oxidation, fatty acid beta-oxidation using acyl-CoA oxidase, very long-chain fatty acid metabolic process	0.93	19.37
TMX3	peptidyl-cysteine oxidation	3.74	12.91
ACP1	peptidyl-tyrosine dephosphorylation	4.67	10.76
YBX3	transcription, DNA-templated	5.60	19.37
UBE2D2	protein K48-linked ubiquitination, protein autoubiquitination, ubiquitin-dependent protein catabolic process	8.40	4.31
HNRNPH2	gene expression, mRNA splicing, via spliceosome	2.80	15.07

[a]Exponentially Modified Protein Abundance Index (http://www.matrixscience.com/help/quant_empai_help.html).

Table S8. Identification of endogenous proteins (Biological Process: response to stimulus) in hADSC; group CDM & DMEM.

Table S8. Identification of endogenous proteins (Biological Process: response to stimulus) in hADSC; group CDM & DMEM.

UniProt/SWISS-Alternate ID	Biological Process response to stimulus	emPAI[a] CDM	emPAI[a] DMEM
ACTA2	response to virus	285.76	439.10
VIM	SMAD protein signal transduction, axon regeneration, cellular response to estradiol stimulus, cellular response to fibroblast growth factor stimulus, cellular response to oxidative stress, response to acrylamide, response to cocaine, response to estradiol,	588.33	1108.52
TUBB6	axon guidance, natural killer cell mediated cytotoxicity	488.41	682.32
TUBB3	axon guidance	206.38	213.10
TUBB	natural killer cell mediated cytotoxicity	304.44	396.06
TUBB4B	natural killer cell mediated cytotoxicity	286.69	380.98
MYH9	aggregation involved in sorocarp development, axon guidance, cardiac muscle hypertrophy in response to stress, chemotaxis, hypotonic response, integrin-mediated signaling pathway, plasma membrane repair, platelet aggregation, response to hydrogen peroxide, response to mechanical stimulus	300.70	901.88
MYH10	axon guidance, plasma membrane repair	18.68	124.84
FLNA	adenylate cyclase-inhibiting dopamine receptor signaling pathway, platelet aggregation, semaphorin-plexin signaling pathway, wound healing, spreading of cells	223.19	884.66
HSP90AB1	ATF6-mediated unfolded protein response, ERBB2 signaling pathway, Fc-gamma receptor signaling pathway involved in phagocytosis, aggregation involved in sorocarp development, cellular response to ATP, cellular response to drug, cellular response to interleukin-4, cellular response t	439.85	454.16
HSP90B1	ATF6-mediated unfolded protein response, cellular response to ATP, response to endoplasmic reticulum stress, response to hypoxia, response to stress, retrograde protein transport, ER to cytosol, toll-like receptor signaling pathway, ubiquitin-dependent ERAD pathway	87.78	180.81
HSP90AA1	ERBB2 signaling pathway, Fc-gamma receptor signaling pathway involved in phagocytosis, response to antibiotic, response to cocaine, response to cold, response to drug, response to estrogen, response to heat, response to salt stress, response to unfolded pro	229.73	165.74
ALB	cellular oxidant detoxification, cellular response to starvation	698.52	47.35
EEF1A1	calcium-mediated signaling, cellular response to epidermal growth factor stimulus	382.88	376.68
HSPA8	ATF6-mediated unfolded protein response, ER overload response, IRE1-mediated unfolded protein response, PERK-mediated unfolded protein response, activation of signaling protein activity involved in unfolded protein response, axon guidance, cellular heat acclimation, cellular res	303.50	559.64
HSPA1A	cellular heat acclimation, cellular response to heat, cellular response to oxidative stress	15.88	51.66
HSPA2	response to cold, response to heat, response to unfolded protein	19.61	36.59
TUBA1B	cellular response to interleukin-4, mechanosensory behavior, thigmotaxis	330.58	292.74
YWHAQ	small GTPase mediated signal transduction	29.88	60.27
YWHAG	cellular response to insulin stimulus	30.82	47.35
YWHAE	hippo signaling, intracellular signal transduction	42.96	51.66
YWHAH	glucocorticoid receptor signaling pathway	10.27	30.13
ENO1	response to virus	254.94	273.36
ACTN4	MAPK cascade, axon guidance, peroxisome proliferator activated receptor signaling pathway, platelet aggregation, response to hypoxia, retinoic acid receptor signaling pathway	135.41	514.44
ACTN1	platelet aggregation	104.59	370.22
TLN1	IRE1-mediated unfolded protein response, platelet aggregation	131.67	374.52
CLTC	Wnt signaling pathway, planar cell polarity pathway, aggregation involved in sorocarp development, response to osmotic stress, signal transduction	163.42	243.22
CLTCL1	signal transduction	20.55	30.13
P4HB	cellular response to hypoxia, response to endoplasmic reticulum stress, response to reactive oxygen species	136.34	236.78
LDHA	response to cAMP, response to drug, response to estrogen, response to glucose, response to hydrogen peroxide, response to hypoxia, response to nutrient, response to organic cyclic compound	107.39	458.48
EEF2	cellular response to brain-derived neurotrophic factor stimulus, cold acclimation, response to cytokinin, response to endoplasmic reticulum stress, response to estradiol, response to ethanol, response to folic acid, response to hydrogen peroxide, response to ischemia	162.49	189.42
ALDOA	mitotic G2 DNA damage checkpoint	110.19	279.82
ANXA2	response to thyroid hormone	116.73	271.20
PLEC	response to nutrient, wound healing	82.18	284.12
LGALS1	cellular response to glucose stimulus, cellular response to organic cyclic compound, multicellular organismal response to stress, plasma cell differentiation, response to axon injury, response to drug, signal transduction	118.60	180.81
FN1	acute-phase response, cellular response to BMP stimulus, cellular response to angiotensin, cellular response to beta-amyloid, cellular response to glucose stimulus, cellular response to interleukin-1, cellular response to lipopolysaccharide, cellular respo	97.12	251.84
COL1A1	blood coagulation, cellular response to amino acid stimulus, cellular response to epidermal growth factor stimulus, cellular response to fibroblast growth factor stimulus, cellular response to fluoride, cellular response to mechanical stimulus, cellular response to retinoic acid, cellular response to transforming growth	76.58	226.00
SERPINH1	response to unfolded protein	127.94	159.28
IQGAP1	ERK1 and ERK2 cascade, Ras protein signal transduction, cellular response to calcium ion, cellular response to epidermal growth factor stimulus, cellular response to fibroblast growth factor stimulus, cellular response to organic substance, cellular response	101.79	191.57
UBA1	cellular response to DNA damage stimulus	103.66	163.59
FASN	cellular response to interleukin-4	130.74	96.86
PFN1	Wnt signaling pathway, planar cell polarity pathway, cellular response to growth factor stimulus	103.66	157.13
CALR	ATF6-mediated unfolded protein response, cellular response to lithium ion, cellular response to organic substance, cellular senescence, glucocorticoid receptor signaling pathway, response to drug, response to estradiol, response to testosterone	115.80	120.54
PDIA3	response to endoplasmic reticulum stress, signal transduction	84.98	142.06
THBS1	behavioral response to pain, cellular response to growth factor stimulus, cellular response to heat, cellular response to tumor necrosis factor, chronic inflammatory response, immune response, response to calcium ion, response to drug, response to endoplasmic reticulum stress, response to glucose, response to	82.18	148.52
CAP1	signal transduction	98.99	120.54
VCP	ER-associated misfolded protein catabolic process, double-strand break repair, endoplasmic reticulum unfolded protein response, error-free translesion synthesis, retrograde protein transport, ER to cytosol, translesion synthesis, ubiquitin-dependent ERAD path	85.92	133.45
CFL1	Rho protein signal transduction, response to virus	128.87	96.86
GSN	striated muscle atrophy	89.65	99.01
ANXA5	blood coagulation, positive regulation of macromitophagy in response to mitochondrial depolarization, response to organic substance, signal transduction, xenophagy	70.97	159.28
MSN	axon guidance, cellular response to cAMP, cellular response to platelet-derived growth factor stimulus, cellular response to testosterone stimulus, cellular response to thyroid hormone stimulus, phosphatidylinositol-mediated signaling, protein kinase A signaling, sphingosine-1-phosphate signaling pathway	56.03	139.91
EZR	axon guidance, cellular response to cAMP, phosphatidylinositol-mediated signaling, protein kinase A signaling, sphingosine-1-phosphate signaling pathway	14.94	32.29
RDX	cellular response to platelet-derived growth factor stimulus, cellular response to thyroid hormone stimulus, protein kinase A signaling	12.14	34.44
VCL	platelet aggregation	47.63	161.43
HSPD1	B cell cytokine production, MyD88-dependent toll-like receptor signaling pathway, cellular response to heat, detection of misfolded protein, isotype switching to IgG isotypes, response to ATP, response to activity, response to cocaine, response to cold, response to drug,	79.38	81.79
COL6A1	cellular response to amino acid stimulus	75.64	81.79
S100A6	signal transduction	101.79	12.91
GSTP1	cellular oxidant detoxification, cellular response to cell-matrix adhesion, cellular response to epidermal growth factor stimulus, cellular response to glucocorticoid stimulus, cellular response to insulin stimulus, cellular response to lipopolysaccharide, response to L-ascorbic acid, response to amino acid, response to	58.83	124.84
EIF4A1	cellular response to brain-derived neurotrophic factor stimulus, cellular response to selenite ion, response to organic cyclic compound	70.97	86.10
ARF4	epidermal growth factor receptor signaling pathway, response to axon injury, small GTPase mediated signal transduction	79.38	60.27
ARF1	small GTPase mediated signal transduction	46.69	23.68
ARF3	small GTPase mediated signal transduction	40.16	17.22
ARF5	small GTPase mediated signal transduction	15.88	15.07
CAPN2	cellular response to amino acid stimulus, response to hypoxia	75.64	86.10
LRP1	protein kinase C-activating G-protein coupled receptor signaling pathway	46.69	124.84
S100A11	signal transduction	79.38	55.96
KPNB1	Ran protein signal transduction	59.77	79.64
ANXA1	G-protein coupled receptor signaling pathway, coupled to cyclic nucleotide second messenger, adaptive immune response, cell surface receptor signaling pathway, cellular response to glucocorticoid stimulus, cellular response to hydrogen peroxide, granulocyte chemotaxis, inflammatory response, innate immune	51.36	88.25
COL1A2	Rho protein signal transduction, blood coagulation, cellular response to amino acid stimulus, platelet activation, transforming growth factor beta receptor signaling pathway	30.82	118.39
MYOF	cellular response to heat, plasma membrane repair	37.35	111.93
STAT1	JAK-STAT cascade, cellular response to cytokine stimulus, cellular response to insulin stimulus, cellular response to interferon-beta, cellular response to organic cyclic compound, defense response to virus, interferon-gamma-mediated signaling pathway, response to cAMP, response to drug, response to hydroger	56.97	64.57
ANXA6	apoptotic signaling pathway	28.02	114.08
MAP1B	cellular response to growth factor stimulus, cellular response to peptide hormone stimulus, induction of synaptic plasticity by chemical substance, peripheral nervous system axon regeneration, response to axon injury, response to carbohydrate, response to drug, response to estradiol, response to inorganic subst	20.55	139.91
MVP	ERBB signaling pathway	46.69	96.86
MYL9	ephrin receptor signaling pathway, platelet aggregation	40.16	92.56
MYL12A	ephrin receptor signaling pathway, platelet aggregation	31.75	73.18
TXN	response to radiation, response to reactive oxygen species, signal transduction	51.36	79.64
HSPB1	cellular response to vascular endothelial growth factor stimulus, intracellular signal transduction, platelet aggregation, positive regulation of endothelial cell chemotaxis by VEGF-activated vascular endothelial growth factor receptor signaling pathway, response to unfolded protein, response to virus, vascular endoth	42.02	94.71
CLIC1	platelet aggregation, signal transduction	44.83	64.57
DPYSL2	axon guidance, response to amphetamine, response to cocaine, response to drug, signal transduction	42.02	129.15
FLNB	signal transduction	40.16	139.91
PRDX6	cellular oxidant detoxification, response to reactive oxygen species	48.56	64.57
EEF1G	response to virus	44.83	66.73
HADHA	response to drug, response to insulin	42.96	66.73
HYOU1	IRE1-mediated unfolded protein response, cellular response to hypoxia, response to endoplasmic reticulum stress, response to ischemia	34.55	83.95
IDH1	response to organic cyclic compound, response to oxidative stress, response to steroid hormone	38.29	64.57
IPO5	cellular response to amino acid stimulus	31.75	103.32
EPRS	cellular response to interferon-gamma	31.75	73.18
PSAP	adenylate cyclase-inhibiting G-protein coupled receptor signaling pathway, cellular response to organic substance	38.29	55.96
CTSB	cellular response to thyroid hormone stimulus, toll-like receptor signaling pathway	42.02	40.90
HSPA4	response to unfolded protein	31.75	73.18
ACTR3	Fc-gamma receptor signaling pathway involved in phagocytosis, ephrin receptor signaling pathway	31.75	58.12
AARS	cellular response to unfolded protein, endoplasmic reticulum unfolded protein response, response to amino acid	28.02	86.10
TXNDC5	response to endoplasmic reticulum stress	35.49	49.51
PDIA6	IRE1-mediated unfolded protein response, platelet aggregation, response to endoplasmic reticulum stress	37.35	60.27
PRKDC	cellular response to insulin stimulus, double-strand break repair, double-strand break repair via alternative nonhomologous end joining, intrinsic apoptotic signaling pathway in response to DNA damage, response to activity, response to gamma radiation, res	51.36	25.83
UCHL1	response to ischemia	28.95	53.81
CALU	peripheral nervous system axon regeneration, response to organic cyclic compound	28.95	53.81
HSPA9	mitochondrial unfolded protein response	28.95	68.88
NNMT	response to drug, response to organonitrogen compound	32.69	62.42

[a] Exponentially Modified Protein Abundance Index (http://www.matrixscience.com/help/quant_empai_help.html).

Table S8. Cont 1.

UniProt/SWISS-Alternate ID	Biological Process biological regulation	emPAI[a] CDM	emPAI[a] DMEM
RPN2	response to drug	36.42	49.51
MME	cellular response to UV-A, cellular response to UV-B, cellular response to cytokine stimulus	31.75	62.42
RRBP1	signal transduction	22.41	77.49
TGFBI	response to stimulus	39.22	36.59
GNB2	G-protein coupled acetylcholine receptor signaling pathway, Ras protein signal transduction, Wnt signaling pathway, calcium modulating pathway, adenylate cyclase-activating dopamine receptor signaling pathway, cellular response to catecholamine stimulus, cellular response to glucagon stimulus, cellular respons	25.21	73.18
GNB1	G-protein coupled acetylcholine receptor signaling pathway, Ras protein signal transduction, Wnt signaling pathway, calcium modulating pathway, adenylate cyclase-activating dopamine receptor signaling pathway, cellular response to catecholamine stimulus, cellular response to glucagon stimulus, cellular respons	12.14	38.74
GNB4	cellular response to glucagon stimulus, signal transduction	4.67	10.76
PARK7	Ras protein signal transduction, cellular oxidant detoxification, cellular response to glyoxal, cellular response to hydrogen peroxide, cellular response to oxidative stress, cellular response to reactive oxygen species, detoxification of copper ion, detoxification of mercury	30.82	51.66
UGGT1	ER-associated misfolded protein catabolic process, endoplasmic reticulum unfolded protein response	25.21	66.73
GPI	humoral immune response, response to cadmium ion, response to estradiol, response to immobilization stress, response to morphine, response to muscle stretch, response to progesterone, response to testosterone	41.09	40.90
CCT5	response to virus	29.88	58.12
XRCC5	cellular hyperosmotic salinity response, cellular response to DNA damage stimulus, cellular response to X-ray, cellular response to fatty acid, cellular response to gamma radiation, double-strand break repair, double-strand break repair via nonhomologous end joining, response to drug	34.55	43.05
PRDX1	natural killer cell mediated cytotoxicity, removal of superoxide radicals, response to reactive oxygen species	38.29	60.27
G6PD	cellular response to oxidative stress, response to ethanol, response to food, response to iron(III) ion, response to organic cyclic compound	24.28	60.27
PSMD2	Fc-epsilon receptor signaling pathway, MAPK cascade, NIK/NF-kappaB signaling, T cell receptor signaling pathway, Wnt signaling pathway, planar cell polarity pathway, stimulatory C-type lectin receptor signaling pathway, tumor necrosis factor-mediated signaling pathway	26.15	58.12
CLIC4	cellular response to calcium ion	19.61	71.03
RACK1	cellular response to glucose stimulus, cellular response to growth factor stimulus	21.48	71.03
PSMD1	Fc-epsilon receptor signaling pathway, MAPK cascade, NIK/NF-kappaB signaling, T cell receptor signaling pathway, Wnt signaling pathway, planar cell polarity pathway, stimulatory C-type lectin receptor signaling pathway, tumor necrosis factor-mediated signaling pathway	24.28	60.27
PPP2R1A	response to organic substance, second-messenger-mediated signaling	33.62	38.74
GDI2	signal transduction, small GTPase mediated signal transduction	45.76	81.79
GNAI2	G-protein coupled acetylcholine receptor signaling pathway, adaptation of signaling pathway by response to pheromone involved in conjugation with cellular fusion, adenosine receptor signaling pathway, adenylate cyclase-activating G-protein coupled receptor signaling pathway, adenylate cyclase-activating dopam	26.15	55.96
GNAS	adenylate cyclase-activating dopamine receptor signaling pathway, platelet aggregation, response to drug	1.87	17.22
ITGB1	cellular defense response, cellular response to low-density lipoprotein particle stimulus, integrin-mediated signaling pathway, transforming growth factor beta receptor signaling pathway, visual learning	24.28	55.96
MAPK1	B cell receptor signaling pathway, BMP signaling pathway, DNA damage induced protein phosphorylation, ERBB signaling pathway, ERK1 and ERK2 cascade, Fc-epsilon receptor signaling pathway, Fc-gamma receptor signaling pathway involved in phagocytosis, G-protein coupled receptor signa	22.41	34.44
MAPK3	BMP signaling pathway, DNA damage induced protein phosphorylation, ERK1 and ERK2 cascade, Fc-epsilon receptor signaling pathway, Fc-gamma receptor signaling pathway involved in phagocytosis, JAK-STAT cascade involved in growth hormone signaling pathway, MAPK import into nucleus, axo	5.60	10.76
PPP1CA	beta-catenin destruction complex disassembly, entrainment of circadian clock by photoperiod, regulation of translational initiation by eIF2 alpha dephosphorylation	30.82	27.98
PPP1CB	entrainment of circadian clock by photoperiod	25.21	25.83
RAB10	cellular response to insulin stimulus, cellular response to nerve growth factor stimulus, defense response to bacterium, fibroblast growth factor receptor signaling pathway, response to cadmium ion, small GTPase mediated signal transduction	15.88	64.57
RAB35	cellular response to nerve growth factor stimulus, small GTPase mediated signal transduction	5.60	12.91
RAB1B	small GTPase mediated signal transduction	6.54	27.98
RAB1A	defense response to bacterium, small GTPase mediated signal transduction	5.60	32.29
SPTAN1	MAPK cascade, axon guidance	19.61	51.66
ACTR2	Fc-gamma receptor signaling pathway involved in phagocytosis, ephrin receptor signaling pathway	29.88	40.90
ARPC2	Fc-gamma receptor signaling pathway involved in phagocytosis, ephrin receptor signaling pathway	25.21	45.20
CNN2	cellular response to mechanical stimulus	18.68	77.49
GCN1	cellular response to amino acid starvation, cellular response to leucine starvation, cellular response to stress, positive regulation of transcription from RNA polymerase II promoter in response to stress	23.35	55.96
COL6A2	response to glucose	28.02	36.59
MIF	DNA damage response, signal transduction by p53 class mediator, cell surface receptor signaling pathway, inflammatory response, innate immune response, positive chemotaxis	31.75	30.13
VPS35	Wnt signaling pathway	19.61	62.42
PLOD1	response to hypoxia	22.41	43.05
ATP6V1A	insulin receptor signaling pathway	10.27	66.73
VDAC1	behavioral fear response	8.40	90.40
AP2A1	Wnt signaling pathway, planar cell polarity pathway, ephrin receptor signaling pathway	11.21	66.73
AP2A2	Wnt signaling pathway, planar cell polarity pathway, ephrin receptor signaling pathway	4.67	27.98
HNRNPK	signal transduction	25.21	38.74
ZYX	integrin-mediated signaling pathway, signal transduction, transforming growth factor beta receptor signaling pathway	21.48	45.20
TNC	cellular response to prostaglandin D stimulus, cellular response to retinoic acid, cellular response to vitamin D, peripheral nervous system axon regeneration, response to ethanol, response to fibroblast growth factor, response to mechanical stimulus, response to wounding, wound healing	5.60	75.34
PDIA4	response to endoplasmic reticulum stress	26.15	32.29
SEC31A	IRE1-mediated unfolded protein response, response to calcium ion	16.81	49.51
XRCC6	cellular hyperosmotic salinity response, cellular response to X-ray, cellular response to gamma radiation, double-strand break repair via classical nonhomologous end joining	32.69	19.37
AP2B1	Wnt signaling pathway, planar cell polarity pathway, ephrin receptor signaling pathway	20.55	62.42
GSTO1	cellular oxidant detoxification, cellular response to arsenic-containing substance, positive regulation of skeletal muscle contraction by regulation of release of sequestered calcium ion, regulation of cardiac muscle contraction by regulation of the release of sequestered calcium ion, xenobiotic catabolic process	26.15	30.13
NPM1	DNA damage response, signal transduction by p53 class mediator resulting in cell cycle arrest, DNA repair, regulation of eIF2 alpha phosphorylation by dsRNA, signal transduction	26.15	27.98
DDOST	response to cytokine	21.48	36.59
PEBP1	MAPK cascade	20.55	38.74
RPS3	DNA damage response, detection of DNA damage, DNA repair, cellular response to hydrogen peroxide, response to TNF agonist	15.88	51.66
IPO7	innate immune response, signal transduction	18.68	36.59
PRDX4	I-kappaB phosphorylation, cellular oxidant detoxification	20.55	60.27
RHOA	Rho protein signal transduction, Roundabout signaling pathway, Wnt signaling pathway, planar cell polarity pathway, apolipoprotein A-I-mediated signaling pathway, ephrin receptor signaling pathway, negative chemotaxis, phosphatidylinositol-mediated signaling, platelet a	21.48	34.44
RHOC	small GTPase mediated signal transduction, wound healing, spreading of cells	16.81	25.83
EEF1B2	response to ethanol	19.61	43.05
CTSK	toll-like receptor signaling pathway	28.95	12.91
FERMT2	Wnt signaling pathway, integrin-mediated signaling pathway, transforming growth factor beta receptor signaling pathway	13.07	49.51
MYO1C	Fc-gamma receptor signaling pathway involved in phagocytosis, positive regulation of cell migration by vascular endothelial growth factor signaling pathway	17.74	40.90
NME1	cellular response to drug, cellular response to fatty acid, cellular response to glucose stimulus, response to amine, response to cAMP, response to drug, response to testosterone	48.56	36.59
PRDX5	cellular oxidant detoxification, cellular response to reactive oxygen species, inflammatory response, response to oxidative stress	14.01	49.51
RAP1B	Rap protein signal transduction, cellular response to cAMP, cellular response to drug, cellular response to glucose stimulus, cellular response to gonadotropin-releasing hormone, cellular response to nerve growth factor stimulus, cellular response to organic cyclic compound, nerve grow	22.41	32.29
RAP1A	Rap protein signal transduction, cellular response to cAMP, cellular response to drug, cellular response to glucose stimulus, cellular response to nerve growth factor stimulus, cellular response to organic cyclic compound, nerve growth factor signaling pathway, response to antineop	16.81	25.83
SPTBN1	MAPK cascade, SMAD protein import into nucleus, axon guidance, common-partner SMAD protein phosphorylation	14.94	38.74
ARHGAP1	Rho protein signal transduction, small GTPase mediated signal transduction	15.88	36.59
SPARC	cellular response to growth factor stimulus, response to L-ascorbic acid, response to cAMP, response to cadmium ion, response to calcium ion, response to cytokine, response to ethanol, response to glucocorticoid, response to gravity, response to lead ion, response to lipopolysaccharide, response to peptide horm	14.94	43.05
ATP1A1	cellular response to mechanical stimulus, cellular response to steroid hormone stimulus, response to drug, response to glycoside	25.21	17.22
ARHGDIA	semaphorin-plexin signaling pathway	16.81	30.13
PTBP1	fibroblast growth factor receptor signaling pathway	13.07	49.51
ATP6V1B2	insulin receptor signaling pathway	11.21	49.51
PLOD2	cellular response to hormone stimulus, response to hypoxia	12.14	45.20
TXNRD1	cellular oxidant detoxification, response to reactive oxygen species, signal transduction	2.80	66.73
ADH5	response to lipopolysaccharide, response to nitrosative stress, response to redox state	17.74	32.29
COMT	cellular response to phosphate starvation, response to drug, response to lipopolysaccharide, response to organic cyclic compound, response to pain	14.01	34.44
NME2	cellular response to fatty acid, cellular response to glucose stimulus, cellular response to oxidative stress, integrin-mediated signaling pathway, response to growth hormone	45.76	36.59
PLOD3	cellular response to hormone stimulus	13.07	47.35
PSMA4	Fc-epsilon receptor signaling pathway, MAPK cascade, NIK/NF-kappaB signaling, T cell receptor signaling pathway, Wnt signaling pathway, planar cell polarity pathway, stimulatory C-type lectin receptor signaling pathway, tumor necrosis factor-mediated signaling pathway	15.88	38.74
SERPINB6	cellular response to osmotic stress	5.60	55.96
SLC25A6	active induction of host immune response by virus	14.94	25.83
ARPC1B	Fc-gamma receptor signaling pathway involved in phagocytosis, ephrin receptor signaling pathway, response to estrogen	15.88	38.74
C1QBP	adaptive immune response, blood coagulation, intrinsic pathway, complement activation, classical pathway, innate immune response, phosphatidylinositol 3-kinase signaling	18.68	25.83
CSRP1	platelet aggregation	5.60	47.35
GFPT1	IRE1-mediated unfolded protein response	15.88	47.35
EIF4G1	behavioral fear response	21.48	17.22
RAB5C	blood coagulation, receptor internalization involved in canonical Wnt signaling pathway, small GTPase mediated signal transduction	9.34	43.05
CD63	endosome to melanosome transport, pigment granule maturation	20.55	12.91
ATP2A2	ER-nucleus signaling pathway, cardiac muscle hypertrophy in response to stress, cellular response to oxidative stress, regulation of cardiac muscle contraction by calcium ion signaling, response to endoplasmic reticulum stress, response to peptide hormone	7.47	45.20
CORO1C	signal transduction	14.94	25.83
DDX3X	Wnt signaling pathway, cellular response to arsenic-containing substance, cellular response to osmotic stress, extrinsic apoptotic signaling pathway via death domain receptors, innate immune response, intracellular signal transduction, intrinsic apoptotic signaling pathway, response to virus	18.68	23.68
PAPSS2	blood coagulation	14.01	32.29
RAN	androgen receptor signaling pathway, signal transduction, small GTPase mediated signal transduction	15.88	25.83
STIP1	response to stress	11.21	34.44
EEF1D	cellular response to ionizing radiation, signal transduction	19.61	34.44
COL3A1	cellular response to amino acid stimulus, integrin-mediated signaling pathway, platelet activation, response to cytokine, response to mechanical stimulus, response to radiation, transforming growth factor beta receptor signaling pathway	17.74	21.52
PRKCSH	intracellular signal transduction	14.01	30.13

[a]Exponentially Modified Protein Abundance Index (http://www.matrixscience.com/help/quant_empai_help.html).

Table S8. Cont 2.

UniProt/SWISS-Alternate ID	Biological Process<br>biological regulation	emPAI[a] CDM	emPAI[a] DMEM
MRC2	signal transduction	8.40	40.90
PSME2	Fc-epsilon receptor signaling pathway, MAPK cascade, NIK/NF-kappaB signaling, T cell receptor signaling pathway, Wnt signaling pathway, planar cell polarity pathway, stimulatory C-type lectin receptor signaling pathway, tumor necrosis factor-mediated signaling pathway	20.55	12.91
ATIC	response to inorganic substance	10.27	36.59
SDCBP	Ras protein signal transduction, ephrin receptor signaling pathway	14.94	30.13
AKR1B1	cellular response to hydrogen peroxide, cellular response to methylglyoxal, cellular response to peptide, response to organic substance, response to thyroid hormone, response to water deprivation, stress-activated protein kinase signaling cascade	16.81	25.83
DPYSL3	cellular response to cytokine stimulus, response to axon injury	20.55	64.57
PCBP2	defense response to virus, innate immune response	25.21	34.44
PSMC4	Fc-epsilon receptor signaling pathway, MAPK cascade, NIK/NF-kappaB signaling, T cell receptor signaling pathway, Wnt signaling pathway, planar cell polarity pathway, stimulatory C-type lectin receptor signaling pathway, tumor necrosis factor-mediated signaling pathway, ubiquitin-dependent ERAD pathway	13.07	25.83
XPO1	response to drug	16.81	15.07
ITGAV	ERK1 and ERK2 cascade, apolipoprotein A-I-mediated signaling pathway, extrinsic apoptotic signaling pathway in absence of ligand, integrin-mediated signaling pathway, negative chemotaxis, vascular endothelial growth factor receptor signaling pathway	3.74	43.05
EHD2	blood coagulation	9.34	36.59
EIF2S1	PERK-mediated unfolded protein response, cellular response to UV, cellular response to amino acid starvation, cellular response to heat, negative regulation of translational initiation in response to stress, positive regulation of macromitophagy in respons	10.27	34.44
PABPC4	blood coagulation	17.74	25.83
PSMA7	Fc-epsilon receptor signaling pathway, MAPK cascade, NIK/NF-kappaB signaling, T cell receptor signaling pathway, Wnt signaling pathway, planar cell polarity pathway, stimulatory C-type lectin receptor signaling pathway, tumor necrosis factor-mediated signaling pathway	14.94	19.37
RPL3	cellular response to interleukin-4	14.94	27.98
YARS	signal transduction	10.27	32.29
ERO1A	cellular response to hypoxia, endoplasmic reticulum unfolded protein response, intrinsic apoptotic signaling pathway in response to endoplasmic reticulum stress, response to reactive oxygen species, response to temperature stimulus	17.74	12.91
PSMD3	Fc-epsilon receptor signaling pathway, MAPK cascade, NIK/NF-kappaB signaling, T cell receptor signaling pathway, Wnt signaling pathway, planar cell polarity pathway, stimulatory C-type lectin receptor signaling pathway, tumor necrosis factor-mediated signaling pathway	8.40	36.59
RSU1	signal transduction	13.07	23.68
TMED10	response to alkaloid	12.14	21.52
MTPN	cellular response to mechanical stimulus, skeletal muscle tissue regeneration	18.68	10.76
POSTN	bone regeneration, cellular response to fibroblast growth factor stimulus, cellular response to transforming growth factor beta stimulus, cellular response to tumor necrosis factor, cellular response to vitamin K, response to estradiol, response to hypoxia, response to mechanical stimulus, response to muscle activi	14.01	23.68
RAD23B	global genome nucleotide-excision repair, nucleotide-excision repair, DNA damage recognition, nucleotide-excision repair, DNA duplex unwinding, nucleotide-excision repair, preincision complex assembly	12.14	23.68
UBE2L3	cellular response to glucocorticoid stimulus, cellular response to steroid hormone stimulus	15.88	17.22
CAPZA1	blood coagulation, innate immune response	15.88	23.68
ELOC	regulation of transcription from RNA polymerase II promoter in response to hypoxia	9.34	25.83
KIF5B	axon guidance	6.54	32.29
PSMA6	Fc-epsilon receptor signaling pathway, MAPK cascade, NIK/NF-kappaB signaling, T cell receptor signaling pathway, Wnt signaling pathway, planar cell polarity pathway, stimulatory C-type lectin receptor signaling pathway, tumor necrosis factor-mediated signaling pathway	11.21	23.68
RAB7A	small GTPase mediated signal transduction	7.47	32.29
HSPE1	response to unfolded protein	16.81	10.76
SLC3A2	response to exogenous dsRNA	1.87	43.05
GDI1	Rab protein signal transduction, response to calcium ion, signal transduction	28.95	55.96
PRKACA	adenylate cyclase-modulating G-protein coupled receptor signaling pathway, blood coagulation, calcium-mediated signaling using intracellular calcium source, cellular response to epinephrine stimulus, cellular response to glucagon stimulus, cellular respons	8.40	19.37
PRKACB	adenylate cyclase-modulating G-protein coupled receptor signaling pathway, blood coagulation, cellular response to glucagon stimulus, response to clozapine, stimulatory C-type lectin receptor signaling pathway	8.40	15.07
PSMB2	Fc-epsilon receptor signaling pathway, MAPK cascade, NIK/NF-kappaB signaling, T cell receptor signaling pathway, Wnt signaling pathway, planar cell polarity pathway, response to organic cyclic compound, response to organonitrogen compound, stimulatory C-type lectin receptor signaling pathway, tumor necrosis	13.07	15.07
HNRNPA1	fibroblast growth factor receptor signaling pathway	8.40	21.52
UBE2V1	DNA double-strand break processing, Fc-epsilon receptor signaling pathway, T cell receptor signaling pathway, double-strand break repair via nonhomologous end joining, error-free postreplication DNA repair, global genome nucleotide-excision repair, nucleot	9.34	23.68
UBE2V2	DNA double-strand break processing, double-strand break repair via nonhomologous end joining, error-free postreplication DNA repair, global genome nucleotide-excision repair	6.54	19.37
HINT1	intrinsic apoptotic signaling pathway by p53 class mediator	7.47	19.37
CTNNA1	axon regeneration, cellular response to indole-3-methanol, prepulse inhibition, response to estrogen, response to organic cyclic compound	0.93	45.20
FKBP1A	SMAD protein complex assembly, transforming growth factor beta receptor signaling pathway	10.27	21.52
RALA	Ras protein signal transduction, chemotaxis	11.21	15.07
TPT1	response to virus	10.27	25.83
ANXA4	Notch signaling pathway, signal transduction	2.80	40.90
ARPC4	Fc-gamma receptor signaling pathway involved in phagocytosis, ephrin receptor signaling pathway	10.27	17.22
DCTN1	IRE1-mediated unfolded protein response	9.34	23.68
DDX1	double-strand break repair, response to exogenous dsRNA, response to virus	5.60	32.29
ENG	BMP signaling pathway, cell chemotaxis, cellular response to mechanical stimulus, detection of hypoxia, response to corticosteroid, response to hypoxia, response to transforming growth factor beta, transforming growth factor beta receptor signaling pathway, wound healing	1.87	32.29
SHMT2	response to type I interferon	10.27	21.52
LAMP1	granzyme-mediated apoptotic signaling pathway	10.27	17.22
PAK2	Fc-epsilon receptor signaling pathway, MAPK cascade, T cell receptor signaling pathway, cellular response to drug, cellular response to heat, cellular response to organic cyclic compound, cellular response to starvation, filamentous growth of a population of unicellular organisms in response to biotic stimulus, filam	12.14	10.76
PFKL	response to glucose	13.07	23.68
PSMC3	Fc-epsilon receptor signaling pathway, MAPK cascade, NIK/NF-kappaB signaling, T cell receptor signaling pathway, Wnt signaling pathway, planar cell polarity pathway, stimulatory C-type lectin receptor signaling pathway, tumor necrosis factor-mediated signaling pathway, ubiquitin-dependent ERAD pathway	3.74	36.59
NPEPPS	cellular response to hypoxia	13.07	12.91
RAP1GDS1	small GTPase mediated signal transduction	3.74	25.83
FABP5	response to wounding	13.07	12.91
PSMA5	Fc-epsilon receptor signaling pathway, MAPK cascade, NIK/NF-kappaB signaling, T cell receptor signaling pathway, Wnt signaling pathway, planar cell polarity pathway, stimulatory C-type lectin receptor signaling pathway, tumor necrosis factor-mediated signaling pathway	0.93	38.74
GOT1	Notch signaling pathway, cellular response to insulin stimulus, response to glucocorticoid	4.67	32.29
COL5A1	wound healing, spreading of epidermal cells	3.74	34.44
CTHRC1	Wnt signaling pathway, planar cell polarity pathway	6.54	30.13
HNRNPM	fibroblast growth factor receptor signaling pathway	9.34	21.52
PSMD14	Fc-epsilon receptor signaling pathway, MAPK cascade, NIK/NF-kappaB signaling, T cell receptor signaling pathway, Wnt signaling pathway, planar cell polarity pathway, double-strand break repair via homologous recombination, double-strand break repair via nonhomologous end joining, response to ethanol, stimula	12.14	10.76
SEC61A1	IRE1-mediated unfolded protein response, response to interferon-gamma	14.94	10.76
AHCY	chronic inflammatory response to antigenic stimulus, response to hypoxia, response to nutrient	12.14	19.37
TPM1	cellular response to reactive oxygen species, wound healing	5.60	103.32
PSMB5	Fc-epsilon receptor signaling pathway, MAPK cascade, NIK/NF-kappaB signaling, T cell receptor signaling pathway, Wnt signaling pathway, planar cell polarity pathway, response to oxidative stress, stimulatory C-type lectin receptor signaling pathway, tumor necrosis factor-mediated signaling pathway	6.54	27.98
AP2M1	Wnt signaling pathway, planar cell polarity pathway, ephrin receptor signaling pathway	8.40	12.91
ARPC3	Fc-gamma receptor signaling pathway involved in phagocytosis, cellular response to nerve growth factor stimulus, ephrin receptor signaling pathway	8.40	19.37
ASNS	PERK-mediated unfolded protein response, cellular response to glucose starvation, cellular response to hormone stimulus, response to amino acid, response to follicle-stimulating hormone, response to light stimulus, response to mechanical stimulus, response t	0.93	36.59
PPP1R14B	innate immune response	15.88	2.15
PSMA2	Fc-epsilon receptor signaling pathway, MAPK cascade, NIK/NF-kappaB signaling, T cell receptor signaling pathway, Wnt signaling pathway, planar cell polarity pathway, response to virus, stimulatory C-type lectin receptor signaling pathway, tumor necrosis factor-mediated signaling pathway	14.94	8.61
PSMB4	Fc-epsilon receptor signaling pathway, MAPK cascade, NIK/NF-kappaB signaling, T cell receptor signaling pathway, Wnt signaling pathway, planar cell polarity pathway, stimulatory C-type lectin receptor signaling pathway, tumor necrosis factor-mediated signaling pathway	7.47	17.22
PSMD13	Fc-epsilon receptor signaling pathway, MAPK cascade, NIK/NF-kappaB signaling, T cell receptor signaling pathway, Wnt signaling pathway, planar cell polarity pathway, stimulatory C-type lectin receptor signaling pathway, tumor necrosis factor-mediated signaling pathway	13.07	12.91
CAD	cellular response to drug, cellular response to epidermal growth factor stimulus, response to amine, response to caffeine, response to cortisol, response to insulin, response to starvation, response to testosterone	8.40	15.07
PSMC2	Fc-epsilon receptor signaling pathway, MAPK cascade, NIK/NF-kappaB signaling, T cell receptor signaling pathway, Wnt signaling pathway, planar cell polarity pathway, stimulatory C-type lectin receptor signaling pathway, tumor necrosis factor-mediated signaling pathway, ubiquitin-dependent ERAD pathway	7.47	23.68
APRT	cellular response to insulin stimulus	10.27	10.76
CDC42	Fc-gamma receptor signaling pathway involved in phagocytosis, Wnt signaling pathway, planar cell polarity pathway, blood coagulation, canonical Wnt signaling pathway, ephrin receptor signaling pathway, small GTPase mediated signal transduction, vascular endothelial growth factor receptor signaling pathway	10.27	17.22
TUFM	response to ethanol	10.27	12.91
MMP14	response to estrogen, response to hormone, response to hypoxia, response to mechanical stimulus, response to organic cyclic compound, response to oxidative stress	0.93	34.44
PPP2CA	response to organic substance, second-messenger-mediated signaling	4.67	12.91
TWF2	cellular response to growth factor stimulus, cellular response to retinoic acid	11.21	12.91
EHD1	blood coagulation, cellular response to nerve growth factor stimulus	5.60	27.98
DPP4	behavioral fear response, response to hypoxia	0.93	36.59
CAPZA2	blood coagulation, innate immune response	7.47	30.13
CDC37	ERBB2 signaling pathway, positive regulation of macromitophagy in response to mitochondrial depolarization	4.67	21.52
CPNE1	cellular response to calcium ion	12.14	10.76
KHSRP	cellular response to cytokine stimulus	6.54	10.76
HNRNPF	fibroblast growth factor receptor signaling pathway	5.60	32.29
PHB	cellular response to interleukin-6, progesterone receptor signaling pathway, signal transduction	9.34	12.91
PNP	immune response, response to drug	4.67	27.98
PSMC5	Fc-epsilon receptor signaling pathway, MAPK cascade, NIK/NF-kappaB signaling, T cell receptor signaling pathway, Wnt signaling pathway, planar cell polarity pathway, stimulatory C-type lectin receptor signaling pathway, tumor necrosis factor-mediated signaling pathway, ubiquitin-dependent ERAD pathway	7.47	17.22
PSMB3	Fc-epsilon receptor signaling pathway, MAPK cascade, NIK/NF-kappaB signaling, T cell receptor signaling pathway, Wnt signaling pathway, planar cell polarity pathway, stimulatory C-type lectin receptor signaling pathway, tumor necrosis factor-mediated signaling pathway	8.40	17.22
CCDC47	ER overload response, ubiquitin-dependent ERAD pathway	5.60	19.37
UBE2N	DNA double-strand break processing, Fc-epsilon receptor signaling pathway, T cell receptor signaling pathway, double-strand break repair via homologous recombination, double-strand break repair via nonhomologous end joining, global genome nucleotide-excisi	9.34	15.07
AKAP12	G-protein coupled receptor signaling pathway, signal transduction	7.47	12.91
FHL2	androgen receptor signaling pathway, response to hormone	3.74	23.68
GBA	cell death in response to oxidative stress, cellular response to starvation, cellular response to tumor necrosis factor, response to estrogen, response to glucocorticoid, response to pH, response to testosterone, response to thyroid hormone	7.47	17.22
SYNCRIP	cellular response to interferon-gamma	3.74	17.22
ILK	integrin-mediated signaling pathway, platelet aggregation, protein kinase B signaling	3.74	23.68
IMPDH2	cellular response to interleukin-4	7.47	19.37

[a]Exponentially Modified Protein Abundance Index (http://www.matrixscience.com/help/quant_empai_help.html).

Table S8. Cont 3.

UniProt/SWISS-Alternate ID	Biological Process biological regulation	emPAI[a] CDM	DMEM
PCNA	DNA damage response, detection of DNA damage, DNA damage response, signal transduction by p53 class mediator resulting in cell cycle arrest, cellular response to UV, cellular response to hydrogen peroxide, error-free translesion synthesis, error-prone translesion synthesis, mismatch repair, nucle	9.34	10.76
PSMB6	Fc-epsilon receptor signaling pathway, MAPK cascade, NIK/NF-kappaB signaling, T cell receptor signaling pathway, Wnt signaling pathway, planar cell polarity pathway, stimulatory C-type lectin receptor signaling pathway, tumor necrosis factor-mediated signaling pathway	8.40	15.07
PSMD7	Fc-epsilon receptor signaling pathway, MAPK cascade, NIK/NF-kappaB signaling, T cell receptor signaling pathway, Wnt signaling pathway, planar cell polarity pathway, stimulatory C-type lectin receptor signaling pathway, tumor necrosis factor-mediated signaling pathway	4.67	19.37
TFG	signal transduction	3.74	17.22
TSPO	behavioral response to pain, cellular hypotonic response, cellular response to lipopolysaccharide, cellular response to zinc ion, peripheral nervous system axon regeneration, response to axon injury, response to drug, response to manganese ion, response to	9.34	8.61
SERPINE1	cellular response to lipopolysaccharide, defense response to Gram-negative bacterium	4.67	21.52
ECM29	ubiquitin-dependent ERAD pathway	6.54	12.91
FBLN1	blood coagulation, fibrin clot formation, integrin-mediated signaling pathway	4.67	23.68
PPP3CA	Fc-epsilon receptor signaling pathway, Wnt signaling pathway, calcium modulating pathway, calcineurin-NFAT signaling cascade, calcium-mediated signaling, cardiac muscle hypertrophy in response to stress, cellular response to drug, cellular response to glucose stimulus, excitatory postsynaptic potential, multice	1.87	17.22
COTL1	defense response to fungus	10.27	8.61
CTNND1	Wnt signaling pathway	4.67	21.52
DNAJB11	IRE1-mediated unfolded protein response	6.54	15.07
DNM1L	release of cytochrome c from mitochondria	5.60	17.22
GPNMB	positive chemotaxis	12.14	4.31
PEA15	DNA damage checkpoint, MAPK cascade, response to morphine	6.54	19.37
PRDX2	cellular response to oxidative stress, removal of superoxide radicals, response to oxidative stress, response to reactive oxygen species	11.21	19.37
PSMB1	Fc-epsilon receptor signaling pathway, MAPK cascade, NIK/NF-kappaB signaling, T cell receptor signaling pathway, Wnt signaling pathway, planar cell polarity pathway, stimulatory C-type lectin receptor signaling pathway, tumor necrosis factor-mediated signaling pathway	6.54	17.22
SCFD1	response to hypoxia, response to toxic substance	8.40	12.91
TXNDC17	cellular oxidant detoxification, tumor necrosis factor-mediated signaling pathway	5.60	21.52
RPS15A	response to virus	5.60	19.37
MCTS1	cellular response to DNA damage stimulus	9.34	12.91
PSMB7	Fc-epsilon receptor signaling pathway, MAPK cascade, NIK/NF-kappaB signaling, T cell receptor signaling pathway, Wnt signaling pathway, planar cell polarity pathway, stimulatory C-type lectin receptor signaling pathway, tumor necrosis factor-mediated signaling pathway	9.34	12.91
PSMC6	Fc-epsilon receptor signaling pathway, MAPK cascade, NIK/NF-kappaB signaling, T cell receptor signaling pathway, Wnt signaling pathway, planar cell polarity pathway, stimulatory C-type lectin receptor signaling pathway, tumor necrosis factor-mediated signaling pathway, ubiquitin-dependent ERAD pathway	7.47	10.76
RAB2A	small GTPase mediated signal transduction	3.74	19.37
ITGB5	integrin-mediated signaling pathway, transforming growth factor beta receptor signaling pathway	3.74	23.68
EIF6	response to insulin	7.47	10.76
PPP1R12A	cellular response to drug, signal transduction	5.60	15.07
SH3GL1	signal transduction	11.21	4.31
CNPY2	enzyme linked receptor protein signaling pathway	5.60	15.07
CYCS	intrinsic apoptotic signaling pathway, response to reactive oxygen species	12.14	4.31
ENAH	axon guidance	4.67	19.37
GSS	response to amino acid, response to cadmium ion, response to nutrient levels, response to oxidative stress, response to tumor necrosis factor, response to xenobiotic stimulus	4.67	19.37
HADH	response to activity, response to drug, response to hormone, response to insulin	5.60	15.07
OTUB1	DNA repair, adaptive immune response, cellular response to interleukin-1	6.54	12.91
VASP	axon guidance	3.74	12.91
KPNA4	response to hydrogen peroxide	9.34	15.07
FSTL1	BMP signaling pathway, response to starvation	9.34	10.76
STAT2	JAK-STAT cascade, defense response to virus, signal transduction, type I interferon signaling pathway	7.47	6.46
ASPH	cellular response to calcium ion, detection of calcium ion, regulation of cardiac muscle contraction by regulation of the release of sequestered calcium ion, response to ATP	10.27	2.15
ACSL3	response to nutrient, response to organic cyclic compound	11.21	6.46
APEX1	base-excision repair, base-free sugar-phosphate removal, cellular response to DNA damage stimulus, cellular response to cAMP, cellular response to hydrogen peroxide, cellular response to organonitrogen compound, cellular response to peptide hormone stimulus, response to drug	12.14	2.15
ARF6	small GTPase mediated signal transduction	5.60	15.07
DDB1	DNA damage response, detection of DNA damage, UV-damage excision repair, Wnt signaling pathway, global genome nucleotide-excision repair, nucleotide-excision repair, DNA damage recognition, nucleotide-excision repair, DNA duplex unwinding, nucleotide-excis	4.67	17.22
DNAJB4	response to heat, response to unfolded protein	6.54	12.91
ENPP1	cellular response to insulin stimulus, immune response	6.54	12.91
GSTK1	cellular oxidant detoxification	6.54	15.07
HEXA	SMAD protein signal transduction	6.54	12.91
LMAN1	blood coagulation	5.60	12.91
POR	cellular response to follicle-stimulating hormone stimulus, cellular response to gonadotropin stimulus, cellular response to peptide hormone stimulus, response to drug, response to nutrient, xenobiotic metabolic process	9.34	6.46
PDLIM1	response to hypoxia, response to oxidative stress	3.74	19.37
PRDX3	cellular oxidant detoxification, cellular response to oxidative stress, cellular response to reactive oxygen species, response to hydrogen peroxide, response to lipopolysaccharide, response to oxidative stress	7.47	12.91
PSMD4	Fc-epsilon receptor signaling pathway, MAPK cascade, NIK/NF-kappaB signaling, T cell receptor signaling pathway, Wnt signaling pathway, planar cell polarity pathway, stimulatory C-type lectin receptor signaling pathway, tumor necrosis factor-mediated signaling pathway	4.67	19.37
GART	response to inorganic substance, response to organic substance	5.60	10.76
SKP1	Fc-epsilon receptor signaling pathway, NIK/NF-kappaB signaling, T cell receptor signaling pathway, Wnt signaling pathway, stimulatory C-type lectin receptor signaling pathway, stress-activated MAPK cascade	3.74	21.52
TAX1BP3	Rho protein signal transduction, Wnt signaling pathway	1.87	23.68
UBE2K	cellular response to interferon-beta, intrinsic apoptotic signaling pathway in response to endoplasmic reticulum stress	7.47	12.91
ATP6V1E1	insulin receptor signaling pathway	5.60	12.91
AP3B1	blood coagulation	4.67	12.91
CD81	cell surface receptor signaling pathway, cellular response to low-density lipoprotein particle stimulus	5.60	17.22
MGST1	cellular oxidant detoxification, cellular response to lipid hydroperoxide, response to drug, response to lipopolysaccharide, response to organonitrogen compound, xenobiotic metabolic process	8.40	10.76
CTPS1	response to drug	7.47	12.91
RAC1	Fc-epsilon receptor signaling pathway, Fc-gamma receptor signaling pathway involved in phagocytosis, G-protein coupled receptor signaling pathway, Wnt signaling pathway, planar cell polarity pathway, axon guidance, blood coagulation, cellular response to mechanical stimulus, ephrin receptor signaling pathway,	7.47	15.07
GPX1	UV protection, angiogenesis involved in wound healing, cellular oxidant detoxification, cellular response to oxidative stress, intrinsic apoptotic signaling pathway in response to oxidative stress, response to gamma radiation, response to hydrogen peroxide, response to hyd	3.74	12.91
CTNNB1	Wnt signaling pathway, calcium modulating pathway, androgen receptor signaling pathway, beta-catenin destruction complex disassembly, canonical Wnt signaling pathway, canonical Wnt signaling pathway involved in midbrain dopaminergic neuron differentiation, canonical Wnt signaling pathway involved in negati	0.93	21.52
CARM1	DNA damage response, signal transduction by p53 class mediator resulting in cell cycle arrest, intracellular estrogen receptor signaling pathway, response to cAMP	3.74	17.22
ERP44	response to endoplasmic reticulum stress, response to unfolded protein	6.54	12.91
HNRNPH1	fibroblast growth factor receptor signaling pathway	7.47	25.83
LIMS1	cellular response to transforming growth factor beta stimulus	6.54	12.91
ME1	response to carbohydrate, response to hormone	3.74	17.22
PSMD11	Fc-epsilon receptor signaling pathway, MAPK cascade, NIK/NF-kappaB signaling, T cell receptor signaling pathway, Wnt signaling pathway, planar cell polarity pathway, stimulatory C-type lectin receptor signaling pathway, tumor necrosis factor-mediated signaling pathway	3.74	19.37
PSMD12	Fc-epsilon receptor signaling pathway, MAPK cascade, NIK/NF-kappaB signaling, T cell receptor signaling pathway, Wnt signaling pathway, planar cell polarity pathway, stimulatory C-type lectin receptor signaling pathway, tumor necrosis factor-mediated signaling pathway	3.74	12.91
PTK7	canonical Wnt signaling pathway, cellular response to retinoic acid, planar cell polarity pathway involved in neural tube closure, signal transduction, wound healing	0.93	21.52
TGFB1I1	Wnt signaling pathway, androgen receptor signaling pathway, response to heat, ubiquitin-dependent SMAD protein catabolic process	1.87	23.68
TRIM28	DNA repair, Ras protein signal transduction, innate immune response	6.54	10.76
VPS29	Wnt signaling pathway	6.54	8.61
SSR1	IRE1-mediated unfolded protein response	4.67	17.22
PCK2	cellular response to glucose stimulus, cellular response to tumor necrosis factor, response to dexamethasone, response to lipopolysaccharide	1.87	23.68
DNAJA1	DNA damage response, detection of DNA damage, androgen receptor signaling pathway, response to heat, response to unfolded protein	7.47	8.61
EXOC1	phosphatidylinositol-mediated signaling	9.34	2.15
ADSL	response to hypoxia, response to muscle activity, response to nutrient, response to starvation	2.80	17.22
PTGES3	signal transduction	3.74	17.22
DNAJA2	response to heat	7.47	8.61
STOML2	T cell receptor signaling pathway, stress-induced mitochondrial fusion	3.74	12.91
MACF1	Wnt signaling pathway, wound healing	4.67	12.91
PSMD6	Fc-epsilon receptor signaling pathway, MAPK cascade, NIK/NF-kappaB signaling, T cell receptor signaling pathway, Wnt signaling pathway, planar cell polarity pathway, stimulatory C-type lectin receptor signaling pathway, tumor necrosis factor-mediated signaling pathway	7.47	6.46
AKR1C1	cellular response to jasmonic acid stimulus, response to organophosphorus, xenobiotic metabolic process	4.67	12.91
PRMT1	DNA damage response, signal transduction by p53 class mediator resulting in cell cycle arrest, cell surface receptor signaling pathway	6.54	8.61
ANXA11	response to calcium ion	0.93	21.52
FUS	cellular response to calcium ion	7.47	12.91
GNAQ	G-protein coupled acetylcholine receptor signaling pathway, adenylate cyclase-activating G-protein coupled receptor signaling pathway, blood coagulation, cellular response to pH, entrainment of circadian clock, glutamate receptor signaling pathway, phospholipase C-activating dopamine receptor signaling pathway	3.74	12.91
PRKAR1A	blood coagulation, cellular response to glucagon stimulus, intracellular signal transduction	5.60	10.76
MESD	Wnt signaling pathway	6.54	8.61
PSME1	Fc-epsilon receptor signaling pathway, MAPK cascade, NIK/NF-kappaB signaling, T cell receptor signaling pathway, Wnt signaling pathway, planar cell polarity pathway, stimulatory C-type lectin receptor signaling pathway, tumor necrosis factor-mediated signaling pathway	2.80	15.07
RECQL	double-strand break repair via homologous recombination	0.93	19.37
TROVE2	response to UV, smoothened signaling pathway	6.54	8.61
SOD1	cellular response to ATP, cellular response to cadmium ion, cellular response to oxidative stress, cellular response to potassium ion, removal of superoxide radicals, response to amphetamine, response to antibiotic, response to antipsychotic drug, response to axon injury, response to carbon monoxide, response t	4.67	15.07
SRP72	response to drug	3.74	15.07
SRPRB	IRE1-mediated unfolded protein response, small GTPase mediated signal transduction	2.80	15.07
TARDBP	response to endoplasmic reticulum stress	5.60	10.76
ANXA7	response to calcium ion, response to organic cyclic compound, response to salt stress	4.67	12.91

[a]Exponentially Modified Protein Abundance Index (http://www.matrixscience.com/help/quant_empai_help.html).

Table S8. Cont 4.

UniProt/SWISS-Alternate ID	Biological Process biological regulation	emPAI[a] CDM	DMEM
TXNL1	cellular response to oxidative stress	2.80	15.07
ANP32A	intracellular signal transduction	2.80	19.37
LGALS3	eosinophil chemotaxis, innate immune response, macrophage chemotaxis, monocyte chemotaxis, neutrophil chemotaxis, positive chemotaxis	2.80	15.07
PARVA	smooth muscle cell chemotaxis	4.67	10.76
SRPRA	IRE1-mediated unfolded protein response	4.67	10.76
TMX3	response to endoplasmic reticulum stress	3.74	12.91
YBX3	cellular hyperosmotic response, cellular response to tumor necrosis factor, response to cold	5.60	19.37
UBE2D2	Fc-epsilon receptor signaling pathway, T cell receptor signaling pathway, TRIF-dependent toll-like receptor signaling pathway, regulation of transcription from RNA polymerase II promoter in response to hypoxia, stimulatory C-type lectin receptor signaling pathway	8.40	4.31
ARPC1A	Fc-gamma receptor signaling pathway involved in phagocytosis, ephrin receptor signaling pathway	3.74	17.22

[a]Exponentially Modified Protein Abundance Index (http://www.matrixscience.com/help/quant_empai_help.html).

Table S9. Primer sequences for quantitative real-time PCR.

Table S9. Primer sequences for quantitative real-time PCR.

Gnens	Accession No.	Sequences (5'→ 3')	Length (bp)
human ASS1	NM_000050.4	Forward: CATCCTTTACCATGCTCATTTAGAC	250
		Reverse: ATTGTAGAGAGACAGTGGGGACTC	
human ITGA5	NM_002205.4	Forward: CTGCTACCTCTCCACAGATAACTTC	229
		Reverse: GATCAGGTACTCGGGGTAATAAGAT	
human p65	XM_011545207.2	Forward: GGAGAAACGTAAAAGGACATATGAG	193
		Reverse: AAACTCATCATAGTTGATGGTGCTC	
human p50	NM_001165412.1	Forward: GAGCCAGAGTTTACATCTGATGATT	164
		Reverse: AAGGCATTATTAAGTATCCCCAGAC	
human cyclin D1	NM_053056.2	Forward: CCCCTTGATTTAAACACACAGATAC	235
		Reverse: AGGTTGAGTACCCTAATTTTCCTTG	
human CD29	NM_002211.3	Forward: CTGAAGACTATCCCATTGACCTCTA	179
		Reverse: GCTAATGTAAGGCATCACAGTCTTT	
human CD34	NM_001025109.1	Forward: CCTGCTCTCTTGTAATGATATAGCC	227
		Reverse: GAGACTAGAACTGAGCTGTTTGTCC	
human CD44	NM_000610.3	Forward: ACTAGTGTTCAAGTGCCTCTTGTTT	227
		Reverse: GCCTCTTTTTGGGAATATCTAGAAG	
human CD45	NM_001267798.1	Forward: TTCTTAGGGTAACAGAGGAGGAAAT	167
		Reverse: ACAAATACTTCTGTGTCCAGAAAGG	
human CD90	NM_001311160.1	Forward: CAATCGGAGATCACTATGTCTTTCT	177
		Reverse: ACTGCTTCATATACGTGAGACACAA	
human Ki67	NM_001145966.1	Forward: AACACCTACAAAATGACTTCTCTGG	204
		Reverse: TAGTAGTGTTGCCTTCTGACCTCTT	
human PCNA	NM_002592.2	Forward: CAAGTAATGTCGATAAAGAGGAGGA	207
		Reverse: TGGGAGCCAAGTAGTATTTTAAGTG	
human PPARγ	NM_001330615.1	Forward: AGCATTTCTACTCCACATTACGAAG	169
		Reverse: CATGAGGCTTATTGTAGAGCTGAGT	
human FABP4	NM_001442.2	Forward: AAGTCAAGAGCACCATAACCTTAGA	217
		Reverse: CCACAGAATGTTGTAGAGTTCAATG	
human C/EBPα	NM_001285829.1	Forward: GTTTGTACTGTATGCCTTCAGCATT	248
		Reverse: AGAGTCTCATTTTGGCAAGTATCC	
human β-actin	NM_001101	Forward: TGGCACCCAGCACAATGAA	186
		Reverse: CTAAGTCATAGTCCGCCTAGAAGCA	
